# Optimal prophylaxis for perioperative drug-induced delayed neurocognitive recovery: a Bayesian network meta-analysis of 18 interventions with mechanism-based stratification

**DOI:** 10.3389/fphar.2026.1877360

**Published:** 2026-09-23

**Authors:** Yaqing Liu, Yuqing Yao, Xincai Wang, Wenxue Fan, Xiuling Shang

**Affiliations:** 1 Shengli Clinical Medical College of Fujian Medical University, Fuzhou, China; 2 Department of Critical Care Medicine, Fuzhou University Affiliated Provincial Hospital, Fujian Provincial Hospital, Shengli Clinical Medical College of Fujian Medical University, Fujian Provincial Center for Critical Care Medicine, Fujian Provincial Key Laboratory of Critical Care Medicine, Fuzhou, China

**Keywords:** dexmedetomidine, meta-analysis, perioperative medicine network, postoperative delayed neurocognitive recovery, propofol, ulinastatin

## Abstract

**Background:**

Postoperative delayed neurocognitive recovery (dNCR) is a common and serious perioperative complication. Although there are various drugs available for prevention, there is a lack of efficacy comparison and significant controversy exists.

**Methods:**

Systematic searches were conducted in PubMed, Embase, Cochrane Library, Web of Science, World Health Organization International Clinical Trials Registry Platform (WHO ICTRP), and ClinicalTrials.gov databases from the establishment of the databases until July 2025. Randomized controlled trials (RCTs) evaluating drug interventions for preventing dNCR were included. According to the clinical mechanism of action, the intervention measures were divided into three independent networks: anesthetic maintenance drugs, adjunctive sedation and neuroprotective agents, and analgesics. Bayesian network meta-analysis was used for each of the three networks to integrate direct and indirect evidence for efficacy comparison and ranking. The effect size was reported as the odds ratio (OR) and its 95% credible interval (CrI). The quality of evidence was evaluated using the CINeMA framework.

**Results:**

A total of 69 randomized controlled trials were included, involving 9,636 patients, and 18 intervention measures (including the saline control group) were evaluated. The anesthesia maintenance drug network: Propofol (SUCRA = 0.908) was the most effective in preventing dNCR, outperforming sevoflurane (OR 0.60, 95% CrI 0.44–0.82) and midazolam (OR 0.21, 95% CrI 0.08–0.51). The adjunctive sedation and neuroprotective drug network: Dexmedetomidine (SUCRA = 0.887) and ulinastatin (SUCRA = 0.886) had similar efficacy rankings, both outperforming ketamine (SUCRA = 0.557), dexamethasone (SUCRA = 0.334), and minocycline (SUCRA = 0.080). The analgesic drug network: Ropivacaine (SUCRA = 0.775), lidocaine (SUCRA = 0.748), parecoxib (SUCRA = 0.691), and butorphanol (SUCRA = 0.679) showed a trend of reducing the incidence of dNCR, but the 95% CrI of each drug compared to saline all exceeded 1.0, and the differences were not statistically significant. The CINeMA evidence quality assessment showed that the majority of the comparative evidence in these three networks had a “low” or “very low” quality.

**Conclusion:**

In this meta-analysis, propofol ranked the highest among the anesthetic maintenance drugs, dexmedetomidine and ulinastatin ranked among the top for adjuvant drugs, and analgesic drugs did not show a definite protective effect. Due to the low quality of evidence, publication bias, and the lack of head-to-head comparisons, the above rankings are only preliminary references and are not sufficient to constitute clinical recommendations.

**Systematic Review Registration:**

https://www.crd.york.ac.uk/prospero/display_record.php?ID=CRD42024607899, identifier CRD42024607899.

## 1 Introduction

Since the late 20th century, delayed neurocognitive recovery (dNCR) has been recognized as a common perioperative neurological complication. It is characterized by a high incidence and significantly prolonged cognitive recovery. The incidence of dNCR ranges from 20% to 40% among high-risk elderly patients (aged >65 years). In highly vulnerable populations, such as those undergoing cardiac surgery or extracorporeal circulation, The incidence can increase to 50%–80%. Patients with concomitant cardiovascular and cerebrovascular diseases are also at higher risk (Zhuang et al., 2023). This condition poses a major challenge to patient prognosis. In 2018, Evered et al. introduced a nomenclature system for perioperative neurocognitive disorders (PND) (Evered et al., 2018). Within this framework, dNCR is defined as early cognitive impairment occurring within 30 days following surgery (Meng et al., 2024; Deiner and Silverstein, 2009). dNCR is now recognized as an intermediate stage in the transition from acute postoperative delirium to persistent long-term cognitive decline (Suraarunsumrit et al., 2024). It affects multiple core cognitive domains, including memory and attention (Evered et al., 2022). This condition has been shown to significantly delay the restoration of postoperative physical function, reduce the ability to perform activities of daily living independently, and exert a substantial negative impact on long-term postoperative quality of life (Kahl et al., 2021; Boone et al., 2020).

Current evidence indicates that the pathogenesis of dNCR involves several interrelated mechanisms. These include neuroinflammation (Terrando et al., 2010), oxidative stress injury (Zhang et al., 2025), impaired cerebral autoregulation, alterations in synaptic plasticity (Hovens et al., 2016), and disruption of the blood--brain barrier (Qiu et al., 2023). These mechanisms may potentiate one another and generate a cascade of amplification effects (Anand et al., 2024; Lin et al., 2020). Existing pharmacological interventions primarily target these core pathological pathways. In clinical practice, underlying risk factors such as advanced age and preexisting cognitive impairment increase susceptibility to dNCR. Among the modifiable factors in perioperative management, the choice of pharmacological agents stands out as a key entry point for the prevention of dNCR.

To date, various pharmacological agents have been investigated for the prevention of dNCR. Dexmedetomidine is an α_2_-adrenergic receptor agonist that confers neuroprotection. Previous meta-analyses have shown that it reduces postoperative cognitive dysfunction in elderly patients (Yu et al., 2022). Dexamethasone is a glucocorticoid that inhibits the NF-κB pathway and reduces proinflammatory cytokine release. However, its clinical benefit varies by surgery type (Glumac et al., 2017). Ulinastatin is a broad-spectrum protease inhibitor that alleviates neural injury by suppressing the inflammatory cascade. Multiple randomized controlled trials have confirmed its ability to improve postoperative cognitive outcomes (Duan et al., 2021). Ketamine antagonizes NMDA receptors and modulates glutamate-induced excitotoxicity, though its clinical efficacy remains debated (Hovaguimian et al., 2018). Parecoxib is a selective COX-2 inhibitor that reduces prostaglandin-mediated neuroinflammation, and has shown protective effects on early postoperative cognition (Huang et al., 2020). Together, these diverse options offer a range of potential preventive strategies. However, most existing evidence comes from pairwise meta-analyses comparing a single drug with placebo. There is a lack of head-to-head comparisons among drugs within the same clinical category. Consequently, clinicians find it difficult to obtain evidence-based guidance when choosing among similar agents.

Therefore, this study starts from clinical application scenarios rather than pharmacological mechanisms. We divided the included interventions into three categories: anesthetic maintenance drugs, adjunctive sedatives/neuroprotective agents, and analgesics. By integrating direct and indirect evidence within each category, we conducted a comparative evaluation of drugs with similar clinical uses. Our aim is to provide more practical evidence-based references for perioperative pharmacological prevention of dNCR.

## 2 Methods

### 2.1 Protocol registration

The protocol of this study was registered on the international prospective register of systematic reviews (PROSPERO) under the registration number CRD42024607899. As this study did not involve any individual patient data, institutional review board approval was not required. The review was conducted in accordance with the Cochrane Handbook for Systematic Reviews of Interventions (Cumpston et al., 2022; Page et al., 2021), and the reporting followed the PRISMA 2020 statement (Preferred Reporting Items for Systematic Reviews and Meta-Analyses).

### 2.2 Search strategy

Literature searches were conducted in PubMed, Embase, Cochrane Library, and Web of Science from database inception to July 2025. The search strategy was structured around three key domains:target population (high-risk patients), interventions (unrestricted by drug class, covering the core pharmacological components of multimodal analgesia and anesthesia strategies, including intravenous and inhaled anesthetics, analgesic adjuvants, opioid analgesics, NMDA receptor antagonists, α_2_-adrenergic receptor agonists, and glucocorticoids), and study design (randomized controlled trials only). Both MeSH terms and free-text words were used. The complete search strategy is provided in Supplementary Appendix S1. To minimize publication bias and identify unpublished studies, we also searched the WHO International Clinical Trials Registry Platform (ICTRP) and the US National Institutes of Health clinical trial registry (ClinicalTrials.gov). In addition, the reference lists of relevant systematic reviews were manually screened to identify potentially missed studies. Only studies published in English were included.

### 2.3 Inclusion and exclusion criteria and study selection

Studies were considered eligible if they met all of the following criteria: (1) a randomized controlled trial (RCT) design; (2) adult patients undergoing any type of surgery; (3) pharmacological intervention, irrespective of drug class; (4) a control group receiving either placebo (e.g., normal saline) or an active comparator; (5) the study clearly defined and reported the incidence of delayed neurocognitive recovery (dNCR) within 30 days after surgery. The diagnosis can be based on any of the following standardized criteria: postoperative cognitive dysfunction (POCD), perioperative neurocognitive disorder (PND), or delayed neurocognitive recovery (dNCR), and must use validated neuropsychological assessment tools (such as MMSE, MoCA, or a comprehensive test combination including at least two cognitive domain subtests); and (6) the full text was published in English.

Exclusion criteria were as follows: (1) patients with pre-existing neurological disorders (e.g., dementia, delirium, cerebral infarction), pregnancy, or age <18 years; (2) non-pharmacological interventions; (3) combination regimens in the experimental group (e.g., dexmedetomidine plus propofol); or any non-pharmacological intervention; (4) case reports, commentaries, or animal studies; (5) unavailable full text, incomplete data, or duplicate publications; (6) non-randomized studies; and (7) publications not in English.

The 18 interventions (including normal saline) included in this analysis were derived from RCTs that met the eligibility criteria and generally reflect the current body of evidence in dNCR research. According to clinical function, the 17 active interventions (excluding saline) were classified into three independent intervention networks:Network 1 (maintenance anesthetics) included intravenous anesthetics (propofol [PRO], remimazolam [RMZ], midazolam [MDZ]) and volatile anesthetics (desflurane [DES], sevoflurane [SEVO], isoflurane [ISO]). Network 2 (adjunct sedatives and neuroprotective agents) comprised α_2_-adrenergic receptor agonists (dexmedetomidine [DEXM]), glucocorticoids (dexamethasone [DEXA]), NMDA receptor antagonists (ketamine [KET]), and other neuroactive agents (ulinastatin [UTI], minocycline [MINO]). Network 3 (analgesics) included local anesthetics (lidocaine [LIDO], ropivacaine [ROPI]), nonsteroidal anti-inflammatory drugs (parecoxib [PARE]), and opioid analgesics (sufentanil [SUF], fentanyl [FENT], butorphanol [BUTO]).

Each network is analyzed independently, and respective comparison frameworks are constructed based on direct and indirect evidence. The stratification is based on the clinical functions during the perioperative period of the drugs, in order to ensure the clinical comparability of the intervention measures within the network, with the aim of evaluating the preventive effects of different drugs on dNCR.

### 2.4 Literature screening and data extraction

Two reviewers (Y.Q.L. and Y.Q.Y.) independently carried out the literature screening and data extraction. All retrieved records were managed using EndNote 21. The reviewers first screened titles and abstracts according to predefined PICOS criteria. After duplicate removal, full-text articles were obtained and re-assessed against the eligibility criteria. Disagreements between the two reviewers were resolved by discussion or by consulting a third reviewer (W.X.C.).

The following data were extracted: (1) study characteristics—first author, year of publication, country where the study was conducted, and trial registration number; (2) participant information—sample size, age, sex, eligibility criteria, and type of surgery; and (3) intervention and outcome details—drug regimen, timing of outcome assessment, assessment tools, and diagnostic criteria for delayed neurocognitive recovery (dNCR).

The primary outcome was the incidence of dNCR occurring within 30 days after surgery or during hospitalisation, expressed as an odds ratio (OR) with 95% credible interval (CrI). For continuous outcomes, we extracted or calculated the mean and standard deviation from baseline to the end of follow-up, applying data conversion methods as recommended by the Cochrane Handbook when needed.

### 2.5 Risk of bias assessment

Two researchers (Y.Q.L. and Y.Q.Y.) independently assessed the risk of bias of the included studies using the Cochrane RoB 2.0 tool. The assessment covered seven core domains:random sequence generation, allocation concealment, blinding of participants and personnel, blinding of outcome assessment, incomplete outcome data, selective reporting, and other potential sources of bias. Each study was rated as “low risk”, “high risk”, or “some concerns”. Disagreements were resolved through discussion or by a third researcher (W.X.C.).

### 2.6 Data analysis

In this study, Bayesian network meta-analysis (NMA) was employed for the comparative evaluation and ranking of the efficacy of three independent intervention networks - Network 1 (anesthesia maintenance drugs), Network 2 (adjuvant sedation/neuroprotective agents), and Network 3 (analgesics) - to ensure methodological consistency. For the analgesic drug network, we further supplemented with trial sequential analysis (TSA) to assess the sufficiency and robustness of the evidence.

#### 2.6.1 Network meta-analysis: direct evidence

Using R version 4.5.1, we calculated ORs with 95% CrIs for the binary outcome (dNCR incidence). Given anticipated clinical and methodological heterogeneity, a random-effects model (DerSimonian-Laird method) was pre-specified. Heterogeneity was quantified using the I^2^ statistic (I^2^ ≤ 25%:low; 25%–50%:moderate; 50%–75%:substantial; >75%:considerable). This analysis provided the basis for direct evidence within the subsequent NMA and was performed only for direct comparisons that included at least two studies.

#### 2.6.2 Network meta-analysis: Bayesian model building, parameter estimation, and ranking

This study employed Bayesian network meta-analysis to integrate direct and indirect comparative evidence from three evidence networks, and evaluated the relative efficacy of each intervention measure. The analysis was conducted using the gemtc package in the R 4.5.1 environment.

The network structure diagrams of each evidence network were drawn based on the comparison relationships among the intervention measures. For multi-arm trials, data transformation and covariance matrix construction were carried out according to the recommended methods in the Cochrane Handbook to correct the internal correlations of the studies. A random effects model was pre-specified to address the potential impact of clinical and methodological heterogeneity. In the analysis, a weak information prior was used: the treatment effect parameter *d* followed a normal distribution N (0, 100^2^), and the heterogeneity parameter τ followed a uniform distribution Uniform (0, 2). Parameter estimation was based on the Markov Chain Monte Carlo (MCMC) method. Each chain underwent 20,000 adaptive iterations to ensure convergence, and then four chains were sampled in parallel for 15,000 times, with a refinement step size of 1/10 for each 10 samples. Convergence was determined jointly by the Brooks-Gelman-Rubin statistic (R̂ < 1.05) and the effective sample size. Model fit was evaluated using the Deviance Information Criterion (DIC).

Under the assumption of consistency, we fitted a consistency model and used the node-splitting method to test the local inconsistencies in each closed loop. If a loop indicated local inconsistency (P ≤ 0.05), a non-consistency model was further fitted and the corresponding results were reported. The effect size was expressed as the odds ratio (OR) and its 95% credible interval (CrI). The efficacy ranking of each intervention measure was quantified using the cumulative sum of the area under the surface under the cumulative ranking curve (SUCRA), and the closer the SUCRA value was to 1, the greater the possibility that the intervention became the optimal choice. Publication bias was detected using a comparison-corrected funnel plot and quantitatively verified by Egger’s linear regression (P < 0.10 was the threshold for potential bias).

#### 2.6.3 Sensitivity analysis and subgroup analysis

To comprehensively assess the robustness of the main results, three networks were analyzed using the leave-one-out method, compared between random effect and fixed effect models, and analyzed within the frequentist framework. Additionally, considering the significant heterogeneity in the included studies regarding surgical types, cognitive assessment tools, and intervention schemes, we conducted supplementary paired meta-analyses using the risk ratio (RR) as the effect indicator, and performed subgroup analyses by surgical risk category, cognitive assessment scale type, and intervention type. At the same time, the aforementioned covariates were included in the univariate and multivariate regression models to quantitatively evaluate their potential impact on the effect estimation. The surgical risk classification was derived indirectly from the surgical name and is an exploratory analysis; its results should be interpreted with caution.

To further evaluate the robustness of the evidence base for the analgesic agents in the network meta-analysis (NMA), we performed trial sequential analysis (TSA). Using the pooled event rate from the normal saline arm within the analgesic network as the control event proportion (pC = 19.4%), with a type I error (α) of 0.05 and a statistical power of 80% (β = 0.2), we calculated whether the cumulative Z-curves for each analgesic agent (with odds ratio [OR] < 1 and higher SUCRA rankings) crossed the conventional significance boundaries or the TSA monitoring boundaries, to determine whether the current evidence met the required information size (RIS).

Additionally, to explore the time-dependent protective effects of the analgesics, we performed subgroup analyses stratified by postoperative assessment time points (≤1 day, 1–3 days, 3–7 days) for agents with sufficient data (lidocaine and parecoxib).

### 2.7 Certainty of evidence assessed using CINeMA

The certainty of evidence was evaluated following the GRADE approach for pairwise comparisons. Specifically, we applied the CINeMA (Confidence in Network Meta-Analysis) framework to assess confidence in the NMA results. Six domains were evaluated:within-study bias, reporting bias (including publication and other reporting biases), indirectness, imprecision, heterogeneity, and incoherence. Each domain was judged as “no concerns”, “some concerns”, or “major concerns”. Based on these judgments, the overall confidence in the evidence was rated as high, moderate, low, or very low. All included RCTs started as high-certainty evidence and could be downgraded according to the seriousness of problems identified in each domain.

## 3 Results

### 3.1 Characteristics of the included studies

We systematically searched four electronic databases: PubMed (n = 3,392), Embase (n = 13,663), the Cochrane Library (n = 1,653), and Web of Science (n = 3,531). In addition, 15 randomized controlled trials (RCTs) were identified from other sources, bringing the total number of records to 22,254. After removing duplicates (n = 4,449), 17,805 records remained and were screened by title and abstract, of which 17,167 were excluded. The remaining 638 articles were retrieved for full-text assessment. Two independent reviewers evaluated the full texts against the prespecified inclusion and exclusion criteria; 569 articles failed to meet the criteria and were excluded. Consequently, 69 RCTs were included in the final analysis. The detailed screening process is illustrated in Figure 1 (PRISMA flowchart).

**FIGURE 1:**
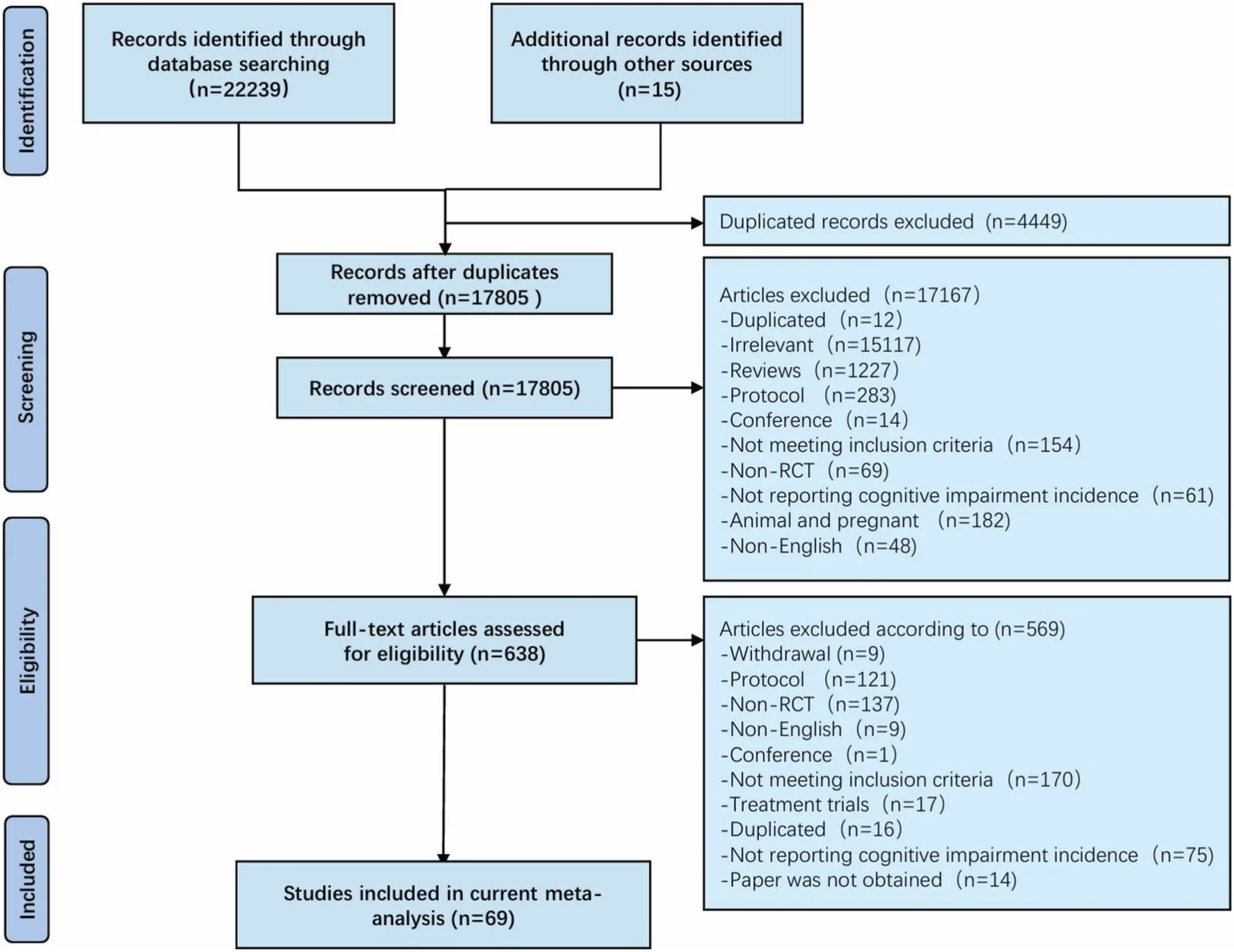
PRISMA Flow Diagram. Study selection process.

This analysis included 69 randomized controlled trials (RCTs) involving a total of 9,636 patients, in which 18 interventions (including a placebo or normal saline control) were evaluated. These interventions were categorized into three independent networks. Network 1 (maintenance anesthetics) included intravenous agents (propofol, remimazolam, midazolam) and inhaled agents (desflurane, sevoflurane, isoflurane). Network 2 (adjunct sedatives and neuroprotective agents) comprised α_2_-adrenergic receptor agonists (dexmedetomidine), glucocorticoids (dexamethasone), NMDA receptor antagonists (ketamine), protease inhibitors (ulinastatin), and a tetracycline antibiotic (minocycline). Network 3 (analgesics) included opioid analgesics (sufentanil, fentanyl, butorphanol), local anesthetics (lidocaine, ropivacaine), and a nonsteroidal anti-inflammatory drug (parecoxib).

The mean age of the study population was 66.8 ± 9.2 years, derived from 9,446 patients (two studies did not report age). Female patients accounted for 46.17% (4,356/9,436), with three studies not reporting sex distribution. Geographically, the majority of the trials were conducted in Asia (54 studies, 78.26%), followed by Europe (9, 13.04%), North America (4, 5.80%), Africa (1, 1.45%), and South America (1, 1.45%). The overall incidence of delayed neurocognitive recovery (dNCR) was 21.30% (2,011/9,443), after excluding patients who failed to complete the final cognitive assessments due to loss to follow-up, withdrawal, or missing data.

Postoperative follow-up durations varied considerably across studies, ranging from 1 h to 30 days. There was also substantial heterogeneity in the methods used for cognitive assessment. Twenty-seven studies (39.1%) used the Mini-Mental State Examination (MMSE) as the primary diagnostic tool, four studies (5.8%) employed the Montreal Cognitive Assessment (MoCA), and the remaining 38 studies (55.1%) adopted comprehensive neuropsychological test batteries. Most of these batteries combined the MMSE or MoCA with additional tests, such as the Trail Making Test (TMT), Digit Symbol Substitution Test (DSST), Digit Span Test, and Stroop Color-Word Test, to evaluate multiple cognitive domains, including executive function, attention, processing speed, and memory. The types of surgery involved included cardiac surgery (e.g., coronary artery bypass grafting, valve replacement), major orthopedic surgery (e.g., total hip or knee arthroplasty), intra-abdominal general surgery procedures (e.g., radical resection of gastrointestinal tumors, laparoscopic cholecystectomy), thoracic surgery (e.g., lobectomy, radical resection of esophageal cancer), and neurosurgery.

Given the considerable clinical and methodological heterogeneity across the included studies in terms of patient baseline characteristics, surgical types, anesthesia protocols, and cognitive assessment methods, interpretation of the present findings and their extrapolation to specific clinical settings should be undertaken with caution. The baseline characteristics of the three network meta-analyses are presented in Tables 1–3 respectively.

**TABLE 1:** Baseline characteristics of included studies (anesthetic maintenance agents, network 1).

Study, year	Type of surgery	Drug	N	Age, years [mean ± SD]	Female [%]	Occurrence of cognitive impairment
(Chen et al., 2001)	Orthopedic surgery	Desflurane Sevoflurane	35 35	75.0 ± 8.0 73.0 ± 9.0	42.9 48.6	0/35 1/35
(Deepak et al., 2013)	Non-cardiac surgery	Desflurane Sevoflurane	30 30	69.2 ± 4.7 69.5 ± 4.4	60.0 36.7	0/30 1/30
(Egawa et al., 2016)	Thoracic Surgery	Propofol Sevoflurane	72 72	69.0 ± 2.5 72.0 ± 2.3	31.9 45.8	16/72 24/72
(Geng et al., 2017)	Abdominal surgery	Propofol Sevoflurane Isoflurane	50 50 50	N/A N/A N/A	60.0 56.0 64.0	2/50 10/50 15/50
(Guo et al., 2020)	Non-cardiac surgery	Sevoflurane Propofol	117 117	69.0 ± 5.9 69.0 ± 4.8	65.0 60.7	32/110 30/110
(Kletecka et al., 2019)	Neurosurgery	Propofol Sevoflurane	20 23	44.8 ± 10.8 43.6 ± 13.2	45 48	4/20 4/23
(Kuzminskaite et al., 2023)	Thyroid surgery	Desflurane Sevoflurane	48 34	52.5 ± 10.3 52.0 ± 13.5	81.3 85.3	1/48 0/34
(Meng et al., 2024; Li et al., 2019)	Orthopedic surgery	Propofol Midazolam	55 54	68.2 ± 6.4 66.9 ± 6.6	56.4 59.3	10/55 28/54
(Li et al., 2021a)	Abdominal surgery	Propofol Sevoflurane	272 272	64.0 ± 4.4 65.0 ± 5.2	25.2 34.4	38/226 46/221
(Evered et al., 2018; Mansouri et al., 2019)	Ophthalmic Surgery	Midazolam NS	50 50	63.6 ± 8.3 64.0 ± 7.3	50.0 62.0	4/50 10/50
(Meineke et al., 2014)	Non-cardiac surgery	Desflurane Sevoflurane	37 47	72.3 ± 2.4 71.9 ± 1.8	64.9 70.2	17/37 32/47
(Evered et al., 2018; Liao et al., 2023)	Abdominal surgery	Remimazolam NS	34 35	70.1 ± 3.6 69.7 ± 2.5	38.2 40.0	3/34 10/35
(Qiao et al., 2023)	ENT Surgery	Desflurane Propofol	31 32	66.7 ± 7.2 63.9 ± 5.3	0.0 6.3	3/31 1/32
(Rörtgen et al., 2010)	Non-cardiac surgery	Desflurane Sevoflurane	40 40	69.0 ± 2.9 69.2 ± 3.0	50.0 45.0	11/27 14/30
(Rohan et al., 2005)	Urological/Gynecological Surgery	Propofol Sevoflurane	15 15	72.9 ± 13.3 73.8 ± 14.1	20.0 27.0	7/15 7/15
(Tanaka et al., 2017)	Orthopedic surgery	Desflurane Propofol	45 45	69.8 ± 1.2 70.6 ± 1.4	44.4 66.7	26/40 19/39
(Varsha et al., 2024)	Cardiac surgery	Propofol Sevoflurane	36 36	59.3 ± 6.9 59.5 ± 10.1	11.1 19.4	11/36 20/36
(Wu et al., 2024)	Orthopedic surgery	Remimazolam Midazolam	35 35	72.2 ± 4.4 72.0 ± 4.9	51.4 57.1	0/35 2/35
(Zhang et al., 2018a)	Orthopedic surgery	Propofol Sevoflurane	195 192	72.8 ± 5.5 72.4 ± 5.6	30.8 33.3	28/189 44/190

Baseline characteristics of RCTs, in the anesthetic maintenance network: first author and year of publication, surgery type, intervention, sample size, patient age (mean ± SD), percentage of female participants (%), and incidence of delayed neurocognitive recovery (dNCR). Data from 19 RCTs, involving 2,478 patients. Abbreviations: dNCR, delayed neurocognitive recovery; N/A, not available.

**TABLE 2:** Baseline characteristics of included studies (adjunct sedatives and neuroprotective agents, network 2).

Study, year	Type of surgery	Drug	N	Age, years [mean ± SD]	Female [%]	Occurrence of cognitive impairment
(Chen et al., 2013)	Abdominal surgery	Dexmedetomidine NS	63 63	66.2 ± 7.5 67.9 ± 6.6	44.1 50.8	9/63 17/63
(Chen et al., 2015)	Non-cardiac surgery	Dexmedetomidine NS	87 61	70.6 ± 4.2 71.4 ± 4.9	35.6 37.7	8/87 13/61
(Chen and Li, 2020)	Abdominal surgery	Dexmedetomidine NS	43 45	64.9 ± 11.4 65.4 ± 11.7	46.5 48.8	7/43 29/45
(Li et al., 2021b)	Neurosurgery	Dexmedetomidine NS	90 30	71.9 ± 4.1 73.4 ± 5.1	43.3 43.3	17/90 12/30
(Fang et al., 2014)	Neurosurgery	Dexamethasone NS	640 320	48.5 ± 5.5 48.0 ± 5.8	61.1 62.1	165/635 71/319
(Gao et al., 2020)	Cardiac surgery	Dexmedetomidine NS	30 30	69.5 ± 5.1 70.4 ± 4.2	53.3 50.0	0/30 5/30
(Gao et al., 2021)	Cardiac surgery	Dexmedetomidine NS	20 20	70.5 ± 4.1 71.4 ± 4.5	45.0 50.0	2/20 8/20
(Ghazaly et al., 2023)	Emergency surgery	Dexmedetomidine Ketamine NS	20 20 20	70.0 ± 3.8 71.2 ± 3.9 69.4 ± 4.7	25.0 50.0 40.0	0/20 2/20 7/20
(Glumac et al., 2017)	Cardiac surgery	Dexamethasone NS	85 84	63.7 ± 9.0 64.2 ± 9.4	21.2 21.0	9/80 21/81
(Gong et al., 2018)	Cardiac surgery	Dexmedetomidine NS	40 40	42.3 ± 1.6 42.4 ± 1.5	30.0 33.0	1/40 10/40
(Liu et al., 2020a)	Abdominal surgery	Dexmedetomidine NS	24 24	69.6 ± 4.4 68.6 ± 3.9	37.5 45.8	5/24 7/24
(Hollinger et al., 2021)	Mixed Major Surgeries	Haloperidol Ketamine NS	45 47 44	73.4 ± 6.3 73.4 ± 6.1 74.8 ± 6.6	35.6 44.7 54.5	7/45 10/47 6/44
(Kim et al., 2022)	Orthopedic surgery	Dexmedetomidine NS	40 40	65.0 ± 5.9 66.3 ± 6.3	42.5 55.3	9/40 9/38
(Lee et al., 2015)	Orthopedic surgery	Ketamine NS	25 26	68.3 ± 5.3 68.4 ± 6.5	44.0 72.4	1/25 8/26
(Li et al., 2015)	Abdominal surgery	Dexmedetomidine NS	60 60	69.0 ± 5.0 70.0 ± 6.0	44.0 48.0	10/50 21/50
(Liu et al., 2020b)	ENT Surgery	Dexmedetomidine NS	30 30	43.4 ± 8.9 40.6 ± 9.4	16.7 13.3	1/30 3/30
(Valentin et al., 2016)	Mixed Major Surgeries	Dexamethasone NS	68 72	68.6 ± 6.0 67.9 ± 6.4	66.2 55.6	14/68 21/72
(Luo et al., 2024)	Thoracic surgery	Ketamine NS	87 42	55.6 ± 9.2 55.9 ± 10.6	51.7 47.6	16/87 9/42
(Ma et al., 2023)	Abdominal surgery	Ketamine NS	31 31	69.5 ± 4.4 70.6 ± 4.2	22.6 22.6	5/31 12/31
(Zhuang et al., 2023; Mansouri et al., 2019)	Ophthalmic Surgery	Dexmedetomidine NS	50 50	66.5 ± 1.6 64.0 ± 7.3	50.0 62.0	6/50 10/50
(Meng et al., 2024; Liao et al., 2023)	Abdominal surgery	Dexmedetomidine NS	35 35	71.3 ± 3.6 69.7 ± 2.5	42.9 40.0	3/35 10/35
(Mohamed and Shaaban, 2014)	Abdominal surgery	Dexmedetomidine NS	25 25	63.9 ± 5.0 67.8 ± 5.4	20.0 0.0	2/25 10/25
(Nagz, 2026)	Orthopedic surgery	Dexmedetomidine NS	60 60	68.3 ± 3.9 68.5 ± 5.0	60.0 48.3	10/60 21/60
(Ottens et al., 2014)	Cardiac surgery	Dexamethasone NS	140 138	63.4 ± 12.3 65.4 ± 11.5	26.6 21.0	19/140 10/138
(Sun et al., 2024)	Abdominal surgery	Dexmedetomidine NS	40 40	68.2 ± 2.2 68.0 ± 1.9	52.5 50.0	5/40 14/40
(Takazawa et al., 2023)	Orthopedic surgery	Minocycline NS	90 95	74.5 ± 6.7 75.0 ± 6.7	78.0 80.0	8/90 4/95
(Verdonk et al., 2024)	Orthopedic surgery	Ketamine NS	149 143	72.3 ± 7.1 72.3 ± 7.3	62.4 60.8	50/149 54/143
(Wang et al., 2017)	Thoracic surgery	Ulinastatin NS	38 37	66.0 ± 6.0 67.0 ± 5.0	7.9 10.8	9/38 17/37
(Zhang et al., 2018b)	Orthopedic surgery	Ulinastatin NS	30 30	71.3 ± 5.0 72.8 ± 5.3	53.3 60.0	5/30 13/30
(Wang et al., 2020)	Abdominal surgery	Dexmedetomidine NS	60 50	68.4 ± 3.3 68.3 ± 2.1	36.7 40.0	12/60 20/50
(Xu et al., 2013)	Abdominal surgery	Ulinastatin NS	40 40	75.6 ± 7.2 74.1 ± 8.1	47.5 45.0	1/40 11/40
(Xu et al., 2017)	Abdominal surgery	Dexmedetomidine NS	48 48	71.9 ± 31.4 72.1 ± 32.2	N/A N/A	3/48 10/48
(Ding et al., 2015)	Abdominal surgery	Dexmedetomidine NS	20 20	N/A N/A	15.0 15.0	1/20 4/20
(Zhang et al., 2019a)	Abdominal surgery	Dexmedetomidine NS	80 60	73.8 ± 14.5 74.1 ± 13.9	36.2 33.3	0/80 8/60
(Zhang et al., 2021)	Non-cardiac surgery	Dexmedetomidine NS	87 87	70.6 ± 4.2 71.4 ± 4.9	36.8 21.8	13/87 8/87
(Zhou et al., 2019)	Cardiac surgery	Ulinastatin Dexmedetomidine NS	39 39 38	70.6 ± 4.4 69.8 ± 5.1 70.0 ± 4.9	56.4 52.8 52.6	7/39 6/38 12/38

Baseline characteristics of RCTs, in the adjunct sedative and neuroprotective network: first author and year of publication, surgery type, intervention, sample size, patient age (mean ± SD), percentage of female participants (%), and incidence of delayed neurocognitive recovery (dNCR). Data from 36 RCTs, involving 4,836 patients. Abbreviations: dNCR, delayed neurocognitive recovery; N/A, not available.

**TABLE 3:** Baseline characteristics of included studies (analgesics, network 3).

Study, year	Type of surgery	Drug	N	Age, years [mean ± SD]	Female [%]	Occurrence of cognitive impairment
(Hashemi et al., 2013)	Urological or Orthopedic Surgery	Lidocaine NS	35 35	66.0 ± 1.0 67.0 ± 2.0	22.9 20.0	10/35 9/35
(Liu et al., 2020a)	Abdominal surgery	Ropivacaine NS	24 24	69.3 ± 4.4 68.6 ± 3.9	41.7 45.8	7/24 7/24
(Zhang et al., 2019b)	Abdominal surgery	Sufentanil Fentanyl	48 48	69 ± 2.1 70 ± 3.1	50.0 52.10	3/48 11/48
(Liu et al., 2025)	Colorectal surgery	Lidocaine NS	154 149	61.7 ± 9.8 63.4 ± 10.2	52.6 55.0	9/154 42/149
(Mu et al., 2017)	Orthopedic surgery	Parecoxib NS	310 310	69.6 ± 6.5 70.5 ± 6.9	73.9 73.2	28/310 53/310
(Tian et al., 2015)	Orthopaedic surgery	Parecoxib NS	35 35	72.5 ± 4.5 71.8 ± 5.1	40.0 45.7	10/35 19/35
(Wang et al., 2002)	Cardiac surgery	Lidocaine NS	43 45	57.8 ± 9.7 59.3 ± 9.4	2.3 2.2	8/43 18/45
(Wen et al., 2024)	Mixed Major Surgeries	Butorphanol Sufentanil	52 52	71.0 ± 3.7 71.5 ± 4.0	46.2 38.5	6/52 17/52
(Yang et al., 2015)	ENT Surgery	Butorphanol Fentanyl NS	65 65 65	72.0 ± 4.0 73.0 ± 4.0 72.0 ± 5.0	0.0 0.0 0.0	9/65 16/65 17/65
(Zhu et al., 2024)	Orthopedic surgery	Ropivacaine Sufentanil	30 30	73.9 ± 5.7 75.2 ± 6.6	56.7 46.7	2/30 9/30
(Zhu et al., 2016)	Orthopedic surgery	Parecoxib NS	60 65	75.1 ± 8.2 74.3 ± 7.6	N/A N/A	10/60 22/65
(Zhu et al., 2021)	Abdominal Surgery	Ropivacaine NS	30 29	71.2 ± 5.3 71.2 ± 5.2	26.7 34.5	2/30 8/29

Baseline characteristics of RCTs, in the analgesic network: first author and year of publication, surgery type, intervention, sample size, patient age (mean ± SD), percentage of female participants (%), and incidence of delayed neurocognitive recovery (dNCR). Data from 14 RCTs, involving 2,427 patients. Abbreviations: dNCR, delayed neurocognitive recovery; N/A, not available.

### 3.2 Risk of bias in the included studies

All 69 included randomized controlled trials reported using a randomized allocation method. Thirty-one studies (44.93%) were judged to have a low risk of bias. Thirty studies (43.47%) had an unclear risk of bias, primarily due to insufficient reporting of methodological details. The remaining eight studies (11.59%) were assessed as having a high risk of bias. Among these eight high-risk studies, four were rated as high risk because the nature of the intervention precluded blinding of personnel. Three studies received a high-risk rating owing to substantial loss to follow-up in the experimental group combined with imbalanced attrition between groups. One additional study was rated as high risk due to both lack of blinding and high loss to follow-up in the experimental group. A summary of the risk of bias for each included study is presented in the Figure 2.

**FIGURE 2:**
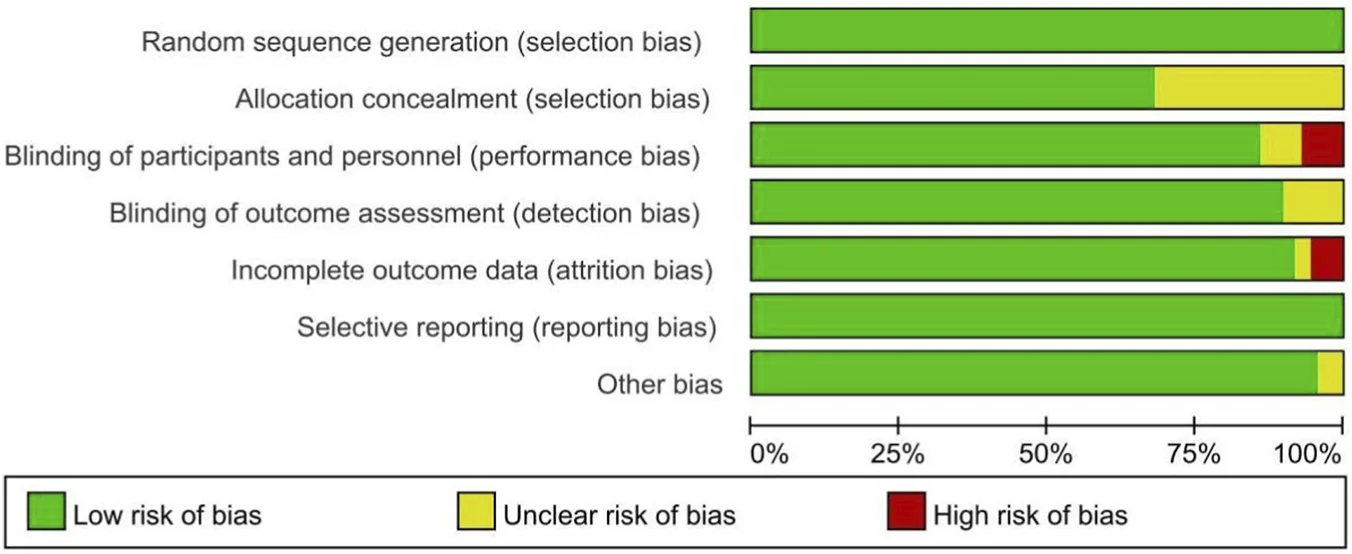
Risk of Bias Summary. Risk of bias distribution across domains of the Cochrane ROB 2.0 tool for the 69 included RCTs. Data are presented as percentages (n = 69).

### 3.3 Model quality assessment

Convergence was evaluated using R̂ and ESS. The R̂ values for all parameters in the three networks ranged from 1.00 to 1.01, and ESS was greater than 400, indicating good convergence. The goodness-of-fit ratios were 1.055, 1.081, and 1.032, respectively, close to 1.00, and the fit was satisfactory. The I^2^ values were 7%, 9%, and 8%, respectively, with very low heterogeneity. The node splitting test showed that the anesthesia maintenance drug and adjunct sedation/neuroprotection agent networks had good overall consistency (p > 0.05), but there was local inconsistency in the comparison between PRO and SEVO (p = 0.03). This result should be interpreted with caution. The analgesic drug network was a star-shaped structure and could not be subjected to consistency testing, and its conclusion needed to be combined with the TSA for comprehensive judgment.

### 3.4 Network meta-analysis results

#### 3.4.1 Bayesian network evidence plots

Figures 3–5 present the network evidence structure showing the direct comparisons between each intervention and the incidence of dNCR, specifically for anesthetic maintenance drugs, adjunctive sedation/neuroprotective agents, and analgesics. Each node represents an intervention, with node size proportional to the relative weight (sample size) of that drug in the network analysis. The thickness of the connecting lines between nodes reflects the volume of direct comparative evidence available for each pairwise comparison.

**FIGURE 3:**
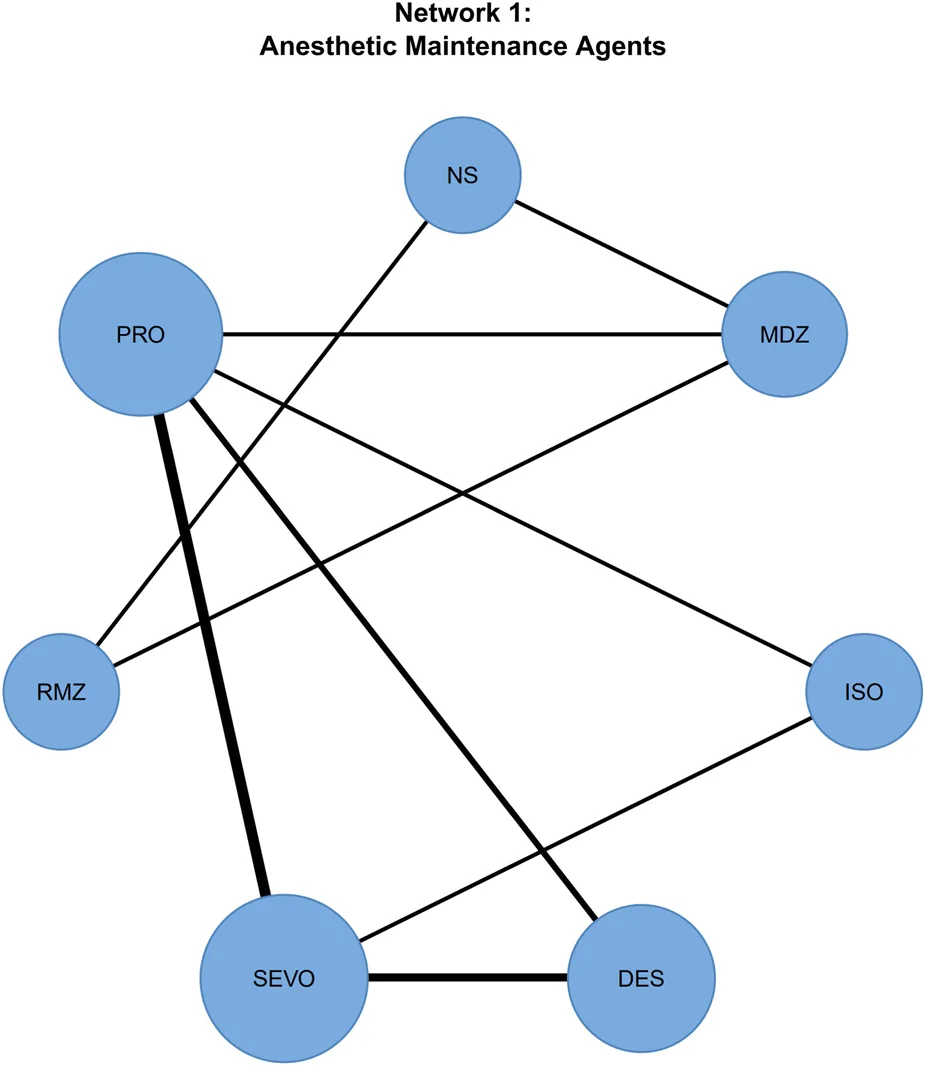
Network of Evidence for Anesthetic Maintenance Agents. Network of anesthetic maintenance agents: midazolam (MDZ), isoflurane (ISO), desflurane (DES), sevoflurane (SEVO), remimazolam (RMZ), propofol (PRO), and normal saline (NS).

**FIGURE 4:**
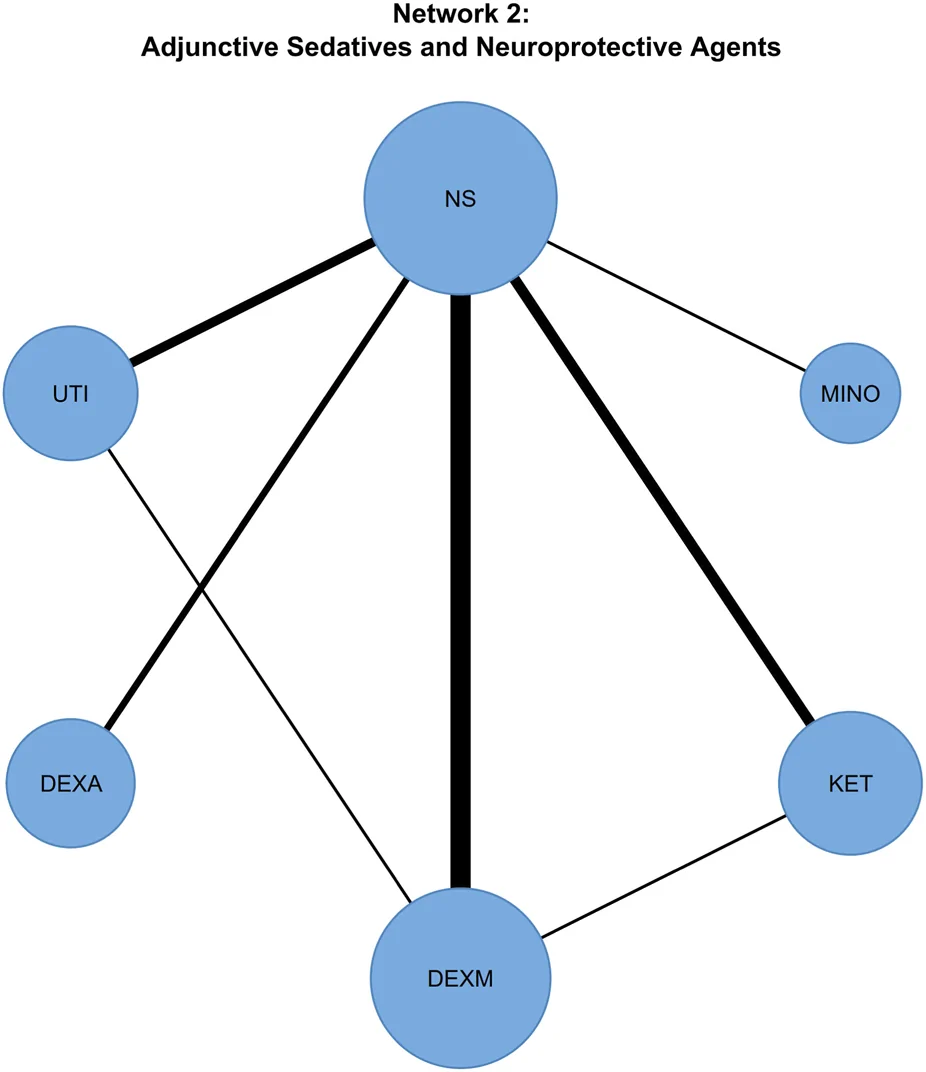
Network of Evidence for Adjunctive Sedatives and Neuroprotective Agents. Network of adjunct sedative and neuroprotective agents: dexmedetomidine (DEX), ketamine (KET), dexamethasone (DEXA), ulinastatin (UTI), minocycline (MINO), and normal saline (NS).

**FIGURE 5:**
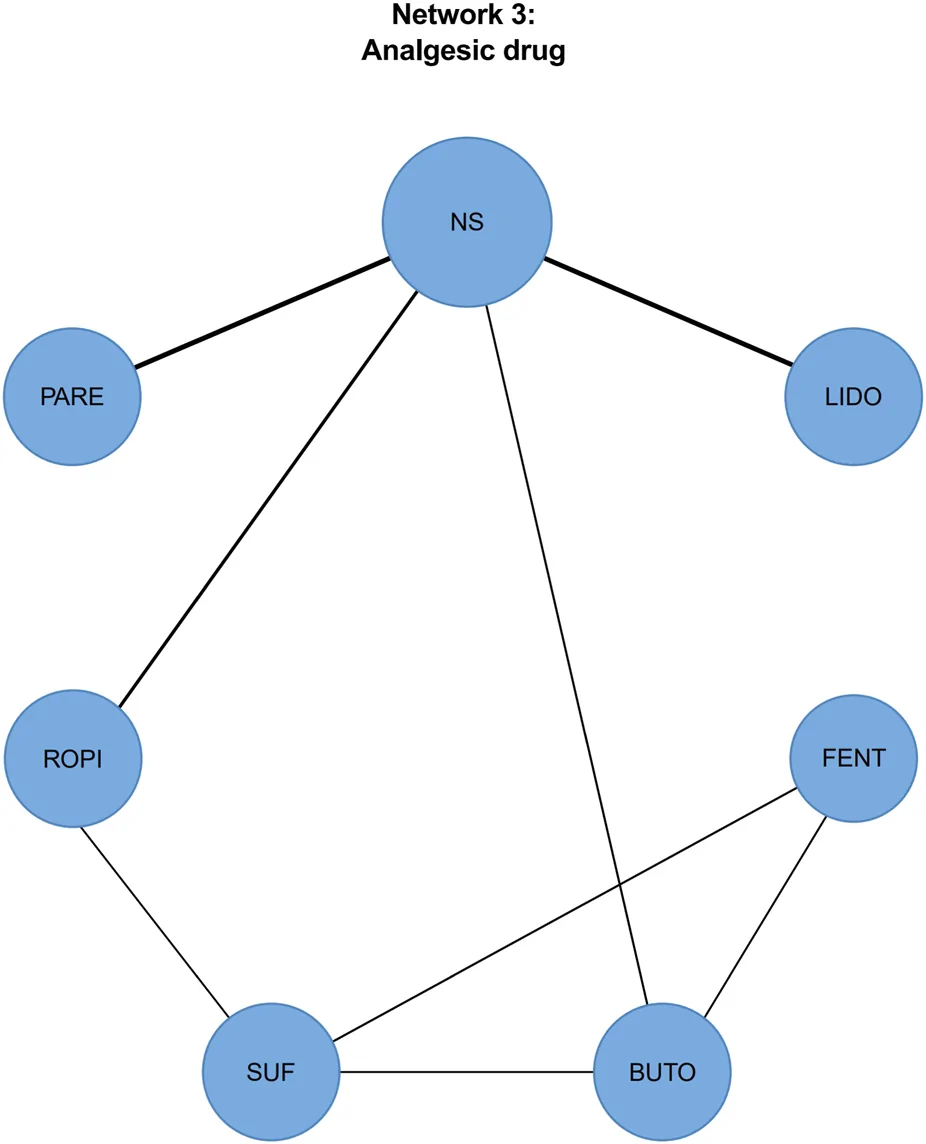
Network of Evidence for Analgesic Agents. Network of analgesic agents: ropivacaine (ROPI), butorphanol (BUTO), parecoxib (PARE), fentanyl (FENT), sufentanil (SUF), lidocaine (LIDO), and normal saline (NS). Node sizes are proportional to sample sizes; edge thickness reflects the number of direct comparisons.

#### 3.4.2 Network 1: anesthetic maintenance agents

This network comprised 19 studies with 2,478 patients and evaluated six anesthetic maintenance agents. Among these, one study employed a three-arm design (Geng et al., 2017). Using normal saline (NS) as the reference, propofol (OR 0.08, 95% CrI 0.02–0.37), remimazolam (OR 0.20, 95% CrI 0.05–0.73), sevoflurane (OR 0.14, 95% CrI 0.03–0.64), and desflurane (OR 0.10, 95% CrI 0.02–0.51) were all associated with a reduced risk of dNCR. Propofol showed a lower OR value compared to sevoflurane (OR 0.60, 95% CrI 0.44–0.82), midazolam (OR 0.21, 95% CrI 0.08–0.51), and isoflurane (OR 0.25, 95% CrI 0.10–0.59). Midazolam was markedly less effective than sevoflurane (OR 2.93, 95% CrI 1.13–7.60) and desflurane (OR 3.97, 95% CrI 1.37–11.51). Similarly, isoflurane exhibited a significantly inferior preventive effect relative to sevoflurane (OR 2.43, 95% CrI 1.07–5.52) and desflurane (OR 3.30, 95% CrI 1.25–8.72) (Figure 6).

**FIGURE 6:**
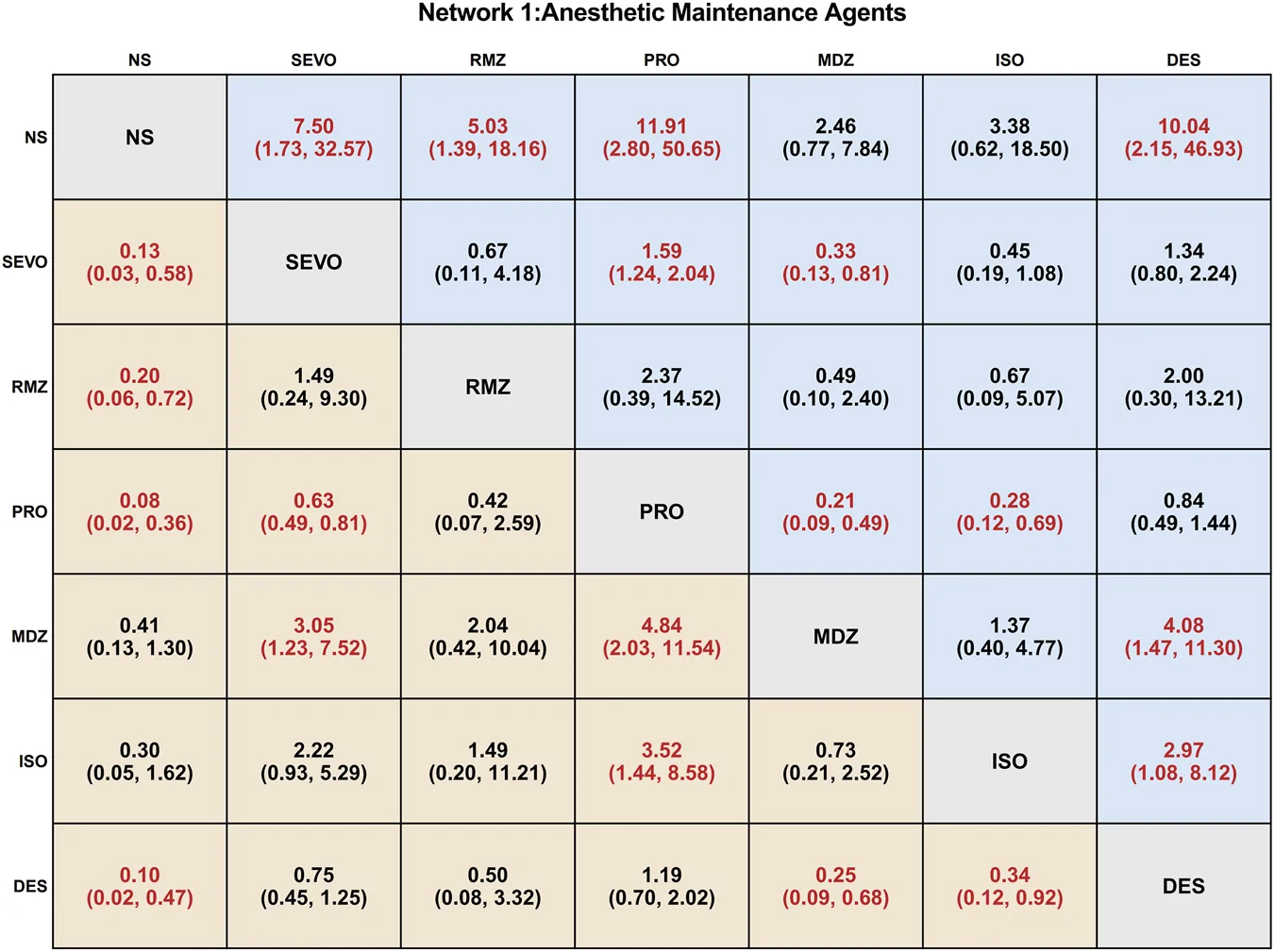
Network Meta-Analysis Forest Plot for Anesthetic Maintenance Agents. Data are presented as odds ratios (OR) with 95% credible intervals (CrI). Abbreviations: MDZ, midazolam; ISO, isoflurane; DES, desflurane; SEVO, sevoflurane; RMZ, remimazolam; PRO, propofol; NS, normal saline.

#### 3.4.3 Network 2: adjunctive sedatives and neuroprotective agents

A total of 36 studies encompassing 4,836 patients were included in this network, which assessed five adjunctive drug interventions. Compared with NS, ulinastatin (OR 0.32, 95% CrI 0.16–0.62) and dexmedetomidine (OR 0.34, 95% CrI 0.24–0.46) were both associated with a reduced risk of dNCR.In the direct comparison, dexmedetomidine was superior to ketamine (OR 0.52, 95% CrI 0.27–0.97), dexamethasone (OR 0.37, 95% CrI 0.20–0.68), and minocycline (OR 0.15, 95% CrI 0.03–0.70); ulinastatin was superior to minocycline (OR 0.14, 95% CrI 0.03–0.74) and dexamethasone (OR 0.35, 95% CrI 0.15–0.82) (Figure 7).

**FIGURE 7:**
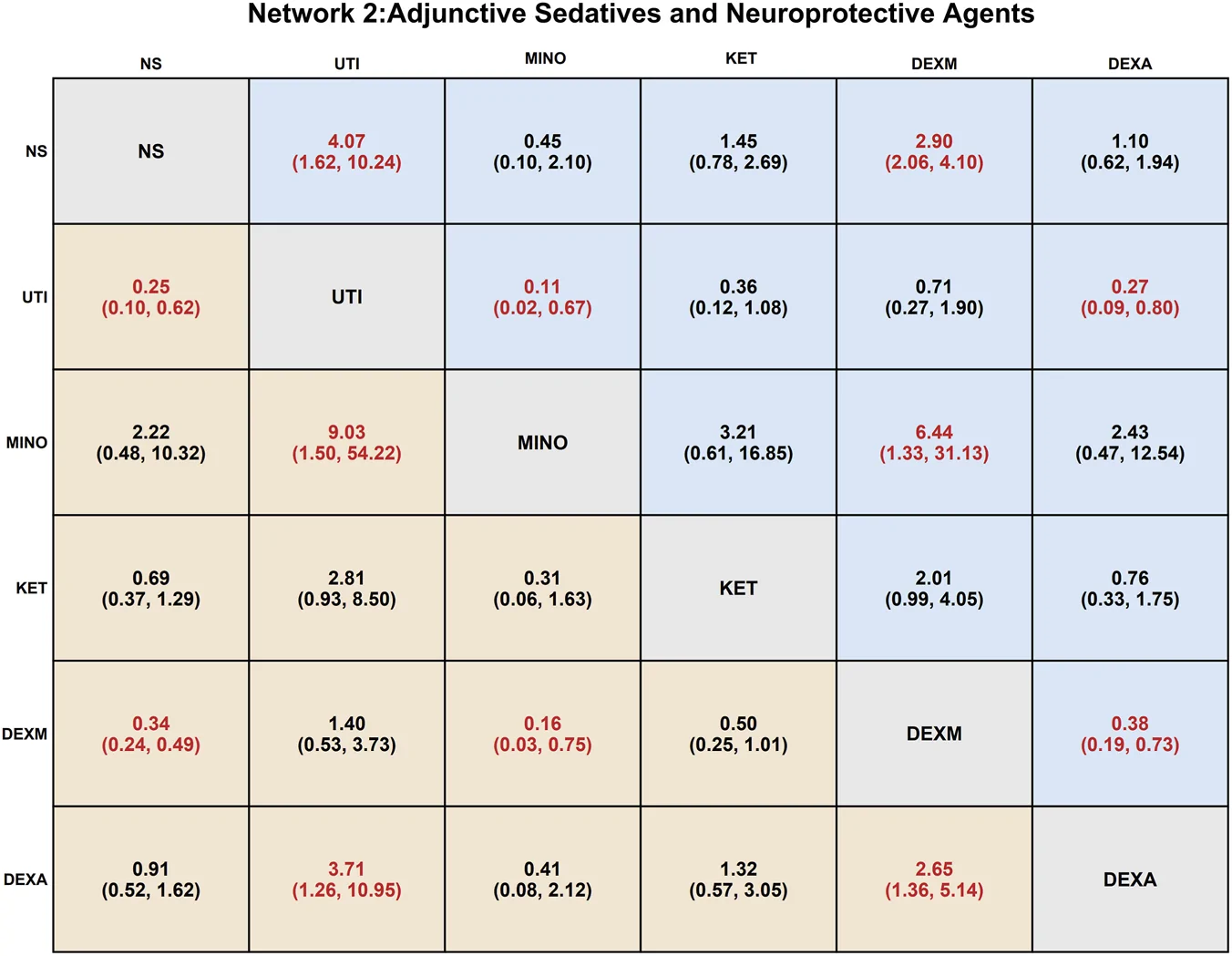
Network Meta-Analysis Forest Plot for Adjunctive Sedatives and Neuroprotective Agents. Data are presented as odds ratios (OR) with 95% credible intervals (CrI). Abbreviations: DEX, dexmedetomidine; KET, ketamine; DEXA, dexamethasone; UTI, ulinastatin; MINO, minocycline; NS, normal saline.

#### 3.4.4 Network 3: analgesic agents

The network meta-analysis included 12 studies (including 1 three-arm design, Yang et al., 2015), involving a total of 2,427 patients. Using normal saline as the reference, the point estimates for ropivacaine (OR 0.33, 95% CrI 0.08–1.30), lidocaine (OR 0.35, 95% CrI 0.13–1.09), parecoxib (OR 0.40, 95% CrI 0.14–1.18), and butorphanol (OR 0.44, 95% CrI 0.11–1.80) all pointed to a lower dNCR incidence. Sufentanil (OR 1.03, 95% CrI 0.22–4.94) was close to normal saline, while fentanyl (OR 1.39, 95% CrI 0.36–6.18) was the opposite. The 95% credible intervals for all comparisons crossed 1, suggesting no statistical difference between these drugs and normal saline. The indirect comparison among the active drugs also showed a large overlap in the credible intervals. In conclusion, this result should be regarded as a reference for exploratory analysis, and these results should be interpreted with caution (Figure 8).

**FIGURE 8:**
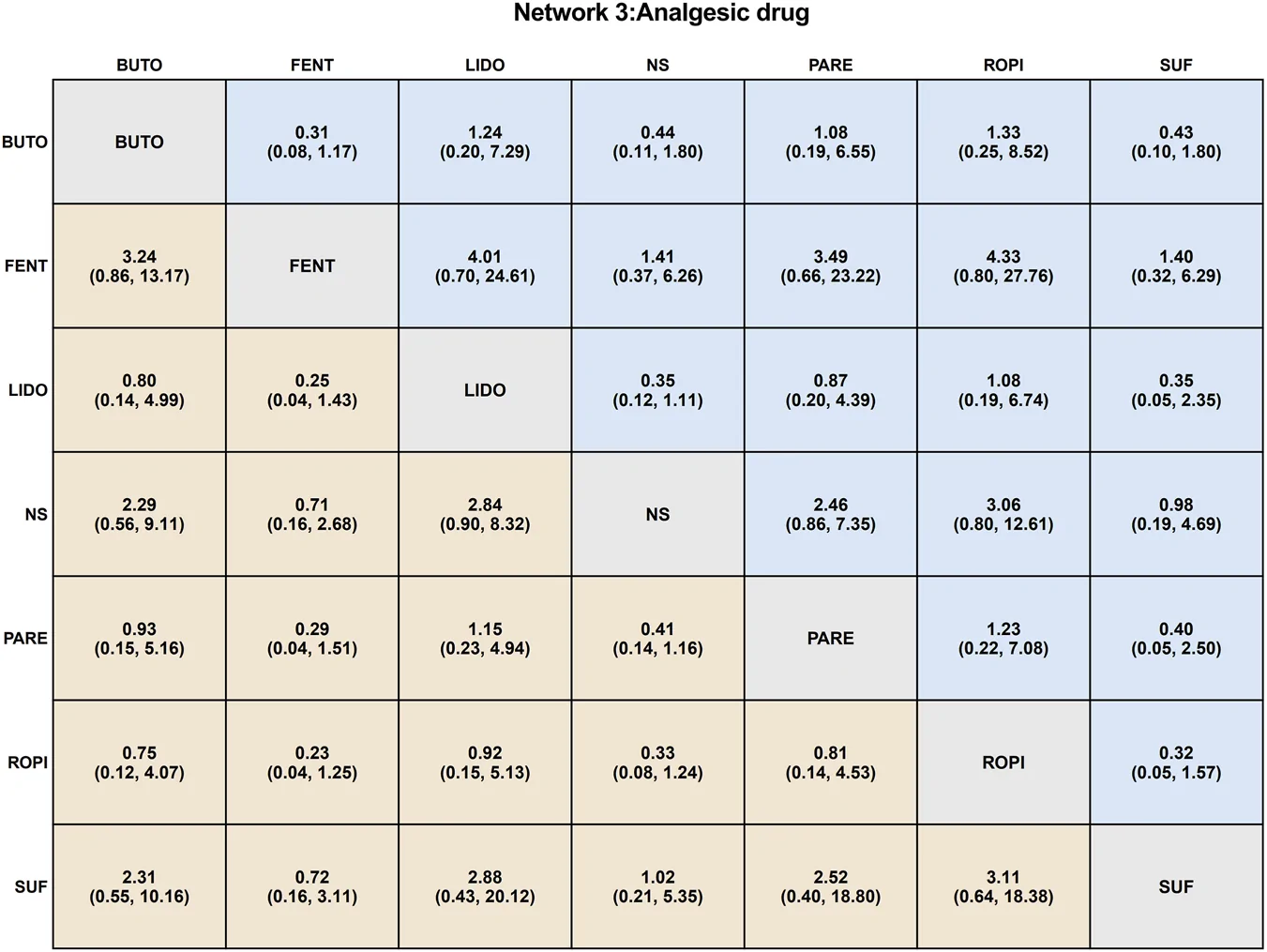
Network Meta-Analysis Forest Plot for Analgesic Agents. Data are presented as odds ratios (OR) with 95% credible intervals (CrI). Abbreviations: BUTO, butorphanol; FENT, fentanyl; LIDO, lidocaine; PARE, parecoxib; ROPI, ropivacaine; SUF, sufentanil; NS, normal saline.

#### 3.4.5 SUCRA value and probability graph

In the anesthesia maintenance drug network, propofol ranks the highest (SUCRA = 0.908), followed by desflurane (SUCRA = 0.773) > remimazolam (SUCRA = 0.657) > sevoflurane (SUCRA = 0.587), while isoflurane (SUCRA = 0.259) and midazolam (SUCRA = 0.258) are closely ranked, and all active drugs are superior to normal saline (Figure 9).

**FIGURE 9:**
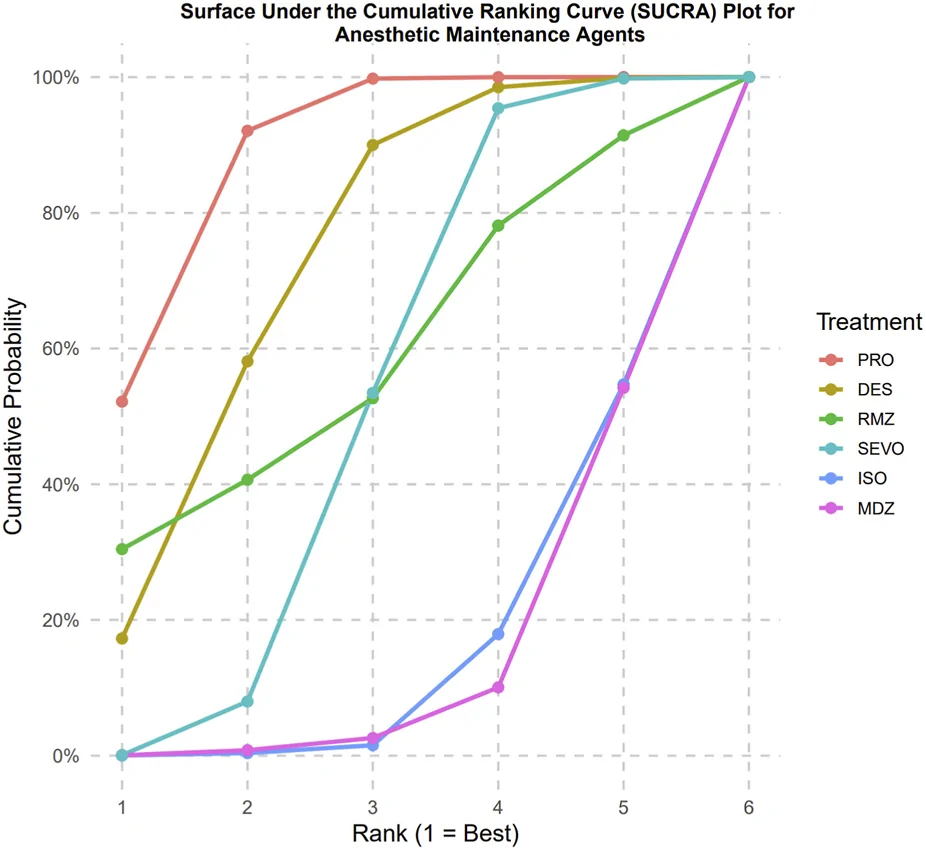
Surface Under the Cumulative Ranking Curve (SUCRA) Plot for Anesthetic Maintenance Agents. SUCRA values indicate relative efficacy ranking. Abbreviations: PRO, propofol; DES, desflurane; RMZ, remimazolam; SEVO, sevoflurane; ISO, isoflurane; MDZ, midazolam.

In the network of adjunctive sedatives/neuroprotective agents, the efficacy rankings of dexmedetomidine (SUCRA = 0.887) and ulinastatin (SUCRA = 0.886) are nearly the same, followed by ketamine (SUCRA = 0.557) > dexamethasone (SUCRA = 0.334); it is notable that minocycline (SUCRA = 0.080) ranks lower than NS (Figure 10).

**FIGURE 10:**
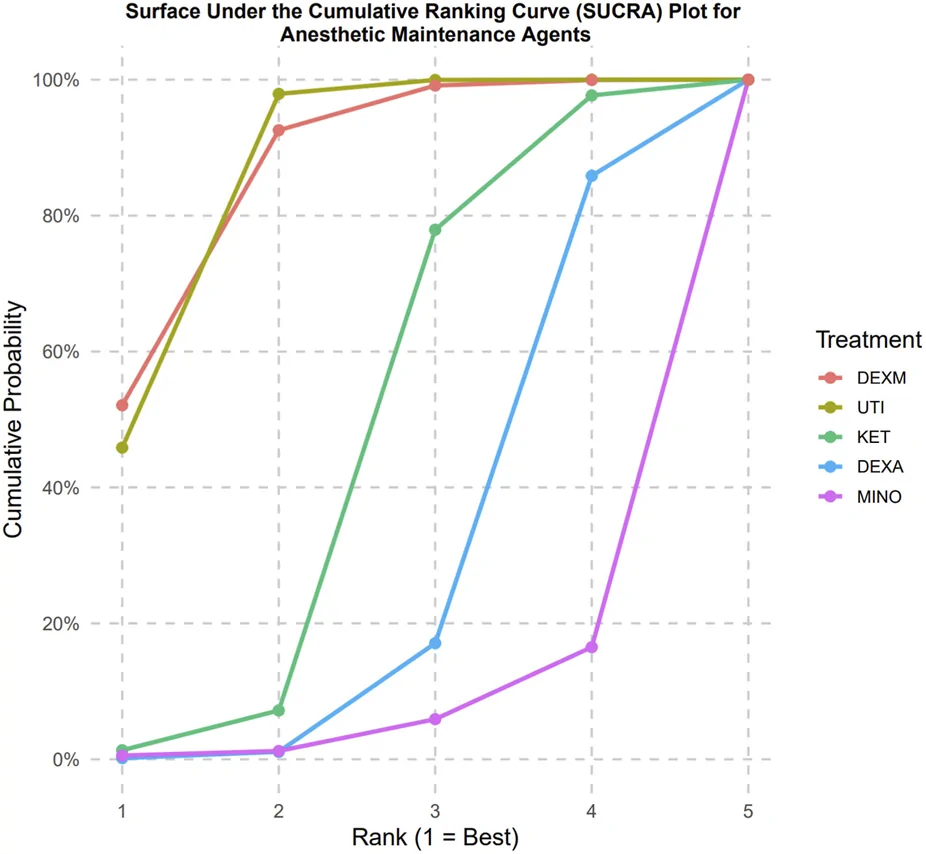
Surface Under the Cumulative Ranking Curve (SUCRA) Plot for Adjunctive Sedatives and Neuroprotective Agents. SUCRA values indicate relative efficacy ranking. Abbreviations: DEX, dexmedetomidine; UTI, ulinastatin; KET, ketamine; DEXA, dexamethasone; MINO, minocycline.

In the analgesic drug network, the SUCRA rankings are as follows: ropivacaine (SUCRA = 0.775) > lidocaine (SUCRA = 0.748) > parecoxib (SUCRA = 0.691) > butorphanol (SUCRA = 0.679) > sufentanil (SUCRA = 0.323) > fentanyl (SUCRA = 0.197). All drugs were compared with normal saline as the control (Figure 11).

**FIGURE 11:**
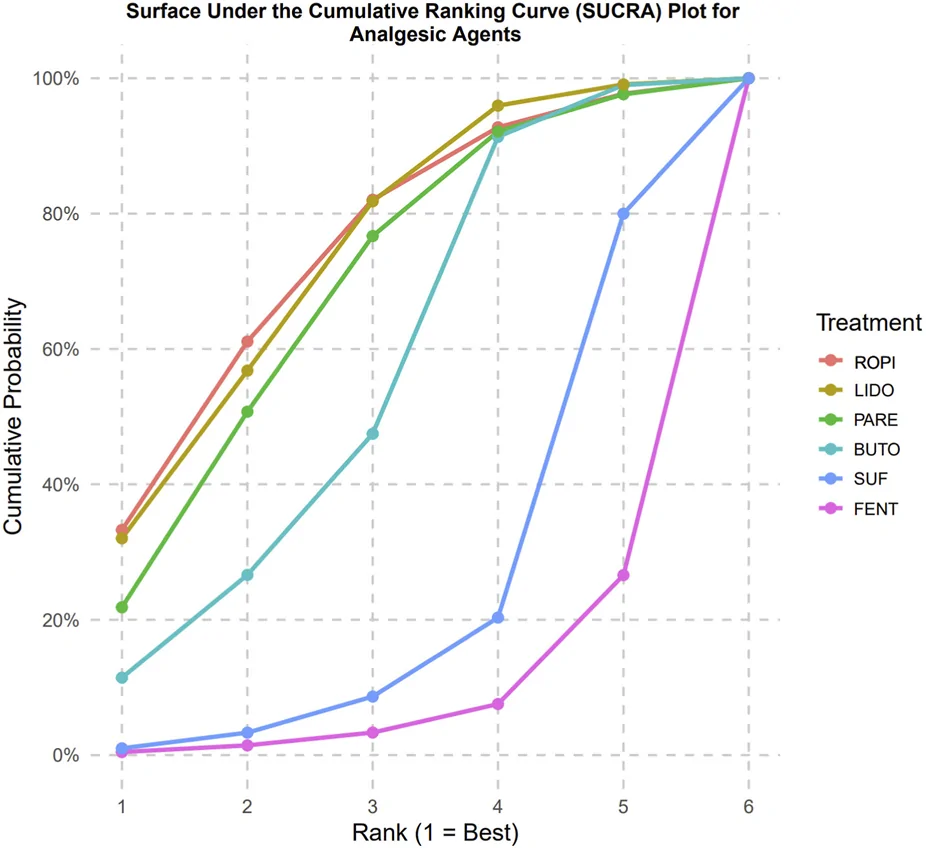
Surface Under the Cumulative Ranking Curve (SUCRA) Plot for Analgesic Agents. SUCRA values indicate relative efficacy ranking. Abbreviations: ROPI, Ropivacaine; LIDO, lidocaine; PARE, parecoxib; BUTO, butorphanol; SUF, sufentanil; FENT, fentanyl.

#### 3.4.6 Subgroup analysis

To examine the consistency of treatment effects under different clinical settings and to explore the potential sources of heterogeneity, we conducted subgroup analyses based on the preset variables. The factors included the category of surgical risk (cardiac surgery, major non-cardiac surgery, and minor/mid-sized surgery) and the type of cognitive assessment tool (MMSE, MoCA, and combined comprehensive neuropsychological tests). After stratification by surgical risk, the relative ranking of drugs within each network was largely consistent across the subgroups, suggesting that the differences in surgical types were not the main source of heterogeneity in this study. After stratification by cognitive assessment tools, the drug ranking patterns among the three subgroups were highly similar, and no systematic influence of the assessment tools on the efficacy ranking was found.

For Network 3, a subgroup analysis was conducted on lidocaine and parecoxib based on the time points of postoperative cognitive assessment. Lidocaine did not significantly reduce the risk of dNCR in the early postoperative period (≤1 day) (RR 1.086, 95% CrI 0.489–2.415, P = 0.839), but showed a protective effect at the 3–7 days assessment (RR 0.339, 95% CrI 0.205–0.560, P < 0.001), and the subgroup differences were statistically significant (P = 0.016). Parecoxib did not show significant differences at the 1–3 days assessment (RR 0.632, 95% CrI 0.328–1.217, P = 0.170), nor at the 3–7 days assessment (RR 0.567, 95% CrI 0.393–0.817, P = 0.002). However, the interaction test between subgroups was not statistically significant (P = 0.777). Although the point estimates of parecoxib at later time points indicated benefits, considering the non-significant subgroup differences and the low quality rating of the evidence, this result should not be overinterpreted (See Supplementary Appendices S11, S12).

#### 3.4.7 Sensitivity analysis

To assess the robustness of the main research results, we conducted a series of sensitivity analyses, as follows:

1. Single trial exclusion method: Each included study was excluded one by one, and then the network estimation was conducted again. The results showed that the SUCRA rankings of all drugs in the three networks did not reverse. In Network 3, ropivacaine was included in only 2 studies. After exclusion, the OR point estimate value fluctuated slightly (range 0.28–0.44), but the SUCRA rankings and effect directions of all drugs did not undergo substantial changes.
2. Model Selection: After replacing the fixed-effect model with the random-effect model, the estimated effect values and 95% credible intervals of various drugs in the three networks were highly consistent with the results of the main analysis. The τ^2^ estimates in the three networks were all close to 0, indicating that the influence of heterogeneity on model selection was weak.
3. Replication of the frequentist framework: The analysis process was re-executed within the frequency analysis framework. The effect directions and SUCRA rankings of all drugs in the three networks were consistent with the results of the Bayesian analysis (specific data omitted).
4. Meta-regression analysis: The surgical type, cognitive assessment tool, publication year, and sample size were included as covariates in the meta-regression model. The results showed that these variables had no significant impact on the effect estimates of all drugs in the three networks (all P values were > 0.05).
5. Trial Sequential Analysis (TSA): For Network 3, the combined event rate of the saline group was used as the control (pC = 19.4%), with α set at 0.05 and the test power at 80%. The results showed that the cumulative sample sizes of lidocaine (461 cases vs. RIS 202 cases) and parecoxib (815 cases vs. RIS 254 cases) had both exceeded the required information quantity (RIS); while ropivacaine (107 cases vs. RIS 185 cases) and butorphanol (130 cases vs. RIS 306 cases) did not reach the RIS threshold.
6. Extension of network consistency test: In the extended network, the I^2^ values of the three networks were 7%, 9%, and 8% respectively. No significant inconsistency was observed overall (except for the comparison of propofol and sevoflurane in Network 1, p = 0.03). The SUCRA rankings of all drugs in the three networks were consistent with the results of the main analysis (specific data omitted).

It is particularly important to note that Network 3 has a star-shaped structure. Its ranking results are entirely dependent on the indirect comparison of each intervention measure with normal saline, lacking direct head-to-head evidence between drugs. Therefore, the ranking conclusion of this network should be regarded as an exploratory result, and interpretation should be cautious.

### 3.5 Quality of evidence

This study systematically evaluated the quality of evidence for three core networks using the CINeMA framework. Each network was judged based on six domains (internal bias in research, reporting bias, indirectness, imprecision, heterogeneity, and incoherence). The detailed results can be found in Supplementary Appendix S9.

In the anesthesia maintenance drug network, the majority of the included comparative evidence had a low to extremely low level of confidence. Only the direct comparison between propofol and sevoflurane reached a high-quality level. In the adjunctive sedation/neuroprotective agent network, the distribution of evidence quality was also uneven. Five pairs of comparisons (ketamine vs. normal saline, dexamethasone vs. minocycline) were of moderate; most of the remaining comparative evidence was weak and was rated as low or extremely low quality. In the analgesic drug network, only four pairs with direct evidence (butorphanol vs. fentanyl, butorphanol vs. normal saline, fentanyl vs. normal saline, ropivacaine vs. sufentanil) reached a moderate; the majority of the other comparisons lacked head-to-head direct evidence and relied solely on indirect comparisons, and were rated as low or extremely low quality. Overall, the majority of the comparisons in the three networks had low to extremely low levels of confidence, concentrated in the low to extremely low range. This result is mainly limited by internal bias in research, reporting bias, imprecision in effect estimation, and the lack of direct evidence support for some comparisons. Therefore, the current conclusions should be regarded as exploratory references and should be interpreted with caution.

### 3.6 Funnel plot and publication bias

In the network of maintenance anesthetic maintenance agents, the funnel plot was basically symmetrical, and the Egger test did not indicate significant publication bias (bias coefficient = 0.42, P = 0.637) (Figure 12).

**FIGURE 12:**
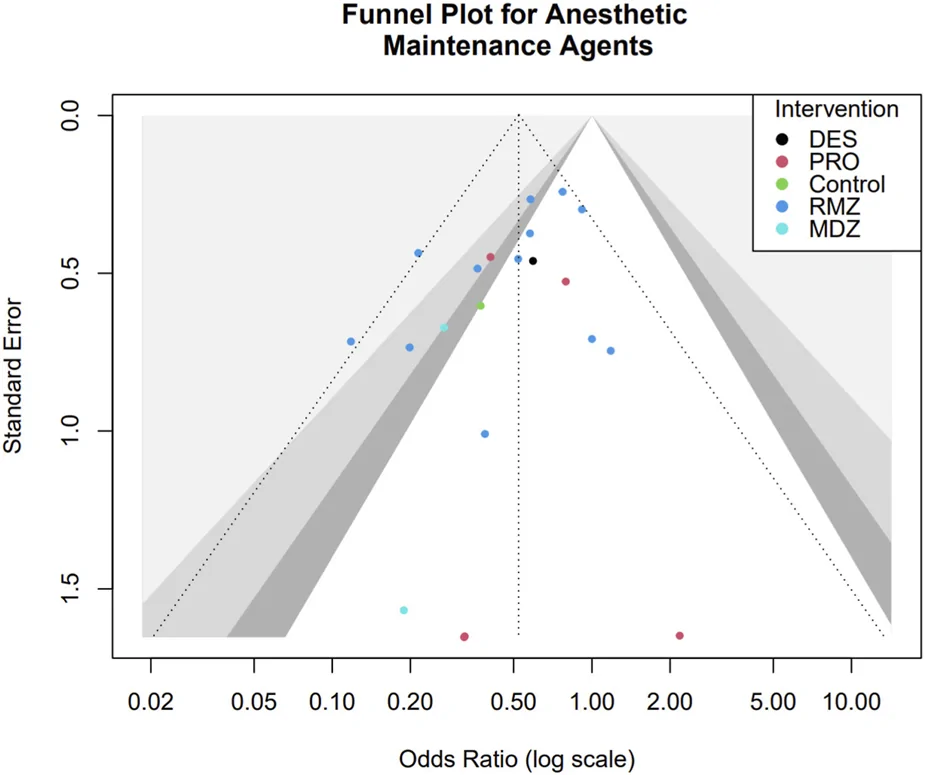
Funnel Plot for Anesthetic maintenance agents. Circle sizes are proportional to study weights. The y-axis represents the standard error; the x-axis shows the odds ratio (log scale). Egger’s test P > 0.05. Abbreviations: PRO, propofol; DES, desflurane; RMZ, remimazolam; SEVO, sevoflurane; ISO, isoflurane; MDZ, midazolam.

In the network of adjunctive sedation/neuroprotective agents, the funnel plot showed asymmetry, and the Egger test detected significant publication bias (bias coefficient = 1.67, P = 0.003). After correction using the trimming and filling method, the combined effect estimate changed from 0.607 (95% CrI: 0.286–0.928) to 0.342 (95% CrI: 0.003–0.681), a reduction of 43.7%. It should be noted that the trimming and filling method relies on the assumption of “symmetry of the funnel plot”, and this assumption could not be independently verified in this study; and the lower limit of the 95% CrI after correction was extremely close to 1.0, with weak statistical significance. Therefore, the original analysis may have overestimated the effect size, and although the potential benefits are still suggested after correction, the uncertainty is large, and it should be interpreted with caution (Figure 13).

**FIGURE 13:**
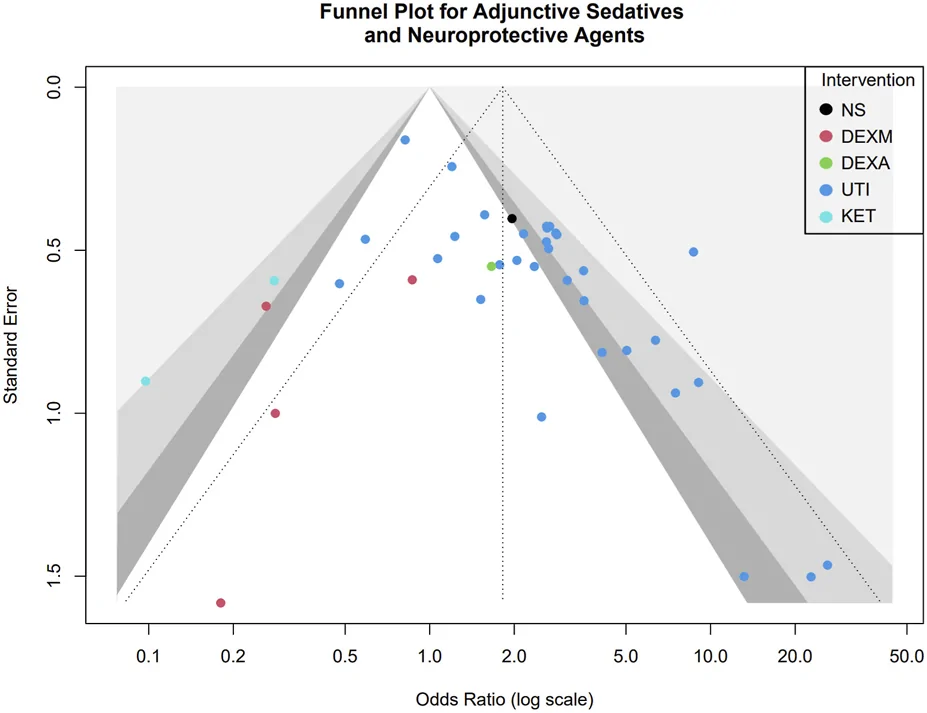
Funnel Plot for adjunctive sedatives and neuroprotective agents. Circle sizes are proportional to study weights. The y-axis represents the standard error; the x-axis shows the odds ratio (log scale). Egger’s test P > 0.05. Abbreviations: PRO, propofol; DES, desflurane; RMZ, remimazolam; SEVO, sevoflurane; ISO, isoflurane; MDZ, midazolam.

In the analgesic drug network, the Egger linear regression test did not detect any significant publication bias (t = 0.57, df = 12, P = 0.581) (Figure 14).

**FIGURE 14:**
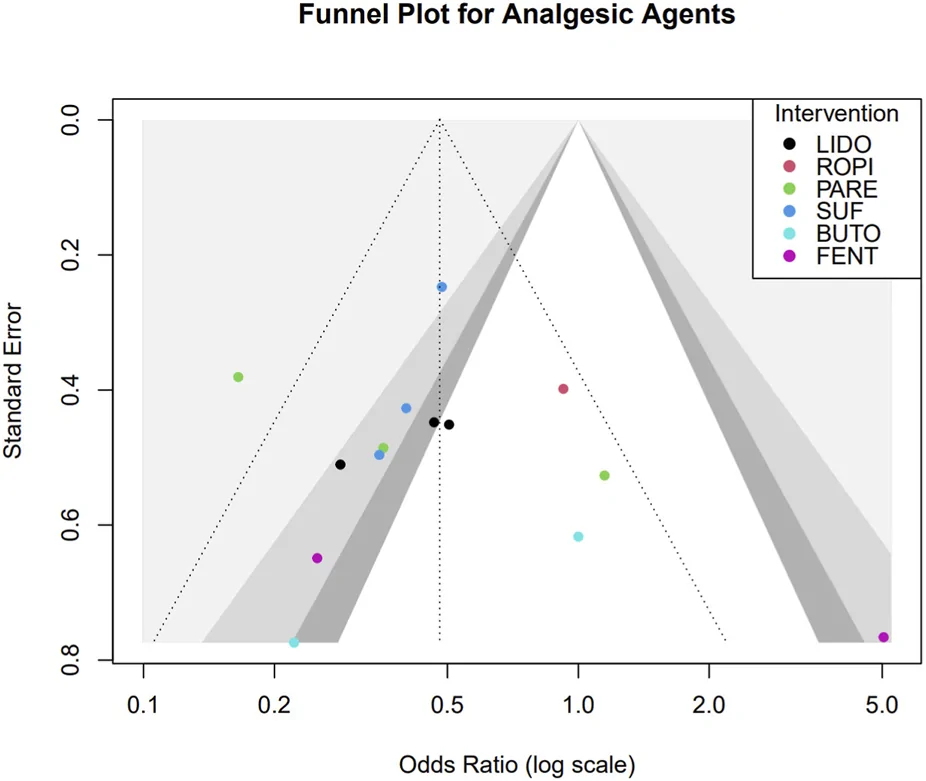
Funnel Plot for analgesic agents. Circle sizes are proportional to study weights. The y-axis represents the standard error; the x-axis shows the odds ratio (log scale). Egger’s test P > 0.05. Abbreviations: ROPI, ropivacaine; LIDO, lidocaine; PARE, parecoxib; BUTO, butorphanol; SUF, sufentanil; FENT, fentanyl.

## 4 Discussion

This study constructed three independent Bayesian network meta-analysis frameworks to compare and rank the relative efficacy of three major types of commonly used clinical drugs - anesthetic maintenance drugs, adjunctive sedatives and neuroprotective drugs, and analgesics - in preventing postoperative delayed neurocognitive recovery (dNCR). To our knowledge, this is the first study to conduct a hierarchical network integration of the evidence for dNCR drug prevention based on clinical medication scenarios.

### 4.1 Anesthetic maintenance agents

Propofol demonstrated the best preventive effect (SUCRA = 0.908). It was significantly superior to sevoflurane (OR 0.60, 95% CrI: 0.44–0.82) and midazolam (OR 0.21, 95% CrI: 0.08–0.51). Most previous studies suggested no significant difference between propofol and sevoflurane in postoperative cognitive function (Guo et al., 2026). However, our study included new evidence with an observation window extended to 30 days after surgery, which might capture later-emerging differences in cognitive recovery. Additionally, the network meta-analysis increased statistical power by combining indirect evidence, which may reveal subtle differences masked in direct comparisons due to insufficient sample size. The node-splitting test showed local inconsistency in the direct comparison between propofol and sevoflurane (P = 0.03). This may be due to heterogeneity in anesthesia depth control, electroencephalogram monitoring use, or patients’ baseline cognitive status across studies (Zeng et al., 2023). Therefore, although propofol ranks higher, a definitive conclusion that “propofol is superior to sevoflurane” cannot be drawn at this time.

Remimazolam (SUCRA = 0.657) ranks higher than sevoflurane and midazolam in this network. As an ultra-short-acting benzodiazepine, it is metabolized by non-specific esterase and does not rely on liver or kidney function. Its hemodynamic stability is superior to propofol (Wang et al., 2025; Shen et al., 2026; Hughes et al., 2025; Dong and Ma, 2025). However, the evidence base for its cognitive protective effect is extremely weak, with only a few studies included and a very low CINeMA rating. Therefore, this ranking should not be interpreted as proof of a cognitive protective advantage. Instead, it should be regarded as a hypothesis requiring verification. In elderly or hemodynamically fragile patients, remimazolam may provide stable anesthesia without increasing—or even while reducing—the risk of dNCR. This finding provides a priority reference for future head-to-head trials with cognitive function as the endpoint (Arias et al., 2025).

Midazolam ranks lowest in this network (SUCRA = 0.258). This is not in conflict with its clinical positioning as a pre-surgical anxiolytic and short-term procedural sedative. In this study, midazolam was used for maintaining general anesthesia, though such use has become quite uncommon in practice. The comparison between midazolam and propofol or sevoflurane is essentially a comparison between different anesthesia regimens—total intravenous anesthesia, inhalation anesthesia, and benzodiazepine-based sedation—rather than a competition between similar drugs (Xu et al., 2023). This result again suggests that for elderly patients requiring general anesthesia, midazolam should be avoided as the main maintenance drug. Its central anticholinergic-like effects and prolonged cognitive inhibition may increase the risk of dNCR.

### 4.2 Auxiliary sedatives and neuroprotective drugs

Dexmedetomidine (DEX, SUCRA = 0.887) and ulinastatin (UTI, SUCRA = 0.886) were significantly superior to other drugs. This finding is consistent with several recent meta-analyses and further consolidates their core adjunctive role in preventing dNCR. However, their evidence bases and applicable populations differ considerably. Dexmedetomidine is more suitable for patients in high-stress states who require continuous postoperative sedation. Its cognitive protective effect is mainly achieved through central anti-inflammatory and sympathetic inhibition mediated by α_2_ receptors (Pan and Yang, 2026). In contrast, ulinastatin is a broad-spectrum serine protease inhibitor that acts primarily on the periphery. It inhibits the systemic inflammatory response triggered by surgical trauma, thereby reducing the “overflow” of pro-inflammatory factors to the central nervous system. This indirectly protects the blood-brain barrier and neural function (Duan et al., 2021). The advantageous population for ulinastatin may not be those requiring sedation, but rather patients undergoing major thoracic or abdominal surgeries who experience significant surgical trauma but do not need long-term postoperative sedation. Therefore, dexmedetomidine and ulinastatin should be viewed as complementary rather than competitive in clinical practice. Clinical decisions should be based on patients’ postoperative sedation needs and surgical trauma severity, rather than solely on SUCRA rankings.

Ketamine (SUCRA = 0.557) and dexamethasone (SUCRA = 0.334) ranked lower. In clinical practice, ketamine’s advantage lies in its rapid antidepressant, analgesic, and opioid-sparing effects, rather than in direct and sustained cognitive protection (Zhou et al., 2019). The core value of dexamethasone is preventing postoperative nausea and vomiting. Its anti-inflammatory effect on cognitive protection is inconsistent and may vary by dose and surgery type (Xue et al., 2026). Therefore, our ranking results should not be interpreted as “ketamine or dexamethasone cannot prevent dNCR”. Rather, they indicate that “current evidence does not sufficiently support their use as preventive medications for dNCR”. Their clinical application should still follow their classic indications.

The SUCRA value of minocycline (0.080) is even lower than that of normal saline. However, its neuroprotective effect is well-documented in basic research, such as inhibiting microglial activation. This efficacy has not been confirmed in clinical studies. Our current results suggest that positive signals from basic research cannot be directly extrapolated to clinical practice (Zhang et al., 2022). The neuroprotective potential of minocycline still requires re-examination through optimized trial designs and larger sample sizes. Any conclusion about “effectiveness” or “ineffectiveness” remains premature.

The Egger test result for Network 2 (P = 0.003) indicates significant publication bias. This suggests that the literature in this field may systematically favor reporting positive results. Consequently, the efficacy of dexmedetomidine and ulinastatin may have been overestimated to some extent. Although the effect direction did not reverse after correction, the stability of the effect estimate declined. This suggests that the true efficacy may still exist but with greater uncertainty. Therefore, caution is warranted when interpreting the “high ranking” of these drugs. Future research should prioritize the complete reporting of negative results to reduce bias interference in evidence synthesis (Xing et al., 2024).

### 4.3 Analgesics

This study included 12 RCTs (2,427 patients). In point estimates, ropivacaine (OR 0.33), lidocaine (OR 0.35), parecoxib (OR 0.40), and butorphanol (OR 0.44) all showed a trend toward reducing dNCR incidence. However, when compared with normal saline, their 95% credible intervals all crossed 1.0, indicating no statistical significance. This suggests that the preventive effect of analgesics on dNCR may be weak. It is also difficult to separate this effect from confounders such as postoperative pain control and opioid-sparing effects (Wang et al., 2022; Shao et al., 2024). This network has a star-shaped structure, with each drug compared indirectly only against normal saline. Direct head-to-head evidence among active drugs is lacking. Therefore, ropivacaine’s highest SUCRA ranking does not mean it is superior to other drugs. It merely reflects a slightly larger effect size in the evidence against normal saline. The value of this ranking lies in providing a priority reference for future head-to-head trials, rather than guiding clinical medication selection. The CINeMA assessment also shows that most comparison evidence in this network is of “low” or “very low” quality, further limiting the certainty of the ranking.

Postoperative pain itself is an important risk factor for dNCR (Yuan et al., 2025). It can activate the HPA axis and promote neuroinflammation. Additionally, pain-induced sleep deprivation and stress may directly impair cognitive function (Zhao et al., 2021). Therefore, a reasonable analgesic strategy should theoretically reduce the risk of dNCR indirectly. However, our study did not show significant benefits. Possible reasons include the following. First, there is considerable heterogeneity in analgesic regimens, administration timing, and doses across studies. Second, dNCR involves multiple pathways—neuroinflammation, oxidative stress, and synaptic plasticity changes—and simple analgesia may not adequately cover all of these aspects (Su et al., 2026).

Subgroup analysis showed that lidocaine exhibited a protective effect 3–7 days after surgery (RR 0.339, P < 0.001). There was a statistically significant difference between the early (≤1 day) and later groups (P = 0.016) (Fernández-Martínez et al., 2025). This time-dependent characteristic is consistent with lidocaine’s anti-inflammatory mechanism. Lidocaine inhibits neutrophil activation and reduces pro-inflammatory factor release. The inflammatory cascade peaks within hours to days after surgery and can continuously affect neurological function (Shin et al., 2024). Parecoxib showed a beneficial trend at 1–3 days and 3–7 days after surgery. However, the interaction between subgroups was not statistically significant (P = 0.777), and the evidence was weak. A large 2025 RCT in hyperlipidemic patients found that a single preoperative 40 mg dose of parecoxib reduced POD incidence (HR 0.491, 95% CrI 0.318–0.759). However, POD and dNCR differ substantially in diagnostic time window and pathophysiology. Whether this result can be extrapolated to the dNCR population remains to be verified. This evidence gap highlights the current lack of large-sample RCTs in the dNCR field that use this outcome as the primary endpoint.

The ranking results for analgesic drugs should be regarded as hypothesis-generating findings. They should not be interpreted as confirmations of therapeutic efficacy in the evidence-based Medicine sense.

## 5 Limitation

1. There is considerable clinical heterogeneity among the studies, manifested in differences in surgical types, cognitive assessment tools, and the time points for outcome observation. Although subgroup analysis and meta-regression did not find that these factors systematically affected the main effect direction, the number of studies in each subgroup was limited, making it difficult to fully control for confounding bias. The surgical risk subgroup analysis was based on the surgical name for indirect derivation and was an exploratory analysis, and its results should be interpreted with caution.
2. The CINeMA assessment shows that, except for a few direct comparisons of moderate to high grades, most evidence relies on indirect comparisons, and the overall quality is low. This highlights the urgent need for head-to-head RCTs with dNCR as the primary endpoint and provides a reference for subsequent trial design.
3. The analgesic drug network is star-shaped, with each active drug only compared with normal saline, lacking direct comparisons among active drugs. Under this structure, the SUCRA ranking is statistically equivalent to a group of independent indirect comparisons based on a common reference, and cannot effectively infer the relative superiority of active drugs. The ranking results should be regarded as a preliminary reference for hypothesis generation.
4. The network of adjunctive sedation/neuroprotection drugs has significant publication bias (Egger test, P = 0.003). After correction using the trimming and filling method, the effect size decreased significantly, and the lower limit of the 95% credible interval was close to 1.0. Although the effect direction did not reverse, the correction method relies on the unverifiable assumption of “symmetry of the funnel plot”, and this result should be interpreted with caution.
5. The drug classification based on clinical scenarios in this study has certain subjectivity. Some drugs have multiple functions, and the heterogeneity of administration timing and schemes has not been finely handled.

## 6 Conclusion

This study compared the prophylactic efficacy of three drug categories—maintenance anesthetics, adjuvant sedatives, and analgesics—against postoperative delayed neurocognitive recovery (dNCR), providing evidence-based guidance for clinical drug selection. Future research should build on these findings by investigating how to optimally combine preferred agents with distinct mechanisms of action, aiming to develop more effective, individualized, multimodal neuroprotective strategies and further advance the prevention and management of dNCR.

## Data Availability

The original contributions presented in the study are included in the article/Supplementary Material, further inquiries can be directed to the corresponding author.
