# Supplementary material for: Optimal prophylaxis for perioperative drug-induced delayed neurocognitive recovery: a Bayesian network meta-analysis of 18 interventions with mechanism-based stratification
Source: Front Pharmacol. 2026 Sep 23;17:1877360. doi: 10.3389/fphar.2026.1877360 (PMC13642292; doi:10.3389/fphar.2026.1877360)
Supplement: Supplementary file 1 [file Supplementaryfile1.docx]

# **Supplementary Appendix**

# **APPENDIX 1** Search Strategye

**A.Pubmed**

| #1 | ("Delirium"[Mesh]) OR (((((((((((Subacute Delirium) OR (Delirium, Subacute)) OR (Deliriums, Subacute)) OR (Subacute Deliriums)) OR (Delirium of Mixed Origin)) OR (Mixed Origin Delirium)) OR (Mixed Origin Deliriums)) OR (Emergence Delirium)) OR (Confusion)) OR (Disorientation)) OR (Bewilderment)) |
| --- | --- |
| #2 | ((((((((((((((((Melatonin) OR (Receptors, Melatonin)) OR (Receptor, Melatonin)) OR (Melatonin Receptor)) OR (Melatonin Receptors)) OR (Receptor, Melatonin, MT3)) OR (Melatonin MT3 Receptor)) OR (MT3 Receptor, Melatonin)) OR (Receptor, Melatonin MT3)) OR (MT3 Receptor)) OR (Receptor, MT3)) OR (Receptor, Melatonin, 1C)) OR (Receptor, Mel1c)) OR (Mel1c Receptor)) OR (Mel(1c) Receptor)) OR (Receptor, Melatonin, MT1)) OR (Receptor, Melatonin, MT2) |
| #3 | ((((((((((((((((((((((((((((((((((((((((((((((((((((((((((((((((((((((((((((((((((((((((((Antipsychotic Agents) OR (Tranquillizing Agents, Major)) OR (Major Tranquillizing Agents)) OR (Major Tranquilizer)) OR (Tranquilizer, Major)) OR (Antipsychotic Drug)) OR (Drug, Antipsychotic)) OR (Antipsychotic Agent)) OR (Agent, Antipsychotic)) OR (Antipsychotic Medication)) OR (Medication, Antipsychotic)) OR (Neuroleptic Agent)) OR (Agent, Neuroleptic)) OR (Neuroleptic Drug)) OR (Drug, Neuroleptic)) OR (Neuroleptic)) OR (Antipsychotic)) OR (Antipsychotic Drugs)) OR (Antipsychotics)) OR (Major Tranquilizers)) OR (Neuroleptic Agents)) OR (Neuroleptic Drugs)) OR (Neuroleptics)) OR (Tranquilizing Agents, Major)) OR (Major Tranquilizing Agents)) OR (Antipsychotic Effect)) OR (Effect, Antipsychotic)) OR (Antipsychotic Effects)) OR (Haloperidol)) OR (Haldol)) OR (Quetiapine)) OR (Ethanol,2-(2-(4-dibenzo(b,f)(1,4)thiazepin-11-yl-1-piperazinyl)ethoxy)-,(E)-2-butenedioate (2:1) (salt))) OR (Seroquel)) OR (ICI 204,636)) OR (ICI-204636)) OR (ICI204636)) OR (ICI 204636)) OR (2-(2-(4-dibenzo(b,f)(1,4)thiazepine-11-yl-1-piperazinyl)ethoxy)ethanol )) OR (Risperidone)) OR (Risperdal Consta)) OR (Consta, Risperdal)) OR (Risperidal)) OR (R-64,766)) OR (R 64,766)) OR (R64,766)) OR (R-64766)) OR (R 64766)) OR (R64766)) OR (Clozapine)) OR (Clozaril)) OR (Leponex)) OR (Ziprasidone)) OR (ziprazidone)) OR (5-(2-(4-(3-benzisothiazolyl)piperazinyl)ethyl)-6-chloro-1,3-dihydro-2H-indol-2-one)) OR (ziprasidone hydrochloride)) OR (CP 88059-01)) OR (CP-88,059-1)) OR (CP-88,059-01)) OR (ziprasidone hydrochloride, monohydrate)) OR (Geodon)) OR (CP 88059)) OR (CP-88,059)) OR (Chlorpromazine)) OR (Thorazine)) OR (Aminazine)) OR (Largactil)) OR (Chlordelazine)) OR (Chlorpromazine Hydrochloride)) OR (Hydrochloride, Chlorpromazine)) OR (Contomin)) OR (Fenactil)) OR (Propaphenin)) OR (Chlorazine)) OR (Chlordiazepoxide)) OR (7-Chloro-N-methyl-5-phenyl-3H-1,4-benzodiazepin-2-amine 4-oxide)) OR (7 Chloro N methyl 5 phenyl 3H 1,4 benzodiazepin 2 amine 4 oxide)) OR (Methaminodiazepoxide)) OR (7-Chloro-2-methylamino-5-phenyl-3H-1,4-benzodiazepine-4-oxide)) OR (7 Chloro 2 methylamino 5 phenyl 3H 1,4 benzodiazepine 4 oxide)) OR (Chlordiazepoxide Hydrochloride)) OR (Hydrochloride, Chlordiazepoxide)) OR (Chlordiazepoxide Monohydrochloride)) OR (Monohydrochloride, Chlordiazepoxide)) OR (Librium)) OR (Chlozepid)) OR (Elenium)) OR (Chlordiazepoxide Hydrobromide)) OR (Hydrobromide, Chlordiazepoxide)) OR (Chlordiazepoxide Perchlorate)) OR (Perchlorate, Chlordiazepoxide)) OR (((((((((((((((((((((((Sultopride) OR (4-Amino-N-((1-ethyl-2-pyrrolidinyl)methyl)-5-(ethylsulfonyl)-2-methoxybenzamide)) OR (DAN 2163)) OR (DAN-2163)) OR (Solian)) OR (Sultopride)) OR (N-(Ethyl-1-pyrrolidinyl- 2-methyl)methoxy-2-ethylsulfonyl-5-benzamide)) OR (LIN 1418)) OR (LIN-1418)) OR (Barnetil)) OR (Sultopride Hydrochloride)) OR (Sulpiride)) OR (Olanzapine)) OR (2-Methyl-4-(4-methyl-1-piperazinyl)-10H-thieno(2,3-b)(1,5)benzodiazepine)) OR (LY-170052)) OR (LY 170052)) OR (LY170052)) OR (Zyprexa)) OR (Zolafren)) OR (LY 170053)) OR (Olanzapine Pamoate))) OR (Amisulpride)) |
| #4 | (((((((((((((((Pregabalin) OR ((S)-3-(aminomethyl)-5-methylhexanoic acid)) OR (3-isobutyl GABA)) OR (3 isobutyl GABA)) OR (GABA, 3-isobutyl)) OR (3-(aminomethyl)-5-methylhexanoic acid)) OR ((R-)-3-isobutyl GABA)) OR ((S+)-3-isobutyl GABA)) OR (Lyrica)) OR (CI 1008)) OR (1008, CI)) OR (CI-1008)) OR (CI1008Cimetidine)) OR (Chlormethiazole)) OR (Clomethiazole)) OR (Distraneurin) |
| #5 | (((((((((((Ketamine) OR (2-(2-Chlorophenyl)-2-(methylamino)cyclohexanone)) OR (CI-581)) OR (CI 581)) OR (CI581)) OR (Ketalar)) OR (Ketaset)) OR (Ketanest)) OR (Calipsol)) OR (Kalipsol)) OR (Calypsol)) OR (Ketamine) |
| #6 | ((((((((((((((((((((((((((((((((((((Baclofen) OR (Baclophen)) OR (PCP-GABA)) OR (beta-(Aminomethyl)-4-chlorobenzenepropanoic Acid)) OR (Chlorophenyl GABA)) OR (GABA, Chlorophenyl)) OR (beta-(p-Chlorophenyl)-gamma-aminobutyric Acid)) OR (Clofen)) OR (Gen-Baclofen)) OR (Gen Baclofen)) OR (GenBaclofen)) OR (Genpharm)) OR (Apo-Baclofen)) OR (Apo Baclofen)) OR (ApoBaclofen)) OR (Atrofen)) OR (Baclofène-Irex)) OR (Baclofène Irex)) OR (BaclofèneIrex)) OR (Baclofen AWD)) OR (AWD, Baclofen)) OR (Lebic)) OR (Lioresal)) OR (Liorésal)) OR (Nu-Baclo)) OR (Nu Baclo)) OR (NuBaclo)) OR (PMS-Baclofen)) OR (PMS Baclofen)) OR (PMSBaclofen)) OR (Baclospas)) OR (Ba-34,647)) OR (Ba34,647)) OR (Ba-34647)) OR (Ba34647)) OR (CIBA-34,647-BA)) OR (CIBA34,647BA) |
| #7 | (((((((((((((((((((((((((((Clonidine) OR (Klofenil)) OR (Clofenil)) OR (Chlophazolin)) OR (Clonidine Dihydrochloride)) OR (Dihydrochloride, Clonidine)) OR (Clonidine Hydrochloride)) OR (Hydrochloride, Clonidine)) OR (Clonidine Monohydrochloride)) OR (Monohydrochloride, Clonidine)) OR (Clonidine Monohydrobromide)) OR (Monohydrobromide, Clonidine)) OR (ST-155)) OR (ST 155)) OR (ST155)) OR (Gemiton)) OR (Hemiton)) OR (Isoglaucon)) OR (Klofelin)) OR (Clofelin)) OR (Clopheline)) OR (M-5041T)) OR (M 5041T)) OR (M5041T)) OR (Catapres)) OR (Catapresan)) OR (Catapressan)) OR (Dixarit) |
| #8 | (((((((((((((((((((((((((((((((((((((((((Anesthetics) OR (Anesthetic Agents)) OR (Agents, Anesthetic)) OR (Anesthetic Drugs)) OR (Drugs, Anesthetic)) OR (Anesthetic)) OR (Anesthetic Effect)) OR (Effect, Anesthetic)) OR (Anesthetic Effects)) OR (Effects, Anesthetic)) OR (Morphine)) OR (Morphia)) OR (Morphine Chloride)) OR (Chloride, Morphine)) OR (Morphine Sulfate)) OR (Sulfate, Morphine)) OR (SDZ 202-250)) OR (SDZ 202 250)) OR (SDZ 202250)) OR (SDZ202-250)) OR (SDZ202 250)) OR (SDZ202250)) OR (Morphine Sulfate (2:1), Pentahydrate)) OR (MS Contin)) OR (Contin, MS)) OR (Oramorph SR)) OR (Duramorph)) OR (Morphine Sulfate (2:1), Anhydrous)) OR (Pethidine)) OR (Isonipecain)) OR (Dolsin)) OR (Dolosal)) OR (Dolin)) OR (Operidine EPJ-I)) OR (Operidine EPJ I)) OR (Dolantin)) OR (Dolargan)) OR (Meperidine Hydrochloride)) OR (Lidol)) OR (Lydol)) OR (Demerol)) OR (Dolcontral) |
| #9 | (((((((((((((Dexamethasone) OR (Methylfluorprednisolone)) OR (Hexadecadrol)) OR (Decameth)) OR (Decaspray)) OR (Dexasone)) OR (deepak)) OR (Maxidex)) OR (Millicorten)) OR (Oradexon)) OR (Decaject)) OR (Decaject-L.A.)) OR (Decaject L.A.)) OR (Hexadrol) |
| #10 | (((((((((((((((((((((((((((((Sedative) OR (Sedatives and Hypnotics)) OR (Sedative and Hypnotic)) OR (Hypnotic and Sedative)) OR (Hypnotic Effect)) OR (Effect, Hypnotic)) OR (Hypnotic Effects)) OR (Effects, Hypnotic)) OR (Sedatives)) OR (Hypnotics)) OR (Hypnotic)) OR (Sedative Effect)) OR (Effect, Sedative)) OR (Sedative Effects)) OR (Effects, Sedative)) OR (Sleep Aids, Pharmaceutical)) OR (Orexin Receptor Antagonists)) OR (Dexmedetomidine)) OR (MPV-1440)) OR (MPV 1440)) OR (MPV1440)) OR (Precedex)) OR (Dexdomitor)) OR (Sedadex)) OR (Sileo)) OR (Cepedex)) OR (Dexdor)) OR (Dexmedetomidine Hydrochloride)) OR (Hydrochloride, Dexmedetomidine)) OR (Igalmi) |
| #11 | ((((((((((Suvorexant) OR ((4-(5-chloro-1,3-benzoxazol-2-yl)-7-methyl-1,4-diazepan-1-yl)(5-methyl-2-(2H-1,2,3-triazol-2-yl)phenyl)methanone)) OR (methanone, ((7R)-4-(5-chloro-2-benzoxazolyl)hexahydro-7-methyl-1H-1,4-diazepin-1-yl)(5-methyl-2-(2H-1,2,3-triazol-2-yl)phenyl)-)) OR (MK 4305)) OR (MK4305)) OR (MK-4305)) OR (Belsomra)) OR (Ramelteon)) OR ((S)-N-(2-(1,6,7,8-tetrahydro-2H-indeno-(5,4)furan-8-yl)ethyl)propionamide)) OR (TAK-375)) OR (Rozerem) |
| #12 | (((((((((((((((((((((((((((((((((((((((((((((((((((((((((((((((((((((((((((((((((Benzodiazepine) OR (Benzodiazepine Compounds)) OR (Benzodiazepine)) OR (Lorazepam)) OR (Ativan)) OR (Orfidal Wyeth)) OR (Wyeth, Orfidal)) OR (Témesta)) OR (Tolid)) OR (Donix)) OR (Duralozam)) OR (Durazolam)) OR (Idalprem)) OR (Laubeel)) OR (Lorazep Von Ct)) OR (Von Ct, Lorazep)) OR (Lorazepam Medical)) OR (Medical, Lorazepam)) OR (Lorazepam-Neuraxpharm)) OR (Lorazepam Neuraxpharm)) OR (Lorazepam-Ratiopharm)) OR (Lorazepam Ratiopharm)) OR (Novo-Lorazem)) OR (Novo Lorazem)) OR (Nu-Loraz)) OR (Nu Loraz)) OR (Sedicepan)) OR (Sinestron)) OR (WY-4036)) OR (WY 4036)) OR (WY4036)) OR (Apo-Lorazepam)) OR (Apo Lorazepam)) OR (Somagerol)) OR (Temesta)) OR (Diazepam)) OR (7-Chloro-1,3-dihydro-1-methyl-5-phenyl-2H-1,4-benzodiazepin-2-one)) OR (Diazemuls)) OR (Faustan)) OR (Valium)) OR (Seduxen)) OR (Sibazon)) OR (Stesolid)) OR (Apaurin)) OR (Relanium)) OR (Oxazepam)) OR (Midazolam)) OR (Midazolam Maleate)) OR (Maleate, Midazolam)) OR (Dormicum)) OR (Versed)) OR (Midazolam Hydrochloride)) OR (Hydrochloride, Midazolam)) OR (Ro 21-3981)) OR (Ro 21 3981)) OR (Ro 213981)) OR (Estazolam)) OR (Tasedan)) OR (ProSom)) OR (D-40TA)) OR (D40TA)) OR (Nuctalon)) OR (Alprazolam)) OR (Alprazolan)) OR (Trankimazin)) OR (U-31,889)) OR (U31,889)) OR (Xanax)) OR (Alprox)) OR (Esparon)) OR (Cassadan)) OR (D-65MT)) OR (D65MT)) OR (Kalma)) OR (Novo-Alprazol)) OR (Novo Alprazol)) OR (Nu-Alpraz)) OR (Nu Alpraz)) OR (Ralozam)) OR (Tafil)) OR (Apo-Alpraz)) OR (Apo Alpraz) |
| #13 | ((((((((((((((Carbamazepine) OR (Carbamazepine Acetate)) OR (Carbamazepine Dihydrate)) OR (Carbamazepine Hydrochloride)) OR (Carbamazepine L-Tartrate (4:1))) OR (Carbamazepine Anhydrous)) OR (Carbamazepine Sulfate (2:1))) OR (Carbazepin)) OR (Epitol)) OR (Finlepsin)) OR (Neurotol)) OR (Tegretol)) OR (Amizepine)) OR (Carbamazepine Phosphate)) OR (((((((((((((((Gabapentin) OR (1-(Aminomethyl)cyclohexaneacetic Acid)) OR (Neurontin)) OR (Gabapentin Hexal)) OR (Convalis)) OR (Gabapentin-Ratiopharm)) OR (Gabapentin Ratiopharm)) OR (Novo-Gabapentin)) OR (Novo Gabapentin)) OR (NovoGabapentin)) OR (PMS-Gabapentin)) OR (Apo-Gabapentin)) OR (Apo Gabapentin)) OR (ApoGabapentin)) OR (Gabapentin Stada)) |
| #14 | (((((((((((((((((((((((((((Sevoflurane) OR (Fluoromethyl-2,2,2-trifluoro-1-(trifluoromethyl)ethyl Ether)) OR (Fluoromethyl Hexafluoroisopropyl Ether)) OR (Sevorane)) OR (Ultane)) OR (BAX 3084)) OR (Propofol)) OR (2,6-Diisopropylphenol)) OR (2,6 Diisopropylphenol)) OR (2,6-Bis(1-methylethyl)phenol)) OR (Disoprofol)) OR (Diprivan)) OR (Disoprivan)) OR (Fresofol)) OR (ICI-35,868)) OR (ICI 35,868)) OR (ICI35,868)) OR (ICI-35868)) OR (ICI 35868)) OR (ICI35868)) OR (Ivofol)) OR (Propofol Fresenius)) OR (Propofol MCT)) OR (Propofol Rovi)) OR (Propofol-Lipuro)) OR (Recofol)) OR (Aquafol)) OR (Propofol Abbott) |
| #15 | ((((((((((((((((((((((((((((((Cimetidine) OR (N-Cyano-N'-methyl-N''-(2-(((5-methyl-1H-imidazol-4-yl)methyl)thio)ethyl)guanidine)) OR (Biomet)) OR (Biomet400)) OR (Cimetidine Hydrochloride)) OR (Hydrochloride, Cimetidine)) OR (Cimetidine HCl)) OR (HCl, Cimetidine)) OR (Histodil)) OR (SK and F-92334)) OR (SK and F 92334)) OR (SK and F92334)) OR (SKF-92334)) OR (SKF 92334)) OR (SKF92334)) OR (Tagamet)) OR (Altramet)) OR (Eureceptor)) OR (Ranitidine)) OR (Ranitidin)) OR (N(2-(((5-((Dimethylamino)methyl)-2-furanyl)methyl)thio)ethyl)-N'-methyl-2-nitro-1,1-ethenediamine)) OR (Biotidin)) OR (Ranisen)) OR (Sostril)) OR (Zantac)) OR (Zantic)) OR (AH-19065)) OR (AH 19065)) OR (AH19065)) OR (Ranitidine Hydrochloride)) OR (Hydrochloride, Ranitidine) |
| #16 | #2 OR #3 OR #4 OR #5 OR #6 OR #7 OR #8 OR #9 OR #10 OR #11 OR #12 OR #13 OR #14 OR #15 |
| #17 | (Randomized controlled trial) OR (controlled clinical trial) OR (Randomized) OR (Randomised) OR (Placebo) OR (Controlled) OR (Random*) OR (Trial) OR Blind* OR Mask* |
| 18 | #1 AND #16 AND #17 |

**B.The Cochrane Library**

| #1 | MeSH descriptor: [Delirium] explode all trees |
| --- | --- |
| #2 | (”Subacute Delirium“):ti,ab,kw OR ("Delirium, Subacute"):ti,ab,kw OR (“Deliriums, Subacute”):ti,ab,kw OR (“Subacute Deliriums”):ti,ab,kw OR (“Delirium of Mixed Origin”):ti,ab,kw OR (“Mixed Origin Delirium”):ti,ab,kw OR (“Mixed Origin Deliriums”):ti,ab,kw OR (“Emergence Delirium”):ti,ab,kw OR (Confusion):ti,ab,kw OR (Disorientation):ti,ab,kw OR (Bewilderment):ti,ab,kw |
| #3 | #1 OR #2 |
| #4 | MeSH descriptor: [Melatonin] explode all trees |
| #5 | MeSH descriptor: [Receptors, Melatonin] explode all trees |
| #6 | ("Receptor, Melatonin"):ti,ab,kw OR ("Melatonin Receptor"):ti,ab,kw OR ("Melatonin Receptors"):ti,ab,kw OR ("Receptor, Melatonin, MT3"):ti,ab,kw OR ("Melatonin MT3 Receptor"):ti,ab,kw OR ("MT3 Receptor, Melatonin"):ti,ab,kw OR ("Receptor, Melatonin MT3"):ti,ab,kw OR ("MT3 Receptor"):ti,ab,kw OR ("Receptor, MT3"):ti,ab,kw OR ("Receptor, Melatonin, 1C"):ti,ab,kw OR ("Receptor, Mel1c"):ti,ab,kw OR ("Mel1c Receptor"):ti,ab,kw OR ("Mel(1c) Receptor"):ti,ab,kw OR ("Receptor, Melatonin, MT1"):ti,ab,kw OR ("Receptor, Melatonin, MT2"):ti,ab,kw |
| #7 | #4 OR #5 OR #6 |
| #8 | MeSH descriptor: [Antipsychotic Agents] explode all trees |
| #9 | ("Tranquillizing Agents, Major"):ti,ab,kw OR ("Major Tranquillizing Agents"):ti,ab,kw OR ("Major Tranquilizer"):ti,ab,kw OR ("Tranquilizer, Major"):ti,ab,kw OR ("Antipsychotic Drug"):ti,ab,kw OR ("Drug, Antipsychotic"):ti,ab,kw OR ("Antipsychotic Agent"):ti,ab,kw OR ("Agent, Antipsychotic"):ti,ab,kw OR ("Antipsychotic Medication"):ti,ab,kw OR ("Medication, Antipsychotic"):ti,ab,kw OR ("Neuroleptic Agent"):ti,ab,kw OR ("Agent, Neuroleptic"):ti,ab,kw OR ("Neuroleptic Drug"):ti,ab,kw OR ("Drug, Neuroleptic"):ti,ab,kw OR (Neuroleptic):ti,ab,kw OR (Antipsychotic):ti,ab,kw OR ("Antipsychotic Drugs"):ti,ab,kw OR (Antipsychotics):ti,ab,kw OR ("Major Tranquilizers"):ti,ab,kw OR ("Neuroleptic Agents"):ti,ab,kw OR ("Neuroleptic Drugs"):ti,ab,kw OR (Neuroleptics):ti,ab,kw OR ("Tranquilizing Agents, Major"):ti,ab,kw OR ("Major Tranquilizing Agents"):ti,ab,kw OR ("Antipsychotic Effect"):ti,ab,kw OR ("Effect, Antipsychotic"):ti,ab,kw OR ("Antipsychotic Effects"):ti,ab,kw |
| #10 | #8 OR #9 |
| #11 | MeSH descriptor: [Haloperidol] explode all trees |
| #12 | (Haldol):ti,ab,kw |
| #13 | #11 OR #12 |
| #14 | MeSH descriptor: [Quetiapine Fumarate] explode all trees |
| #15 | ("Ethanol,2-(2-(4-dibenzo(b,f)(1,4)thiazepin-11-yl-1-piperazinyl)ethoxy)-,(E)-2-butenedioate (2:1) (salt)"):ti,ab,kw OR (Seroquel):ti,ab,kw OR ("ICI 204,636"):ti,ab,kw OR ("ICI-204636"):ti,ab,kw OR ("ICI204636"):ti,ab,kw OR ("ICI 204636"):ti,ab,kw OR (Quetiapine):ti,ab,kw OR ("2-(2-(4-dibenzo(b,f)(1,4)thiazepine-11-yl-1-piperazinyl)ethoxy)ethanol "):ti,ab,kw |
| #16 | #14 OR #15 |
| #17 | MeSH descriptor: [Risperidone] explode all trees |
| #18 | ("Risperdal Consta"):ti,ab,kw OR ("Consta, Risperdal"):ti,ab,kw OR (Risperidal):ti,ab,kw OR ("R-64,766"):ti,ab,kw OR ("R 64,766"):ti,ab,kw OR ("R64,766"):ti,ab,kw OR ("R-64766"):ti,ab,kw OR ("R 64766"):ti,ab,kw OR (R64766):ti,ab,kw |
| #19 | #17 OR #18 |
| #20 | MeSH descriptor: [Clozapine] explode all trees |
| #21 | (Clozaril):ti,ab,kw OR (Leponex):ti,ab,kw |
| #22 | #20 OR #21 |
| #23 | MeSH descriptor: [Ziprasidone] explode all trees |
| #24 | (ziprazidone):ti,ab,kw OR (“ziprasidone hydrochloride”):ti,ab,kw OR (Geodon):ti,ab,kw OR ("CP 88059"):ti,ab,kw OR (CP-88,059):ti,ab,kw |
| #25 | #23 OR #24 |
| #26 | MeSH descriptor: [Chlorpromazine] explode all trees |
| #27 | (Thorazine):ti,ab,kw OR (Aminazine):ti,ab,kw OR (Largactil):ti,ab,kw OR ("Chlorpromazine Hydrochloride"):ti,ab,kw OR ("Hydrochloride, Chlorpromazine"):ti,ab,kw OR (Contomin):ti,ab,kw OR (Fenactil):ti,ab,kw OR (Propaphenin):ti,ab,kw OR (Chlorazine):ti,ab,kw |
| #28 | #26 OR #27 |
| #29 | MeSH descriptor: [Chlordiazepoxide] explode all trees |
| #30 | ("7-Chloro-N-methyl-5-phenyl-3H-1,4-benzodiazepin-2-amine 4-oxide"):ti,ab,kw OR ("7 Chloro N methyl 5 phenyl 3H 1,4 benzodiazepin 2 amine 4 oxide"):ti,ab,kw OR ("Methaminodiazepoxide"):ti,ab,kw OR ("7-Chloro-2-methylamino-5-phenyl-3H-1,4-benzodiazepine-4-oxide"):ti,ab,kw OR ("7 Chloro 2 methylamino 5 phenyl 3H 1,4 benzodiazepine 4 oxide"):ti,ab,kw OR ("Chlordiazepoxide Hydrochloride"):ti,ab,kw OR (Hydrochloride, Chlordiazepoxide):ti,ab,kw OR ("Chlordiazepoxide Monohydrochloride"):ti,ab,kw OR ("Monohydrochloride, Chlordiazepoxide"):ti,ab,kw OR (Librium):ti,ab,kw OR (Chlozepid):ti,ab,kw OR (Elenium):ti,ab,kw OR ("Chlordiazepoxide Hydrobromide"):ti,ab,kw OR (Hydrobromide, Chlordiazepoxide):ti,ab,kw OR ("Chlordiazepoxide Perchlorate"):ti,ab,kw OR (Perchlorate, Chlordiazepoxide):ti,ab,kw |
| #31 | #29 OR #30 |
| #32 | MeSH descriptor: [Amisulpride] explode all trees |
| #33 | ("4-Amino-N-((1-ethyl-2-pyrrolidinyl)methyl)-5-(ethylsulfonyl)-2-methoxybenzamide"):ti,ab,kw OR ("DAN 2163"):ti,ab,kw OR (DAN-2163):ti,ab,kw OR (Solian):ti,ab,kw OR (Sultopride):ti,ab,kw OR ("N-(Ethyl-1-pyrrolidinyl-2-methyl)methoxy-2-ethylsulfonyl-5-benzamide"):ti,ab,kw OR (LIN 1418):ti,ab,kw OR (LIN-1418):ti,ab,kw OR (Barnetil):ti,ab,kw OR ("Sultopride Hydrochloride"):ti,ab,kw |
| #34 | #32 OR #33 |
| #35 | MeSH descriptor: [Sulpiride] explode all trees |
| #36 | MeSH descriptor: [Olanzapine] explode all trees |
| #37 | ("2-Methyl-4-(4-methyl-1-piperazinyl)-10H-thieno(2,3-b)(1,5)benzodiazepine"):ti,ab,kw OR ("10H-thieno(2,3-b)(1,5)benzodiazepine, 2-Methyl-4-(4-methyl-1-piperazinyl)"):ti,ab,kw OR (LY-170052):ti,ab,kw OR ("52, LY-1700"):ti,ab,kw OR ("LY 170052"):ti,ab,kw OR ("52, LY 1700"):ti,ab,kw OR (LY170052):ti,ab,kw OR ("52, LY1700"):ti,ab,kw OR (Zyprexa):ti,ab,kw OR ("Zyprexa"):ti,ab,kw OR (Zolafren):ti,ab,kw OR ("Zolafren"):ti,ab,kw OR (LY 170053):ti,ab,kw OR ("53, LY 1700"):ti,ab,kw OR ("Olanzapine Pamoate"):ti,ab,kw OR ("Pamoate, Olanzapine"):ti,ab,kw |
| #38 | MeSH descriptor: [Amisulpride] explode all trees |
| #39 | MeSH descriptor: [Pregabalin] explode all trees |
| #40 | ("(S)-3-(aminomethyl)-5-methylhexanoic acid"):ti,ab,kw OR ("3-isobutyl GABA"):ti,ab,kw OR ("3 isobutyl GABA"):ti,ab,kw OR ("GABA, 3-isobutyl"):ti,ab,kw OR ("3-(aminomethyl)-5-methylhexanoic acid"):ti,ab,kw OR ("(R-)-3-isobutyl GABA"):ti,ab,kw OR ("(S+)-3-isobutyl GABA"):ti,ab,kw OR (Lyrica):ti,ab,kw OR ("CI 1008"):ti,ab,kw OR ("1008, CI"):ti,ab,kw OR (CI-1008):ti,ab,kw OR (CI1008Cimetidine):ti,ab,kw |
| #41 | #35 OR #36 OR #37 OR #38 OR #39 OR #40 |
| #42 | MeSH descriptor: [Chlormethiazole] explode all trees |
| #43 | (Clomethiazole):ti,ab,kw OR (Distraneurin):ti,ab,kw |
| #44 | #42 OR #43 |
| #45 | MeSH descriptor: [Ketamine] explode all trees |
| #46 | ("2-(2-Chlorophenyl)-2-(methylamino)cyclohexanone"):ti,ab,kw OR ("CI-581"):ti,ab,kw OR ("CI 581"):ti,ab,kw OR (CI581):ti,ab,kw OR (Ketalar):ti,ab,kw OR (Ketaset):ti,ab,kw OR (Ketanest):ti,ab,kw OR (Calipsol):ti,ab,kw OR (Kalipsol):ti,ab,kw OR (Calypsol):ti,ab,kw OR ("Ketamine Hydrochloride"):ti,ab,kw |
| #47 | #45 OR #46 |
| #48 | MeSH descriptor: [Baclofen] explode all trees |
| #49 | (Baclophen):ti,ab,kw OR (PCP-GABA):ti,ab,kw OR ("beta-(Aminomethyl)-4-chlorobenzenepropanoic Acid"):ti,ab,kw OR ("Chlorophenyl GABA"):ti,ab,kw OR ("GABA, Chlorophenyl"):ti,ab,kw OR ("beta-(p-Chlorophenyl)-gamma-aminobutyric Acid"):ti,ab,kw OR (Clofen):ti,ab,kw OR (Gen-Baclofen):ti,ab,kw OR ("Gen Baclofen"):ti,ab,kw OR (GenBaclofen):ti,ab,kw OR (Genpharm):ti,ab,kw OR (Apo-Baclofen):ti,ab,kw OR ("Apo Baclofen"):ti,ab,kw OR (ApoBaclofen):ti,ab,kw OR (Atrofen):ti,ab,kw OR (Baclofène-Irex):ti,ab,kw OR ("Baclofène Irex"):ti,ab,kw OR (BaclofèneIrex):ti,ab,kw OR ("Baclofen AWD"):ti,ab,kw OR ("AWD, Baclofen"):ti,ab,kw OR (Lebic):ti,ab,kw OR (Lioresal):ti,ab,kw OR (Liorésal):ti,ab,kw OR (Nu-Baclo):ti,ab,kw OR ("Nu Baclo"):ti,ab,kw OR (NuBaclo):ti,ab,kw OR (PMS-Baclofen):ti,ab,kw OR ("PMS Baclofen"):ti,ab,kw OR (PMSBaclofen):ti,ab,kw OR (Baclospas):ti,ab,kw OR ("Ba-34,647"):ti,ab,kw OR (Ba34,647):ti,ab,kw OR (Ba-34647):ti,ab,kw OR (Ba34647):ti,ab,kw OR ("CIBA-34,647-BA"):ti,ab,kw OR ("CIBA34,647BA"):ti,ab,kw |
| #50 | #48 OR #49 |
| #51 | MeSH descriptor: [Clonidine] explode all trees |
| #52 | (Klofenil):ti,ab,kw OR (Clofenil):ti,ab,kw OR (Chlophazolin):ti,ab,kw OR ("Clonidine Dihydrochloride"):ti,ab,kw OR ("Dihydrochloride, Clonidine"):ti,ab,kw OR ("Clonidine Hydrochloride"):ti,ab,kw OR ("Hydrochloride, Clonidine"):ti,ab,kw OR ("Clonidine Monohydrochloride"):ti,ab,kw OR ("Monohydrochloride, Clonidine"):ti,ab,kw OR ("Clonidine Monohydrobromide"):ti,ab,kw OR ("Monohydrobromide, Clonidine"):ti,ab,kw OR (ST-155):ti,ab,kw OR ("ST 155"):ti,ab,kw OR (ST155):ti,ab,kw OR (Gemiton):ti,ab,kw OR (Hemiton):ti,ab,kw OR (Isoglaucon):ti,ab,kw OR (Klofelin):ti,ab,kw OR (Clofelin):ti,ab,kw OR (Clopheline):ti,ab,kw OR (M-5041T):ti,ab,kw OR ("M 5041T"):ti,ab,kw OR (M5041T):ti,ab,kw OR (Catapres):ti,ab,kw OR (Catapresan):ti,ab,kw OR (Catapressan):ti,ab,kw OR (Dixarit):ti,ab,kw |
| #53 | #51 OR #52 |
| #54 | MeSH descriptor: [Anesthetics] explode all trees |
| #55 | ("Anesthetic Agents"):ti,ab,kw OR ("Agents, Anesthetic"):ti,ab,kw OR ("Anesthetic Drugs"):ti,ab,kw OR ("Drugs, Anesthetic"):ti,ab,kw OR (Anesthetic):ti,ab,kw OR ("Anesthetic Effect"):ti,ab,kw OR ("Effect, Anesthetic"):ti,ab,kw OR ("Anesthetic Effects"):ti,ab,kw OR ("Effects, Anesthetic"):ti,ab,kw |
| #56 | #54 OR #55 |
| #57 | MeSH descriptor: [Morphine] explode all trees |
| #58 | (Morphia):ti,ab,kw OR ("Morphine Chloride"):ti,ab,kw OR ("Chloride, Morphine"):ti,ab,kw OR ("Morphine Sulfate"):ti,ab,kw OR ("Sulfate, Morphine"):ti,ab,kw OR ("SDZ 202-250"):ti,ab,kw OR ("SDZ 202 250"):ti,ab,kw OR ("SDZ 202250"):ti,ab,kw OR ("SDZ202-250"):ti,ab,kw OR ("SDZ202 250"):ti,ab,kw OR (SDZ202250):ti,ab,kw OR ("Morphine Sulfate (2:1), Pentahydrate"):ti,ab,kw OR ("MS Contin"):ti,ab,kw OR ("Contin, MS"):ti,ab,kw OR ("Oramorph SR"):ti,ab,kw OR (Duramorph):ti,ab,kw OR ("Morphine Sulfate (2:1), Anhydrous"):ti,ab,kw |
| #59 | #57 OR #58 |
| #60 | MeSH descriptor: [Meperidine] explode all trees |
| #61 | (Pethidine):ti,ab,kw OR (Isonipecain):ti,ab,kw OR (Dolsin):ti,ab,kw OR (Dolosal):ti,ab,kw OR (Dolin):ti,ab,kw OR (“Operidine EPJ-I”):ti,ab,kw OR (“Operidine EPJ I”):ti,ab,kw OR (Dolantin):ti,ab,kw OR (Dolargan):ti,ab,kw OR (Lidol):ti,ab,kw OR (Lydol):ti,ab,kw OR (Demerol):ti,ab,kw OR (Dolcontral):ti,ab,kw |
| #62 | #60 OR #61 |
| #63 | MeSH descriptor: [Dexamethasone] explode all trees |
| #64 | (Methylfluorprednisolone):ti,ab,kw OR (Hexadecadrol):ti,ab,kw OR (Decameth):ti,ab,kw OR (Decaspray):ti,ab,kw OR (Dexasone):ti,ab,kw OR (Dexpak):ti,ab,kw OR (Maxidex):ti,ab,kw OR (Millicorten):ti,ab,kw OR (Oradexon):ti,ab,kw OR (Decaject):ti,ab,kw OR ("Decaject-L.A."):ti,ab,kw OR ("Decaject L.A."):ti,ab,kw OR (Hexadrol):ti,ab,kw |
| #65 | #63 OR #64 |
| #66 | MeSH descriptor: [Hypnotics and Sedatives] explode all trees |
| #67 | ("Sedatives and Hypnotics"):ti,ab,kw OR ("Sedative and Hypnotic"):ti,ab,kw OR ("Hypnotic and Sedative"):ti,ab,kw OR ("Hypnotic Effect"):ti,ab,kw OR ("Effect, Hypnotic"):ti,ab,kw OR ("Hypnotic Effects"):ti,ab,kw OR ("Effects, Hypnotic"):ti,ab,kw OR (Sedatives):ti,ab,kw OR (Hypnotics):ti,ab,kw OR (Hypnotic):ti,ab,kw OR ("Sedative Effect"):ti,ab,kw OR ("Effect, Sedative"):ti,ab,kw OR ("Sedative Effects"):ti,ab,kw OR ("Effects, Sedative"):ti,ab,kw OR ("Sleep Aids, Pharmaceutical"):ti,ab,kw OR ("Orexin Receptor Antagonists"):ti,ab,kw |
| #68 | #66 OR #67 |
| #69 | MeSH descriptor: [Dexmedetomidine] explode all trees |
| #70 | ("MPV-1440"):ti,ab,kw OR ("MPV 1440"):ti,ab,kw OR (MPV1440):ti,ab,kw OR (Precedex):ti,ab,kw OR (Dexdomitor):ti,ab,kw OR (Sedadex):ti,ab,kw OR (Sileo):ti,ab,kw OR (Cepedex):ti,ab,kw OR (Dexdor):ti,ab,kw OR ("Dexmedetomidine Hydrochloride"):ti,ab,kw OR ("Hydrochloride, Dexmedetomidine"):ti,ab,kw OR (Igalmi):ti,ab,kw |
| #71 | #69 OR #70 |
| #72 | MeSH descriptor: [Suvorexant] explode all trees |
| #73 | ("(4-(5-chloro-1,3-benzoxazol-2-yl)-7-methyl-1,4-diazepan-1-yl)(5-methyl-2-(2H-1,2,3-triazol-2-yl)phenyl)methanone"):ti,ab,kw OR ("methanone,((7R)-4-(5-chloro-2-benzoxazolyl)hexahydro-7-methyl-1H-1,4-diazepin-1-yl)(5-methyl-2-(2H-1,2,3-triazol-2-yl)phenyl)-MK 4305"):ti,ab,kw OR (MK4305):ti,ab,kw OR ("MK-4305"):ti,ab,kw OR (Belsomra):ti,ab,kw OR Ramelteon OR ("(S)-N-(2-(1,6,7,8-tetrahydro-2H-indeno-(5,4)furan-8-yl)ethyl)propionamide"):ti,ab,kw OR ("TAK-375"):ti,ab,kw OR (Rozerem):ti,ab,kw |
| #74 | #72 OR #73 |
| #75 | MeSH descriptor: [Benzodiazepines] explode all trees |
| #76 | ("Benzodiazepine Compounds"):ti,ab,kw OR ("Benzodiazepine"):ti,ab,kw |
| #77 | #75 OR #76 |
| #78 | MeSH descriptor: [Lorazepam] explode all trees |
| #79 | (Ativan):ti,ab,kw OR ("Orfidal Wyeth"):ti,ab,kw OR ("Wyeth, Orfidal"):ti,ab,kw OR (Témesta):ti,ab,kw OR (Tolid):ti,ab,kw OR (Donix):ti,ab,kw OR (Duralozam):ti,ab,kw OR (Durazolam):ti,ab,kw OR (Idalprem):ti,ab,kw OR (Laubeel):ti,ab,kw OR ("Lorazep Von Ct"):ti,ab,kw OR ("Von Ct, Lorazep"):ti,ab,kw OR ("Lorazepam Medical"):ti,ab,kw OR ("Medical, Lorazepam"):ti,ab,kw OR ("Lorazepam-Neuraxpharm"):ti,ab,kw OR ("Lorazepam Neuraxpharm"):ti,ab,kw OR ("Lorazepam-Ratiopharm"):ti,ab,kw OR ("Lorazepam Ratiopharm"):ti,ab,kw OR ("Novo-Lorazem"):ti,ab,kw OR ("Novo Lorazem"):ti,ab,kw OR ("Nu-Loraz"):ti,ab,kw OR ("Nu Loraz"):ti,ab,kw OR (Sedicepan):ti,ab,kw OR (Sinestron):ti,ab,kw OR ("WY-4036"):ti,ab,kw OR ("WY 4036"):ti,ab,kw OR (WY4036):ti,ab,kw OR ("Apo-Lorazepam"):ti,ab,kw OR ("Apo Lorazepam"):ti,ab,kw OR (Somagerol):ti,ab,kw OR (Temesta):ti,ab,kw |
| #80 | #78 OR #79 |
| #81 | MeSH descriptor: [Diazepam] explode all trees |
| #82 | ("7-Chloro-1,3-dihydro-1-methyl-5-phenyl-2H-1,4-benzodiazepin-2-one"):ti,ab,kw OR (Diazemuls):ti,ab,kw OR (Faustan):ti,ab,kw OR (Valium):ti,ab,kw OR (Seduxen):ti,ab,kw OR (Sibazon):ti,ab,kw OR (Stesolid):ti,ab,kw OR (Apaurin):ti,ab,kw OR (Relanium):ti,ab,kw |
| #83 | #81 OR #82 |
| #84 | MeSH descriptor: [Oxazepam] explode all trees |
| #85 | ("Adumbran"):ti,ab,kw OR ("Tazepam"):ti,ab,kw OR ("Serax"):ti,ab,kw |
| #86 | #84 OR #85 |
| #87 | MeSH descriptor: [Midazolam] explode all trees |
| #88 | ("Midazolam Maleate"):ti,ab,kw OR ("Maleate, Midazolam"):ti,ab,kw OR (Dormicum):ti,ab,kw OR (Versed):ti,ab,kw OR ("Midazolam Hydrochloride"):ti,ab,kw OR ("Hydrochloride, Midazolam"):ti,ab,kw OR ("Ro 21-3981"):ti,ab,kw OR ("Ro 21 3981"):ti,ab,kw OR ("Ro 213981"):ti,ab,kw |
| #89 | #87 OR #88 |
| #90 | MeSH descriptor: [Estazolam] explode all trees |
| #91 | ("Tasedan"):ti,ab,kw OR ("ProSom"):ti,ab,kw OR ("D-40TA"):ti,ab,kw OR ("D40TA"):ti,ab,kw OR ("Nuctalon"):ti,ab,kw |
| #92 | #90 OR #91 |
| #93 | MeSH descriptor: [Alprazolam] explode all trees |
| #94 | (Alprazolan):ti,ab,kw OR (Trankimazin):ti,ab,kw OR (“U-31,889”):ti,ab,kw OR ("U31,889"):ti,ab,kw OR (Xanax):ti,ab,kw OR (Alprox):ti,ab,kw OR (Esparon):ti,ab,kw OR (Cassadan):ti,ab,kw OR (D-65MT):ti,ab,kw OR (D65MT):ti,ab,kw OR (Kalma):ti,ab,kw OR ("Novo-Alprazol"):ti,ab,kw OR ("Novo Alprazol"):ti,ab,kw OR (Nu-Alpraz):ti,ab,kw OR ("Nu Alpraz"):ti,ab,kw OR (Ralozam):ti,ab,kw OR (Tafil):ti,ab,kw OR (Apo-Alpraz):ti,ab,kw OR ("Apo Alpraz"):ti,ab,kw |
| #95 | #93 OR #94 |
| #96 | MeSH descriptor: [Carbamazepine] explode all trees |
| #97 | ("Carbamazepine Acetate"):ti,ab,kw OR ("Carbamazepine Dihydrate"):ti,ab,kw OR ("Carbamazepine Hydrochloride"):ti,ab,kw OR ("Carbamazepine Anhydrous"):ti,ab,kw OR (Carbazepin):ti,ab,kw OR (Epitol):ti,ab,kw OR (Finlepsin):ti,ab,kw OR (Neurotol):ti,ab,kw OR (Tegretol):ti,ab,kw OR (Amizepine):ti,ab,kw OR ("Carbamazepine Phosphate"):ti,ab,kw |
| #98 | #96 OR #97 |
| #99 | MeSH descriptor: [Gabapentin] explode all trees |
| #100 | ("1-(Aminomethyl)cyclohexaneacetic Acid"):ti,ab,kw OR (Neurontin):ti,ab,kw OR ("Gabapentin Hexal"):ti,ab,kw OR (Convalis):ti,ab,kw OR ("Gabapentin-Ratiopharm"):ti,ab,kw OR ("Gabapentin Ratiopharm"):ti,ab,kw OR ("Novo-Gabapentin"):ti,ab,kw OR ("Novo Gabapentin"):ti,ab,kw OR (NovoGabapentin):ti,ab,kw OR (PMS-Gabapentin):ti,ab,kw OR (Apo-Gabapentin):ti,ab,kw OR ("Apo Gabapentin"):ti,ab,kw OR (ApoGabapentin):ti,ab,kw OR ("Gabapentin Stada"):ti,ab,kw |
| #101 | #99 OR #100 |
| #102 | MeSH descriptor: [Sevoflurane] explode all trees |
| #103 | ("Fluoromethyl-2,2,2-trifluoro-1-(trifluoromethyl)ethyl Ether"):ti,ab,kw OR ("Fluoromethyl Hexafluoroisopropyl Ether"):ti,ab,kw OR (Sevorane):ti,ab,kw OR (Ultane):ti,ab,kw OR ("BAX 3084"):ti,ab,kw |
| #104 | #102 OR #103 |
| #105 | MeSH descriptor: [Propofol] explode all trees |
| #106 | ("2,6-Diisopropylphenol"):ti,ab,kw OR ("2,6 Diisopropylphenol"):ti,ab,kw OR ("2,6-Bis(1-methylethyl)phenol"):ti,ab,kw OR (Disoprofol):ti,ab,kw OR (Diprivan):ti,ab,kw OR (Disoprivan):ti,ab,kw OR (Fresofol):ti,ab,kw OR ("ICI-35,868"):ti,ab,kw OR ("ICI 35,868"):ti,ab,kw OR ("ICI35,868"):ti,ab,kw OR ("ICI-35868"):ti,ab,kw OR ("ICI 35868"):ti,ab,kw OR ("ICI35868"):ti,ab,kw OR (Ivofol):ti,ab,kw OR ("Propofol Fresenius"):ti,ab,kw OR ("Propofol MCT"):ti,ab,kw OR ("Propofol Rovi"):ti,ab,kw OR (Propofol-Lipuro):ti,ab,kw OR (Recofol):ti,ab,kw OR (Aquafol):ti,ab,kw OR ("Propofol Abbott"):ti,ab,kw |
| #107 | #105 OR #106 |
| #108 | MeSH descriptor: [Cimetidine] explode all trees |
| #109 | ("N-Cyano-N'-methyl-N''-(2-(((5-methyl-1H-imidazol-4-yl)methyl)thio)ethyl)guanidine"):ti,ab,kw OR (Biomet):ti,ab,kw OR (Biomet400):ti,ab,kw OR ("Cimetidine Hydrochloride"):ti,ab,kw OR ("Hydrochloride, Cimetidine"):ti,ab,kw OR ("Cimetidine HCl"):ti,ab,kw OR ("HCl, Cimetidine"):ti,ab,kw OR (Histodil):ti,ab,kw OR ("SK and F-92334"):ti,ab,kw OR ("SK and F 92334"):ti,ab,kw OR ("SK and F92334"):ti,ab,kw OR (SKF-92334):ti,ab,kw OR ("SKF 92334"):ti,ab,kw OR (SKF92334):ti,ab,kw OR (Tagamet):ti,ab,kw OR (Altramet):ti,ab,kw OR (Eureceptor):ti,ab,kw |
| #110 | #108 OR #109 |
| #111 | MeSH descriptor: [Ranitidine] explode all trees |
| #112 | (Ranitidin):ti,ab,kw OR ("N(2-(((5-((Dimethylamino)methyl)-2-furanyl)methyl)thio)ethyl)-N'-methyl-2-nitro-1,1-ethenediamine"):ti,ab,kw OR (Biotidin):ti,ab,kw OR (Ranisen):ti,ab,kw OR (Sostril):ti,ab,kw OR (Zantac):ti,ab,kw OR (Zantic):ti,ab,kw OR ("AH-19065"):ti,ab,kw OR ("AH 19065"):ti,ab,kw OR (AH19065):ti,ab,kw OR ("Ranitidine Hydrochloride"):ti,ab,kw OR ("Hydrochloride, Ranitidine"):ti,ab,kw |
| #113 | #111 OR #112 |
| #114 | MeSH descriptor: [Randomized Controlled Trial] explode all trees |
| #115 | MeSH descriptor: [Controlled Clinical Trial] explode all trees |
| #116 | ("Randomized"):ti,ab,kw OR ("Randomised"):ti,ab,kw OR ("Placebo"):ti,ab,kw OR ("Controlled"):ti,ab,kw OR ("Random*"):ti,ab,kw OR ("Trial"):ti,ab,kw OR ("Blind* OR Mask*"):ti,ab,kw |
| #117 | #114 OR #115 OR #116 |
| #118 | #7 OR #10 OR #13 OR #16 OR #19 OR #22 OR #25 OR #28 OR #31 OR #34 OR #41 OR #44 OR #47 OR #50 OR #53 OR #56 OR #59 OR #62 OR #65 OR #68 OR #71 OR #74 OR #77 OR #80 OR #83 OR #86 OR #89 OR #92 OR #95 OR #98 OR #101 OR #104 OR #107 OR #110 OR #113 |
| #119 | #3 AND #118 AND #117 |

**C.EMbase**

| #1 | 'delirium'/exp OR 'delirium' |
| --- | --- |
| #2 | 'acute delirium' OR 'chronic delirium' OR 'delier' OR 'delire' OR 'deliria' OR 'delirious manifestation' OR 'delirious state' OR 'delirious syndrome' OR 'delirium acutum' OR 'delirium':ab,kw,ti |
| #3 | #1 OR #2 |
| #4 | 'melatonin'/exp OR 'melatonin' |
| #5 | '5 methoxy n acetyltryptamine' OR 'adaflex' OR 'apl 510' OR 'apl510' OR 'aritonin' OR 'bci 049' OR 'bci049' OR 'celton xl' OR 'ceyestaeusom' OR 'ceyesto' OR 'circadin' OR 'civasta' OR 'eusom' OR 'jan 13004' OR 'jan13004' OR 'ki 1001' OR 'ki1001' OR 'lestinora' OR 'mallozen' OR 'mecastrin' OR 'melabiorytm' OR 'melatal' OR 'melatan' OR 'melatol' OR 'melatonina' OR 'melatonine' OR 'melatonite' OR 'mellaras' OR 'mellozzan' OR 'melovine' OR 'mucomel' OR 'n acetyl 5 methoxytryptamine' OR 'noxarem' OR 'oniria (melatonin)' OR 'orlogin' OR 'regulin (melatonin)' OR 'sental' OR 'slenyto' OR 'sloremina' OR 'sp 13004' OR 'sp13004' OR 'syncrodin' OR 'waferest' OR 'melatonin':ab,kw,ti |
| #6 | #4 OR #5 |
| #7 | 'melatonin receptor'/exp OR 'melatonin receptor' |
| #8 | 'receptors, melatonin' OR 'melatonin receptor':ab,kw,ti |
| #9 | #7 OR #8 |
| #10 | #6 OR #9 |
| #11 | 'neuroleptic agent'/exp OR 'neuroleptic agent' |
| #12 | 'antipsychotic agent' OR 'antipsychotic agents' OR 'antipsychotic agents, butyrophenone' OR 'antipsychotic agents, phenothiazine' OR 'antipsychotic drug' OR 'antipsychotics' OR 'butyrophenone tranquilizers' OR 'classical antipsychotic' OR 'classical antipsychotic agent' OR 'classical antipsychotic drug' OR 'long acting neuroleptic' OR 'major tranquilizer' OR 'major tranquillizer' OR 'neuroleptic' OR 'neuroleptic drug' OR 'neurolepticum' OR 'phenothiazine tranquilizers' OR 'tranquilizer, major' OR 'tranquilizing agents, major' OR 'typical antipsychotic' OR 'typical antipsychotic agent' OR 'typical antipsychotic drug' OR 'typical neuroleptic' OR 'typical neuroleptic agent' OR 'typical neuroleptic drug' OR 'neuroleptic agent':ab,kw,ti |
| #13 | #11 OR #12 |
| #14 | 'haloperidol'/exp OR 'haloperidol' |
| #15 | '4 (para chlorophenyl) 1 [3 (para fluorobenzoyl) propyl] 4 piperidinol' OR '4 [4 (4 chlorophenyl) 4 hydroxy 1 piperidinyl] 1 (4 fluorophenyl) 1 butanone' OR '4 [4 (para chlorophenyl) 4 hydroxypiperidino] 4` fluorobutyrophenone' OR '4 p chlorophenyl 1 [3 (para fluorobenzoyl) propyl] 4 piperidinol' OR 'alased' OR 'aloperidin' OR 'aloperidine' OR 'apo-haloperidol' OR 'avant (drug)' OR 'binison' OR 'brotopon' OR 'celenase' OR 'cereen' OR 'cerenace' OR 'cizoren' OR 'depidol' OR 'dores' OR 'dozic' OR 'duraperidol' OR 'einalon s' OR 'fortunan' OR 'govotil' OR 'haldol' OR 'haldol solutab' OR 'halidol' OR 'halo-p' OR 'halojust' OR 'halomed' OR 'haloneural' OR 'haloper' OR 'haloperidol hydrochoride' OR 'haloperidol intensol' OR 'haloperidol lactate' OR 'haloperil' OR 'haloperin' OR 'haloperitol' OR 'halopidol' OR 'halopol' OR 'halosten' OR 'haricon' OR 'haridol-d' OR 'keselan' OR 'linton' OR 'lodomer-2' OR 'mcn jr 1625' OR 'mcn jr1625' OR 'mixidol' OR 'novoperidol' OR 'nsc 170973' OR 'nsc170973' OR 'peluces' OR 'perida' OR 'peridol' OR 'peridor' OR 'r 1625' OR 'r1625' OR 'selezyme' OR 'seranace' OR 'serenace' OR 'serenase' OR 'serenelfi' OR 'siegoperidol' OR 'sigaperidol' OR 'trancodol-10' OR 'trancodol-5' OR 'haloperidol':ab,kw,ti |
| #16 | #14 OR #15 |
| #17 | 'quetiapine'/exp OR 'quetiapine' |
| #18 | '11 [4 [2 (2 hydroxyethoxy) ethyl] 1 piperazinyl] dibenzo [b, f] [1, 4] thiazepine' OR '2 [2 (4 benzo [b] [1, 4] benzothiazepin 6 ylpiperazin 1 yl) ethoxy] ethanol' OR '2 [2 [4 (dibenzo [b, f] [1, 4] thiazepin 11 yl) 1 piperazinyl] ethoxy] ethanol' OR 'alaquet' OR 'alaquet xl' OR 'alcreno' OR 'alzen (drug)' OR 'alzen sr' OR 'atrolak' OR 'atrolak prolong' OR 'atrolak xl' OR 'aurinetine' OR 'biquelle' OR 'biquelle xl' OR 'bonogren' OR 'bonogren sr' OR 'brancico' OR 'brancico xl' OR 'centroqueen' OR 'derin' OR 'derin prolong' OR 'desiquet' OR 'etiagen' OR 'etiapin' OR 'fk 949' OR 'fk 949e' OR 'fk949' OR 'fk949e' OR 'geroquel' OR 'hedonin' OR 'hedonin prolong' OR 'hedonin xr' OR 'ici 204636' OR 'ici 204646' OR 'ici204636' OR 'ici204646' OR 'kefrenex' OR 'ketileppt' OR 'ketilept' OR 'ketilept prolong' OR 'ketilept retard' OR 'ketipinor' OR 'ketrel (quetiapine)' OR 'kvelux' OR 'kvelux sr' OR 'kventiax' OR 'kventiax prolong' OR 'kventiax sr' OR 'kwetaplex' OR 'loquen' OR 'loquen sr' OR 'matepil' OR 'mintreleq' OR 'mintreleq xl' OR 'norsic' OR 'notiabolfen' OR 'notiabolfen xl' OR 'pinexet' OR 'psicotric' OR 'psicotric prolong' OR 'psicotric retard' OR 'psyquet' OR 'psyquet xl' OR 'q-pin' OR 'qr 103' OR 'qr103' OR 'qudix' OR 'qudix prolong' OR 'quelapin' OR 'quental' OR 'quentiax' OR 'quentiax sr' OR 'quepin' OR 'questax' OR 'questax prolong' OR 'questax xr' OR 'quetamed' OR 'quetex' OR 'quetex xr' OR 'quethartic' OR 'quethartic xl' OR 'quetialan' OR 'quetialan xr' OR 'quetiapin' OR 'quetiapina' OR 'quetiapine fumarate' OR 'quetiapine xr' OR 'quettor' OR 'quettor xl' OR 'rocoz' OR 'rocoz prolong' OR 'seresano' OR 'seropia' OR 'seropia xr' OR 'seropin' OR 'seroquel' OR 'seroquel depot' OR 'seroquel ls' OR 'seroquel prolong' OR 'seroquel sr' OR 'seroquel xl' OR 'seroquel xr' OR 'serotiapin' OR 'serotiapin xr' OR 'setinin' OR 'socalm' OR 'sondate' OR 'sondate xl' OR 'symquel' OR 'symquel xr' OR 'tevaquel' OR 'tiaquel' OR 'tienapine' OR 'tomel' OR 'uniquet' OR 'xeroquel' OR 'xeroquel lp' OR 'zaluron' OR 'zaluron xl' OR 'zd 5077' OR 'zd5077' OR 'quetiapine':ab,kw,ti |
| #19 | #17 OR #18 |
| #20 | 'risperidone'/exp OR 'risperidone' |
| #21 | '3 [2 [4 (6 fluoro 1, 2 benzisoxazol 3 yl) 1 piperidinyl] ethyl] 6, 7, 8, 9 tetrahydro 2 methyl 4h pyrido [1, 2 a] pyrimidin 4 one' OR '3 [2 [4 (6 fluoro 1, 2 benzisoxazol 3 yl) 1 piperidyl] ethyl] 6, 7, 8, 9 tetrahydro 2 methyl 4h pyrido [1, 2 a] pyrimidin 4 one' OR '3 [2 [4 (6 fluoro 1, 2 benzisoxazol 3 yl) piperidin 1 yl] ethyl] 6, 7, 8, 9 tetrahydro 2 methyl 4h pyrido [1, 2 a] pyrimidin 4 one' OR '3 [2 [4 (6 fluoro 1, 2 benzisoxazol 3 yl) piperidino] ethyl] 6, 7, 8, 9 tetrahydro 2 methyl 4h pyrido [1, 2 a] pyrimidin 4 one' OR '[2 [4 (6 fluoro 1, 2 benzoxazol 3 yl) 1 piperidinyl] ethyl] 2 methyl 6, 7, 8, 9 tetrahydropyrido [1, 2 a] pyrimidin 4 one' OR '[2 [4 (6 fluoro 1, 2 benzoxazol 3 yl) 1 piperidyl] ethyl] 2 methyl 6, 7, 8, 9 tetrahydropyrido [1, 2 a] pyrimidin 4 one' OR '[2 [4 (6 fluoro 1, 2 benzoxazol 3 yl) piperidin 1 yl] ethyl] 2 methyl 6, 7, 8, 9 tetrahydropyrido [1, 2 a] pyrimidin 4 one' OR 'bb 0817' OR 'bb0817' OR 'belivon' OR 'consta' OR 'dlp 114' OR 'dlp114' OR 'doria' OR 'en 3342' OR 'en3342' OR 'eperon' OR 'jnj 410397' OR 'jnj410397' OR 'ly 03004' OR 'ly03004' OR 'lyn 005' OR 'lyn005' OR 'neripros' OR 'noprenia' OR 'okedi' OR 'orb 215' OR 'orb215' OR 'perseris' OR 'perseris kit' OR 'r 064766' OR 'r 64766' OR 'r064766' OR 'r64766' OR 'rbp 7000' OR 'rbp7000' OR 'rcn 3028' OR 'rcn3028' OR 'relday' OR 'riperidon' OR 'risolept' OR 'rispen' OR 'risperdal' OR 'risperdal consta' OR 'risperdal depot' OR 'risperdal flas' OR 'risperdal instasolv' OR 'risperdal m-tab' OR 'risperdal quicklet' OR 'risperdalconsta lp' OR 'risperdaloro' OR 'risperidon' OR 'risperisphere' OR 'rispid' OR 'rispolept' OR 'rispolept consta' OR 'rispolet' OR 'rispolux neo' OR 'rizodal' OR 'rykindo' OR 'sequinan' OR 'tv 46000' OR 'tv46000' OR 'uzedy' OR 'val 401' OR 'val401' OR 'zargus' OR 'zofredal' OR 'zx 003' OR 'zx003' OR 'risperidone':ab,kw,ti |
| #22 | #20 OR #21 |
| #23 | 'clozapine'/exp OR 'clozapine' |
| #24 | '3 chloro 6 (4 methyl 1 piperazinyl) 11h benzo [b] [1, 4] benzodiazepine' OR '3 chloro 6 (4 methylpiperazin 1 yl) 11h benzo [b] [1, 4] benzodiazepine' OR '8 chloro 11 (4 methyl 1 piperazinyl) 5h dibenzo [b, e] [1, 4] diazepine' OR '8 chloro 11 (4 methylpiperazin 1 yl) 5h dibenzo [b, e] [1, 4] diazepine' OR 'alemoxan' OR 'azaleptin' OR 'clopine' OR 'clopsine' OR 'clozapin' OR 'clozaril' OR 'denzapine' OR 'dorval' OR 'dozapine' OR 'elcrit' OR 'fazaclo' OR 'fazaclo odt' OR 'hf 1854' OR 'hf1854' OR 'lapenax' OR 'leponex' OR 'lozapin' OR 'lozapine' OR 'lx 100 129' OR 'lx100129' OR 'sizopin' OR 'versacloz' OR 'wander compound' OR 'zapen' OR 'zaponex' OR 'clozapine':ab,kw,ti |
| #25 | #23 OR #24 |
| #26 | 'ziprasidone'/exp OR 'ziprasidone' |
| #27 | '5 [2 [4 (1, 2 benzisothiazol 3 yl) 1 piperazinyl] ethyl] 6 chloro 1, 3 dihydro 2h indol 2 one' OR '5 [2 [4 (1, 2 benzothiazol 3 yl) 1 piperazinyl] ethyl] 6 chloro 1, 3 dihydroindol 2 one' OR '5 [2 [4 (1, 2 benzothiazol 3 yl) piperazin 1 yl] ethyl] 6 chloro 1, 3 dihydroindol 2 one' OR 'cp 88059' OR 'cp 88059 01' OR 'cp 88059 27' OR 'cp 88059-01' OR 'cp 88059-27' OR 'cp 8805901' OR 'cp 8805927' OR 'cp88059' OR 'cp88059 01' OR 'cp88059 27' OR 'cp88059-01' OR 'cp88059-27' OR 'cp8805901' OR 'cp8805927' OR 'empreval' OR 'geodon' OR 'me 2112' OR 'me2112' OR 'rq 00000003' OR 'rq00000003' OR 'zeldox' OR 'zeldrox' OR 'ziprasidone hydrochloride' OR 'ziprasidone hydrochloride monohydrate' OR 'ziprasidone mesilate' OR 'ziprasidone mesylate' OR 'ziprasidone methanesulfonate' OR 'zipsilan' OR 'zipsydon' OR 'zipwell' OR 'zypsila' OR 'zypsilan' OR 'ziprasidone':ab,kw,ti |
| #28 | #26 OR #27 |
| #29 | 'chlorpromazine'/exp OR 'chlorpromazine' |
| #30 | '2 chloro 10 (3 dimethylaminopropyl) phenothiazide' OR '2 chloro 10 (3 dimethylaminopropyl) phenothiazine' OR '2 chloro 10 [3 (dimethylamino) propyl] phenothiazine' OR '2 chloropromazine' OR '2601 a' OR '3 (2 chlorophenothiazin 10 yl) n, n dimethylpropan 1 amine' OR '4560 r p' OR 'adversive reaction, chlorpromazine' OR 'aminasin' OR 'aminasine' OR 'aminazin' OR 'aminazine' OR 'ampliactil' OR 'amplictil' OR 'ancholactil' OR 'aspersinal' OR 'bellacina' OR 'cepezet' OR 'chlomazine' OR 'chlopromazine' OR 'chlor pz' OR 'chloractil' OR 'chlorbromasin' OR 'chlordelazine' OR 'chlorderazin' OR 'chlormazine' OR 'chloropromazine' OR 'chlorpromanyl' OR 'chlorpromazin' OR 'chlorpromazine chloride' OR 'chlorpromazine hydrochloride' OR 'chlorpromazine hydrochloride intensol' OR 'chlorpromazine radical' OR 'chlorpromed' OR 'clonazine' OR 'clordelazin' OR 'clorpromaz' OR 'clorpromazine' OR 'clozine' OR 'contomin' OR 'duncan' OR 'elmarin' OR 'esmino' OR 'fenactil' OR 'hibanil' OR 'hibernal' OR 'hibernol' OR 'hl 3746' OR 'hl 5746' OR 'klorproman' OR 'klorpromazin' OR 'klorpromex' OR 'laractyl' OR 'largactil' OR 'largactil forte' OR 'largactyl' OR 'matcine' OR 'megaphen' OR 'megatil' OR 'ml 5746' OR 'n (3 dimethylaminopropyl) 3 chlorophenothiazine' OR 'neomazine' OR 'neurazine' OR 'novomazina' OR 'phenathyl' OR 'plegomazin' OR 'plegomazine' OR 'proma' OR 'promacid' OR 'promactil' OR 'promapar' OR 'promazil' OR 'promexin' OR 'propaphen' OR 'propaphenin' OR 'prozil' OR 'prozin' OR 'psychozine' OR 'psynor' OR 'rp 4560' OR 'sanopron' OR 'skf 2601 a' OR 'solidon' OR 'sonazine' OR 'taroctil' OR 'taroctyl' OR 'thor prom' OR 'thorazene' OR 'thorazine' OR 'torazina' OR 'vegetamin a' OR 'vegetamin b' OR 'winsumin' OR 'wintamine' OR 'wintermin' OR 'zuledin' OR 'chlorpromazine':ab,kw,ti |
| #31 | #29 OR #30 |
| #32 | 'chlordiazepoxide'/exp OR 'chlordiazepoxide' |
| #33 | '7 chloro 2 methylamino 5 phenyl 3h 1, 4 benzodiazepine 4 oxide' OR '7 chloro 2 methylamino 5 phenyl 3h 1, 4 benzodiazepine 4 oxide hydrochloride' OR '7 chloro n methyl 5 phenyl 3h 1, 4 benzodiazepin 2 amine 4 oxide' OR 'a poxide' OR 'a-poxide' OR 'ansiacal' OR 'apo-chlordiazepoxide' OR 'benpine' OR 'bent' OR 'benzodiapin' OR 'cebrum' OR 'cetabrium' OR 'chlordiazachel' OR 'chlordiazepoxid' OR 'chlordiazepoxide hcl' OR 'chlordiazepoxide hydrochloride' OR 'chlordiazepoxide salt' OR 'chlordiazepoxidum' OR 'chlordiazepoxyde' OR 'chlorodiazepoxide' OR 'clopoxid' OR 'clopoxide' OR 'contol' OR 'cozep' OR 'decacil' OR 'defobin' OR 'diazebrum' OR 'diazepina' OR 'disarim' OR 'dizepin' OR 'dizepine' OR 'dopoxide' OR 'droxol' OR 'eden psich' OR 'elenium' OR 'elenum' OR 'eposal' OR 'equibral' OR 'huberplex' OR 'kalmocaps' OR 'klopoxid' OR 'klorpo' OR 'labican' OR 'lentotran' OR 'liberth' OR 'librelease' OR 'libritabs' OR 'librium' OR 'lipoxide' OR 'lygen' OR 'mesural' OR 'metaminodiazepoxide' OR 'methaminodiazepoxide' OR 'mildmen' OR 'mitran' OR 'multum' OR 'murcil' OR 'napoto' OR 'napoton' OR 'napotone' OR 'neo-gnostorid' OR 'normide' OR 'nova-pam' OR 'novopoxide' OR 'novosed' OR 'o c m' OR 'o.c.m.' OR 'ocm 505' OR 'omnalio' OR 'paxium' OR 'psichial' OR 'psicofar' OR 'psicosan' OR 'psicosedin' OR 'psicoterina' OR 'radepur' OR 'raysedan' OR 'reliberan' OR 'reposans 10' OR 'retcol' OR 'risachief' OR 'risolid' OR 'ro 5 0690' OR 'seren' OR 'seren vita' OR 'servium' OR 'silibrin' OR 'sintesedan' OR 'sk lygen' OR 'sonimen' OR 'tensinyl' OR 'timosin' OR 'tropium (chlordiazepoxide)' OR 'viansin' OR 'viopsico' OR 'viopsicol' OR 'chlordiazepoxide':ab,kw,ti |
| #34 | #32 OR #33 |
| #35 | 'sulpiride'/exp OR 'sulpiride' |
| #36 | 'abilit' OR 'aiglonyl' OR 'aplacid' OR 'arminol' OR 'azugastan' OR 'betamaks' OR 'championyl' OR 'devodil' OR 'dobren' OR 'dogmacare' OR 'dogmatil' OR 'dogmatyl' OR 'dolmatil' OR 'eglonyl' OR 'ekilid' OR 'equilid' OR 'fk 880' OR 'fk880' OR 'isnamide' OR 'levair' OR 'levobren' OR 'levopraid' OR 'levosulpiride' OR 'meresa' OR 'miradol' OR 'n (1 ethylpyrrolidin 2 ylmethyl) 2 methoxy 5 sulfamoylbenzamide' OR 'neogama' OR 'sulfiride' OR 'sulpivert' OR 'sulpivol' OR 'sulpor' OR 'sulpyride' OR 'synedil' OR 'vipral' OR 'sulpiride':ab,kw,ti |
| #37 | #35 OR #36 |
| #38 | 'amisulpride'/exp OR 'amisulpride' |
| #39 | '4 amino n [ (1 ethyl 2 pyrrolidinyl) methyl] 5 (ethylsulfonyl) 2 methoxybenzamide' OR '4 amino n [ (1 ethyl 2 pyrrolidinyl) methyl] 5 (ethylsulfonyl) ortho anisamide' OR '4 amino n [ (1 ethyl 2 pyrrolidyl) methyl] 5 (ethylsulfonyl) 2 methoxybenzamide' OR '4 amino n [ (1 ethylpyrrolidin 2 yl) methyl] 5 (ethylsulfonyl) 2 methoxybenzamide' OR '4 azanyl n [ (1 ethyl 2 pyrrolidinyl) methyl] 5 ethylsulfonyl 2 methoxybenzamide' OR '4 azanyl n [ (1 ethyl 2 pyrrolidyl) methyl] 5 ethylsulfonyl 2 methoxybenzamide' OR '4 azanyl n [ (1 ethylpyrrolidin 2 yl) methyl] 5 ethylsulfonyl 2 methoxybenzamide' OR 'aktiprol' OR 'amilia (drug)' OR 'aminosultopride' OR 'amiprid' OR 'amisan' OR 'amissulprida' OR 'amisulgen' OR 'amisulid' OR 'amisulpiride' OR 'amisulpisan' OR 'amisulprid' OR 'amisulprida' OR 'amisulpride hydrochloride' OR 'amisulpridlich' OR 'amisulpridum' OR 'amitrex' OR 'amsulgen' OR 'apd 403' OR 'apd 421' OR 'apd403' OR 'apd421' OR 'aposuprid' OR 'aracalm' OR 'baremsis' OR 'barhemsys' OR 'dan 2163' OR 'dan2163' OR 'deniban' OR 'isofredil' OR 'n [ (1 ethyl 2 pyrrolidinyl) methyl] 2 methoxy 4 amino 5 (ethylsulfonyl) benzamide' OR 'n [ (1 ethyl 2 pyrrolidyl) methyl] 2 methoxy 4 amino 5 (ethylsulfonyl) benzamide' OR 'n [ (1 ethylpyrrolidin 2 yl) methyl] 2 methoxy 4 amino 5 (ethylsulfonyl) benzamide' OR 'nodasic' OR 'pridosil (drug)' OR 'sertol' OR 'socian' OR 'solian' OR 'sulamid' OR 'sulpitac' OR 'amisulpride':ab,kw,ti 2575  #38 'amisulpride'/exp OR 'amisulpride' |
| #40 | #38 OR #39 |
| #41 | 'sultopride'/exp OR 'sultopride' |
| #42 | 'barnetil' OR 'barnotil' OR 'ms 5024' OR 'ms5024' OR 'n [ (1 ethyl 2 pyrrolidinyl) methyl] 5 (ethylsulfonyl) 2 methoxybenzamide' OR 'sulfopride' OR 'sultopride':ab,kw,ti |
| #43 | #41 OR #42 |
| #44 | 'olanzapine'/exp OR 'olanzapine' |
| #45 | '2 methyl 10 (4 methyl 1 piperazinyl) 4h 3 thia 4, 9 diaza benzo [f] azulene' OR '2 methyl 10 (4 methyl 1 piperazyl) 4h 3 thia 4, 9 diaza benzo [f] azulene' OR '2 methyl 10 (4 methylpiperazin 1 yl) 4h 3 thia 4, 9 diaza benzo [f] azulene' OR '2 methyl 4 (4 methyl 1 piperazinyl) 10h benzo [b] thieno [2, 3 e] [1, 4] diazepine' OR '2 methyl 4 (4 methyl 1 piperazinyl) 10h thieno [2, 3 b] [1, 5] benzodiazepine' OR '2 methyl 4 (4 methyl 1 piperazyl) 10h benzo [b] thieno [2, 3 e] [1, 4] diazepine' OR '2 methyl 4 (4 methyl 1 piperazyl) 10h thieno [2, 3 b] [1, 5] benzodiazepine' OR '2 methyl 4 (4 methylpiperazin 1 yl) 10h benzo [b] thieno [2, 3 e] [1, 4] diazepine' OR '2 methyl 4 (4 methylpiperazin 1 yl) 10h thieno [2, 3 b] [1, 5] benzodiazepine' OR '5 methyl 8 (4 methyl 1 piperazinyl) 4 thia 2, 9 diazatricyclo [8.4.0.0 (3, 7)] tetradeca 1 (10), 3 (7), 5, 8, 11, 13 hexaene' OR '5 methyl 8 (4 methyl 1 piperazyl) 4 thia 2, 9 diazatricyclo [8.4.0.0 (3, 7)] tetradeca 1 (10), 3 (7), 5, 8, 11, 13 hexaene' OR '5 methyl 8 (4 methylpiperazin 1 yl) 4 thia 2, 9 diazatricyclo [8.4.0.0 (3, 7)] tetradeca 1 (10), 3 (7), 5, 8, 11, 13 hexaene' OR 'aedon' OR 'anzatric' OR 'anzorin' OR 'arenbil' OR 'arkolamyl' OR 'bloonis' OR 'caprilon (olanzapine)' OR 'dopin tab' OR 'egolanza' OR 'fredilan' OR 'inp 105' OR 'inp105' OR 'jolyon md' OR 'lanopin' OR 'lanzac' OR 'lapenza' OR 'lapozan' OR 'lazap' OR 'ly 170053' OR 'ly170053' OR 'malanxin' OR 'meltolan' OR 'midax' OR 'norpen oro' OR 'nykob' OR 'olace' OR 'oladay' OR 'olan' OR 'olandix' OR 'olandus' OR 'olanex' OR 'olanex instab' OR 'olansek' OR 'olanzalet' OR 'olanzalux' OR 'olanzapine mylan' OR 'olanzapine pamoate' OR 'olanzapine pamoate dihydrate' OR 'olanzapine pamoate monohydrate' OR 'olanzaran' OR 'olanzin' OR 'olapin' OR 'olapine' OR 'olazax' OR 'olazax disperzi' OR 'oleanz' OR 'olenxa' OR 'olexar' OR 'olmyzem' OR 'olpinat' OR 'oltal' OR 'olzap' OR 'olzapin' OR 'onza' OR 'ozapex' OR 'ozapin md' OR 'ozapram' OR 'psychozap' OR 'ranofren' OR 'relprevv' OR 'rolyprexa' OR 'stygapon' OR 'vaira' OR 'vaira-v' OR 'xoltiva' OR 'xyquila' OR 'zalasta' OR 'zalepin' OR 'zelta' OR 'ziora' OR 'zolafren' OR 'zolaxa' OR 'zolrix' OR 'zoxil' OR 'zylanza' OR 'zypadhera' OR 'zyprex' OR 'zyprexa' OR 'zyprexa adhera' OR 'zyprexa relprevv' OR 'zyprexa velotab' OR 'zyprexa zydis' OR 'zyprexav' OR 'olanzapine':ab,kw,ti |
| #46 | #44 OR #45 |
| #47 | #13 OR #16 OR #19 OR #22 OR #25 OR #28 OR #31 OR #34 OR #37 OR #40 OR #43 OR #46 |
| #48 | 'pregabalin'/exp OR 'pregabalin' |
| #49 | '3 aminomethyl 5 methylhexanoic acid' OR '3 isobutyl 4 aminobutanoic acid' OR '3 isobutyl 4 aminobutyric acid' OR '3 isobutyl gaba' OR '3 isobutylgaba' OR '4 amino 3 (2 methylpropyl) butanoic acid' OR '4 amino 3 isobutylbutanoic acid' OR '4 amino 3 isobutylbutyric acid' OR 'algerika' OR 'alivax (pregabalin)' OR 'alyse (drug)' OR 'alzain' OR 'andogablin' OR 'aprion (drug)' OR 'averopreg' OR 'axalid' OR 'axual' OR 'balfibro' OR 'bonqat' OR 'brieka' OR 'ci 1008' OR 'ci1008' OR 'clasica (drug)' OR 'convugabalin' OR 'dismedox' OR 'dolica' OR 'dragonor' OR 'ecubalin' OR 'epica' OR 'epiron' OR 'gaba-p' OR 'gabanext' OR 'gabarol' OR 'gabarol cr' OR 'gabica' OR 'gablin' OR 'gablovac' OR 'gabrika' OR 'gavin (pregabalin)' OR 'gialtyn' OR 'glonervya' OR 'helimon' OR 'hexgabalin' OR 'irenypathic' OR 'kabian (pregabalin)' OR 'kemirica' OR 'kineptia (pregabalin)' OR 'lecaent' OR 'lingabat' OR 'linprel' OR 'lyribastad' OR 'lyric' OR 'lyrica' OR 'lyrica cr' OR 'lyrineur' OR 'lyrolin' OR 'lyzalon' OR 'martesia (drug)' OR 'maxgalin' OR 'maxgalin er' OR 'mystika' OR 'nervalin' OR 'nervalin cr' OR 'neugaba' OR 'neugaba er' OR 'neuragabalin' OR 'neurega' OR 'neurica' OR 'neuristan' OR 'neurolin' OR 'neurovan' OR 'neurum' OR 'nuramed' OR 'nurogab' OR 'paden' OR 'pagadin' OR 'pagamax' OR 'painica' OR 'pd 144723' OR 'pd144723' OR 'pergadel' OR 'plenica' OR 'pragiola' OR 'prebanal' OR 'prebel' OR 'prebictal' OR 'prebien' OR 'prefaxil (pregabalin)' OR 'pregalin' OR 'pregalin sr' OR 'pregalodos' OR 'pregamid' OR 'pregan' OR 'pregastar' OR 'pregatrend' OR 'pregavalex' OR 'pregeb' OR 'pregeb od' OR 'pregobin' OR 'prelin' OR 'priga' OR 'provelyn' OR 'regapen' OR 'symra' OR 'vronogabic' OR 'xablin' OR 'xil' OR 'ynp 1807' OR 'ynp1807' OR 'pregabalin':ab,kw,ti 11360  #48 'pregabalin'/exp OR 'pregabalin' |
| #50 | 'clomethiazole'/exp OR 'clomethiazole' |
| #51 | '4 methyl 5 (beta chloroethyl) thiazole' OR '5 (2 chloroethyl) 4 methylthiazole' OR 'chlomethiazole' OR 'chlorethiazol' OR 'chlorethiazol ethane disulfonate' OR 'chlorethiazole' OR 'chlormethiazol' OR 'chlormethiazole' OR 'clomethiazole edisylate' OR 'clomethiazole ethanedisulfonate' OR 'clometiazole' OR 'distraneurin' OR 'distraneurine' OR 'emineurina' OR 'hemineurin' OR 'hemineurine' OR 'heminevrin' OR 'heminevrine' OR 'hemithiamine' OR 's c t z' OR 'zendra' OR 'clomethiazole':ab,kw,ti |
| #52 | #48 OR #49 |
| #53 | #50 OR #51 |
| #54 | 'ketamine'/exp OR 'ketamine' |
| #55 | '2 (2 chlorophenyl) 2 (methylamino) cyclohexanone' OR '2 (2 chlorophenyl) 2 (methylamino) cyclohexanone hydrochloride' OR '2 (2 chlorophenyl) 2 methylaminocyclohexanone' OR '2 (methylamino) 2 (2 chlorophenyl) cyclohexanone' OR '2 (ortho chlorophenyl) 2 (methylamino) cyclohexanone' OR '2 (ortho chlorophenyl) 2 methylaminocyclohexanone' OR '2 (ortho chlorophenyl) 2 methylaminocyclohexanone hydrochloride' OR '2 methylamino 2 (2 chlorophenyl) cyclohexanone' OR '2 ortho chlorophenyl 2 methylaminocyclohexanone' OR 'anesject' OR 'calipsol' OR 'calypsol' OR 'ci 581' OR 'ci581' OR 'cl 369' OR 'cl369' OR 'cn 52, 372 2' OR 'cn 52372 2' OR 'cn 523722' OR 'cn52, 372 2' OR 'cn52372 2' OR 'cn523722' OR 'ereska' OR 'imalgene' OR 'kalipsol' OR 'katamine' OR 'keta-hameln' OR 'ketaject' OR 'ketalar' OR 'ketalin' OR 'ketamax' OR 'ketamine hcl' OR 'ketamine hydrochloride' OR 'ketaminol vet' OR 'ketanest' OR 'ketased' OR 'ketaset' OR 'ketaved' OR 'ketavet' OR 'ketmin' OR 'ketoject' OR 'ketolar' OR 'narkamon' OR 'narketan' OR 'pmi 100' OR 'pmi 150' OR 'pmi100' OR 'pmi150' OR 'sls 002' OR 'sls002' OR 'soon-soon' OR 'tekam' OR 'tur 002' OR 'tur002' OR 'velonarcon' OR 'vetalar' OR 'ketamine':ab,kw,ti |
| #56 | 'baclofen'/exp OR 'baclofen' |
| #57 | '3 (4 chlorophenyl) 4 aminobutanoic acid' OR '3 (4 chlorophenyl) 4 aminobutyric acid' OR '3 (para chlorophenyl) 4 aminobutanoic acid' OR '3 (para chlorophenyl) 4 aminobutyric acid' OR '4 amino 3 (4 chlorophenyl) butanoic acid' OR '4 amino 3 (4 chlorophenyl) butyric acid' OR 'all 1401' OR 'all1401' OR 'alpha-baclofen' OR 'ba 34 647' OR 'ba 34647' OR 'ba34 647' OR 'ba34647' OR 'backen' OR 'baclan' OR 'baclapone' OR 'baclaxiro' OR 'baclo' OR 'baclocur' OR 'baclofene' OR 'baclon' OR 'baclopar' OR 'baclophen' OR 'baclosal' OR 'bacofen' OR 'bacron' OR 'bafen' OR 'baklofen' OR 'baropan' OR 'beta (4 chlorophenyl) 4 aminobutyric acid' OR 'beta (4 chlorophenyl) gamma aminobutyric acid' OR 'beta (aminomethyl) 4 chlorobenzenepropanoic acid' OR 'beta (aminomethyl) 4 chlorocinnamic acid' OR 'beta (aminomethyl) 4 chlorohydrocinnamic acid' OR 'beta (aminomethyl) para chlorocinnamic acid' OR 'beta (para chlorophenyl) 4 aminobutyric acid' OR 'beta 4 chlorophenyl 4 aminobutanoic acid' OR 'beta 4 chlorophenyl gamma aminobutyric acid' OR 'beta amino methyl chlorohydrocinnamic acid' OR 'beta aminomethyl para chlorohydrocinnamic acid' OR 'beta para chlorophenyl gamma aminobutyric acid' OR 'bigafen' OR 'ciba 34647 ba' OR 'ciba 34647ba' OR 'ciba34647ba' OR 'colmifen' OR 'curofen' OR 'dextro baclofen' OR 'espast' OR 'fleqsuvy' OR 'gabalon' OR 'gablofen' OR 'intralcal' OR 'ipx 056' OR 'ipx056' OR 'kemstro' OR 'lebic' OR 'levo baclofen' OR 'liofen' OR 'lionova' OR 'lioresal' OR 'lioresal intrathecal' OR 'lioresyl' OR 'liotec' OR 'lyflex' OR 'lyvispah' OR 'miorel (baclofen)' OR 'neteka' OR 'onelaxant-r' OR 'ozobax' OR 'ozobax ds' OR 'pacifen' OR 'spacyr' OR 'spinax' OR 'stelax' OR 'vioridon' OR 'baclofen':ab,kw,ti |
| #58 | #56 OR #57 |
| #59 | #54 OR #55 |
| #60 | 'clonidine'/exp OR 'clonidine' |
| #61 | '(2, 6 dichlorophenylamine) 2 imidazoline hydrochloride' OR '2 (2, 6 dichloranilino) 2 imidazoline' OR '2 (2, 6 dichloroanilino) 2 imidazoline' OR '2 (2, 6 dichloroanilino) 2 imidazoline hydrochloride' OR '2 (2, 6 dichloroanilino) imidazoline' OR '2 (2, 6 dichlorophenyl 1 amino) 2 imidazoline' OR '2 (2, 6 dichlorophenylamino) 2 imidazoline' OR '2 (2, 6 dichlorophenylamino) 2 imidazoline hydrochloride' OR '2 (2, 6 dichlorophenylamino) imidazoline' OR '2 [2, 6 dichlorphenylamino] 2 imidazoline hydrochloride' OR '2, 6 dichlorophenylamine 2 imidazoline hydrochloride' OR 'adesipress tts 2' OR 'adesipress tts2' OR 'arkamin' OR 'atensina' OR 'calotep' OR 'caprysin' OR 'catapres' OR 'catapres tts' OR 'catapres-tts-1' OR 'catapres-tts-2' OR 'catapres-tts-3' OR 'catapresan' OR 'catapresan 100' OR 'catapresan depot' OR 'catapresan tts' OR 'catapressan' OR 'catapressan perlonguettes' OR 'catapressant' OR 'catasan' OR 'chlofazolin' OR 'chlophazolin' OR 'chlophelin' OR 'clinidine' OR 'clofelin' OR 'clofeline' OR 'clomidine' OR 'clondine' OR 'clonicel' OR 'clonidin' OR 'clonidine chlorhydrate' OR 'clonidine hydrochloride' OR 'clonidine tts-1' OR 'clonidine tts-2' OR 'clonidine tts-3' OR 'clonipresan' OR 'clonistada' OR 'clonistada retard' OR 'clonnirit' OR 'clophelin' OR 'clopheline' OR 'daipres' OR 'dcai' OR 'dichlorophenylaminoimidazoline' OR 'dichlorophenylaminoimidazoline hydrochloride' OR 'dixarit' OR 'duraclon' OR 'haemiton' OR 'hemiton' OR 'huma-clonidine' OR 'hypodine' OR 'isoglaucon' OR 'jenloga' OR 'kapvay' OR 'm 5041t' OR 'm5041t' OR 'melzin' OR 'nexiclon' OR 'nexiclon xr' OR 'normopresan' OR 'normopresin' OR 'paracefan' OR 'st 155' OR 'st155' OR 'sulmidine' OR 'taitecin' OR 'tenso timelets' OR 'clonidine':ab,kw,ti |
| #62 | #60 OR #61 |
| #63 | #52 OR #53 OR #58 OR #59 OR #62 |
| #64 | 'anesthetic agent'/exp OR 'anesthetic agent' |
| #65 | 'anaesthetic' OR 'anaesthetic agent' OR 'anaesthetic drug' OR 'anaesthetics' OR 'anaesthetics, combined' OR 'anaesthetics, dissociative' OR 'anaesthetics, general' OR 'anesthetic' OR 'anesthetic drug' OR 'anesthetics' OR 'anesthetics, combined' OR 'anesthetics, dissociative' OR 'anesthetics, general' OR 'general anaesthetic' OR 'general anaesthetic agent' OR 'general anesthetic' OR 'general anesthetic agent' OR 'preanaesthetics' OR 'preanesthetics' OR 'anesthetic agent':ab,kw,ti |
| #66 | 'morphine'/exp OR 'morphine' |
| #67 | '(5alpha, 6alpha) 17 methyl 7, 8 didehydro 4, 5 epoxymorphinan 3, 6 diol' OR '12 methyl 4a, 5, 7a, 8 tetrahydro 9h 9, 9c iminoethanophenanthro [4, 5 bcd] furan 3, 5 diol' OR '2 [4 [2, 4 diamino 6 pteridinylmethyl (methyl) amino] phenylcarboxamido] pentanedioic acid' OR '2, 3, 4, 4a, 7, 7a hexahydro 3 methyl 1h 4, 12 methano [1] benzofuro [3, 2 e] isoquinoline 7, 9 diol' OR '2, 3, 4, 4a, 7, 7a hexahydro 3 methyl 4, 12 methano [1] benzofuro [3, 2 e] isoquinoline 7, 9 diol' OR '2, 4, 4a, 7, 7a, 13 hexahydro 3 methyl 1h 4, 12 methanobenzofuro [3, 2 e] isoquinoline 7, 9 diol' OR '2, 4, 4a, 7, 7a, 13 hexahydro 3 methyl 4, 12 methanobenzofuro [3, 2 e] isoquinoline 7, 9 diol' OR '3 methyl 2, 3, 4, 4a, 7, 7a hexahydro 1h 4, 12 methano [1] benzofuro [3, 2 e] isoquinoline 7, 9 diol' OR '3 methyl 2, 3, 4, 4a, 7, 7a hexahydro 4, 12 methano [1] benzofuro [3, 2 e] isoquinoline 7, 9 diol' OR '3 methyl 2, 4, 4a, 7, 7a, 13 hexahydro 1h 4, 12 methanobenzofuro [3, 2 e] isoquinoline 7, 9 diol' OR '3 methyl 2, 4, 4a, 7, 7a, 13 hexahydro 4, 12 methanobenzofuro [3, 2 e] isoquinoline 7, 9 diol' OR '3, 6 dihydroxy n methyl 4, 5 epoxy 7 morphinene' OR '4 methyl 12 oxa 4 azapentacyclo [9.6.1.0 (1, 13) .0 (5, 17) .0 (7, 18)] octadeca 7 (18), 8, 10, 15 tetraene 10, 14 diol' OR '4, 5 epoxy n methyl 7 morphinen 3, 6 diol' OR '4, 5alpha epoxy 17 methyl 7 morphinen 3, 6alpha diol' OR '4a, 5, 7a, 8 tetrahydro 12 methyl 4a, 5, 7a, 8 tetrahydro 9, 9c iminoethanophenanthro [4, 5 bcd] furan 3, 5 diol' OR '4a, 5, 7a, 8 tetrahydro 12 methyl 9, 9c iminoethanophenanthro [4, 5 bcd] furan 3, 5 diol' OR '4a, 5, 7a, 8 tetrahydro 12 methyl 9h 9, 9c iminoethanophenanthro [4, 5 bcd] furan 3, 5 diol' OR '7, 8 didehydro 4, 5 epoxy 17 methylmorphinan 3, 6 diol' OR 'anpec' OR 'cis morphine' OR 'duromorph' OR 'epimorph' OR 'maracex' OR 'microcrystalline morphine suspension' OR 'miro' OR 'morfin' OR 'morfine' OR 'morphia' OR 'morphin' OR 'morphine alkaloid' OR 'morphine chlorhydrate' OR 'morphine chloride' OR 'morphine hydrochloride' OR 'morphine suspension' OR 'morphinium' OR 'morphium' OR 'opso' OR 'sendolor' OR 'skenan' OR 'trans morphine' OR 'morphine':ab,kw,ti |
| #68 | 'pethidine'/exp OR 'pethidine' |
| #69 | '1 methyl 4 phenyl 4 carbethoxypiperidine' OR '1 methyl 4 phenylisonipecotic acid ethyl ester' OR '4 ethoxycarbonyl 1 methyl 4 phenylpiperidine' OR 'algil' OR 'alodan' OR 'alodan gerot' OR 'centralgin' OR 'centralgine' OR 'cluyer' OR 'demero' OR 'demerol' OR 'demerol hcl' OR 'demerol hydrochloride' OR 'dispadol' OR 'dolanquifa' OR 'dolantal' OR 'dolantin' OR 'dolantina' OR 'dolantine' OR 'dolargan' OR 'dolcontral' OR 'dolenal' OR 'dolestin' OR 'dolestine' OR 'dolin' OR 'dolocontral' OR 'doloneurin' OR 'doloneurotrat' OR 'dolor' OR 'dolosa' OR 'dolosal' OR 'dolosan' OR 'dolsin' OR 'dolvanol' OR 'endolate' OR 'ethyl 1 methyl 4 phenylpiperidine 4 carboxylate' OR 'isonipecaine' OR 'l pethidine' OR 'lidol' OR 'lydol' OR 'mefedina' OR 'mepadin' OR 'meperdol' OR 'meperiden' OR 'meperidin' OR 'meperidine' OR 'meperidine hydrobromide' OR 'meperidine hydrochloride' OR 'meperidine hydrochloride preservative free' OR 'meperidol' OR 'meperitab' OR 'mephedine' OR 'mepiridine' OR 'methylphenylcarbethoxypiperidine hydrochloride' OR 'mialgin' OR 'n methyl 4 phenyl 4 carbethoxypiperidine hydrochloride' OR 'neomochin' OR 'opistan' OR 'pantalgin' OR 'petadin' OR 'petantin' OR 'petantina' OR 'pethanol' OR 'pethedine' OR 'pethidin' OR 'pethidine hydrobromide' OR 'pethidine hydrochloride' OR 'pethidine injection' OR 'pethidine roche' OR 'pethidine tablet' OR 'petidin' OR 'phetidine' OR 'piridosal' OR 'sauteralgyl' OR 'simesalgina' OR 'supplosal' OR 'synlaudine' OR 'pethidine':ab,kw,ti |
| #70 | 'dexamethasone'/exp OR 'dexamethasone' |
| #71 | '1 fluoro 14, 17 dihydroxy 14 (2 hydroxyacetyl) 2, 13, 15 trimethyltetracyclo [8.7.0.0 (2, 7) .0 (11, 15)] heptadeca 3, 6 dien 5 one' OR '16alpha methyl 9alpha fluoroprednisolone' OR '9 alpha fluoro 16 alpha methyl delta corticosterone' OR '9 fluoro 11, 17 dihydroxy 17 (2 hydroxyacetyl) 10, 13, 16 trimethyl 6, 7, 8, 11, 12, 14, 15, 16 octahydrocyclopenta [a] phenanthren 3 one' OR '9alpha fluoro 11beta, 17alpha, 21 trihydroxy 16alpha methyl 1, 4 pregnadiene 3, 20 dione' OR '9alpha fluoro 11beta, 17alpha, 21 trihydroxy 16alpha methylpregna 1, 4 diene 3, 20 dione' OR '9alpha fluoro 16alpha methyl delta corticosterone' OR 'adrecort' OR 'adrenocot' OR 'aeroseb dex' OR 'aeroseb-d' OR 'aeroseb-dex' OR 'aflucoson' OR 'aflucosone' OR 'alfalyl' OR 'anaflogistico' OR 'anaflogistico novobios' OR 'aphtasolon' OR 'arcodexan' OR 'arcodexane' OR 'artrosone' OR 'auxiron' OR 'azium' OR 'baycadron' OR 'bidexol' OR 'bisu ds' OR 'calonat' OR 'cebedex' OR 'cetadexon' OR 'colofoam' OR 'corsona' OR 'corsone' OR 'cortastat' OR 'cortastat 10' OR 'cortastat la' OR 'cortidex' OR 'cortidexason' OR 'cortidrona' OR 'cortidrone' OR 'cortisumman' OR 'dacortina fuerte' OR 'dacortine fuerte' OR 'dalalone' OR 'dalalone d.p.' OR 'dalalone l.a.' OR 'danasone' OR 'de-sone la' OR 'decacortin' OR 'decadeltosona' OR 'decadeltosone' OR 'decaderm' OR 'decadion' OR 'decadran' OR 'decadron' OR 'decadron 5-12 pak' OR 'decadron la' OR 'decadronal' OR 'decadrone' OR 'decaesadril' OR 'decagel' OR 'decaject' OR 'decalix' OR 'decamethasone' OR 'decasone' OR 'decaspray' OR 'decasterolone' OR 'decdan' OR 'decilone' OR 'decilone forte' OR 'decofluor' OR 'dectancyl' OR 'dekacort' OR 'delladec' OR 'deltafluoren' OR 'deltafluorene' OR 'dergramin' OR 'deronil' OR 'desacort' OR 'desacortone' OR 'desadrene' OR 'desalark' OR 'desameton' OR 'desametone' OR 'desigdron' OR 'dexa cortisyl' OR 'dexa dabrosan' OR 'dexa korti' OR 'dexa scherosan' OR 'dexa scherozon' OR 'dexa scherozone' OR 'dexa-p' OR 'dexacen 4' OR 'dexacen-4' OR 'dexachel' OR 'dexacollyre' OR 'dexacort' OR 'dexacortal' OR 'dexacorten' OR 'dexacortin' OR 'dexacortisyl' OR 'dexadabroson' OR 'dexadecadrol' OR 'dexadrol' OR 'dexafarma' OR 'dexagel' OR 'dexagen' OR 'dexahelvacort' OR 'dexakorti' OR 'dexalien' OR 'dexaliquid geriasan' OR 'dexalocal' OR 'dexame' OR 'dexamecortin' OR 'dexameson' OR 'dexamesone' OR 'dexametason' OR 'dexametasone' OR 'dexameth' OR 'dexamethason' OR 'dexamethasone alcohol' OR 'dexamethasone intensol' OR 'dexamethazon' OR 'dexamethazone' OR 'dexamethonium' OR 'dexamonozon' OR 'dexan' OR 'dexane' OR 'dexano' OR 'dexapolcort' OR 'dexapot' OR 'dexascheroson' OR 'dexascherozon' OR 'dexascherozone' OR 'dexason' OR 'dexasone' OR 'dexasone la' OR 'dexasone s' OR 'dexinoral' OR 'dexionil' OR 'dexmethsone' OR 'dexona' OR 'dexone' OR 'dexone 0.5' OR 'dexone 0.75' OR 'dexone 1.5' OR 'dexone 4' OR 'dexpak taperpak' OR 'dextelan' OR 'dextenza' OR 'dextrasone' OR 'dexycu' OR 'dexycu kit' OR 'dezone' OR 'dibasona' OR 'doxamethasone' OR 'esacortene' OR 'ex s1' OR 'exadion' OR 'exadione' OR 'firmalone' OR 'fluormethyl prednisolone' OR 'fluormethylprednisolon' OR 'fluormethylprednisolone' OR 'fluormone' OR 'fluorocort' OR 'fluorodelta' OR 'fluoromethylprednisolone' OR 'fortecortin' OR 'gammacorten' OR 'gammacortene' OR 'glensoludex' OR 'grosodexon' OR 'grosodexone' OR 'hemady' OR 'hexadecadiol' OR 'hexadecadrol' OR 'hexadiol' OR 'hexadrol' OR 'isicort' OR 'isnacort' OR 'isopto dex' OR 'isopto maxidex' OR 'isopto-dex' OR 'isopto-maxidex' OR 'isoptodex' OR 'isoptomaxidex' OR 'isv 305' OR 'isv305' OR 'lokalison f' OR 'loverine' OR 'luxazone' OR 'marvidione' OR 'maxidex' OR 'mediamethasone' OR 'megacortin' OR 'mephameson' OR 'mephamesone' OR 'metasolon' OR 'metasolone' OR 'methazon ion' OR 'methazone ion' OR 'methazonion' OR 'methazonione' OR 'metisone lafi' OR 'mexasone' OR 'millicorten' OR 'millicortenol' OR 'mk 125' OR 'mk125' OR 'mymethasone' OR 'neoforderx' OR 'neofordex' OR 'nisomethasona' OR 'nodexon' OR 'novocort' OR 'nsc 34521' OR 'nsc34521' OR 'oftan-dexa' OR 'opticorten' OR 'opticortinol' OR 'oradexan' OR 'oradexon' OR 'oradexone' OR 'orgadrone' OR 'oto 104' OR 'oto104' OR 'ozurdex' OR 'perazone' OR 'pidexon' OR 'policort' OR 'posurdex' OR 'predni f tablinen' OR 'predni-f' OR 'prednisolone f' OR 'prodexona' OR 'prodexone' OR 'sanamethasone' OR 'santenson' OR 'santeson' OR 'sawasone' OR 'sk 0503' OR 'sk0503' OR 'solurex' OR 'solurex la' OR 'spoloven' OR 'spt 2101' OR 'spt2101' OR 'sterasone' OR 'thilodexine' OR 'triamcimetil' OR 'vexamet' OR 'visumetazone' OR 'visumethazone' OR 'zeqmelit' OR 'dexamethasone':ab,kw,ti |
| #72 | #70 OR #71 |
| #73 | #68 OR #69 |
| #74 | #66 OR #67 |
| #75 | #64 OR #65 |
| #76 | #72 OR #73 OR #74 OR #75 |
| #77 | 'sedative agent'/exp OR 'sedative agent' |
| #78 | 'neurosedative drug' OR 'sedative' OR 'sedative drug' OR 'sedative mixture' OR 'sedatives, barbiturate' OR 'sedatives, nonbarbiturate' OR 'soporific agent' OR 'sedative agent':ab,kw,ti |
| #79 | 'dexmedetomidine'/exp OR 'dexmedetomidine' |
| #80 | 'bxcl 501' OR 'bxcl501' OR 'cepedex' OR 'da 9501' OR 'da9501' OR 'delos' OR 'dexamedetomidine' OR 'dexdomitor' OR 'dexdor' OR 'dexmedetomidine hydrochloride' OR 'igalmi' OR 'mpv 1440' OR 'mpv1440' OR 'precedex' OR 'primadex' OR 'sedadex' OR 'sileo' OR 'tpu 006' OR 'tpu006' OR 'dexmedetomidine':ab,kw,ti |
| #81 | #79 OR #80 |
| #82 | #77 OR #78 |
| #83 | 'suvorexant'/exp OR 'suvorexant' |
| #84 | '[4 (5 chloro 1, 3 benzoxazol 2 yl) 7 methyl 1, 4 diazepan 1 yl] [5 methyl 2 (2h 1, 2, 3 triazol 2 yl) phenyl] methanone' OR '[4 (5 chloro 2 benzoxazolyl) hexahydro 7 methyl 1h 1, 4 diazepin 1 yl] [5 methyl 2 (2h 1, 2, 3 triazol 2 yl) phenyl] methanone' OR '[4 (5 chlorobenzoxazol 2 yl) 7 methylhexahydro 1h 1, 4 diazepin 1 yl] [5 methyl 2 (2h 1, 2, 3 triazol 2 yl) phenyl] methanone' OR 'belsomra' OR 'mk 4305' OR 'mk4305' OR 'suvarexant' OR 'suvorexant':ab,kw,ti |
| #85 | #83 OR #84 |
| #86 | 'ramelteon'/exp OR 'ramelteon' |
| #87 | 'n [2 (1, 6, 7, 8 tetrahydro 2h indeno [5, 4 b] furan 8 yl) ethyl] propanamide' OR 'n [2 (2, 6, 7, 8 tetrahydro 1h indeno [5, 4 b] furan 8 yl) ethyl] propionamide' OR 'rozerem' OR 'tak 375' OR 'tak375' OR 'ramelteon':ab,kw,ti |
| #88 | #86 OR #87 |
| #89 | #81 OR #82 OR #85 OR #88 |
| #90 | 'lorazepam'/exp OR 'lorazepam' |
| #91 | '7 chloro 5 (2 chlorophenyl) 1, 3 dihydro 3 hydroxy 2h 1, 4 benzodiazepin 2 one' OR '7 chloro 5 (2 chlorophenyl) 3 hydroxy 1, 3 dihydro 1, 4 benzodiazepin 2 one' OR '7 chloro 5 (o chlorophenyl) 1, 3 dihydro 3 hydroxy 2h 1, 4 benzodiazepin 2 one' OR '7 chloro 5 (ortho chlorophenyl) 1, 3 dihydro 3 hydroxy 1, 4 benzdiazepin 2 one' OR '7 chloro 5 (ortho chlorophenyl) 1, 3 dihydro 3 hydroxy 2h 1, 4 benzodiazepin 2 one' OR 'almazine' OR 'alzapam' OR 'anxiedin' OR 'anxira' OR 'anzepam' OR 'aplacasse' OR 'apo-lorazepam' OR 'aripax' OR 'ativan' OR 'azurogen' OR 'bonatranquan' OR 'donix' OR 'duralozam' OR 'edg 004' OR 'edg004' OR 'efasedan' OR 'emotival' OR 'kalmalin' OR 'kendol' OR 'larpose' OR 'laubeel' OR 'lonza' OR 'lopam' OR 'lorabenz' OR 'loram' OR 'loranase' OR 'loranaze' OR 'lorans' OR 'lorapam' OR 'loravan' OR 'lorax' OR 'loraz' OR 'lorazene' OR 'lorazep' OR 'lorazepam intensol' OR 'lorazepam preservative free' OR 'lorazin' OR 'lorazon' OR 'loreev' OR 'loreev xr' OR 'lorenin' OR 'loridem' OR 'lorivan' OR 'lorsedal' OR 'lorzem' OR 'merlit' OR 'mesmerin' OR 'nervistop l' OR 'novhepar' OR 'novo-lorazem' OR 'nrl 2' OR 'nrl 3' OR 'nrl2' OR 'nrl3' OR 'orfidal' OR 'orifadal' OR 'placinoral' OR 'pro dorm' OR 'punktyl' OR 'quait' OR 'renaquil' OR 'rocosgen' OR 'saphira' OR 'securit' OR 'sedatival' OR 'sidenar' OR 'sinestron' OR 'stapam' OR 'subveta' OR 'tavor' OR 'temesta' OR 'temesta expidet' OR 'titus' OR 'tolid' OR 'tranqipam' OR 'trapax' OR 'trapex' OR 'truveta (drug)' OR 'upan' OR 'wy 4036' OR 'wypax' OR 'lorazepam':ab,kw,ti |
| #92 | #90 OR #91 |
| #93 | 'benzodiazepine'/exp OR 'benzodiazepine' |
| #94 | 'benzodiazepine'/exp OR 'benzodiazepine':ab,kw,ti |
| #95 | #93 OR #94 |
| #96 | 'diazepam'/exp OR 'diazepam' |
| #97 | '1, 4 benzodiazepin 2 one, 7 chloro 1, 3 dihydro 1 methyl 5 phenyl 2h' OR '7 chloro 1 methyl 5 phenyl 3h 1, 4 benzodiazepin 2 (1h) one' OR '7 chloro 1, 3 dihydro 1 methyl 5 phenyl (2h) 1, 4 benzodiazepin 2 one' OR '7 chloro 1, 3 dihydro 1 methyl 5 phenyl 2h 1, 4 benzodiazepin 2 one' OR 'alboral' OR 'aliseum' OR 'alupram' OR 'amiprol' OR 'ansiolin' OR 'antenex' OR 'anxicalm' OR 'anxionil' OR 'apaurin' OR 'apaurine' OR 'apo-diazepam' OR 'apozepam' OR 'aqst 203' OR 'aqst203' OR 'armonil (diazepam)' OR 'arzepam' OR 'assival' OR 'atensine' OR 'audium' OR 'azedipamin' OR 'azepium' OR 'benzopin' OR 'betapam' OR 'bialzepam' OR 'bialzepan' OR 'calmpose' OR 'caudel' OR 'cercin' OR 'cercine' OR 'cersine' OR 'chlordiazepam' OR 'compaz' OR 'desconet' OR 'diaceplex' OR 'dialag' OR 'dialar' OR 'diano' OR 'diapam' OR 'diapanil' OR 'diapax' OR 'diapin' OR 'diapine' OR 'diapo' OR 'diaquel' OR 'diastat' OR 'diastat acudial' OR 'diastat paediatric' OR 'diastat pediatric' OR 'diazelium' OR 'diazem (diazepam)' OR 'diazemuls' OR 'diazepam intensol' OR 'diazepan' OR 'diazepeks' OR 'diazepen' OR 'diazepin' OR 'diazetop' OR 'diazidem' OR 'dipaz' OR 'dipezona' OR 'dizac' OR 'doval' OR 'drenian' OR 'ducene' OR 'dupin' OR 'duxen' OR 'elcion cr' OR 'eridan' OR 'euphorin p' OR 'eurosan' OR 'evacalm' OR 'fanstan' OR 'faustan' OR 'gewacalm' OR 'gubex' OR 'kratium' OR 'kratium 2' OR 'la 111' OR 'la iii' OR 'la111' OR 'lamra' OR 'lembrol' OR 'libervant' OR 'lipodiazepam' OR 'lorinon' OR 'lovium' OR 'melode' OR 'mentalium' OR 'methyl diazepinone' OR 'methyldiazepinon' OR 'methyldiazepinone' OR 'micropam' OR 'morosan' OR 'neocalme' OR 'neorelium' OR 'neurolytril' OR 'nivalen' OR 'noan' OR 'normabel' OR 'novazam' OR 'nrl 1' OR 'nrl1' OR 'nsc 77518' OR 'nsc77518' OR 'ortopsique' OR 'paceum' OR 'pacitran' OR 'paxum' OR 'placidox 10' OR 'placidox 2' OR 'placidox 5' OR 'plidan' OR 'propam' OR 'psychopax' OR 'q-pam' OR 'radizepam' OR 'relanium' OR 'relium' OR 'reliver' OR 'relsed' OR 'remedium' OR 'reposepan' OR 'ro 05 2807' OR 'ro 5 2807' OR 'ro052807' OR 'ro52807' OR 'saromet' OR 'sedapam' OR 'seduxen' OR 'serendin' OR 'setonil' OR 'simasedan' OR 'sipam' OR 'sonacon' OR 'stedon' OR 'stesolid' OR 'stesolid novum' OR 'stesolid rectal tube' OR 'stesolin' OR 'tanquo tablinen' OR 'tensium (diazepam)' OR 'tranimul' OR 'tranquirit' OR 'tranquo puren' OR 'trazepam' OR 'umbrium' OR 'unisedil' OR 'valaxona' OR 'valiquid' OR 'valium' OR 'valium mixed micelles' OR 'valium mm' OR 'valpam' OR 'valrelease' OR 'valtoco' OR 'vanconin' OR 'vatran' OR 'vazen' OR 'vival' OR 'vivol' OR 'wy 3467' OR 'wy3467' OR 'xp 0863' OR 'xp0863' OR 'zetran' OR 'diazepam':ab,kw,ti |
| #98 | #96 OR #97 |
| #99 |  |
| #100 | '7 chloro 1, 3 dihydro 3 hydroxy 5 phenyl 2 h 1, 4 benzodiazepin 2 one' OR '7 chloro 1, 3 dihydro 3 hydroxy 5 phenyl 2h 1, 4 benzodiazepin 2 one' OR '7 chloro 1, 3 dihydro 3 hydroxy 5 phenyl 2h 1, 4 benzodiazepine 2 one' OR '7 chloro 3 hydroxy 5 phenyl 1, 3 dihydro 2h 1, 4 benzodiazepin 2 one' OR 'abboxapam' OR 'adumbran' OR 'alepam' OR 'alopam' OR 'anxiolit' OR 'anxiolit retard' OR 'apo-oxazepam' OR 'azutranquil' OR 'benzotran' OR 'durazepam' OR 'expidet' OR 'hilong' OR 'isodin' OR 'linbial' OR 'medopam' OR 'murelax' OR 'nesontil' OR 'noctazepam' OR 'noripam' OR 'oksazepam' OR 'opamox' OR 'ox-pam' OR 'oxahexal' OR 'oxaline' OR 'oxapam' OR 'oxapuren' OR 'oxepam' OR 'praxiten' OR 'primizum' OR 'propax' OR 'psiquiwas' OR 'purata' OR 'quilibrex' OR 'serax' OR 'serefar' OR 'serenid' OR 'serepax' OR 'seresta' OR 'seresta forte' OR 'serpax' OR 'sigacalm' OR 'simazepan' OR 'sobile' OR 'sobril' OR 'tazepam' OR 'uskan' OR 'vaben' OR 'wakazepam' OR 'wy 3498' OR 'z 10tr' OR 'zaxopam' OR 'oxazepam':ab,kw,ti |
| #101 | #99 OR #100 |
| #102 | 'midazolam'/exp OR 'midazolam' |
| #103 | '8 chloro 6 (2 fluorophenyl) 1 methyl 4 hydroimidazo [1, 5 a] [1, 4] benzodiazepine' OR '8 chloro 6 (2 fluorophenyl) 1 methyl 4h imidazo [1, 5 a] [1, 4] benzodiazepine' OR '8 chloro 6 (2 fluorophenyl) 1 methylimidazo [1, 5 a] [1, 4] benzodiazepine' OR '8 chloro 6 (2 fluorophenyl) 4 hydro 1 methylimidazo [1, 5 a] [1, 4] benzodiazepine' OR 'adv 6209' OR 'adv6209' OR 'af 0901' OR 'af0901' OR 'buccolam' OR 'dalam' OR 'doricum' OR 'dormonid' OR 'fortanest' OR 'fulsed' OR 'hypnoval' OR 'hypnovel' OR 'hypnoyvel' OR 'ipnovel' OR 'iti 111' OR 'iti111' OR 'midacum' OR 'midafresa' OR 'midazo' OR 'midazol' OR 'midazolam hydrochloride' OR 'midazolam hydrochloride preservative free' OR 'midazolam in sodium chloride 0.9%' OR 'midolam' OR 'miloz' OR 'miprosed' OR 'nasolam' OR 'nayzilam' OR 'nazolam' OR 'nvd 301' OR 'nvd301' OR 'ozalin' OR 'ozased' OR 'ro 21 3981' OR 'ro 21 3981 003' OR 'ro 21-3981' OR 'ro 21-3981-003' OR 'ro 213981' OR 'ro 213981003' OR 'ro21 3981' OR 'ro21 3981 003' OR 'ro21-3981' OR 'ro21-3981-003' OR 'ro213981' OR 'ro213981003' OR 'seizalam' OR 'shp 615' OR 'shp615' OR 'suda 003' OR 'suda003' OR 'tak 815' OR 'tak815' OR 'usl 261' OR 'usl261' OR 'versed' OR 'midazolam':ab,kw,ti |
| #104 | #102 OR #103 |
| #105 | 'estazolam'/exp OR 'estazolam' |
| #106 | '12 chloro 9 phenyl 2, 4, 5, 8 tetraazatricyclo [8.4.0.0 (2, 6)] tetradeca 1 (10), 3, 5, 8, 11, 13 hexaene' OR '8 chloro 6 phenyl 4h 1, 2, 4 triazolo [4, 3 a] [1, 4] benzodiazepine' OR '8 chloro 6 phenyl 4h 2, 3, 5, 10b tetraazabenzo [e] azulene' OR '8 chloro 6 phenyl 4h s triazolo [4, 3 a] [1, 4] benzodiazepine' OR 'abbott 47631' OR 'd 40 ta' OR 'd 40ta' OR 'd40ta' OR 'domnamid' OR 'esilgan' OR 'eszo 2' OR 'eurodin' OR 'kainever' OR 'nuctalon' OR 'prosom' OR 'tasedan' OR 'u 33737' OR 'u33737' OR 'estazolam':ab,kw,ti |
| #107 | #105 OR #106 |
| #108 | 'alprazolam'/exp OR 'alprazolam' |
| #109 | '8 chloro 1 methyl 6 phenyl 4h 1, 2, 4 triazolo [4, 3 a] [1, 4] benzodiazepine' OR '8 chloro 1 methyl 6 phenyl 4h [1, 2, 4] triazolo [4, 3 a] [1, 4] benzodiazepine' OR '8 chloro 1 methyl 6 phenyl 4h s triazolo [4, 3 a] [1, 4] benzodiazepine' OR 'abaxon' OR 'aceprax' OR 'afobam' OR 'aisi' OR 'alcelam' OR 'aldeprelam' OR 'alganax' OR 'alnax' OR 'alpaz' OR 'alplax' OR 'alpragen' OR 'alpralid' OR 'alpraline' OR 'alpram' OR 'alpranax' OR 'alprasedon' OR 'alprastad' OR 'alpratop' OR 'alprax' OR 'alpraz' OR 'alprazig' OR 'alprocontin' OR 'alprox (alprazolam)' OR 'altraxic' OR 'altrox' OR 'alviz' OR 'alzam' OR 'alzax' OR 'alzolam' OR 'anax' OR 'anpress' OR 'anxirid' OR 'anzion' OR 'apo-alpraz' OR 'apraz' OR 'aprazolam' OR 'az 002' OR 'az002' OR 'azolitan' OR 'bestrol' OR 'calmdown (drug)' OR 'calmlet' OR 'cassadan' OR 'constan' OR 'constante (drug)' OR 'daclor' OR 'dixin' OR 'dizolam' OR 'drimpam' OR 'feprax' OR 'frixitas' OR 'frontal (drug)' OR 'frontin' OR 'gerax' OR 'helex' OR 'helex (drug)' OR 'kalma (alprazolam)' OR 'kinax' OR 'ksalol' OR 'marzolam' OR 'medepolin' OR 'mialin' OR 'misar' OR 'misar sr' OR 'neupax' OR 'neurol (alprazolam)' OR 'neurol sr (alprazolam)' OR 'niravam' OR 'nu alpraz' OR 'nu-alpraz' OR 'panix' OR 'pazolam' OR 'pharnax' OR 'prazol' OR 'prazolan (alprazolam)' OR 'prazolex' OR 'prinox' OR 'ralozam' OR 'renax' OR 'restyl' OR 'sapram' OR 'saturnil' OR 'solanax' OR 'staccato alprazolam' OR 'stap 001' OR 'stap001' OR 'tafil' OR 'tafil ap' OR 'tafil d' OR 'tafil retard' OR 'tensium (alprazolam)' OR 'tensivan' OR 'trankimazin' OR 'tranquinal' OR 'tricalma (alprazolam)' OR 'u 31889' OR 'u31889' OR 'ucb 7538' OR 'ucb7538' OR 'unilan' OR 'val 1182' OR 'val1182' OR 'valeans' OR 'xanacine' OR 'xanagis' OR 'xanax' OR 'xanax sr' OR 'xanax ts' OR 'xanax xr' OR 'xanolam' OR 'xanor' OR 'xanor xr' OR 'zacetin' OR 'zanapam' OR 'zenax' OR 'zolam' OR 'zolaram' OR 'zolarem' OR 'zoldac' OR 'zomiren' OR 'zomiren sr' OR 'zopax' OR 'zopic' OR 'zotran' OR 'zypraz' OR 'alprazolam':ab,kw,ti 7478  #108 'alprazolam'/exp OR 'alprazolam' |
| #110 | #108 OR #109 |
| #111 | #92 OR #95 OR #98 OR #101 OR #104 OR #107 OR #110 |
| #112 | 'carbamazepine'/exp OR 'carbamazepine' |
| #113 | '(5h) dibenz (b, f) azepine 5 carboxamide' OR '5 carbamoyl 5h dibenz [b, f] azepine' OR '5h dibenz [b, f] azepine 5 carboxamide' OR '5h dibenzo [b, f] azepine 5 carboxamide' OR 'amizepin' OR 'amizepine' OR 'apo-carbamazepine' OR 'atretol' OR 'bipotrol' OR 'biston' OR 'calepsin' OR 'camapine' OR 'carbadac' OR 'carbadura' OR 'carbagen' OR 'carbalex' OR 'carbamazepin' OR 'carbategral' OR 'carbatol' OR 'carbatrol' OR 'carbazene' OR 'carbazep' OR 'carbazina' OR 'carbella' OR 'carbepsil' OR 'carmaz' OR 'carnexiv' OR 'carpaz' OR 'carzepin' OR 'carzepine' OR 'cezemin' OR 'clostedal' OR 'convuline (carbamazepine)' OR 'curatil' OR 'epileptol' OR 'epileptol cr' OR 'epimax' OR 'epitol' OR 'eposal retard' OR 'equetro' OR 'espa-lepsin' OR 'finlepsin' OR 'foxalepsin' OR 'foxalepsin retard' OR 'g 32883' OR 'g32883' OR 'geigy 32883' OR 'geigy32883' OR 'hermolepsin' OR 'karbamazepin' OR 'karbelex' OR 'kodapan' OR 'lexin' OR 'mazepine' OR 'mazetol' OR 'meizine' OR 'neugeron' OR 'neurotol' OR 'neurotop' OR 'neurotop retard' OR 'nordotol' OR 'painbrake (drug)' OR 'panitol' OR 'servimazepin' OR 'sirtal' OR 'spd 417' OR 'spd417' OR 'stazepine' OR 'storilat' OR 'syntopine' OR 'tardotol' OR 'taver' OR 'tegol' OR 'tegral' OR 'tegretal' OR 'tegretol' OR 'tegretol cr' OR 'tegretol divitab' OR 'tegretol retard' OR 'tegretol xr' OR 'tegretol-s' OR 'tegretol-xr' OR 'tegrital' OR 'telesmin' OR 'temporal slow' OR 'temporol' OR 'teril' OR 'timonil' OR 'timonil retard' OR 'carbamazepine':ab,kw,ti |
| #114 | 'gabapentin'/exp OR 'gabapentin' |
| #115 | '1 (aminomethyl) cyclohexaneacetic acid' OR '2 [1 (aminomethyl) cyclohexyl] acetic acid' OR 'ci 945' OR 'ci945' OR 'dineurin' OR 'dm 1796' OR 'dm 5689' OR 'dm1796' OR 'dm5689' OR 'gabalept' OR 'gabaliquid geriasan' OR 'gabapen' OR 'gabatin' OR 'gantin' OR 'go 3450' OR 'go3450' OR 'goe 3450' OR 'goe3450' OR 'gralise' OR 'kaptin' OR 'keneil' OR 'neurontin' OR 'neurotonin' OR 'nupentin' OR 'sefelsa' OR 'serada' OR 'gabapentin':ab,kw,ti |
| #116 | #114 OR #115 |
| #117 | #112 OR #113 |
| #118 | #116 OR #117 |
| #119 | 'sevoflurane'/exp OR 'sevoflurane' |
| #120 | 'sevoflurane'/exp OR 'sevoflurane':ab,kw,ti |
| #121 | #119 OR #120 |
| #122 | 'propofol'/exp OR 'propofol' |
| #123 | '2, 6 bis (1 methylethyl) phenol' OR '2, 6 di (2 propanyl) phenol' OR '2, 6 di (2 propyl) phenol' OR '2, 6 di (propan 2 yl) phenol' OR '2, 6 diisopropanylphenol' OR '2, 6 diisopropylphenol' OR 'anepol' OR 'anesia' OR 'cryotol' OR 'diisoprofol' OR 'diprivan' OR 'diprofol' OR 'disoprivan' OR 'disoprofol' OR 'fresofol' OR 'gobbifol' OR 'hiremon (propofol)' OR 'ici 35 868' OR 'ici 35868' OR 'plofed' OR 'pofol' OR 'profast' OR 'propocam' OR 'propoflo' OR 'propofol lipoven' OR 'propofol lipuro' OR 'propofol mct/lct' OR 'propofol-lipuro' OR 'propolipid' OR 'propoven' OR 'provive' OR 'rapanofal' OR 'rapinovet' OR 'rapiva' OR 'recofol' OR 'recofol n' OR 'ripol (propofol)' OR 'safol' OR 'spifol' OR 'spiva (drug)' OR 'unifol (propofol)' OR 'propofol':ab,kw,ti |
| #124 | 'cimetidine'/exp OR 'cimetidine' |
| #125 | '1 cyano 2 methyl 3 [2 [ [(5 methylimidazol 4 yl) methyl] thio] ethyl] guanidine' OR '1 methyl 3 [2 (5 methylimidazol 4 ylmethylthio) ethyl] guanidine 2 carbonitrile' OR '4 [ [[2 (3 cyano 2 methylguanidin 1 yl) ethyl] thio] methyl] 5 methylimidazole' OR 'aci-med' OR 'acibilin' OR 'acidnor' OR 'acinil' OR 'aidar' OR 'altramet' OR 'antag' OR 'apo-cimetidine' OR 'asaurex' OR 'azucimet' OR 'belomet' OR 'biomag' OR 'biomet' OR 'brumetidina' OR 'campanex' OR 'cemedin 200' OR 'cemedin 400' OR 'cemedin 800' OR 'cementin' OR 'ciclem' OR 'cigamet' OR 'cignatin' OR 'ciket m' OR 'cimal' OR 'cimbene' OR 'cimedine' OR 'cimehexal' OR 'cimeldine' OR 'cimeloc' OR 'cimet' OR 'cimetag' OR 'cimetalgin' OR 'cimetase' OR 'cimetex' OR 'cimetid' OR 'cimetidin' OR 'cimetidina' OR 'cimetidine hydrochloride' OR 'cimetidine hydrochloride in sodium chloride 0.9%' OR 'cimetidine monohydrochloride monohydrate' OR 'cimetigal' OR 'cimetin' OR 'cimetum' OR 'cimewell' OR 'cimlok' OR 'cimulcer' OR 'cinadine' OR 'cinulcus' OR 'cismetin' OR 'citidine' OR 'citius' OR 'contracid' OR 'corsamet' OR 'cylock' OR 'cytine' OR 'dispamet' OR 'duomet' OR 'dyspamet' OR 'edalene' OR 'eladene' OR 'erlmetin' OR 'eureceptor' OR 'fremet' OR 'gastab' OR 'gastidine' OR 'gastrobitan' OR 'gastroprotect' OR 'gawei' OR 'getidin' OR 'haldin' OR 'hexamet' OR 'himetin' OR 'histodil' OR 'inesfay' OR 'lenamet' OR 'lenamet otc' OR 'lock 2' OR 'magicul' OR 'manomet' OR 'maritidine' OR 'med-gastramet' OR 'meticon' OR 'n cyano n` methyl n`` [2 [ [(5 methyl 1h imidazol 4 yl) methyl] thio] ethyl] guanidine' OR 'neutromed' OR 'neutronorm' OR 'neutronorm retard' OR 'novocimetine' OR 'nulcer' OR 'peptol' OR 'piovalen' OR 'pondarmett' OR 'powegon' OR 'promet' OR 'sanmetidin' OR 'secapine' OR 'senoba' OR 'sertidine' OR 'shintamet' OR 'siamidine' OR 'sigmetadine' OR 'simaglen' OR 'sk and f 92234' OR 'skf 92334' OR 'stogamet' OR 'stomakon' OR 'stomedine' OR 'stomet' OR 'tagagel' OR 'tagamet' OR 'tagamet hb' OR 'tagamet hb 200' OR 'tagamet hydrochloride in sodium chloride 0.9%' OR 'tagma' OR 'tametin' OR 'tobymet' OR 'ulcedin' OR 'ulcedine (cimetidine)' OR 'ulcemet' OR 'ulcenon' OR 'ulcerfen' OR 'ulcidine' OR 'ulcim' OR 'ulcimet' OR 'ulcodina' OR 'ulcolind h2' OR 'ulcomedina' OR 'ulcomet' OR 'ulcumet' OR 'ulsikur' OR 'umamett' OR 'weisdin' OR 'xepamet' OR 'zymerol' OR 'cimetidine':kw,ab,ti |
| #126 | #124 OR #125 |
| #127 | #122 OR #123 |
| #128 | 'ranitidine'/exp OR 'ranitidine' |
| #129 | 'acidex (ranitidine)' OR 'aciloc' OR 'acloral' OR 'acran' OR 'ah 19065' OR 'ah19065' OR 'aldin' OR 'alquen' OR 'anistal' OR 'antagonin' OR 'apo-ranitidine' OR 'ardoral' OR 'atural' OR 'ausran' OR 'avintac' OR 'axoban' OR 'azantac' OR 'baroxal' OR 'consec' OR 'coralen' OR 'cygran' OR 'd 14951' OR 'd14951' OR 'duractin' OR 'eltidine' OR 'eu-ran' OR 'ezopta' OR 'galidrin' OR 'gastran' OR 'gastrial' OR 'gastridina' OR 'gastrosedol' OR 'heartburn and indigestion relief (ranitidine)' OR 'hexer' OR 'histac' OR 'histak' OR 'hyzan' OR 'incid' OR 'indigestion relief (ranitidine)' OR 'iqfadina' OR 'kemoranin' OR 'kiradin' OR 'lumaren' OR 'lydin' OR 'microtid' OR 'midaven' OR 'n [2 [ [5 [ (dimethylamino) methyl] 2 furanyl] methylsulfanyl] ethyl] n` methyl 2 nitroethene 1, 1 diamine' OR 'n [2 [ [5 [ (dimethylamino) methyl] 2 furanyl] methylthio] ethyl] n` methyl 2 nitroethene 1, 1 diamine' OR 'n [2 [ [5 [ (dimethylamino) methyl] furan 2 yl] methylsulfanyl] ethyl] n` methyl 2 nitroethene 1, 1 diamine' OR 'n [2 [ [5 [ (dimethylamino) methyl] furan 2 yl] methylthio] ethyl] n` methyl 2 nitroethene 1, 1 diamine' OR 'n [2 [ [5 [ (dimethylamino) methyl] furfuryl] thio] ethyl] n` methyl 2 nitro 1, 1 ethenediamine' OR 'nadine' OR 'neoceptin-r' OR 'novo-ranidine' OR 'pilorex' OR 'ponaltin' OR 'ptinolin' OR 'quantor' OR 'quicran' OR 'r-loc' OR 'radinat' OR 'radine' OR 'rafitaz' OR 'ranacid' OR 'rancet' OR 'randin' OR 'rani 2' OR 'ranial' OR 'raniben' OR 'ranibloc' OR 'ranicalm' OR 'ranidil' OR 'ranidine' OR 'ranidura' OR 'ranigast max' OR 'ranigast pro' OR 'ranihexal' OR 'ranimex' OR 'ranin' OR 'raniogas' OR 'raniplex' OR 'ranisan' OR 'ranisen' OR 'ranitab' OR 'ranital' OR 'ranitax' OR 'ranitidin' OR 'ranitidina' OR 'ranitidine hydrochloride' OR 'ranitil' OR 'ranitin' OR 'ranitine' OR 'ranolta' OR 'rantac' OR 'rantacid' OR 'rantin' OR 'ranuber' OR 'ranzac' OR 'ratic' OR 'raticina' OR 'raxide' OR 'retamin' OR 'rolan' OR 'rosimol' OR 'simetac' OR 'sostril' OR 'tanidina' OR 'taural' OR 'terodul' OR 'toriol' OR 'ulcaid' OR 'ulceran' OR 'ulceranin' OR 'ulcex' OR 'ulcin' OR 'ulcocur' OR 'ulsal' OR 'ultak' OR 'ultidine' OR 'urantac' OR 'verlost' OR 'vesyca' OR 'vizerul' OR 'weichilin' OR 'weidos' OR 'xanidine' OR 'zantab' OR 'zantac' OR 'zantac 150' OR 'zantac 25' OR 'zantac 300' OR 'zantac 75' OR 'zantac efferdose' OR 'zantac fr' OR 'zantac relief' OR 'zantadin' OR 'zantic' OR 'zinetac' OR 'ranitidine':ab,kw,ti |
| #130 | #128 OR #129 |
| #131 | 'randomized controlled trial'/exp OR 'randomized controlled trial' |
| #132 | 'controlled trial, randomized' OR 'randomised controlled study' OR 'randomised controlled trial' OR 'randomized controlled study' OR 'trial, randomized controlled' OR 'randomized controlled trial':ab,ti,kw |
| #133 | #121 OR #126 OR #127 OR #130 |
| #134 | #10 OR #47 OR #63 OR #76 OR #89 OR #111 OR #118 OR #133 |
| #135 | 'subacute delirium' OR 'delirium, subacute' OR 'deliriums, subacute' OR 'subacute deliriums' OR 'delirium of mixed origin' OR 'mixed origin delirium' OR 'mixed origin deliriums' OR 'emergence delirium' OR 'confusion' OR 'disorientation' OR 'bewilderment':ab,kw,ti |
| #136 | #3 OR #135 |
| #137 | 'controlled clinical trial' OR randomized OR randomised OR placebo OR controlled OR random* OR trial OR 'blind* or mask*':ab,kw,ti |
| #138 | #131 OR #132 OR #137 |
| #139 | #134 AND #136 AND #138 |

**D.Web of Science**

| #1 | TS=（“Delirium” OR “Subacute Delirium” OR “Delirium, Subacute” OR “Deliriums, Subacute” OR “Subacute Deliriums” OR “Delirium of Mixed Origin” OR “Mixed Origin Delirium” OR “Mixed Origin Deliriums” OR “Emergence Delirium” OR “Confusion” OR “Disorientation” OR “Bewilderment”） |
| --- | --- |
| #2 | TS=(Melatonin OR “Receptors, Melatonin” OR “Receptor, Melatonin” OR “Melatonin Receptor” OR “Melatonin Receptors” OR “Receptor, Melatonin, MT3” OR “Melatonin MT3 Receptor” OR “MT3 Receptor, Melatonin” OR “Receptor, Melatonin MT3” OR “MT3 Receptor” OR “Receptor, MT3” OR “Receptor, Melatonin, 1C” OR “Receptor, Mel1c” OR “Mel1c Receptor” OR “Mel(1c) Receptor” OR “Receptor, Melatonin, MT1” OR “Receptor, Melatonin, MT2”) |
| #3 | TS=(“Antipsychotic Agents” OR “Tranquillizing Agents, Major” OR “Major Tranquillizing Agents” OR “Major Tranquilizer” OR “Tranquilizer, Major” OR “Antipsychotic Drug” OR “Drug, Antipsychotic” OR “Antipsychotic Agent” OR “Agent, Antipsychotic” OR “Antipsychotic Medication” OR “Medication, Antipsychotic” OR “Neuroleptic Agent” OR “Agent, Neuroleptic” OR “Neuroleptic Drug” OR “Drug, Neuroleptic” OR “Neuroleptic” OR “Antipsychotic” OR “Antipsychotic Drugs” OR “Antipsychotics” OR “Major Tranquilizers” OR “Neuroleptic Agents” OR “Neuroleptic Drugs” OR “Neuroleptics” OR “Tranquilizing Agents, Major” OR “Major Tranquilizing Agents” OR “Antipsychotic Effect” OR “Effect, Antipsychotic” OR “Antipsychotic Effects” OR “Haloperidol” OR “Haldol” OR “Quetiapine” OR “Ethanol,2-(2-(4-dibenzo(b,f)(1,4)thiazepin-11-yl-1-piperazinyl)ethoxy)-,(E)-2-butenedioate (2:1) (salt)” OR “Seroquel” OR “ICI 204,636” OR “ICI-204636” OR “ICI204636” OR “ICI 204636” OR “2-(2-(4-dibenzo(b,f)(1,4)thiazepine-11-yl-1-piperazinyl)ethoxy)ethanol” OR “Risperidone” OR “Risperdal Consta” OR “Consta, Risperdal” OR “Risperidal” OR “R-64,766” OR “R 64,766” OR “R64,766” OR “R-64766” OR “R 64766” OR “R64766” OR “Clozapine” OR “Clozaril” OR “Leponex” OR “Ziprasidone” OR “ziprasidone” OR “5-(2-(4-(3-benzisothiazolyl)piperazinyl)ethyl)-6-chloro-1,3-dihydro-2H-indol-2-one” OR “ziprasidone hydrochloride” OR “CP 88059-01” OR “CP-88,059-1” OR “CP-88,059-01” OR “ziprasidone hydrochloride, monohydrate” OR “Geodon” OR “CP 88059” OR “CP-88,059” OR “Chlorpromazine” OR “Thorazine” OR “Aminazine” OR “Largactil” OR “Chlordelazine” OR “Chlorpromazine Hydrochloride” OR “Hydrochloride, Chlorpromazine” OR “Contomin” OR “Fenactil” OR “Propaphenin” OR “Chlorazine OR Chlordiazepoxide” OR “7-Chloro-N-methyl-5-phenyl-3H-1,4-benzodiazepin-2-amine 4-oxide” OR “7 Chloro N methyl 5 phenyl 3H 1,4 benzodiazepin 2 amine 4 oxide” OR “Methaminodiazepoxide” OR “7-Chloro-2-methylamino-5-phenyl-3H-1,4-benzodiazepine-4-oxide” OR “7 Chloro 2 methylamino 5 phenyl 3H 1,4 benzodiazepine 4 oxide” OR “Chlordiazepoxide Hydrochloride” OR “Hydrochloride, Chlordiazepoxide” OR “Chlordiazepoxide Monohydrochloride” OR “Monohydrochloride, Chlordiazepoxide” OR “Librium” OR “Chlozepid” OR “Elenium” OR “Chlordiazepoxide Hydrobromide” OR “Hydrobromide, Chlordiazepoxide” OR “Chlordiazepoxide Perchlorate” OR “Perchlorate, Chlordiazepoxide” OR “Sultopride” OR “4-Amino-N-((1-ethyl-2-pyrrolidinyl)methyl)-5-(ethylsulfonyl)-2-methoxybenzamide” OR “DAN 2163” OR “DAN-2163” OR “Solian” OR “Sultopride” OR “N-(Ethyl-1-pyrrolidinyl- 2-methyl)methoxy-2-ethylsulfonyl-5-benzamide” OR “LIN 1418” OR “LIN-1418” OR “Barnetil” OR “Sultopride Hydrochloride” OR “Sulpiride” OR “Olanzapine” OR “2-Methyl-4-(4-methyl-1-piperazinyl)-10H-thieno(2,3-b)(1,5)benzodiazepine” OR “LY-170052” OR “LY 170052” OR “LY170052” OR “Zyprexa” OR “Zolafren” OR “LY 170053” OR “Olanzapine Pamoate” OR “Amisulpride” ) |
| #4 | TS=(Pregabalin OR “(S)-3-(aminomethyl)-5-methylhexanoic acid” OR “3-isobutyl GABA” OR “3 isobutyl GABA” OR “GABA, 3-isobutyl” OR “3-(aminomethyl)-5-methylhexanoic acid” OR “(R-)-3-isobutyl GABA” OR “(S+)-3-isobutyl GABA” OR Lyrica OR “CI 1008” OR “1008, CI” OR “CI-1008” OR CI1008Cimetidine OR Chlormethiazole OR Clomethiazole OR Distraneurin) |
| #5 | TS=(Ketamine OR “2-(2-Chlorophenyl)-2-(methylamino)cyclohexanone” OR CI-581 OR “CI 581” OR CI581 OR Ketalar OR Ketaset OR Ketanest OR Calipsol OR Kalipsol OR Calypsol OR Ketamine) |
| #6 | TS=(Baclofen OR Baclophen OR PCP-GABA OR “beta-(Aminomethyl)-4-chlorobenzenepropanoic Acid” OR “Chlorophenyl GABA” OR “GABA, Chlorophenyl” OR “beta-(p-Chlorophenyl)-gamma-aminobutyric Acid” OR Clofen OR Gen-Baclofen OR “Gen Baclofen” OR GenBaclofen OR Genpharm OR Apo-Baclofen OR “Apo Baclofen” OR ApoBaclofen OR Atrofen OR Baclofène-Irex OR Baclofène Irex OR BaclofèneIrex OR “Baclofen AWD” OR “AWD, Baclofen” OR Lebic OR Lioresal OR Liorésal OR Nu-Bacl OR Nu Bacl OR NuBaclo OR PMS-Baclofen OR “PMS Baclofen” ORPMSBaclofen OR Baclospas OR Ba-34,647 OR “Ba34,647” OR Ba-34647 ORBa34647 OR CIBA-34,647-BA OR “CIBA34,647BA”) |
| #7 | TS=(Clonidine OR Klofenil OR Clofenil OR Chlophazolin OR “Clonidine Dihydrochloride” OR “Dihydrochloride, Clonidine” OR “Clonidine Hydrochloride” OR “Hydrochloride, Clonidine” OR “Clonidine Monohydrochloride” OR “Monohydrochloride, Clonidine” OR “Clonidine Monohydrobromide” OR “Monohydrobromide, Clonidine” OR ST-155 OR “ST 155” OR ST155 OR Gemiton OR Hemiton OR Isoglaucon OR Klofelin OR Clofelin OR Clopheline OR M-5041T OR M 5041T OR M5041T OR Catapres OR Catapresan OR Catapressan OR Dixarit) |
| #8 | TS=(Anesthetics OR “Anesthetic Agents” OR “Agents, Anesthetic” OR “Anesthetic DrugsZ” OR “Drugs, Anesthetic” OR Anesthetic OR “Anesthetic Effect” OR “Effect, Anesthetic” OR “Anesthetic Effects” OR “Effects, Anesthetic” OR Morphine OR Morphia OR “Morphine Chloride” OR “Chloride, Morphine” OR “Morphine Sulfate” OR “Sulfate, Morphine” OR “SDZ 202-250” OR “SDZ 202 250” OR “SDZ 202250” OR “SDZ202-250” OR “SDZ202 250” OR SDZ202250 OR “Morphine Sulfate (2:1), Pentahydrate” OR “MS Contin” OR “Contin, MS” OR “Oramorph SR” OR Duramorph OR “Morphine Sulfate (2:1), Anhydrous” OR Pethidine OR Isonipecain OR Dolsin OR Dolosal OR Dolin OR “Operidine EPJ-I” OR “Operidine EPJ I” OR Dolantin OR Dolargan OR “Meperidine Hydrochloride” OR Lidol OR Lydol OR Demerol OR Dolcontral) |
| #9 | TS=(Dexamethasone OR Methylfluorprednisolone OR Hexadecadrol OR Decamet OR Decaspray OR Dexasone OR deepak OR Maxidex OR Millicorten OR Oradexo OR Decajec OR Decaject-L.A. OR Decaject L.A. OR Hexadrol) |
| #10 | TS=(Sedative OR “Sedatives and Hypnotics” OR “Sedative and Hypnotic” OR “Hypnotic and Sedative” OR “Hypnotic Effect” OR “Effect, Hypnotic” OR “Hypnotic Effects” OR “Effects, Hypnotic” OR “Sedatives” OR “Hypnotics” OR “Hypnotic” OR “Sedative Effect” OR “Effect, Sedative” OR “Sedative Effects” OR “Effects, Sedative” OR “Sleep Aids, Pharmaceutical” OR “Orexin Receptor Antagonists” OR Dexmedetomidine OR MPV-1440 OR “MPV 1440” OR MPV1440 OR Precedex OR Dexdomitor OR Sedadex OR Sileo OR Cepedex OR Dexdor OR “Dexmedetomidine Hydrochloride” OR “Hydrochloride, Dexmedetomidine” OR Igalmi) |
| #11 | TS=(Suvorexant OR “(4-(5-chloro-1,3-benzoxazol-2-yl)-7-methyl-1,4-diazepan-1-yl)(5-methyl-2-(2H-1,2,3-triazol-2-yl)phenyl)methanone” OR “methanone, ((7R)-4-(5-chloro-2-benzoxazolyl)hexahydro-7-methyl-1H-1,4-diazepin-1-yl)(5-methyl-2-(2H-1,2,3-triazol-2-yl)phenyl)-“ OR “MK 4305” OR “MK4305” OR “MK-4305” OR Belsomra OR Ramelteon OR “(S)-N-(2-(1,6,7,8-tetrahydro-2H-indeno-(5,4)furan-8-yl)ethyl)propionamide” OR TAK-375 OR Rozerem) |
| #12 | TS=(Benzodiazepine OR “Benzodiazepine Compounds” OR Benzodiazepine OR “Lorazepa” OR “Ativan” OR “Orfidal Wyeth” OR “Wyeth, Orfidal” OR Témesta OR Tolid OR Doni OR Duralozam OR Durazolam OR Idalprem OR Laubeel OR “Lorazep Von Ct” OR “Von Ct, Lorazep” OR “Lorazepam Medical” OR “Medical, Lorazepam” OR “Lorazepam-Neuraxpharm” OR “Lorazepam Neuraxpharm” OR “Lorazepam-Ratiopharm” OR “Lorazepam Ratiopharm” OR Novo-Lorazem OR “Novo Lorazem” OR Nu-Loraz OR “Nu Loraz” OR Sedicepan OR Sinestron OR WY-4036 OR “WY 4036” OR WY4036 OR Apo-Lorazepam OR “Apo Lorazepam” OR Somagerol OR Temesta OR Diazepam OR “7-Chloro-1,3-dihydro-1-methyl-5-phenyl-2H-1,4-benzodiazepin-2-one” OR Diazemuls OR Faustan OR Valium OR Seduxen OR Sibazon OR Stesolid OR Apaurin OR Relanium OR Oxazepam OR Midazolam OR “Midazolam Maleate” OR “Maleate, Midazolam“ OR Dormicum OR Versed OR “Midazolam Hydrochloride” OR “Hydrochloride, Midazolam” OR “Ro 21-3981” OR “Ro 21 3981” OR “Ro 213981” OR Estazolam OR Tasedan OR ProSom OR D-40TA OR D40TA OR Nuctalon OR Alprazolam OR Alprazolan OR Trankimazin OR “U-31,889” OR “U31,889” OR Xanax OR Alprox OR Esparon OR Cassadan OR “D-65MT” OR D65MT OR Kalma OR “Novo-Alprazol” OR “Novo Alprazol” OR Nu-Alpraz OR Nu Alpraz OR Ralozam OR Tafil OR Apo-Alpraz OR “Apo Alpraz”) |
| #13 | TS=(Carbamazepine OR “Carbamazepine Acetate” OR “Carbamazepine Dihydrate” OR “Carbamazepine Hydrochloride” OR “Carbamazepine L-Tartrate (4:1)” OR “Carbamazepine Anhydrous” OR “Carbamazepine Sulfate (2:1)” OR Carbazepin OR Epitol OR Finlepsin OR Neurotol OR Tegretol OR Amizepine OR “Carbamazepine Phosphate” OR Gabapentin OR “1-(Aminomethyl)cyclohexaneacetic Acid” OR Neurontin OR “Gabapentin Hexal” OR Convalis OR Gabapentin-Ratiopharm OR “Gabapentin Ratiopharm” OR Novo-Gabapentin OR “Novo Gabapentin” OR NovoGabapentin OR PMS-Gabapentin OR Apo-Gabapentin OR “Apo Gabapentin” OR ApoGabapentin OR “Gabapentin Stada”) |
| #14 | TS=(Sevoflurane OR “Fluoromethyl-2,2,2-trifluoro-1-(trifluoromethyl)ethyl Ether” OR “Fluoromethyl Hexafluoroisopropyl Ether” OR Sevorane OR Ultane OR “BAX 3084” OR Propofol OR “2,6-Diisopropylphenol” OR “2,6 Diisopropylphenol” OR “2,6-Bis(1-methylethyl)phenol” OR Disoprofol OR Diprivan OR Disoprivan OR Fresofol OR “ICI-35,868” OR “ICI 35,868” OR “ICI35,868” OR “ICI-35868” OR “ICI 35868“ OR ICI35868 OR Ivofol OR “ropofol Fresenius” OR “Propofol MCT” OR “Propofol Rovi” OR “Propofol-Lipuro” OR Recofol OR Aquafol OR “Propofol Abbott”) |
| #15 | TS=(Cimetidine OR “N-Cyano-N'-methyl-N''-(2-(((5-methyl-1H-imidazol-4-yl)methyl)thio)ethyl)guanidine” OR Biomet OR Biomet400 OR “Cimetidine Hydrochloride” OR “Hydrochloride, Cimetidine” OR “Cimetidine HCl” OR “HCl, Cimetidine” OR “Histodil” OR “SK and F-92334” OR “SK and F 92334” OR “SK and F92334” OR “SKF-92334” OR “SKF 92334” OR SKF92334 OR Tagamet OR Altramet OR Eureceptor OR Ranitidine OR Ranitidin OR “N(2-(((5-((Dimethylamino)methyl)-2-furanyl)methyl)thio)ethyl)-N'-methyl-2-nitro-1,1-ethenediamine” OR Biotidin OR Ranisen OR Sostril OR Zantac OR Zantic OR AH-19065 OR “AH 19065” OR AH19065 OR “Ranitidine Hydrochloride” OR “Hydrochloride, Ranitidine”) |
| #16 | #2 OR #3 OR #4 OR #5 OR #6 OR #7 OR #8 OR #9 OR #10 OR #11 OR #12 OR #13 OR #14 OR #15 |
| #17 | TS=(“Randomized controlled trial” OR “ontrolled clinical trial” OR Randomized OR Randomised OR Placebo OR Controlled OR Random* OR Trial OR “Blind* OR Mask*”) |
| #18 | #1 AND #16 AND #17 |

# **APPENDIX 2** All baseline characteristics of the studies

**2.1 The baseline characteristics and the incidence of postoperative cognitive dysfunction of included studies**

| **Study, Year** | **Type of surgery** | **Drug** | **N** | **Age, Years**  **[mean ± SD ]** | **Female**  **[%]** | **Occurrence of cognitive impairment** |
| --- | --- | --- | --- | --- | --- | --- |
| **Chen et al.(2001)** | Orthopedic surgery | Desflurane  Sevoflurane | 35  35 | 75.0±8.0  73.0±9.0 | 42.9 48.6 | 0/35  1/35 |
| **Glumac et al.(2017)** | Cardiac surgery | Dexamethasone  NS | 85  84 | 63.7±9.0  64.2±9.4 | 21.2 21.0 | 9/80 21/81 |
| **Fang et al.(2014)** | Neurosurgery | Dexamethasone NS | 640 320 | 48.5±5.5 48.0 ± 5.8 | 61.1 62.1 | 165/635 71/319 |
| **Guo et al.(2020)** | Non-cardiac surgery | Sevoflurane Propofol | 117 117 | 69.0±5.9 69.0±4.8 | 65.0 60.7 | 32/110  30/110 |
| **Ma et al.(2023)** | Abdominal surgery | Ketamine  NS | 31 31 | 69.5±4.4 70.6±4.2 | 22.6 22.6 | 5/31 12/31 |
| **Lee et al.(2015)** | Orthopedic surgery | Ketamine  NS | 25 26 | 68.3±5.3 68.4±6.5 | 44.0 72.4 | 1/25 8/26 |
| **Verdonk et al.(2024)** | Orthopedic surgery | Ketamine  NS | 149 143 | 72.3±7.1 72.3±7.3 | 62.4 60.8 | 50/149  54/143 |
| **Luo et al.(2024)** | Thoracic surgery | Ketamine  NS | 87 42 | 55.6±9.2 55.9±10.6 | 51.7 47.6 | 16/87 9/42 |
| **Hollinger et al.(2021)** | Mixed Major Surgeries | Haloperidol  Ketamine  NS | 45 47 44 | 73.4±6.3 73.4±6.1 74.8±6.6 | 35.6 44.7 54.5 | 7/45 10/47 6/44 |
| **Li et al.(2019)** | Orthopedic surgery | Dexmedetomidine Propofol Midazolam | 55 55 54 | 69.3±7.1 68.2±6.4 66.9±6.6 | 63.6 56.4 59.3 | 22/55  10/55  28/54 |
| **Takazawa et al.(2023)** | Orthopedic surgery | Minocycline NS | 90 95 | 74.5±6.7 75.0±6.7 | 78.0 80.0 | 8/90  4/95 |
| **Mu et al.(2017)** | Orthopedic surgery | Parecoxib NS | 310 310 | 69.6±6.5 70.5±6.9 | 73.9 73.2 | 28/310 53/310 |
| **Egawa et al.(2016)** | Thoracic Surgery | Propofol Sevoflurane | 72 72 | 69.0±2.5 72.0±2.3 | 31.9 45.8 | 16/72  24/72 |
| **Geng et al.(2017)** | Abdominal surgery | Propofol Sevoflurane Isoflurane | 50 50 50 | N/A N/A N/A | 60.0 56.0 64.0 | 2/50 10/50 15/50 |
| **Wittwer et al.(2023)** | Cardiac surgery | Ketamine Propofol | 25 24 | 76.4±3.5 76.1±5.3 | 36.0 42.0 | 14/22 9/21 |
| **Racman et al.(2023)** | Cardiac surgery | Dexmedetomidine Propofol | 37 34 | 83.0±5.9 83.5±5.9 | 54.1 50.0 | 9/37 20/34 |
| **Kurup et al.(2023)** | Abdominal surgery | Dexmedetomidine Lidocaine | 29 31 | 65.0±8.2 64.0±7.4 | 72.4 64.5 | 7/29 9/31 |
| **Liu et al.(2020)** | ENT Surgery | Dexmedetomidine NS | 30 30 | 43.4±8.9 40.6±9.4 | 16.7 13.3 | 1/30 3/30 |
| **Meineake et al.(2014)** | Non-cardiac surgery | Desflurane Sevoflurane | 37 47 | 72.3±2.4 71.9±1.8 | 64.9 70.2 | 17/37 32/47 |
| **Nagz et al.(2020)** | Orthopedic surgery | Dexmedetomidine NS | 60 60 | 68.3±3.9 68.5±5.0 | 60.0 48.3 | 10/60 21/60 |
| **Ghazaly et al.(2023)** | Emergency surgery | Dexmedetomidine Ketamine NS | 20 20 20 | 70.0±3.8 71.2±3.9 69.4±4.7 | 25.0 50.0 40.0 | 0/20 2/20 7/20 |
| **Chawdhary et al.(2020)** | Non-cardiac surgery | Dexmedetomidine Propofol | 40 40 | 66.2±6.6 64.8±5.9 | 30.0 27.5 | 8/40 11/40 |
| **Gao et al.(2020)** | Cardiac surgery | Dexmedetomidine NS | 30 30 | 69.5±5.1 70.4±4.2 | 53.3 50.0 | 0/30 5/30 |
| **Chen et al.( 2020)** | Abdominal surgery | Dexmedetomidine NS | 43 45 | 64.9±11.4 65.4±11.7 | 46.5 48.8 | 7/43 29/45 |
| **Chen et al.(2013)** | Abdominal surgery | Dexmedetomidine NS | 63 63 | 66.2±7.5  67.9±6.6 | 44.1 50.8 | 9/63 17/63 |
| **Chen et al.(2015)** | Non-cardiac surgery | Dexmedetomidine NS | 87 61 | 70.6±4.2 71.4±4.9 | 35.6 37.7 | 8/87 13/61 |
| **Gao et al.(2021)** | Cardiac surgery | Dexmedetomidine NS | 20 20 | 70.5±4.1 71.4±4.5 | 45.0 50.0 | 2/20 8/20 |
| **Gong et al.(2018)** | Cardiac surgery | Dexmedetomidine NS | 40 40 | 42.3±1.6 42.4±1.5 | 30.0 33.0 | 1/40 10/40 |
| **Li et al.(2015)** | Abdominal surgery | Dexmedetomidine NS | 60 60 | 69.0±5.0 70.0±6.0 | 44.0 48.0 | 10/50 21/50 |
| **Cheng et al.(2021)** | Neurosurgery | Dexmedetomidine NS | 90 30 | 71.9±4.1 73.4±5.1 | 43.3 43.3 | 17/90 12/30 |
| **He et al.(2020)** | Abdominal surgery | Dexmedetomidine Ropivacaine NS | 24 24 24 | 69.6±4.4 69.3±4.4 68.6±3.9 | 37.5 41.7 45.8 | 5/24 7/24 7/24 |
| **Zhang et al.(2021)** | Non-cardiac surgery | Dexmedetomidine NS | 87 87 | 70.6±4.2 71.4±4.9 | 36.8 21.8 | 13/87 8/87 |
| **Wang et al.(2020)** | Abdominal surgery | Dexmedetomidine NS | 60 50 | 68.4±3.3 68.3±2.1 | 36.7 40.0 | 12/60 20/50 |
| **Xu et al.(2017)** | Abdominal surgery | Dexmedetomidine NS | 48 48 | 71.9±31.4 72.1±32.2 | N/A  N/A | 3/48 10/48 |
| **He et al.(2019)** | Abdominal surgery | Sufentanil  Fentanyl | 48  48 | 69 ± 2.1  70 ± 3.1 | 50.0  52.10 | 3/48  11/48 |
| **Wu et al.(2024)** | Orthopedic surgery | Remimazolam Midazolam | 35 35 | 72.2±4.4 72.0±4.9 | 51.4 57.1 | 0/35 2/35 |
| **Sun et al.(2024)** | Abdominal surgery | Dexmedetomidine NS | 40 40 | 68.2±2.2 68.0±1.9 | 52.5 50.0 | 5/40 14/40 |
| **Varsha et al. (2024)** | Cardiac surgery | Propofol Sevoflurane | 36 36 | 59.3±6.9 59.5± 10.1 | 11.1 19.4 | 11/36 20/36 |
| **Zhou et al.(2019)** | Cardiac surgery | Ulinastatin Dexmedetomidine NS | 39 39 38 | 70.6±4.4 69.8±5.1 70.0±4.9 | 56.4 52.8 52.6 | 7/39 6/38 12/38 |
| **Wang et al.(2017)** | Thoracic surgery | Ulinastatin NS | 38 37 | 66.0±6.0 67.0±5.0 | 7.9 10.8 | 9/38 17/37 |
| **Mansouri et al.(2019)** | Ophthalmic Surgery | Dexmedetomidine Midazolam NS | 50 50 50 | 66.5±1.6 63.6±8.3 64.0±7.3 | 50.0 50.0 62.0 | 6/50 4/50 10/50 |
| **Livia et al.(2016)** | Mixed Major Surgeries | Dexamethasone NS | 68 72 | 68.6±6.0 67.9±6.4 | 66.2 55.6 | 14/68 21/72 |
| **Zhang et al.(2019)** | Abdominal surgery | Dexmedetomidine NS | 80 60 | 73.8±14.5 74.1±13.9 | 36.2 33.3 | 0/80 8/60 |
| **Yang et al.(2015)** | ENT Surgery | Butorphanol  Fentanyl NS | 65 65  65 | 72.0± 4.0  73.0± 4.0 72.0±5.0 | 0.0 0.0  0.0 | 9 / 65  16/65 17/65 |
| **Kim et al.(2022)** | Orthopedic surgery | Dexmedetomidine NS | 40 40 | 65.0±5.9 66.3±6.3 | 42.5 55.3 | 9/40 9/38 |
| **Tanaka et al.(2017)** | Orthopedic surgery | Desflurane Propofol | 45 45 | 69.8±1.2 70.6±1.4 | 44.4 66.7 | 26/40 19/39 |
| **Zhao et al.(2020)** | Non-cardiac surgery | Dexmedetomidine Sufentanil | 315 101 | 69.6±4.2 69.2±4.1 | 42.6 44.1 | 40/315 30/101 |
| **Qiao et al.(2023)** | ENT Surgery | Desflurane propofol | 31 32 | 66.7±7.2 63.9±5.3 | 0.0 6.3 | 3/31 1/32 |
| **Li et al.（2021）** | Abdominal surgery | Propofol Sevoflurane | 272 272 | 64.0±4.4  65.0±5.2 | 25.2 34.4 | 38/226 46/221 |
| **Deepak et al.(2013)** | Non-cardiac surgery | Desflurane Sevoflurane | 30 30 | 69.2±4.7  69.5±4.4 | 60.0 36.7 | 0/30 1/30 |
| **Zhu et al.(2024)** | Orthopedic surgery | Ropivacaine Sufentanil | 30 30 | 73.9±5.7 75.2±6.6 | 56.7 46.7 | 2/30 9/30 |
| **Rörtgen et al.(2010)** | Non-cardiac surgery | Desflurane Sevoflurane | 40 40 | 69.0±2.9 69.2±3.0 | 50.0 45.0 | 11/27 14/30 |
| **Rohan et al.(2005)** | Urological/Gynecological Surgery | propofol Sevoflurane | 15 15 | 72.9±13.3 73.8±14.1 | 20.0 27.0 | 7/15 7/15 |
| **Mohamed et al. (2014)** | Abdominal surgery | Dexmedetomidine NS | 25 25 | 63.9±5.0 67.8±5.4 | 20.0 0.0 | 2/25 10/25 |
| **Zhang et al.(2018)** | Orthopedic surgery | Propofol Sevoflurane | 195 192 | 72.8±5.5 72.4±5.6 | 30.8 33.3 | 28/189 44/190 |
| **Min et al.(2023)** | Abdominal surgery | Remimazolam  Dexmedetomidine  NS | 34  35  35 | 70.1±3.6  71.3±3.6  69.7±2.5 | 38.2  42.9  40.0 | 3/34  3/35  10/35 |
| **Zhang et al.(2015)** | Abdominal surgery | Dexmedetomidine  NS | 20 20 | N/A N/A | 15.0 15.0 | 1/20 4/20 |
| **Ottens et al.(2014)** | Cardiac surgery | Dexamethasone NS | 140 138 | 63.4±12.3 65.4±11.5 | 26.6 21.0 | 19/140 10/138 |
| **Xu et al.(2013)** | Abdominal surgery | Ulinastatin NS | 40 40 | 75.6±7.2 74.1±8.1 | 47.5 45.0 | 1/40 11/40 |
| **Wang et al.(2018)** | Orthopedic surgery | Ulinastatin NS | 30 30 | 71.3±5.0 72.8±5.3 | 53.3 60.0 | 5/30 13/30 |
| **Zhu et al.(2016)** | Orthopedic surgery | Parecoxib NS | 60 65 | 75.1±8.2 74.3±7.6 | N/A N/A | 10/60 22/65 |
| **Hashemi et al.(2013)** | Urological or Orthopedic Surgery | Lidocaine NS | 35 35 | 66.0±1.0 67.0±2.0 | 22.9 20.0 | 10/35 9/35 |
| **Wang et al.(2002)** | Cardiac surgery | Lidocaine NS | 43 45 | 57.8±9.7 59.3±9.4 | 2.3 2.2 | 8/43 18/45 |
| **Liu et al.(2025)** | Colorectal surgery | Lidocaine  NS | 154  149 | 61.7±9.8  63.4±10.2 | 52.6  55.0 | 9/154  42/149 |
| **Tian et al.（2015）** | Orthopaedic surgery | Parecoxib  NS | 35  35 | 72.5±4.5  71.8±5.1 | 40.0  45.7 | 10/35  19/35 |
| **Wen et al. (2024)** | Mixed Major Surgeries | Butorphanol  Sufentanil | 52  52 | 71.0 ± 3.7  71.5 ± 4.0 | 46.2  38.5 | 6/52  17/52 |
| **Zhu et al. (2021)** | Abdominal Surgery | Ropivacaine  NS | 30  29 | 71.2 ± 5.3  71.2 ± 5.2 | 26.7  34.5 | 2 / 30  8 / 29 |
| **Kletecka et al. (2019)** | Neurosurgery | Propofol  Sevoflurane | 20  23 | 44.8 ± 10.8  43.6 ± 13.2 | 45  48 | 4/20  4/23 |
| **Kuzminskaite et al.(2023)** | Thyroid surgery | Desflurane  Sevoflurane | 48  34 | 52.5 ± 10.3  52.0 ± 13.5 | 81.3  85.3 | 1/48  0/34 |

**2.2 The trial registration information, design and intervention plan of included studies**

| **Study, Year [reference]Country,No. of centres, Registration No.** | **Drug** | **Inclusion criteria** | **Exclusion criteria** | **Interventions** |
| --- | --- | --- | --- | --- |
| Chawdhary et al.(2020) India single-centre No registration reported | Dexmedetomidine Propofol | 1. Patients aged 55 years or older 2. With at least a secondary level of education 3. Scheduled for elective non - cardiac surgery 4. American Society of Anesthesiologists (ASA) status I - III 5. Duration of surgery of at least 120 minutes | 1. Preoperative Mini - Mental State Examination (MMSE) score of 23 or less 2. Any cognitive impairment 3. Substance abuse 4. Cardiac comorbidity 5. History of neurological deficits (stroke or seizures) 6. Inability to comprehend neuropsychological tests (NPTs) 7. Significant hearing impairment 8. Language impairment 9. Visual impairment 10. Patient refusal | Dexmedetomidine group:  Loading Dose: A loading dose of 0.5–0.7 µg/kg of Dexmedetomidine was administered intravenously over a period of 10 minutes before the induction of anesthesia  Maintenance Infusion: Following the loading dose, a continuous infusion of Dexmedetomidine at a rate of 0.5–0.7 µg/(kg·h) was maintained until the end of the surgery  Administration Method:Intravenous  Propofol group:  Loading Dose: The control group received a loading dose of 2 mg/kg of Propofol intravenously before the induction of anesthesia  Maintenance Infusion: Following the loading dose, a continuous infusion of Propofol at a rate of 80–100 µg/(kg·min) was maintained until the end of the surgery  Administration Method:Intravenous |
| Chen et al.(2001)  USA  Multi-centre  No registration reported | Desflurane  Sevoflurane | 1. ASA physical status I–III  2. Age ≥65 years  3. Scheduled for total knee or hip replacement  4. Consenting elderly patients | 1. Clinically significant cardiovascular, respiratory, hepatic, renal, neurological, psychiatric, or metabolic diseases  2. Weight ≥50% above ideal body weight  3. General anesthesia within the previous seven days | Desflurane group:  Maintenance: Desflurane was administered via inhalation at an end-tidal concentration of 2%–4%, adjusted to maintain a BIS value of 55–65 and MAP within 20% of baseline. Administration Method: Inhalatio Sevoflurane group:  Maintenance: Sevoflurane was administered via inhalation at an end-tidal concentration of 1%–1.5%, adjusted to maintain a BIS value of 55–65 and MAP within 20% of baselin Administration Method: Inhalation. |
| Chen et al.(2013) China single-centre No registration reported | Dexmedetomidine NS | 1. Patients who had undergone laparoscopic cholecystectomy with general anesthesia 2. Age between 60 and 75 years 3. Patients who provided written informed consent | 1. Preoperative hypotension (mean arterial blood pressure <60 mmHg)  2. Preoperative bradycardia (heart rate <45 beats/min) 3. Central nervous system disease 4. History of mental illness, recent use of sedatives or analgesics, or impaired sensation 5. Refusal to participate in the study | Dexmedetomidine group： Loading Dose：A loading dose of 1 µg/kg of Dexmedetomidine (DEX) was administered intravenously over a period of 10 minutes before the induction of anesthesia. Maintenance Infusion：Following the loading dose, a continuous infusion of DEX at a rate of 0.4 µg/(kg·h) was maintained until the end of the surgery. Administration Method：intravenous Control group： Loading Dose：The control group received a placebo in the form of an equivalent volume of 0.9% NS solution. Infusion Rate：The saline was administered at the same volume and rate as the Dexmedetomidine in the experimental group. Administration Method：intravenous |
| Chen et al.(2015) China single-centre No registration reported | Dexmedetomidine NS | 1. Aged surgical patients who underwent time - selection surgery under general anesthesia (I - III grade according to the American Society of Anesthesiologists) 2. Patients with complete clinical documents 3. Patients who did not have a history of psychosis 4. Patients who were able to undergo the Mini - Mental State Examination (MMSE) before and after surgery | 1. Patients with a history of psychosis 2. Patients who did not have complete clinical documents 3. Patients who were unable to undergo the MMSE before or after surgery 4. Patients who did not undergo time - selection surgery under general anesthesia 5. Patients who had significant differences in age, sex, body weight, educational levels, or ASA grading compared to the other group | Dexmedetomidine Group: Loading Dose: A loading dose of 0.5 µg/kg of Dexmedetomidine (Dex) was administered intravenously over a period of 10 minutes before the induction of anesthesia Maintenance Infusion: Following the loading dose, a continuous infusion of Dex at a rate of 0.2 µg/(kg·h) was maintained until 30 minutes before the end of the surgery Administration Method: Intravenous Control Group: Loading Dose: The control group received a placebo in the form of an equivalent volume of 0.9% NS solution Infusion Rate: The saline was administered at the same volume and rate as the Dexmedetomidine in the experimental group Administration Method: Intravenous |
| Chen et al.( 2020) China single-centre No registration reported | Dexmedetomidine NS | 1. American Society of Anesthesiologists assessment I - II  2. Age at least 60 years  3. Mini - Mental State Examination (MMSE) score at least 25 points  4. Normal kidney and liver function  5. Voluntarily signing informed consent form | 1. History of hypertension, heart disease, and diabetes 2. History of cerebral hemorrhage, brain trauma, and cerebral infarction 3. Mental illness or mental abnormality to the point of being unable to cooperate with the researcher 4. Alcohol and drug abuse before inclusion in the study | Dexmedetomidine group： Loading Dose：A loading dose of 1 µg/kg of Dexmedetomidine (Dex) was administered intravenously over a period of more than 10 minutes before the induction of anesthesia. Maintenance Infusion：Following the loading dose, a continuous infusion of Dex at a rate of 0.3 µg/(kg·h) was maintained intraoperatively until the end of the surgery. Administration Method：intravenous Control group： Loading Dose：The control group received a placebo in the form of an equivalent volume of 0.9% NS solution. Infusion Rate：The saline was administered at the same volume and rate as the Dexmedetomidine in the experimental group. Administration Method：intravenous |
| Cheng et al.(2021) China single-centre No registration reported | Dexmedetomidine NS | 1. Patients aged over 65 years 2. ASA (American Society of Anesthesiologists) physical status classification of I - III 3. Scheduled for spine surgery | 1. Patients aged <65 or >90 years 2. Mini-Mental State Examination (MMSE) score less than 24 3. Presence of mental or neurological diseases that may affect the level of consciousness 4. Abnormalities in hepatic or renal function 5. Preoperative bradycardia or hypotension | Dexmedetomidine Group : Loading Dose: A loading dose of 0.3 µg/kg of Dexmedetomidine (Dex) was administered intravenously over a period of 10 minutes before the induction of anesthesia Maintenance Infusion: Following the loading dose, a continuous infusion of Dex at a rate of 0.5-0.8 µg/(kg·h) was maintained until 40 minutes before the end of the surgery Administration Method: Intravenous Control Group Loading Dose: The control group received a placebo in the form of an equivalent volume of 0.9% NS solution Infusion Rate: The saline was administered at the same volume and rate as the Dexmedetomidine in the experimental group Administration Method: Intravenous |
| Deepak et al.(2013) India single-centre No registration reported | Desflurane Sevoflurane | 1. Patients aged over 65 years  2. American Society of Anaesthesiology (ASA) physical status I, II, III  3. Undergoing surgeries under general anesthesia lasting from 45 minutes to 3 hours | 1. Patients who had general anesthesia within seven days prior to the procedure under study  2. Patients undergoing surgeries that required the Trendelenburg position  3. Patients with a history of neuropsychiatric disorders, alcohol consumption, or BMI > 30  4. Patients with clinically significant cardiovascular, respiratory, hepatic, renal, neurological, psychiatric, and metabolic diseases  5. Patients unable to read and write or with impaired hearing  6. Patients with pre-operative MMSE score < 23 | Desflurane Group： Anesthetic Induction ：Anesthesia was induced with fentanyl (1.02 mcg/kg IV) and propofol (1.0 - 2.0 mg/kg IV) until loss of response to oral commands. Anesthetic Maintenance ：After induction, desflurane 5% was administered through a vaporizer. Anesthesia was maintained with desflurane 2 - 6% in combination with nitrous oxide 66% in oxygen, corresponding to 1 - 1.8 MAC (age - adjusted MAC). Administration Method ：Inhalation. Sevoflurane Group： Anesthetic Induction ：Anesthesia was induced with fentanyl (1.02 mcg/kg IV) and propofol (1.0 - 2.0 mg/kg IV) until loss of response to oral commands. Anesthetic Maintenance ：After induction, sevoflurane 2% was administered through a vaporizer. Anesthesia was maintained with sevoflurane 1 - 1.5% in combination with nitrous oxide 66% in oxygen, corresponding to 1 - 1.8 MAC (age - adjusted MAC). Administration Method ：Inhalation. |
| Egawa et al.(2016) Japan single-centre UMIN 000002826 | Propofol sevoflurane | 1. Age 20-85 years, scheduled for elective lung surgery 2. American Society of Anesthesiologists physical status I-III 3. Fluency in Japanese, ability to read 4. Absence of serious hearing or visual impairments that would preclude neuropsychological testing | 1. interstitial lung disease or lung fibrosis  2. pregnancy or possibility of pregnancy  3. a history of neurological or mental illness  4. baseline Mini Mental State Examination (MMSE) score below 24  5. renal insufficiency (serum creatinine in excess of 1.5 mg dL-1)  6. active liver disease (aspartate aminotransferase in excess of 40 U dL-1)  7. documented coagulopathy (prothrombin time [15 sec, activated partial thromboplastin time [32 sec, or platelet count \150,000 lL-1) | Propofol group:  Loading Dose: An initial target-controlled infusion of propofol was administered with a target concentration of 3–4 µg/mL before the induction of anesthesia  Maintenance Infusion: Anesthesia was maintained with a continuous infusion of propofol, adjusting the target blood concentration to keep the BIS score within 40–60 throughout the surgery  Administration Method:Intravenous  Sevoflurane group:  Loading Dose: An initial bolus administration of propofol (1–2 mg/kg) was given before the induction of anesthesia, followed by a bolus administration of fentanyl (2.0–2.5 µg/kg)  Maintenance Infusion: Anesthesia was maintained with sevoflurane, adjusting the expired concentration to keep the BIS score within 40–60 throughout the surgery  Administration Method: Inhalation anesthesia combined with intravenous administration of fentanyl and epidural anesthesia |
| Fang et al.(2014) China single-centre No registration reported | Dexamethasone NS | Patients aged 40 to 60 years with facial spasm undergoing microvascular decompression (MVD) | 1. History of neurologic or mental disease  2. Renal failure  3. Active liver disease  4. Cardiac or pulmonary dysfunction  5. Endocrine, metabolic, or peptic ulcer disease  6. History of past surgery  7. Less than 6 years of schooling  8. Inability to complete neuropsychological testing  9. Visual dysfunction  10. Auditory dysfunction  11. Additional steroid medication required | Dexamethasone group：  Loading Dose: Intravenous injection of dexamethasone at 0.1-0.2 mg/kg, given before anesthesia induction.  Administration Method: Intravenous injection.  Control group：  Loading Dose: The control group received an equal volume of 0.9% NS as a placebo.  Infusion Rate: The infusion volume and rate of NS were the same as those of dexamethasone in the experimental group. |
| Gao et al.(2020) China single-centre No registration reported | Dexmedetomidine NS | 1. Patients aged 65 to 75 years old  2. Body weight between 55 and 85 kg  3. American Society of Anesthesiologists (ASA) physical status II or III  4. New York Heart Association (NYHA) functional class II or III  5. No history of previous acute myocardial infarction  6. No history of heart failure  7. No diabetes mellitus  8. No history of brain trauma, cerebrovascular accident, or mental illness  9. Left ventricular ejection fraction >40% | 1. Abnormal liver or kidney function 2. Use of sedatives or antidepressants 3. History of drug abuse 4. Presence of sick sinus syndrome | Dexmedetomidine Group:  Loading Dose: A loading dose of 1 µg/kg of Dexmedetomidine (Dex) was administered intravenously over a period of 15 minutes before the incision  Maintenance Infusion: Following the loading dose, a continuous infusion of Dex at a rate of 0.3–0.5 µg/(kg·h) was maintained until the end of the surgery  Administration Method: Intravenous  Control Group:  Loading Dose: The control group received a placebo in the form of an equivalent volume of 0.9% NS solution  Infusion Rate: The saline was administered at the same volume and rate as the Dexmedetomidine in the experimental group  Administration Method: Intravenous |
| Gao et al.(2021) China single-centre 2019 Medical Science Research Project of Hebei Provincial Health Commission (20190890) | Dexmedetomidine NS | 1. Patients aged 65-77 years 2. Weight of 56-75 kg 3. ASA (American Society of Anesthesiologists) physical status II or III 4. NYHA (New York Heart Association) functional class II or III | 1. Patients with a history of dementia or mental illness 2. Patients with severe cerebrovascular disease 3. Patients with a history of multiple surgeries 4. Patients with a history of severe systemic infection during the perioperative period 5. Patients with severe perioperative blood loss 6. Patients with severe acid-base balance or electrolyte disturbances 7. Patients with severe abnormal liver or kidney function 8. Patients with a history of acute myocardial infarction 9. Patients with a history of brain trauma or cerebrovascular accident 10. Patients with a history of diabetes. 11. Patients with a history of mental illness | Dexmedetomidine group： Loading Dose: A loading dose of 0.6 µg/kg of Dexmedetomidine (Dex) was administered intravenously over a period of 15 minutes before the induction of anesthesia Maintenance Infusion: Following the loading dose, a continuous infusion of Dex at a rate of 0.2 µg/(kg·h) was maintained until the end of the surgery Administration Method:intravenous Control group: Loading Dose: The control group received a placebo in the form of an equivalent volume of 0.9% NS solution Infusion Rate: The saline was administered at the same volume and rate as the Dexmedetomidine in the experimental group Administration Method:intravenous |
| Geng et al.(2017) China single-centre ChiCTR-OCC-11001411 | Propofol Sevoflurane Isoflurane | 1. Patients with an American Society of Anesthesiologists (ASA) score of II–III 2. Age ≥ 65 years 3. Sufficient level of education to be capable of completing neuropsychological tests | 1. History of allergy to anesthetics 2. Dialysis-dependent renal failure 3. Liver transaminase level ≤ 1.5 times the normal value 4. Mini-Mental State Examination score ≤ 26 5. Preexisting diagnosis of schizophrenia or dementia 6. Recent stroke 7. Known disorder affecting cognition 8. Mental dysfunction 9. History of cerebral surgery 10. Severe anxiety 11. Recent history of alcohol abuse 12. History of chronic opioid or other psychotropic drug use | Propofol Group:  Loading Dose: No specific loading dose mentioned.  Maintenance Infusion: A target-controlled infusion of propofol was initiated at a concentration of 3.0 µg/mL before intubation. After intubation, the concentration was maintained between 2.5–3.0 µg/mL throughout the surgery.  Administration Method: Intravenous.  Sevoflurane Group:  Loading Dose: No specific loading dose mentioned.  Maintenance Infusion: Sevoflurane was administered at a concentration of 1.0–1.5 minimum alveolar concentration (MAC) throughout the surgery.  Administration Method: Inhalation.  Isoflurane Group:  Loading Dose: No specific loading dose mentioned.  Maintenance Infusion: Isoflurane was administered at a concentration of 1.0–1.5 minimum alveolar concentration (MAC) throughout the surgery.  Administration Method: Inhalation. |
| Ghazaly et al.(2023) Egypt single-centre NCT05341154 | Dexmedetomidine Ketamine  NS | 1. Aged 60 years or older  2. American Society of Anesthesiologists (ASA) physical status I or II  3. Scheduled for emergency surgery (gastrointestinal, orthopedic, vascular, obstetric, urologic, or plastic surgery) | 1. Presence of preoperative delirium determined by the Delirium Observation Screening Scale (DOSS) 2. Preoperative cognitive impairment as measured by the Mini-Mental State Examination (MMSE) scores 3. History of intolerance to dexmedetomidine or Ketamine  4. Lack of cooperation or communication 5. Presence of Parkinson’s disease or epilepsy 6. High probability of postoperative care in the intensive care unit (ICU) | Dexmedetomidine Group：  Loading Dose: A single dose of 1 µg/kg dexmedetomidine was administered intravenously over a period of 10 minutes right before anesthesia induction  Maintenance Infusion: No continuous infusion was used in this study  Administration Method: Intravenous  Ketamine Group：  Loading Dose: A single dose of 1 mg/kg Ketamine was administered intravenously over a period of 10 minutes right before anesthesia induction  Maintenance Infusion: No continuous infusion was used in this study  Administration Method: Intravenous injection  Placebo Group：  Loading Dose: The control group received an equivalent volume of 0.9% NS solution intravenously over a period of 10 minutes right before anesthesia induction  Maintenance Infusion: No continuous infusion was used in this study  Administration Method: Intravenous injection |
| Glumac et al.(2017)  Croatia  single-centre  NCT02767713 | Dexamethasone  NS | 1. Aged 41–84 years 2. Scheduled for elective coronary artery bypass graft (CABG)  3. Scheduled for heart valve surgery  4. Scheduled for combined surgery (CABG and valve surgery)  5. With or without cardiopulmonary bypass (CPB) | 1. Cerebrovascular incident in the last 3 years  2. Mental illness  3. Visual, hearing, or motor impairment affecting cognitive assessment  4. Previous cardiac or carotid surgery  5. Left-ventricular ejection fraction <35%  6. Adrenal gland disease requiring steroid treatment for >7 days in the past year  7. Alcohol (>50 g/day or >500 g/week) or controlled substance abuse  8. Preoperative MMSE score <26 points  9. Preoperative BDI-II score >19 points  10. Preoperative CRP level >5 mg/L  11. Preoperative WBC count <4 or >10 × 10^9/L  12. Perioperative stroke  13. Additional corticosteroid treatment during the study period | Dexamethasone group:  Loading Dose: A single intravenous bolus of 0.1 mg/kg dexamethasone was administered 10 hours before the surgery.  Maintenance Infusion: The single bolus dose was sufficient for the study protocol.  Administration Method: Intravenous injection.  Placebo group:  Loading Dose: The placebo group received a single intravenous bolus of an equivalent volume of 0.9% sodium chloride (NS) solution 10 hours before the surgery  Infusion Rate: The single bolus of placebo was administered similarly to the dexamethasone group.  Administration Method: Intravenous injection. |
| Gong et al.(2018) China single-centre No registration reported | Dexmedetomidine NS | 1.Patients undergoing general anesthesia  2.Extracorporeal coronary artery bypass surgery  3.At the People's Hospital of Guangxi Zhuang Autonomous Region  4.Diagnosed preoperatively  5.Through imaging  6.Clinical manifestations  7.Direct observations during surgery | 1. Patients with cognitive impairment 2. Patients with mental and psychological illnesses 3. Chronic alcoholics 4. Drug addicts 5. Those who abuse psychotropic substances 6. Patients with illiterate education 7. Those with intraoperative use of epinephrine and norepinephrine 8. Patients allergic to drugs used in this study | Dexmedetomidine group： Loading Dose: A loading dose of 1 µg/kg of Dexmedetomidine (Dex) was administered intravenously over a period of 10 minutes before the induction of anesthesia. Maintenance Infusion: Following the loading dose, a continuous infusion of Dex at a rate of 0.2 µg/kg was maintained until the end of the surgery. Administration Method: intravenous. Control group： Loading Dose: The control group received a placebo in the form of an equivalent volume of 0.9% NS solution. Infusion Rate: The saline was administered at the same volume and rate as the Dexmedetomidine in the experimental group. Administration Method: intravenous. |
| Guo et al.(2020) China single-centre  ChiCTR-IOR-16009851 | Sevoflurane Propofol | 1. Patients aged 65 to 86 years undergoing tumor resection 2. Expected surgery duration of more than 2 hours and post-operative hospitalization for at least 7 days  3. American Society of Anesthesiologists (ASA) grade I, II, or III 4. Elective tumor resection under general anesthesia  5. Fluent in Chinese (speaking and reading) 6. Able to independently complete neuropsychological tests | 1. Refusal to participate in the study 2. Mini-Mental State Examination (MMSE) score ≤23 at screening 3. History of neurosurgery or cardiosurgery  4. Use of tranquillizers or antidepressants  5. Severe anxiety disorder or serious hearing and visual decline 6. Severe hepatic dysfunction (Child-Pugh stage C) or renal dysfunction (requiring renal replacement therapy)  7. Parkinson’s disease, Alzheimer’s disease, or coma  8. Alcoholism or drug dependence  9. Tumor metastasis or cancer cachexia  10. Cancelled surgery | Sevoflurane Group:  Anesthetic Induction:Induction was performed with etomidate, sufentanil, and rocuronium. No premedication was administered  Anesthetic Maintenance:Sevoflurane was used for anesthesia maintenance in Group S.Sufentanil was used for analgesia.Rocuronium was administered to maintain muscle relaxation  Administration Method: Inhalational (sevoflurane) and intravenous (sufentanil and rocuronium)  Propofol Group:  Anesthetic Induction:Induction was performed with etomidate, sufentanil, and rocuronium. No premedication was administered  Anesthetic Maintenance:Propofol was used for anesthesia maintenance in Group P.Sufentanil was used for analgesia.Rocuronium was administered to maintain muscle relaxation  Administration Method: Intravenous (propofol, sufentanil, and rocuronium) |
| Hashemi et al.(2013)  Iran  single-centre  No registration reported | Lidocaine NS | 1.Age > 65 years, undergoing urologic or orthopedic surgery. 2. ASA I–III. 3.No history of neurological/psychiatric disorders 4.No renal/hepatic dysfunction. 5.No alcohol/opioid/sedative/anticholinergic abuse. 6.no lidocaine allergy. | 1.Blood pressure variation > 20% from baseline. 2. severe bleeding requiring transfusion. 3. hemodynamic instability. | Lidocaine Group: Induction of anesthesia: Intravenous induction with 5 mg/kg sodium thiopental, 0.5 mg/kg atracurium, and 0.1 µg/kg fentanyl. Maintenance of anesthesia: Maintained with isoflurane (0.8% to 1.2%) in N₂O/O₂ (50%) and 0.1 mg/kg morphine. Reversal: At the end of surgery, reversed with 0.02 mg/kg atropine and 0.04 mg/kg neostigmine. Intervention: Lidocaine (1.5 mg/kg) was administered as an intravenous bolus 1 to 2 minutes before tracheal extubation. Postoperative intervention: None. Administration method: Intravenous bolus. Control Group: Induction of anesthesia: Intravenous induction with 5 mg/kg sodium thiopental, 0.5 mg/kg atracurium, and 0.1 µg/kg fentanyl. Maintenance of anesthesia: Maintained with isoflurane (0.8% to 1.2%) in N₂O/O₂ (50%) and 0.1 mg/kg morphine. Reversal: At the end of surgery, reversed with 0.02 mg/kg atropine and 0.04 mg/kg neostigmine. Intervention: Placebo (NS of the same volume) was administered as an intravenous bolus 1 to 2 minutes before tracheal extubation. Postoperative intervention: None. Administration method: Intravenous bolus. |
| He et al.(2019) China single-centre No registration reported | Sufentanil Fentanyl | 1. Age≥65 years old 2. at ASA levels I–II prior to the surgery 3. normal preoperative heart, lung, liver and kidney functions and basically normal conscious functions | 1. Allergic to aspirin or parecoxib sodium 2. Respiratory depression, bronchial asthma, peptic ulcer, heart disease, history of severe hypertension 3. Coagulation and platelet abnormalities, tendency to bleeding or history of blood system diseases 4. Severe hepatorenal dysfunction 5. Emergent craniocerebral trauma, preoperative consciousness or cognitive dysfunction 6. Gynecological disorders and abnormal hemoglobin (Hb) | Sufentanil group： Loading Dose：A loading dose of 0.4 µg/kg of Sufentanil was administered intravenously before the induction of anesthesia. Maintenance Infusion：No continuous infusion was maintained after the initial dose. Administration Method：Intravenous Fentanyl group： Loading Dose：A loading dose of 4 µg/kg of Fentanyl was administered intravenously before the induction of anesthesia. Maintenance Infusion：No continuous infusion was maintained after the initial dose. Administration Method：Intravenous |
| He et al.(2020) China single-centre ChiCTR1900021176 | Dexmedetomidine Ropivacaine NS | 1.Patients aged 65 years or older 2.Diagnosed with colorectal cancer and scheduled for radical resection between December 2018 and April 2019 at Fudan University Shanghai Cancer Center 3.American Society of Anesthesiologists (ASA) physical status scores ranging from I to II 4.Mini-Mental State Examination (MMSE) score ≥23 | 1.Presence of coagulation disorders or other severe diseases (e.g., severe cardiac, liver, or renal diseases) 2.Presence of distant metastasis or other cancers 3.Presence of other gastrointestinal diseases (e.g., esophageal regurgitation or peptic ulcer) 4.Use of certain drugs (e.g., acesodyne, sedatives, or antidepressant drugs) within 2 weeks before the study | Dexmedetomidine Group: Loading Dose: A loading dose of 0.5 µg/kg of Dexmedetomidine (Dex) was administered intravenously over a period of 15 minutes during surgery Maintenance Infusion: Following the loading dose, a continuous infusion of Dex at a rate of 0.6 µg/(kg·h) was maintained from the induction of anesthesia until 30 minutes before the end of surgery Administration Method: Intravenous Ropivacaine Group: Loading Dose: Epidural blockade was performed by intermittent epidural injection of 0.25% ropivacaine. A test dose of 3 mL of 2% lidocaine was injected before anesthesia induction Maintenance Infusion: Boluses of 5 mL of 0.25% ropivacaine were administered at 50-minute intervals during surgery, with a total volume of 14 to 18 mL Administration Method: Epidural injection Control Group: Loading Dose: The control group received a loading dose of 0.9% NS at a volume of 5 µL/kg intravenously over 15 minutes during surgery Maintenance Infusion: A continuous infusion of 0.9% saline at a rate of 6 µL/kg/h was maintained from anesthesia induction until 30 minutes before the end of surgery Administration Method: Intravenous |
| Hollinger et al.(2021) Switzerland Multi-centre  NCT02433041 | Haloperidol  Ketamine  NS | 1.Age: ≥65 years  2.Surgery type: Scheduled for elective or emergency surgery under general or combined (general + regional) anaesthesia  3.Surgical disciplines: Visceral, orthopaedic, vascular, gynaecological, cardiac, or thoracic surgery | 1. Pre-existing delirium or delirium upon hospital admission  2. MMSE score <24  3. DOS (Delirium Observation Scale) score ≥3  4. Dementia or cognitive impairment  5. High risk for postoperative ICU treatment (as per standard exclusion)  6. Known intolerance to haloperidol or Ketamine  7. QTc prolongation: ≥460 ms (men) or ≥470 ms (women), or use of drugs affecting QT interval  8. Neurological/psychiatric conditions: Parkinson’s disease, parkinsonism, use of dopaminergic drugs, epilepsy  9. Communication barriers: Aphasia, coma, terminal illness, drug/alcohol abuse  10. Surgery delay >72 hours after indication  11. Body weight >100 kg  12. Inability to read German | Haloperidol Group:  Loading Dose:A loading dose of 5 µg/kg body weight (BW) of Haloperidol was administered before the induction of anesthesia  Maintenance Infusion:No continuous infusion was maintained after the loading dose  Administration Method:Intravenous  Ketamine Group:  Loading Dose:A loading dose of 1 mg/kg BW of Ketamine was administered before the induction of anesthesia  Maintenance Infusion:No continuous infusion was maintained after the loading dose  Administration Method:Intravenous  Placebo Group:  Loading Dose:The placebo group received a placebo in the form of an equivalent volume of 0.9% NS solution  Infusion Rate:The saline was administered at the same volume and rate as the combined Haloperidol and Ketamine in the experimental group  Administration Method:Intravenous |
| Kim et al.(2022) South Korea single-centre NCT02643017 | Dexmedetomidine NS | 1. Patients aged over 60 years 2. Scheduled for arthroscopic shoulder surgery in the beach chair position 3. American Society of Anesthesiologists (ASA) physical status I to III 4. Capable of undergoing perioperative neurologic examination | 1. Severe pulmonary disease 2. Heart failure (New York Heart Association class > II) 3. Atherosclerotic carotid stenosis 4. Ischemic cerebrovascular disease 5. Psychotic disorder 6. Alcohol abuse 7. Dementia 8. Previous cognitive dysfunction 9. Severe functional liver or kidney disease 10. Uncontrolled diabetes mellitus 11. Korean version of the Mini - Mental State Examination (MMSE - K) score < 23 | Dexmedetomidine group: Loading Dose: No loading dose was administered. Maintenance Infusion: Dexmedetomidine (0.6 µg/kg/h) was administered intravenously throughout the surgery. Administration Method: Intravenous infusion. Control group: Loading Dose: No loading dose was administered. Maintenance Infusion: An equivalent volume of NS was administered at the same rate as the Dexmedetomidine group (0.6 µg/kg/h equivalent volume). Administration Method: Intravenous infusion. |
| Kletecka et al. (2019) Czech Republic single-centre ACTRN12613000362796 | Propofol Sevoflurane | 1. Age 18–65 years  2. Scheduled for single-level lumbar disc herniation surgery  3. Native Czech speaker  4. Normal preoperative neuropsychological examination  5. Normal preoperative electrophysiological examination  6. Signed informed consent form | 1. Non-native speakers of Czech  2. Age less than 18 or greater than 65  3. History of central nervous system diseases  4. Previous cognitive impairment  5. Use of psychotropic drugs  6. Mental illness, addiction, obstructive sleep apnea  7. Sensory impairment affecting the test | Propofol group:  Loading Dose: 1 mg/kg propofol, single intravenous bolus for induction.  Maintenance: Continuous infusion following the Roberts "10-8-6" protocol, adjusted to maintain BIS 45–60.  Administration Method: Intravenous bolus followed by continuous infusion.  Sevoflurane group:  Loading Dose: 2 mg/kg propofol, single intravenous bolus for induction.  Maintenance: Sevoflurane at 1.0 MAC, inhaled to maintain BIS 45–60.  Administration Method: Intravenous bolus for induction, then inhalational. |
| Kurup et al.(2023) India single-centre CTRI/2018/08/015358 | Dexmedetomidine Lidocaine | 1. Patients aged 60 - 80 years 2. ASA physical status I or II 3. Undergoing open abdominal surgery under general anesthesia 4. With an expected duration of more than 2 hours 5. Without a history of substance abuse 6. Without any disorder affecting cognition | 1. Preoperative Mini Mental State Examination Score below 24  2. Electrolyte imbalance  3. Bradycardia (heart rate below 45 beats per minute)  4. History of alcohol or substance abuse  5. Seizures  6. Cerebrovascular accidents or intracranial surgeries  7. Psychotic disorders  8. Dementia  9. Any disorder affecting cognition and higher mental functions  10. Diseases associated with systemic or central nervous system inflammation  11. Patients with difficulty in hearing/speech/unable to read even with assisted vision  12. Refractory intraoperative hypotension (mean arterial blood pressure below 60 mmHg) causing discontinuation of intervention agent | Dexmedetomidine group：  Loading Dose: A loading dose of 0.5 µg/kg of Dexmedetomidine was administered intravenously over a period of 10 minutes before the induction of anesthesia.  Maintenance Infusion: Following the loading dose, a continuous infusion of Dexmedetomidine at a rate of 0.5 µg/(kg·h) was maintained until the end of the surgery.  Administration Method: Intravenous.  Lidocaine group：  Loading Dose: A loading dose of 1 mg/kg of Lidocaine was administered intravenously over a period of 10 minutes before the induction of anesthesia.  Maintenance Infusion: Following the loading dose, a continuous infusion of Lidocaine at a rate of 1.5 mg/(kg·h) was maintained until the end of the surgery.  Administration Method: Intravenous. |
| Kuzminskaite et al.(2023) Lithuania Single-centre No registration reported | Desflurane Sevoflurane | 1. Age ≥ 40 yr  2. Elective thyroid surgery under general anaesthesia  3. Hospitalisation ≥ 1 day before surgery  4. Signed informed consent | 1. Refusal to participate  2. Unable to speak Lithuanian  3. Pre-existing neurological, psychiatric or cognitive disorder  4. Discharge < 24 h after surgery | Sevoflurane group:  Maintenance: sevoflurane inhalation to keep entropy 40–60; no loading dose  Administration: inhalation  Desflurane group:  Maintenance: desflurane inhalation to keep entropy 40–60; no loading dose  Administration: inhalation |
| Lee et al.(2015) South Korea single-centre No registration reported | Ketamine  NS | 1. Aged >60 years 2. ASA physical status 1-3 3. Scheduled for elective orthopedic surgery under general anesthesia 4. Anticipated anesthesia duration >2 hours | 1. Alcohol abuse or drug dependence 2. Central nervous system-related diseases (including dementia) 3. History of cerebrovascular accident within previous three years 4. Inability to perform neurocognitive function tests 5. Medication with tranquilizers or antidepressants 6. Severe visual or auditory dysfunction | Ketamine Group:  Loading Dose: A loading dose of 0.5 mg/kg of Ketamine was administered intravenously over a period of 3 mL mixed with 0.9% NS before the induction of anesthesia  Maintenance Infusion: No continuous infusion was maintained after the loading dose  Administration Method:Intravenous  Control Group (NS Group):  Loading Dose: The control group received a placebo in the form of an equivalent volume of 0.9% NS solution (3 mL)  Infusion Rate: The saline was administered at the same volume as the Ketamine in the experimental group  Administration Method:Intravenous |
| Li et al.(2015) China single-centre No registration reported | Dexmedetomidine NS | 1. American Society of Anesthesiology (ASA) status I - III 2. Aged > 60 years old 3. Scheduled for elective laparoscopic cholecystectomy (LC) under intravenous general anesthesia | 1. Aged <60 or >75 years 2. Patients with accompanying medical conditions that may affect the level of consciousness such as stroke stupor or dementia or patients with abnormalities in hepatic or renal function 3. Patients suffering from preoperative bradycardia heart rate HR <45 bpm or hypotension mean arterial blood pressure MAP <60 mmHg 4. Patients who had recently received a sedative or opioid drug 5. Patients with a Mini Mental State Examination MMSE score <24 | Dexmedetomidine Group: Loading Dose: A loading dose of 0.5 µg/kg of Dexmedetomidine (Dex) was administered intravenously over a period of 15 minutes during surgery. Maintenance Infusion: Following the loading dose, a continuous infusion of Dex at a rate of 0.6 µg/(kg·h) was maintained from the induction of anesthesia until 30 minutes before the end of surgery. Administration Method: Intravenous. Control Group: Loading Dose: The control group received a loading dose of 0.9% NS at a volume of 5 µL/kg intravenously over 15 minutes during surgery. Maintenance Infusion: A continuous infusion of 0.9% saline at a rate of 6 µL/kg/h was maintained from anesthesia induction until 30 minutes before the end of surgery. Administration Method: Intravenous. |
| Li et al.(2019) China single-centre No registration reported | Dexmedetomidine Propofol Midazolam | 1. Patients aged 65 years or older 2. Scheduled to undergo elective unilateral total hip replacement surgery or total knee replacement surgery at China-Japan Friendship Hospital 3. No significant contraindications to spinal anesthesia (e.g., aortic stenosis, coagulopathy, concurrent use of anticoagulants, spinal cord disease, or refusal of spinal anesthesia) 4. No severe hepatic or renal insufficiency 5. No history of stroke or transient ischemic attack within the past month 6. ASA grade not higher than III 7. No anticipated difficulty with neuropsychological assessment (e.g., receiving less than 9 years of education or having an existing mental disorder) | 1. Severe hepatic or renal insufficiency 2. History of stroke or transient ischemic attack within the past month 3. ASA grade higher than III 4. Anticipated difficulty with neuropsychological assessment (e.g., receiving less than 9 years of education or having an existing mental disorder) 5. Contraindications to spinal anesthesia (e.g., aortic stenosis, coagulopathy, concurrent use of anticoagulants, spinal cord disease, or refusal of spinal anesthesia) | Dexmedetomidine Group:  Loading Dose: A loading dose of 0.6 µg/kg of Dexmedetomidine (Dex) was administered intravenously over a period of 15 minutes before the induction of anesthesia  Maintenance Infusion: Following the loading dose, a continuous infusion of Dex at a rate of 0.2 µg/(kg·h) was maintained until the end of the surgery  Administration Method: Intravenous  Propofol Group:  Loading Dose: A loading dose of 1.0 mg/kg of Propofol was administered intravenously over a period of 10 minutes before the induction of anesthesia  Maintenance Infusion: Following the loading dose, adjusted to achieve a bispectral index (BIS) score between 70 and 85  Administration Method: Intravenous  Midazolam Group:  Loading Dose: A loading dose of 0.05 mg/kg of Midazolam was administered intravenously over a period of 5 minutes before the induction of anesthesia  Maintenance Infusion: Bolus doses of 0.02–0.03 mg/kg were administered as needed to maintain a BIS score between 70 and 85  Administration Method: Intravenous |
| Li et al.（2021） China Multi-centre NCT01809041 | Propofol Sevoflurane | 1. Age 60 years or older  2. Scheduled for elective major laparoscopic abdominal surgery (e.g., gastrointestinal and gynecologic surgery) with an expected duration of 2 hours or longer  3. No severe hearing and vision impairment and able to read | 1. Severe diseases in cardiovascular, respiratory, liver, kidney, or central nervous systems with a life span of less than 3 months  2. Mini-mental status examination score of less than 23  3. History of dementia or psychiatric illness  4. Current use of sedatives, antidepressants, or corticosteroids  5. Alcoholism and drug dependence  6. Previous inclusion in this study  7. Difficulty with follow-up or poor compliance  8. Uncontrolled hypertension (more than 180/100 mmHg) | Propofol group： Loading Dose: Not applicable Maintenance Infusion: Anesthesia was maintained with intravenous propofol infusion (50 to 150 µg · kg–1 · min–1) and remifentanil infusion (0.1 to 0.5 µg · kg–1 · min–1). Administration Method: Intravenous Sevoflurane group： Loading Dose: Not applicable Maintenance Infusion: Anesthesia was maintained with inhaled sevoflurane (1.0 to 1.5 minimum alveolar concentrations) and intravenous remifentanil infusion (0.1 to 0.5 µg · kg–1 · min–1). Administration Method: Inhalation (for sevoflurane) and intravenous (for remifentanil) |
| Liu et al.(2020) China single-centre No registration reported | Dexmedetomidine NS | 1. Patients diagnosed with sleep apnea syndrome (SAS) 2. Aged between 18 and 65 years, with ASA levels I or II 3. Preoperative Mini - Mental State Exam (MMSE) score > 27 points 4. Poor efficacy after 3 months of conventional treatment and indications for UPPP 5. Complete clinical data, good treatment compliance, and informed consent | 1. Diseases in the mouth, nose, pharynx, or larynx 2. Severe hypertension, hepatic or renal dysfunction, cardiovascular or cerebrovascular diseases 3. Severe anxiety and depression 4. Allergy to dexmedetomidine 5. Use of drugs for cognitive disorders and psychotropic diseases | Dexmedetomidine group：  Loading Dose: A loading dose of 0.8 µg/kg of Dexmedetomidine (Dex) was administered intravenously over a period of 10 minutes after the anesthesia induction.  Maintenance Infusion: Following the loading dose, a continuous infusion of Dex at a rate of 0.2 µg/(kg·h) was maintained until approximately 10 minutes before the end of the surgery.  Administration Method: intravenous  Control group：  Loading Dose: The control group received a placebo in the form of an equivalent volume of 0.9% NS solution.  Infusion Rate: The saline was administered at the same volume and rate as the Dexmedetomidine in the experimental group.  Administration Method: intravenous |
| Liu et al.(2025) China Multi-centre No registration reported | Lidocaine NS | 1. Elective laparoscopic radical resection for colorectal cancer;  2. Age 18–70 years;  3. BMI 18–30 kg m⁻²;  4. ASA I–III;  5. Expected surgery ≤4 h with propofol-based anesthesia;  6. Able to give written informed consent;  7. Not participating in another drug trial within 3 months. | 1. Hypersensitivity to lidocaine/amide local anaesthetics;  2. Severe hepatic, renal, cardiopulmonary, neurologic or hematologic disease;  3. Uncontrolled hyperthyroidism;  4. Alcohol or drug abuse;  5. Epilepsy, psychiatric disorder or antipsychotic medication;  6. Chronic opioid or lidocaine use;  7. Emergency surgery;  8. Pre-existing cognitive dysfunction;  9. Any condition judged unsuitable by investigators. | Lidocaine Group: Pre-induction bolus 2 mg kg⁻¹ iv 30 min before anesthesia, followed by continuous infusion 2 mg kg⁻¹ h⁻¹ until skin closure. Control Group: Equal volume of 0.9% saline at identical time points. Administration method: intravenous bolus + continuous infusion. |
| Livia et al.(2016) Brazil single-centre NCT01332812 | Dexamethasone NS | 1.Patients aged 60 years or older scheduled for noncardiac and nonneurologic surgery 2.Surgery duration not exceeding 6 hours 3.No history of brain disease, dementia, or other psychiatric disorders affecting cognition 4.Literate 5.No preoperative continuous use of corticosteroids, antidepressants, or opioids MMSE score meeting the Brazilian population standard (18 points for those with up to 4 years of formal education and 23 points for higher education levels) | 1.MMSE score below the inclusion standard (18 or 23 points, depending on education level) 2.Other unspecified reasons (e.g., patient refusal) | Dexamethasone group：  Loading Dose: A loading dose of 8 mg of Dexamethasone was administered intravenously before the induction of anesthesia.  Maintenance Infusion: There was no continuous infusion of Dexamethasone after the loading dose.  Administration Method: intravenous  Control group：  Loading Dose: The control group received a placebo in the form of an equivalent volume of 0.9% NS solution.  Infusion Rate: The saline was administered at the same volume and rate as the Dexamethasone in the experimental group.  Administration Method: intravenous |
| Luo et al.(2024) China single-centre ChiCTR2100047067 | Ketamine  NS | 1. Adult patients 2. ASA physical status I-III 3. Scheduled elective resection of lung/mediastinal tumors 4. One-lung ventilation required | 1. Abnormal liver/kidney function 2. Drug abuse 3. HR >120 bpm 4. Allergy to study drugs 5. Preoperative neuropsychiatric disease/cognitive impairment 6. SBP >180 mmHg or DBP >110 mmHg 7. High-risk conditions for BP elevation (e.g., aortic dissection) | Ketamine Groups:  Dosing: A single intraoperative dose of 0.2-0.5mg/kg of EsKetamine was administered  Administration Method: Intravenous  Control Group:  Dosing: The control group received a placebo in the form of an equivalent volume of 0.9% NS solution  Administration Method: Intravenous |
| Ma et al.(2023) China single-centre ChiCTR2200059003 | Ketamine  NS | 1. Age ≥65 years  2. ASA physical status I–III  3. BMI 18–30 kg/m²  4. Elective major abdominal surgery for gastrointestinal tumors under general anesthesia  5. Operation time ≥2 hours | 1. Preoperative cognitive dysfunction (MMSE score below criteria) 2. Inability to communicate (aphasia, severe hearing impairment, etc.) 3. Use of psychotropic drugs 4. History of alcohol abuse or acetylcholine drug use 5. Use of sevoflurane, dexmedetomidine, scopolamine, penehyclidine hydrochloride during study 6. Severe circulatory or respiratory diseases 7. History of cerebral hemorrhage 8. Coagulation abnormalities 9. Contraindications to esKetamine use 10. Severe subcutaneous emphysema or serious adverse events 11. Emergency surgery 12. Admitted to ICU postoperatively 13. Withdrawal during study period 14. Second operation within 3 postoperative days | EsKetamine Group:  Loading Dose: A loading dose of 0.25 mg/kg of EsKetamine was administered intravenously before the induction of anesthesia  Maintenance Infusion: Following the loading dose, a continuous infusion of EsKetamine at a rate of 0.125 mg/(kg·h) was maintained until 20 minutes before the end of the surgery  Administration Method: Intravenous  Control Group:  Loading Dose: The control group received a placebo in the form of an equivalent volume of 0.9% NS solution  Infusion Rate: The saline was administered at the same volume and rate as the EsKetamine in the experimental group  Administration Method: Intravenous |
| Mansouri et al.(2019)  Iran  single-centre No registration reported | Dexmedetomidine Midazolam NS | 1. Over 65 years old  2. ASA score of I or II  3. Candidates for cataract surgery  4. Patient consent to participate in the study  5. No history of sensitivity to anesthetics  6. No history of moderate to severe mental disorders | 1. Severe hemodynamic disorders during the surgery leading to a change in anesthesia  2. Extended surgery duration for >1 h  3. Patient’s refusal to complete the questionnaire after the surgery  4. Sensitivity to the examined anesthetics | Dexmedetomidine group： Loading Dose：No loading dose was mentioned in this study. Maintenance Infusion：A single intravenous dose of 1 µg/kg of Dexmedetomidine was administered simultaneously with the induction of general anesthesia. Administration Method：Intravenous Midazolam group： Loading Dose：No loading dose was mentioned in this study. Maintenance Infusion：A single intravenous dose of 0.1 mg/kg of Midazolam was administered simultaneously with the induction of general anesthesia. Administration Method：Intravenous Control group： Loading Dose：The control group received an equivalent volume of NS. Infusion Rate：The saline was administered at the same volume as the Dexmedetomidine and Midazolam in the experimental group. Administration Method：Intravenous |
| Meineake et al.(2014) USA single-centre NCT01199913 | Desflurane Sevoflurane | 1. Patients aged ≥65 years 2. Scheduled for elective surgery requiring general anesthesia with a duration of ≥120 minutes 3. American Society of Anesthesiologists (ASA) physical status classification of 2, 3, or 4 4. Patients who provided written informed consent | 1. Patients with clinically significant cardiovascular respiratory hepatic renal neurological psychiatric or metabolic disease  2. Patients with a history of cerebral vascular disease or dementia  3. Patients weighing more than 150% of their ideal body weight male ideal body weight in kg = 50 + 2.3 kg per inch >5 feet female ideal body weight in kg = 45.5 + 2.3 kg per inch >5 feet  4. Patients who did not speak English or had less than an elementary school education  5. Patients who had undergone general anesthesia within the past 7 days  6. Patients with an abnormal baseline Mini Mental Status Examination MMSE score  7. Patients who were unable to complete the cognitive function tests due to inability to perform postoperative MMSE or data capture failure  8. Patients who withdrew consent after surgery | Desflurane Group:  Loading Dose: No specific loading dose was mentioned for desflurane  Maintenance Infusion: Desflurane was administered via inhalation in oxygen and air for maintenance of anesthesia. The dose was adjusted to maintain moderate general anesthesia based on processed electroencephalography (Patient State Index 25 to 50)  Administration Method: Inhalation  Sevoflurane Group:  Loading Dose: No specific loading dose was mentioned for sevoflurane  Maintenance Infusion: Sevoflurane was administered via inhalation in oxygen and air for maintenance of anesthesiaThe dose was adjusted to maintain moderate general anesthesia based on processed electroencephalography (Patient State Index 25 to 50)  Administration Method: Inhalation |
| Min et al.(2023)  China  Single-centre  ChiCTR2300068036 | Remimazolam  Dexmedetomidine  NS | 1.Aged 65–80 years undergoing elective laparoscopic radical gastrectomy  2.ASA II–III  3.BMI 18–24 kg/m² | 1.Preoperative cognitive dysfunction (MMSE/MoCA cutoff by education)  2.History of cardiac surgery, CVA, alcoholism  3.Severe organ dysfunction  4.Coagulation disorders  5. Severe visual/hearing impairment  6.Psychiatric history or long-term sedative/antidepressant use | Remimazolam group : Induction of anesthesia: Intravenous induction with cisatracurium, sufentanil, and propofol. On this basis, remimazolam mesylate 0.2 mg/kg was added. Maintenance of anesthesia: Intravenous infusion of propofol + remifentanil. On this basis, intravenous pumping of remimazolam 0.3-0.5 mg/kg/h was added. BIS monitoring: BIS values were maintained between 40 and 60. Administration method: Intravenous infusion. Dexmedetomidine group : Induction of anesthesia: Intravenous induction with cisatracurium, sufentanil, and propofol. Before induction, dexmedetomidine 0.5 µg/kg was pumped over 10 min. Maintenance of anesthesia: Intravenous infusion of propofol + remifentanil. On this basis, intravenous pumping of dexmedetomidine 0.3-0.5 µg/kg/h was added. BIS monitoring: BIS values were maintained between 40 and 60. Administration method: Intravenous infusion. Control group: Induction of anesthesia: Intravenous induction with cisatracurium, sufentanil, and propofol. On this basis, saline 5 mL was added. Maintenance of anesthesia: Intravenous infusion of propofol + remifentanil. On this basis, intravenous pumping of saline 0.3-0.5 mL/kg/h was added. BIS monitoring: BIS values were maintained between 40 and 60. Administration method: Intravenous infusion. |
| Mohamed et al. (2014)  Saudi Arabia single-centre ACTRN126130003787 | Dexmedetomidine NS | 1. Patients aged ≥60 years  2. ASA physical status I–III  3. Scheduled for elective abdominal surgery expected to last >2 hours  4. Hospital stay >48 hours  5. Educable for neuropsychological testing | 1. Heart rate <60 bpm  2. Arrhythmias  3. Severe ventricular dysfunction (EF <35%)  4. Hypovolemia  5. Psychiatric disorders  6. Preexisting cognitive impairment  7. Alcohol or drug abuse  8. Preexisting CNS deficit or neurological disorders confirmed by MRI  9. Inability to follow protocol  10. Poor language comprehension  11. Severe visual or auditory impairment | Dexmedetomidine group: Loading Dose: A loading dose of 1 µg/kg of Dexmedetomidine was administered intravenously over a period of 10 minutes before the induction of anesthesia Maintenance Infusion: Following the loading dose, a continuous infusion of Dexmedetomidine at a rate of 0.4 µg/(kg·h) was maintained until the end of the surgery Administration Method:Intravenous Control group: Loading Dose: The control group received a placebo in the form of an equivalent volume of 0.9% NS solution Infusion Rate: The saline was administered at the same volume and rate as the Dexmedetomidine in the experimental group Administration Method:Intravenous |
| Mu et al.(2017) China Multi-centre  ChiCTR-TRC-10001101 | Parecoxib NS | 1. Age ≥60 years  2. Elective total hip or knee replacement surgery | 1. Preoperative delirium 2. American Society of Anesthesiologists (ASA) physical status classification ≥ IV 3. New York Heart Association (NYHA) functional classification ≥ III 4. Allergy to nonsteroid anti-inflammatory drugs (NSAIDs) 5. Severe renal dysfunction requiring dialysis 6. Severe hepatic dysfunction (Child-Pugh score ≥ 10) 7. History of previous coronary heart disease (unstable angina or myocardial infarction), cerebrovascular disease (transient ischemic accident or stroke), or peripheral arterial disease (stenosis or occlusion) 8. Active peptic ulcers or inflammatory bowel diseases | Parecoxib Group:  Loading Dose: A single intravenous dose of parecoxib sodium 40 mg was administered at the end of surgery  Maintenance Dosing: Subsequent doses of 40 mg parecoxib sodium were administered intravenously every 12 hours for a total of 3 days postoperatively  Administration Method: Intravenous  Control Group:  Loading Dose: The control group received a placebo in the form of an equivalent volume of 0.9% NS solution at the end of surgery  Maintenance Dosing: The saline placebo was administered at the same volume and frequency as the parecoxib in the experimental group (every 12 hours for a total of 3 days postoperatively)  Administration Method: Intravenous |
| Nagz et al.(2020) India Multi-centre NCT03793751 | Dexmedetomidine NS | 1. American society of Anesthesiology (ASA) status of I - III 2. Age between 60 - 75 years 3. Scheduled for elective hip surgery under spinal anesthesia | 1. Patient not willing to be a part of the study 2. Patients were aged <60 or >75 years 3. Patients with accompanying medical conditions that may affect the level of consciousness, such as stroke, stupor or dementia, or patients with abnormalities in hepatic or renal function, electrolyte imbalance 4. Patients suffering from preoperative bradycardia [heart rate (HR) <60 bpm] or hypotension [mean arterial blood pressure (MAP) <70 mmHg] 5. Patients who had recently received a sedative or opioid drug 6. Patients with a MoCA (Montreal Cognitive Assessment) score <26 7. Patients with persistent hypotension and bradycardia intra-operatively even after giving Mephentermine will be excluded from the study | Dexmedetomidine group：  Loading Dose: A loading dose of 1 µg/kg of Dexmedetomidine (Dex) was administered intravenously over a period of 10 minutes after spinal anesthesia and before the start of surgery.  Maintenance Infusion: Following the loading dose, a continuous infusion of Dex at a rate of 0.4 µg/(kg·h) was maintained until the end of the surgery.  Administration Method: Intravenous.  Control group：  Loading Dose: The control group received a placebo in the form of an equivalent volume of 0.9% NS solution.  Infusion Rate: The saline was administered at the same volume and rate as the Dexmedetomidine in the experimental group.  Administration Method: Intravenous. |
| Ottens et al.(2014)  Netherlands  Multi-centre  NCT00293592 | Dexamethasone NS | 1.Adult patients scheduled for cardiac surgery with cardiopulmonary bypass (CPB). | 1.Significant impairments of vision, hearing, or motor skills (e.g., hemiplegia). 2.Mental illness precluding the ability to complete the baseline cognitive assessment. 3.Patients who had already given informed consent but could not complete a baseline assessment. | Dexamethasone Group: Induction of anesthesia: Intravenous induction with cisatracurium, sufentanil, and propofol. On this basis, dexamethasone 1 mg/kg was administered as a single intravenous bolus shortly after induction of general anesthesia. Maintenance of anesthesia: Intravenous infusion of propofol + remifentanil. No additional specific maintenance related to dexamethasone was mentioned. BIS monitoring: BIS values were maintained between 40 and 60. Administration method: Intravenous bolus. Placebo Group: Induction of anesthesia: Intravenous induction with cisatracurium, sufentanil, and propofol. On this basis, placebo (0.9% NaCl) was administered as a single intravenous bolus shortly after induction of general anesthesia. Maintenance of anesthesia: Intravenous infusion of propofol + remifentanil. No additional specific maintenance related to placebo was mentioned. BIS monitoring: BIS values were maintained between 40 and 60. Administration method: Intravenous bolus. |
| Qiao et al.(2023) China single-centre NCT03194074 | Desflurane Propofol | 1. Age ≥ 65 years  2. American Society of Anesthesiologists (ASA) grade I or II  3. Scheduled for elective laser laryngeal surgery (LLS) | 1. Refusal to participate  2. Uncontrolled hypertension or diabetes  3. Untreated cardiac or pulmonary disease  4. Hepatic or renal failure  5. Epilepsy  6. Current medications potentially affecting the central nervous system  7. Preexisting mental status alterations  8. Preoperative mini - mental state examination (MMSE) score ≤ 23 | Desflurane group: Loading Dose: No specific loading dose was mentioned. Maintenance Infusion: Desflurane was administered with an end-tidal minimum alveolar concentration of 0.8~1.0. Administration Method: Inhalation Propofol group: Loading Dose: An infusion of propofol (2000 mg/h) was administered until the patient lost consciousness. Maintenance Infusion: Infusions of propofol (70–140 μg/kg/min) were administered throughout the procedure. Administration Method: Intravenous |
| Racman et al.(2023) Slovenia single-centre NCT03747432 | Dexmedetomidine Propofol | 1. Aged 18 years or older 2. Voluntary consent to participate in the study 3. Scheduled for TAVR via the transfemoral approach | 1. Age under 18 years 2. Pregnant or lactating women 3. Inability to perform MMSE or baseline MMSE score <25 points 4. Known allergy to local anesthetic lidocaine or study drugs 5. History of alcohol abuse 6. Diagnosed dementia of any origin 7. Psychiatric illness including mood and psychotic disorders 8. Cerebrovascular insult or non - vascular neurological disease 9. Decompensated liver disease 10. Chronic kidney failure on hemodialysis 11. Severe pulmonary disease (chronic obstructive pulmonary disease, asthma) 12. Surgical aortic valve replacement 13. Severe coronary artery disease 14. Presentation of acute myocardial infarction requiring simultaneous percutaneous coronary intervention 15. Patients within 4 weeks after acute myocardial infarction | Dexmedetomidine group:  Loading Dose: A loading dose of 0.5 µg/kg of Dexmedetomidine was administered intravenously over a period of 10 minutes before the start of the procedure.  Maintenance Infusion: Following the loading dose, a continuous infusion of Dexmedetomidine at a rate of 0.2-1.0 µg/(kg·h) was maintained until the end of the procedure.  Administration Method: Intravenous  Propofol group:  Loading Dose: No loading dose was administered.  Maintenance Infusion: A continuous infusion of Propofol at a rate of 0.5-2.5 mg/(kg·h) was maintained throughout the procedure.  Administration Method: Intravenous |
| Rohan et al.(2005) Ireland single-centre No registration reported | propofol Sevoflurane | 1. Elderly patients ≥ 65 years  2. Scheduled for minor urological (cystoscopy) or gynaecological (hysteroscopy) surgery  3. Under general anaesthesia  4. With an anticipated one - night postoperative stay | 1. Central nervous system disease  2. Pre - existing cognitive dysfunction (MMSE < 24)  3. Use of phenothiazines or antidepressants  4. History of cardiac or neurosurgery  5. Previous neuropsychological testing  6. Poor language comprehension  7. Alcoholism or drug dependence | Propofol group: Loading Dose: Not applicable. Propofol was administered as total intravenous anesthesia (TIVA) without a specific loading dose Maintenance Infusion: Propofol was delivered using a Diprifusor™ TCI system. The target concentrations of propofol were adjusted by the anesthesiologist to maintain adequate depth of anesthesia throughout the surgery Administration Method:Intravenous Sevoflurane group: Loading Dose: Not applicable. Sevoflurane was administered via inhalation without a specific loading dose Maintenance Infusion: Sevoflurane was delivered using an incremental dose, tidal volume inhalation induction technique. Once a satisfactory depth of anesthesia was reached, a laryngeal mask airway was positioned, and sevoflurane was maintained at a concentration that ensured adequate anesthesia until the end of the surgery Administration Method: Inhalation |
| Rörtgen et al.(2010) Germany single-centre No registration reported | Desflurane Sevoflurane | 1. Patients aged 65–75 yr  2. ASA physical status I–III  3. Scheduled for elective surgery (trauma, ENT, gynaecology, urology, or spinal neurosurgery)  4. With planned anaesthesia duration 60–180 min  5. Able to cooperate with neuropsychological testing  6. Who provided written informed consent | 1. Age > 75 yr  2. Chronic alcohol or drug abuse  3. Impaired renal or hepatic function  4. Diabetes mellitus  5. Disabling neuropsychiatric disorders  6. History of stroke, brain trauma, or cardiopulmonary resuscitation within the last 12 months  7. Increased intracranial pressure  8. Known allergy to volatile anaesthetics  9. Emergency surgery  10. History of myocardial infarction  11. Adrenal insufficiency  12. Congestive heart failure  13. Inability to cooperate  14. Legal incapacity | Desflurane group： Loading Dose：N/A. Maintenance Infusion：N/A. Administration Method:inhalation. The depth of anesthesia was controlled by adjusting desflurane to age - adapted equipotent minimum alveolar concentration (MAC) between 4.2 and 4.5 vol% in oxygen 30%. Sevoflurane group： Loading Dose：N/A. Maintenance Infusion：N/A. Administration Method：inhalation. The depth of anesthesia was controlled by adjusting sevoflurane to age - adapted equipotent minimum alveolar concentration (MAC) between 1.1 and 1.4 vol% in oxygen 30%. |
| Sun et al.(2024) China single-centre ChiCTR2300078598 | Dexmedetomidine NS | 1. Patients aged ≥65 years 2. ASA physical status I - III 3. Scheduled for ERCP under general anesthesia | 1. Lack of informed consent  2. Severe cardiovascular dysfunction  3. Severe respiratory dysfunction  4. Severe central nervous system dysfunction  5. Severe liver dysfunction  6. Severe kidney dysfunction  7. Glaucoma  8. Communication or assessment impairments  9. Suspected severe neurocognitive impairment (MMSE score <24)  10. Anticipated airway difficulties  11. Alcohol or drug dependence  12. Current use of sedatives  13. Current use of antidepressants  14. Current use of corticosteroids  15. Allergy to dexmedetomidine or other anesthetics  16. Baseline heart rate <50 bpm  17. Sick sinus syndrome or second-degree or higher atrioventricular block without a pacemaker  18. Previously enrolled | Dexmedetomidine group：  Loading Dose：No loading dose was administered.  Maintenance Infusion：Dexmedetomidine was administered at a rate of 0.4 µg/(kg·h) starting 15 minutes before surgery and continued until the end of the surgery.  Administration Method：Intravenous infusion.  Control group：  Loading Dose：No loading dose was administered.  Infusion Rate：The control group received an equivalent volume of 0.9% NS solution at the same rate as the Dexmedetomidine group.  Administration Method：Intravenous infusion. |
| Takazawa et al.(2023) Japan Multi-centre  UMIN000014620 | Minocycline NS | 1. Patients aged between 60 and 90 years  2. undergoing total knee arthroplasty  3. under general anesthesia  4. between January 2015 and October 2018  5. were included in the study | 1. Patients with American Society of Anesthesiologists (ASA) Physical Status III or higher 2. Patients with a history of mental disease, drug dependency, or alcoholism 3. Patients with liver or renal disorders that can affect the pharmacokinetics of minocycline 4. Patients unable to discontinue medication with possible interactive effects with minocycline, including digoxin, methotrexate, warfarin sodium, and sulfonylurea-based hypoglycemic drugs 5. Patients with dementia before surgery 6. Patients deemed unfit for study participation based on the physician’s judgment | Minocycline Group:  Loading Dose: No specific loading dose was administered  Maintenance Dosage: Patients received 100 mg of minocycline orally twice daily (morning and evening) starting from the day before surgery until the seventh day after surgery  Administration Method: Oral  Placebo Group:  Loading Dose: No specific loading dose was administered  Maintenance Dosage: Patients received a placebo capsule, identical in appearance to the minocycline capsule, orally twice daily (morning and evening) on the same schedule as the minocycline group  Administration Method: Oral. |
| Tanaka et al.(2017) USA single-centre NCT01270620 | Desflurane Propofol | 1. Age over 65 years  2. Undergoing elective total knee arthroplasty (TKA)  3. ASA physical status II or III  4. BMI ≥ 30 kg/m² | 1. Refusal or failure of regional block 2. Pre-existing neurocognitive disorders (MMSE score ≤ 23) 3. Known intolerance to any of the drugs used in the study | Desflurane group： Loading Dose: Not applicable. Maintenance Infusion: Desflurane was used for maintenance anesthesia. Administration Method: Inhalation. Propofol group： Loading Dose: Not applicable. Maintenance Infusion: Propofol was used for maintenance anesthesia. Administration Method: Intravenous. |
| Tian et al.（2015） China single-centre No registration reported | Parecoxib NS | 1. Age ≥ 65 y  2. ASA I–II  3. Elementary-school education completed  4. Pre-operative MMSE ≥ 21  5. Scheduled for total-hip replacement or femoral-head resurfacing  6. No neurological/psychiatric disease  7. No chronic use of tranquillisers or antidepressants  8. No alcohol- or drug-abuse history | 1. MMSE < 21  2. Neurological or psychiatric disorder  3. Chronic use of tranquillisers/antidepressants  4. Alcohol or drug dependence  5. Inability to co-operate with cognitive testing | Parecoxib Group:  Loading Dose: 40 mg parecoxib sodium diluted to 5 ml with normal saline, single intravenous bolus administered immediately before induction of general anaesthesia.  Administration Method: Intravenous bolus.  Control group:  Loading Dose: Equal volume (5 ml) of normal saline, single intravenous bolus at the same time-point.  Administration Method: Intravenous bolus. |
| Varsha et al. (2024) India single-centre No registration reported | Propofol Sevoflurane | 1. Adult patients 2. Aged ≥18 years 3. Scheduled for elective coronary artery bypass grafting (CABG) 4. With cardiopulmonary bypass (CPB) | 1. Emergency surgery 2. Limited language comprehension 3. Preoperative morbidities such as Parkinson's disease , Alzheimer's disease , mild cognitive impairment , and epilepsy 4. Concurrent use of anticholinergics , antiepileptic , or antidepressant medications 5. Allergies to any of the anesthesia components | Propofol group: Loading Dose: No specific loading dose of propofol was mentioned. Anesthesia was induced with a bolus dose of propofol up to 2 mg/kg. Maintenance Infusion: Propofol was titrated to maintain the bispectral index (BIS) between 40 and 60 throughout the surgery. Administration Method: Intravenous Sevoflurane group: Loading Dose: No specific loading dose of sevoflurane was mentioned. Anesthesia was induced with a bolus dose of propofol up to 2 mg/kg (initial induction), and then sevoflurane was used for maintenance. Maintenance Infusion: Sevoflurane was titrated to maintain the bispectral index (BIS) between 40 and 60 throughout the surgery. Administration Method: Inhalation (volatile anesthesia) |
| Verdonk et al.(2024) France Multi-centre  NCT02892916 | Ketamine  NS | 1. Age ≥60 years 2. Competent to provide informed consent 3. Scheduled for major orthopedic surgery 4. Procedure under general anesthesia | 1. ASA score >4 2. Allergy/contraindication to Ketamine  3. Chronic antipsychotic medication use 4. Drug misuse history 5. Emergency surgery (e.g., hip fracture) 6. Expected hospital stay <48 hours 7. Non-French-speaking patients 8. Severe alcoholic liver disease (TP <50% or bilirubin >50 mmol/L) 9. Severe auditory/visual disorders | Ketamine group:  Loading Dose: A single intravenous bolus of 0.5 mg/kg of Ketamine was administered after the induction of anesthesia and before the surgical incision  Maintenance Infusion: No further infusion was administered after the initial bolus  Administration Method:Intravenous  Control group:  Loading Dose: The control group received a placebo in the form of an equivalent volume of 0.9% NS solution  Infusion Rate: The saline was administered at the same volume and rate as the Ketamine in the experimental group  Administration Method:Intravenous |
| Wang et al.(2002)  China  single-centre  No registration reported | Lidocaine NS | 1.Elective coronary artery bypass surgery;  2.Age ≤70 years; 3. LVEF >35%;  4.No neurological or psychiatric disorders;  5.No lidocaine allergy;  6.No severe renal/hepatic dysfunction;  7.Able to complete neuropsychological tests. | 1.Age >70 years;  2.Previous cardiac surgery;  3.Combined other cardiac procedures;  4.Preoperative cognitive impairment;  5.Severe comorbidities. | Lidocaine Group: Induction of anesthesia: Standard induction with midazolam, morphine, fentanyl, and vecuronium. On this basis, lidocaine 1.5 mg/kg was administered as a bolus over 5 min at the opening of the pericardium. Maintenance of anesthesia: Intravenous infusion of fentanyl and midazolam, supplemented with isoflurane or propofol. On this basis, intravenous infusion of lidocaine 4 mg/min was added until the end of the operation. Additional administration: Lidocaine 4 mg/kg was added to the cardiopulmonary bypass (CPB) priming solution. Administration method: Intravenous infusion and CPB priming. Control Group: Induction of anesthesia: Standard induction with midazolam, morphine, fentanyl, and vecuronium. On this basis, NS was administered in the same volume and rate as the 2% lidocaine bolus. Maintenance of anesthesia: Intravenous infusion of fentanyl and midazolam, supplemented with isoflurane or propofol. On this basis, intravenous infusion of NS was added at the same volume and rate as the lidocaine infusion. Additional administration: NS was added to the cardiopulmonary bypass (CPB) priming solution in the same volume as the lidocaine group. Administration method: Intravenous infusion and CPB priming. |
| Wang et al.(2017) China single-centre No registration reported | Ulinastatin NS | 1. Aged ≥60 years 2. ASA I and II 3. TNM stage II and III tumors 4. Having received neoadjuvant chemotherapy 5. Scheduled for radical resection of esophageal cancer under one-lung ventilation 6. Having at least a secondary education level 7. A preoperative mini-mental state examination (MMSE) score of >23 | 1. Any significant cardiovascular disease  2. Abnormal pulmonary function test results  3. History of central nervous system disease  4. Serious hearing or visual impairment  5. Any communication impediment  6. History of antipsychotic drug administration within 6 months | Ulinastatin group： Loading Dose：Before anesthesia induction, the U group was administered 10,000 U/kg ulinastatin diluted to 100 mL with NS and infused intravenously over a period of 20 min. Maintenance Infusion：This was followed by 5000 U/kg ulinastatin daily after surgery on days 1 to 3. Administration Method：intravenous Control group： Loading Dose：The control group received an equivalent amount of NS. Infusion Rate：The saline was administered at the same volume and rate as the ulinastatin in the experimental group. Administration Method：intravenous |
| Wang et al.(2018)  China  single-centre  ChiCTR-IPR-16008931 | Ulinastatin NS | 1.aged ≥65 years, scheduled for elective lumbar discectomy under general anesthesia. 2.ASA I-II. | 1. MMSE score ≤23 2. Stroke within 6 months 3. Any other central nervous system (CNS) diseases 4. Acute or chronic infectious diseases 5. Trauma 6. Tumor 7. Lower digestive diseases 8. Taking anti-inflammatory drugs or immunosuppressants 9. Preoperative WBC ≥10×10⁹/L 10. Severe deafness or vision problems 11. Illiteracy 12. Communication difficulties related to pronunciation or dialect 13. Postoperative delirium 14. Refusal or unexpected discharge | Ulinastatin Group: Induction of anesthesia: Intravenous induction with sufentanil 0.3 µg/kg, etomidate 0.2 mg/kg, and rocuronium 0.8 mg/kg. Intervention: Ulinastatin 10,000 units/kg (diluted in NS to 100 ml) was administered as an intravenous infusion over 30 min before surgical incision. Maintenance of anesthesia: Intravenous infusion of propofol 3–5 mg/kg/h and remifentanil 0.3 µg/kg/min until the end of operation, with intermittent sufentanil (up to 0.5 µg/kg). Patients were intubated and ventilated with 50% oxygen. Postoperative intervention: Ulinastatin 5,000 units/kg was administered intravenously on postoperative days 1 and 2. Administration method: Intravenous infusion. Placebo Group: Induction of anesthesia: Intravenous induction with sufentanil 0.3 µg/kg, etomidate 0.2 mg/kg, and rocuronium 0.8 mg/kg. Intervention: Placebo (100 ml NS) was administered as an intravenous infusion over 30 min before surgical incision. Maintenance of anesthesia: Intravenous infusion of propofol 3–5 mg/kg/h and remifentanil 0.3 µg/kg/min until the end of operation, with intermittent sufentanil (up to 0.5 µg/kg). Patients were intubated and ventilated with 50% oxygen. Postoperative intervention: The same volume of NS was administered intravenously on postoperative days 1 and 2. Administration method: Intravenous infusion. |
| Wang et al.(2020) China single-centre No registration reported | Dexmedetomidine NS | 1.Patients diagnosed with gastric cancer who consented to surgical resection 2.No prior radiotherapy, chemotherapy, or comorbidity with other malignant tumors | 1.Patients with liver and renal dysfunction 2.Patients with communication and cognitive dysfunction 3.Patients who did not cooperate with the requirements of the study | Dexmedetomidine Group： Loading Dose: A loading dose of 0.5 µg/kg of Dexmedetomidine was administered intravenously before the induction of anesthesia. Maintenance Infusion: Following the loading dose, a continuous infusion of Dexmedetomidine at a rate of 0.4 µg/(kg·h) was maintained until the end of the surgery. Administration Method: Intravenous. Control Group： Loading Dose: The control group received a placebo in the form of an equivalent volume of 0.9% NS solution. Infusion Rate: The saline was administered at the same volume and rate as the Dexmedetomidine in the experimental group. Administration Method: Intravenous. |
| Wen et al. (2024) China single-centre ChiCTR2300070805 | Butorphanol Sufentanil | 1. Age ≥ 65 years  2. ASA physical status I–III  3. Elective surgery under general anesthesia with expected duration ≥ 2 h  4. BMI ≤ 30 kg/m²  5. Pre-operative MMSE > 23 and MoCA ≥ 26  6. No contraindication to butorphanol  7. No severe neurologic, psychiatric, hepatic, renal, visual or auditory disease  8. No use of sedatives, analgesics or antidepressants within 1 month  9. No active infection | 1. Peri-operative red-blood-cell transfusion > 3 units  2. Post-operative infection  3. Inadequate post-operative analgesia  4. Inability to provide blood samples or complete neurocognitive tests  5. Refusal to participate  6. ICU admission after surgery  7. Loss to follow-up | Butorphanol group:  Induction: 40 μg kg⁻¹ i.v. bolus  Maintenance: supplementary boluses at anaesthetist’s discretion  Post-op PCIA: 0.15 mg kg⁻¹ butorphanol in 100 mL NS (background 1.5 mL h⁻¹, PCA 2 mL, lock-out 15 min)  Route: intravenous  Control group:  Induction: 0.4 μg kg⁻¹ sufentanil i.v. bolus  Maintenance: supplementary boluses as needed  Post-op PCIA: 1.5 μg kg⁻¹ sufentanil in 100 mL NS (same pump settings)  Route: intravenous |
| Wittwer et al.(2023) USA single-centre NCT02554253 | Ketamine  Propofol | 1. Patients aged 70 and older 2. Requiring complex cardiac surgery at a tertiary care center 3. Involving more than one heart valve 4. Redo sternotomy procedures 5. Combined valvular and Coronary Artery Bypass Grafting (CABG) procedures | 1. Left or right ventricular assist device implantation or explantation 2. Procedures not requiring Cardiopulmonary Bypass (CPB) 3. Active infection or sepsis 4. Severe hepatic disease or ascites 5. Preoperative renal dysfunction including a baseline creatinine ≥1.5 mg/dL or requiring dialysis 6. Use of immunosuppressive medication (including steroid use) 7. Immunodeficiency syndrome 8. Known neurologic or psychiatric disorder 9. Use of drugs for psychosis | Ketamine group:  Induction Dose: Patients in the Ketamine group received 1–2 mg/kg of intravenous Ketamine for induction of anesthesia  Maintenance: Additional medication was administered as needed to achieve intubating conditions. The remainder of the anesthetic was as per institutional routine including a maximal fentanyl bolus dosing of 10 µg/kg and maintenance with isoflurane. Dexmedetomidine was started as an infusion at sternal closure for patient transfer to the intensive care unit (ICU) and ICU sedation as per our standard practice, dose 0.5–1.5 mcg/kg/hr  Administration Method: intravenous  Propofol group:  Induction Dose: Patients in the propofol group received 0.5–1 mg/kg of intravenous propofol for induction of anesthesia  Maintenance: Additional medication was administered as needed to achieve intubating conditions. The remainder of the anesthetic was as per institutional routine including a maximal fentanyl bolus dosing of 10 µg/kg and maintenance with isoflurane. Dexmedetomidine was started as an infusion at sternal closure for patient transfer to the intensive care unit (ICU) and ICU sedation as per our standard practice, dose 0.5–1.5 mcg/kg/hr  Administration Method: intravenous |
| Wu et al.(2024) China single-centre NCT04601350 | Remimazolam Midazolam | 1. Patients older than 65 years  2. Gender unrestricted  3. American Society of Anesthesiologists (ASA) physical status of class I to III  4. Undergoing lower extremity fracture surgery  5. No preoperative cognitive impairment  6. Assessed by the Mini Mental State Examination (MMSE) score: >17 for illiteracy, >20 for primary school, and >24 for middle school and above  7. Estimated surgery duration ≥1 h | 1. Refusal or non-cooperation of patients and family members 2. Inability to understand and answer the questionnaire without help 3. Previous history of allergy to sedative or analgesic drugs 4. Previous history of psychiatric disorders 5. Long-term use of sedative, analgesic drugs or anti-anxiety drugs 6. Serious interference of intraoperative EEG signals 7. Serious circulatory failure during the operation 8. Need to be transferred to ICU for further treatment after surgery 9. Incomplete follow-up information | Remimazolam group：  Loading Dose: A loading dose of 0.1 mg/kg of Remimazolam was administered intravenously over a period of 1 minute before the induction of anesthesia.  Maintenance Infusion: Following the loading dose, a continuous infusion of Remimazolam at a rate of 0.1 mg/(kg·h) was maintained until the end of the surgery.  Administration Method: Intravenous.  Midazolam group：  Loading Dose: The control group received a loading dose of 0.05 mg/kg of Midazolam intravenously over a period of 1 minute before the induction of anesthesia.  Maintenance Infusion: Following the loading dose, a continuous infusion of NS at a rate of 0.1 mL/(kg·h) was maintained until the end of the surgery.  Administration Method: Intravenous. |
| Xu et al.(2013)  China  single-centre  No registration reported | Ulinastatin NS | 1.age > 65 years, scheduled for elective abdominal surgery. 2.ASA I-II | 1. MMSE ≤ 23 2. Diabetes mellitus 3. Cardiovascular or cerebrovascular disease 4. Hyperlipidemia 5. Osteoarthritis 6. Neurological or psychiatric disease 7. Abnormal renal or hepatic function 8. Multiple drug use 9. Hearing or vision impairment 10. Illiteracy 11. Mental retardation 12. Sedative and antidepressant medication use 13. History of alcoholism 14. History of past anesthesia | Ulinastatin Group: Induction of anesthesia: Intravenous induction with midazolam, propofol, remifentanil, and cisatracurium. Intervention: On this basis, ulinastatin 10,000 units/kg was administered as an intravenous infusion over 30 min before surgical incision. Maintenance of anesthesia: Inhalation of sevoflurane and intravenous infusion of remifentanil. No additional specific maintenance related to ulinastatin was mentioned. Postoperative intervention: Ulinastatin 5,000 units/kg was administered intravenously on postoperative days 1-3. BIS monitoring: BIS values were maintained between 40 and 60. Administration method: Intravenous infusion. Control Group: Induction of anesthesia: Intravenous induction with midazolam, propofol, remifentanil, and cisatracurium. Intervention: On this basis, placebo (100 mL of 0.9% NaCl) was administered as an intravenous infusion over 30 min before surgical incision. Maintenance of anesthesia: Inhalation of sevoflurane and intravenous infusion of remifentanil. No additional specific maintenance related to placebo was mentioned. Postoperative intervention: The same volume of NS was administered intravenously on postoperative days 1-3. BIS monitoring: BIS values were maintained between 40 and 60. Administration method: Intravenous infusion. |
| Xu et al.(2017) China single-centre No registration reported | Dexmedetomidine NS | 1.Patients aged above 60 years old  2.Patients or their families who were informed of the study content and signed the written informed consent  3.Indications for laparoscopic ovarian cystectomy  4.Patients without primary mental disorders or dementia | 1.Patients allergic to dexmedetomidine  2.Patients with alcoholism  3.Patients who are deaf or mute  4.Patients who voluntarily refused to participate in this study  5.Patients complicated with primary diseases in the heart, lungs, liver, kidneys, or urinary system | Dexmedetomidine group： Loading Dose: A loading dose of 0.8 µg/kg of Dexmedetomidine was administered intravenously at the beginning of the operation. Maintenance Infusion: Following the loading dose, a continuous maintenance infusion of Dexmedetomidine at a rate of 0.5 µg/(kg·h) was maintained until the end of the surgery. Administration Method: intravenous. Control group： Loading Dose: The control group received a placebo in the form of an equivalent volume of 0.9% NS solution. Infusion Rate: The saline was administered at the same volume and rate as the Dexmedetomidine in the experimental group. Administration Method: intravenous. |
| Yang et al.(2015) China single-centre ChiCTR-TRC-14004121 | Butorphanol  Fentanyl NS | 1. Male patients aged 65 to 77 years 2. Patients with obstructive sleep apnea hypopnea syndrome (OSAHS) 3. Scheduled for H - UPPP under general anesthesia 4. Weight 88 - 111 kg, BMI 28 - 35 kg/m² 5. Apnea - hypopnea index 21 - 42 times/h 6. American Society of Anesthesiologists (ASA) physical status grade I or II 7. Mallampati airway classification grade I to IV 8. Simple mental state examination score > 23 9. Junior high school or higher education 10. No significant history of neurological disease or hyperthyroidism 11. No liver or renal dysfunction 12. No severe visual or hearing impairment 13. No recent use of sedatives, anti - coagulants, anti - depressants, or analgesics | 1. Patients with a history of drug use  2. Patients with significant neurological disease or hyperthyroidism  3. Patients with liver or renal dysfunction  4. Patients with severe visual or hearing impairment  5. Patients who recently used sedatives, anti - coagulants, anti - depressants, or analgesics | Butorphanol Group:  Loading Dose: A loading dose of 2 mg of butorphanol was administered intravenously 10 minutes before the induction of anesthesia.  Maintenance Infusion: No continuous infusion was maintained after the loading dose.  Administration Method: Intravenous  Fentanyl Group： Loading Dose: A loading dose of 0.1 mg of fentanyl was administered intranasally 10 minutes before the induction of anesthesia. Maintenance Infusion: No continuous infusion was maintained after the loading dose. Administration Method: Intranasal Control Group： Loading Dose: The control group received a placebo in the form of 2 mL of 0.9% NS solution intravenously 10 minutes before the induction of anesthesia. Infusion Rate: No continuous infusion was maintained after the initial dose. Administration Method: Intravenous |
| Zhang et al.(2015) China single-centre No registration reported | Dexmedetomidine  NS | 1.Aged 45–80 years undergoing elective robot-assisted laparoscopic radical cystectomy and ileal conduit diversion. 2.ASA I–III 3.BMI 18–25 kg/m² | 1.Preoperative cognitive dysfunction. 2.History of serious cardiovascular disease, chronic obstructive pulmonary disease. 3.Severe hepatic and renal dysfunction. 4.Endocrine and metabolic diseases. 5.Central nervous system disease. 6.Acute upper respiratory infection. 7.Severe visual and hearing disorders. 8.Drug dependence, excessive drinking. 9.Long-term use of sedatives, antidepressants, and anxiolytics. | Dexmedetomidine Group: Induction of anesthesia: Intravenous induction with cisatracurium, sufentanil, and propofol. On this basis, dexmedetomidine 1 µg/kg was infused over 10 minutes. Maintenance of anesthesia: Intravenous infusion of propofol + remifentanil. On this basis, intravenous infusion of dexmedetomidine 0.5 µg/kg/h was added. BIS monitoring: BIS values were maintained between 40 and 60. Administration method: Intravenous infusion. Control Group: Induction of anesthesia: Intravenous induction with cisatracurium, sufentanil, and propofol. On this basis, NS 5 mL was added. Maintenance of anesthesia: Intravenous infusion of propofol + remifentanil. On this basis, intravenous infusion of NS 0.5 mL/kg/h was added. BIS monitoring: BIS values were maintained between 40 and 60. Administration method: Intravenous infusion. |
| Zhang et al.(2018) China Multi-centre NCT02662257 | Propofol Sevoflurane | 1. Adults aged ≥65 and <90 years  2. Scheduled for first - time cancer surgery expected to last ≥2 hours under general anesthesia  3. Without prior radio - or chemotherapy | 1. History of schizophrenia  2. History of epilepsy  3. History of Parkinsonism  4. History of myasthenia gravis  5. Inability to communicate preoperatively  6. ASA physical status > III  7. Severe hepatic (Child - Pugh C) dysfunction  8. Severe renal (dialysis - dependent) dysfunction  9. Neurosurgery | Propofol group: Induction of anesthesia: Intravenous induction with midazolam, remifentanil and/or sufentanil, propofol, and rocuronium or cisatracurium. Maintenance of anesthesia: Propofol infusion was used to maintain anesthesia. BIS monitoring: BIS values were maintained between 40 and 60 by adjusting the infusion rate of propofol. Administration method: Intravenous infusion. Sevoflurane group: Induction of anesthesia: Intravenous induction with midazolam, remifentanil and/or sufentanil, propofol, and rocuronium or cisatracurium. Maintenance of anesthesia: Sevoflurane inhalation was used to maintain anesthesia. BIS monitoring: BIS values were maintained between 40 and 60 by adjusting the inhaled concentration of sevoflurane. Administration method: Inhalation. |
| Zhang et al.(2019) China single-centre No registration reported | Dexmedetomidine NS | 1. Patients with colorectal cancer confirmed by preoperative or intraoperative pathology 2. Patients who actively received and cooperated with the treatment 3. Patients with complete data 4. Patients without serious immune system diseases 5. Patients with ASA grade II-III aged 60-85 years and normal cognitive function MMSE score >27 points | 1. Patients with central nervous system conditions and mental illness  2. Patients taking sedatives or antidepressants  3. Patients with hypertension, diabetes, coronary heart disease, cerebral infarction, or liver and kidney dysfunction  4. Patients who underwent radiotherapy or chemotherapy before surgery | Dexmedetomidine group： Loading Dose: A loading dose of 1 µg/kg of Dexmedetomidine (Dex) was administered intravenously over a period of 15 minutes before the induction of anesthesia. Maintenance Infusion: Following the loading dose, a continuous infusion of Dex at a rate of 0.2 - 0.7 µg/(kg·h) was maintained until 30 minutes before the end of the surgery. Administration Method: intravenous. Control group： Loading Dose: The control group received a placebo in the form of an equivalent volume of 0.9% NS solution. Infusion Rate: The saline was administered at the same volume and rate as the Dexmedetomidine in the experimental group. Administration Method: intravenous. |
| Zhang et al.(2021) China single-centre No registration reported | Dexmedetomidine NS | 1. Age between 18 - 79 years 2. ASA physical status classification I - III 3. Operation time within 24 hours 4. Preoperative approval by the ethical board committee and voluntary signing of the study consent form | 1. History of dementia and psychosis 2. Severe cerebrovascular disease 3. History of multiple surgeries 4. History of severe perioperative infections in various systems 5. Perioperative massive hemorrhage and severe acid - base imbalance and electrolyte imbalance 6. Diabetes mellitus 7. Perioperative hormone use | Dexmedetomidine group： Loading Dose: A loading dose of 1 µg/kg of Dexmedetomidine (DEX) was administered intravenously over a period of 10 minutes before the induction of anesthesia. Maintenance Infusion: Following the loading dose, a continuous infusion of DEX at a rate of 4 ml/h was maintained for postoperative patient-controlled analgesia (PCA) for up to 72 hours. Administration Method: Intravenous Control group： Loading Dose: The control group received a placebo in the form of an equivalent volume of 0.9% NS solution. Infusion Rate: The saline was administered at the same volume and rate as the Dexmedetomidine in the experimental group (4 ml/h) for postoperative PCA for up to 72 hours. Administration Method: Intravenous |
| Zhao et al.(2020) China single-centre ChiCTR - IPD - 17010950 | Dexmedetomidine Sufentanil | 1. Patients aged over 65 years  2. Scheduled for non-cardiac major surgery (thoracic, general, genitourinary, gynecologic, and orthopedic surgeries)  3. ASA physical status I-III | 1. Regular use of opioids, sedatives, antidepressants, or anxiolytic drugs prior to surgery  2. Drug addiction  3. Severe visual or hearing disorders  4. Preoperative history of schizophrenia, epilepsy, Parkinsonism, or myasthenia gravis  5. Inability to communicate during the preoperative period (owing to coma, profound dementia, or a language barrier)  6. Brain injury or a history of neurosurgery  7. Serious hepatic dysfunction (Child-Pugh class C)  8. Serious renal dysfunction (undergoing dialysis before surgery)  9. A preoperative left ventricular ejection fraction less than 50%  10. Sick sinus syndrome, severe sinus bradycardia (<50/min), or a second-degree or greater atrioventricular block without a pacemaker  11. Allergy to sufentanil or DEX  12. Preoperative MMSE scores below specific thresholds | Dexmedetomidine group： Loading Dose：A loading dose of 1 µg/kg of Dexmedetomidine (DEX) was intravenously infused over 10 minutes before anesthesia induction. Maintenance Infusion：Following the loading dose, a continuous infusion of DEX at a rate of 200 µg or 400 µg was maintained for postoperative patient - controlled analgesia (PCA) for up to 72 hours. Administration Method：intravenous Control group： Loading Dose：The control group received a placebo in the form of an equivalent volume of 0.9% NS solution. Infusion Rate：The saline was administered at the same volume and rate as the Dexmedetomidine in the experimental group. Administration Method：intravenous |
| Zhou et al.(2019) China single-centre  ChiCTR-IPR-17012544 | Ulinastatin Dexmedetomidine NS | 1. Aged 60–80 years 2. ASA grades I–III 3. Education level above elementary school 4. Either gender 5. Undergoing heart valve replacement surgery with cardiopulmonary bypass | 1. MMSE score ≤23 2. acute or chronic infectious diseases 3. taking anti-inflammatory drugs or immunosuppressants 4. a stroke in the prior 6 months or other central nervous system diseases 5. peptic ulcer disease 6. ICU stay ≥3 days 7. BMI >35 8. severe deafness or vision problems 9. illiteracy or communication difficulties 10. postoperative delirium 11. refusal or unexpected discharge | Ulinastatin group： Loading Dose: A loading dose of 20,000 UI/kg of Ulinastatin (U) was administered intravenously immediately after induction. Maintenance Infusion: The same dose of 20,000 UI/kg was administered again on the first day after surgery. Administration Method: intravenous. Dexmedetomidine group： Loading Dose: A loading dose of 0.4 µg/kg of Dexmedetomidine (Dex) was administered intravenously over a period of 15 minutes before the induction of anesthesia. Maintenance Infusion: Following the loading dose, a continuous infusion of Dex at a rate of 0.4 µg/(kg·h) was maintained until 2 hours before extubation. Administration Method: intravenous. Control group： Loading Dose: The control group received a placebo in the form of an equivalent volume of 0.9% NS solution. Infusion Rate: The saline was administered at the same volume and rate as the Ulinastatin and Dexmedetomidine in the experimental groups. Administration Method: intravenous. |
| Zhu et al. (2021) China single-centre ChiCTR1900027574 | Ropivacaine  NS | 1. ASA I–III  2. Age > 65 yr  3. Elementary education or above  4. Preoperative MMSE ≥ 24  5. BMI < 31 kg/m² | 1. severe cardio-cerebrovascular or respiratory disease  2. hepatic/renal dysfunction  3. history of psychiatric or neurologic disorders  4. chronic use of antipsychotics or narcotic analgesics  5. alcohol abuse  6. severe visual/hearing/speech impairment  7. allergy to local anesthetics | Ropivacaine group:  Loading Dose: Single-shot ultrasound-guided bilateral quadratus lumborum block with 20 mL 0.3 % ropivacaine per side (total 40 mL) before induction of general anaesthesia;  Control group:  Loading Dose: Same volume (20 mL/side) of 0.9 % saline injected identically.  Route: Both injections given via local infiltration / perineural route. |
| Zhu et al.(2016)  China  single-centre  No registration reported | Parecoxib NS | 1.aged 65–80 years,scheduled for total knee arthroplasty. 2.ASA I or II | 1. ASA > II 2. BMI > 35 3. MMSE < 23 4. Neurological or psychiatric disorders 5. History of allergic reactions to NSAIDs 6. History of drug and alcohol abuse 7. Hepatic and/or kidney dysfunction 8. Peptic ulcer disease 9. Cardiac-cerebral vascular disease 10. Diabetes mellitus 11. Use of analgesics or antidepressants 12. Inability to comply with the study protocol or procedures | Parecoxib Group: Induction of anesthesia: Intravenous induction with 0.1 mg/kg midazolam, 0.2 mg/kg cisatracurium, 2 mg/kg propofol, and 0.6 µg/kg sufentanil. Intervention: Parecoxib sodium 40 mg was administered as an intravenous bolus shortly after induction of general anesthesia. Maintenance of anesthesia: Maintained with remifentanil and propofol. Bispectral Index Score was maintained at 40–60. Patients revived spontaneously without anesthetic antagonists. Postoperative intervention: Parecoxib sodium 40 mg was administered as an intravenous bolus 12 hours post-surgery. Administration method: Intravenous bolus. Control Group: Induction of anesthesia: Intravenous induction with 0.1 mg/kg midazolam, 0.2 mg/kg cisatracurium, 2 mg/kg propofol, and 0.6 µg/kg sufentanil. Intervention: Placebo (NS) was administered as an intravenous bolus shortly after induction of general anesthesia. Maintenance of anesthesia: Maintained with remifentanil and propofol. Bispectral Index Score was maintained at 40–60. Patients revived spontaneously without anesthetic antagonists. Postoperative intervention: Placebo (NS) was administered as an intravenous bolus 12 hours post-surgery. Administration method: Intravenous bolus. |
| Zhu et al.(2024) China single-centre ChiCTR2200061055 | Ropivacaine Sufentanil | 1. Age between 65 and 85 years  2. American Society of Anesthesiologists (ASA) II or III  3. Body mass index (BMI) between 18 and 28 kg/m²  4. Received primary school education or above  5. Mini - Mental State Examination (MMSE) score ≥ 24 before surgery  6. Scheduled for hip arthroplasty (HA) | 1. Serious cardiopulmonary or other vital organ disease  2. History of neurological and psychiatric disorders  3. Coagulopathy  4. Long - term use of drugs  5. Severe impairment of visual, hearing or speech functions  6. Allergic to local anesthetics | Ropivacaine Group Loading Dose: A single dose of 20 mL of 0.375% ropivacaine was administered via PENG block using an ultrasound-guided technique before spinal anesthesia. Administration Method: Ultrasound-guided perineural injection. Control group:  Loading Dose: The control group did not receive a specific loading dose of sufentanil before surgery.  Maintenance Infusion: The control group received sufentanil as part of the patient-controlled intravenous analgesia (PCIA) pump after surgery. The PCIA pump contained 2 µg/kg sufentanil, 0.15 mg/kg tropisetron, and 100 mL NS. The background infusion rate was 2 mL/h, with a 0.5 mL intravenous bolus on demand and a 15-minute lockout period.  Administration Method: intravenous infusion. |

# **APPENDIX 3** Cognitive assessment tools and diagnostic criteria **of included studies**

| **Study, Year [reference]Country,No. of centres, Registration No.** | **Drug** | **Assessment scale** | **Diagnostic criteria** |
| --- | --- | --- | --- |
| **Chawdhary et al.(2020)** | Dexmedetomidine Propofol | MMSE、VVLT、DSST、Trail Making Test (Part A and Part B)、Stroop Colour Word Test (Part A and Part B） | In two or more neuro-psychological tests, the postoperative performance decreased by 1 standard deviation or more compared to the preoperative score. |
| **Chen et al.(2001)** | Desflurane  Sevoflurane | MMSE | The postoperative MMSE score decreased by ≥ 2 points compared to the preoperative score. |
| **Chen et al.(2013)** | Dexmedetomidine NS | MMSE | Operational definition of cognitive impairment based on the MMSE score range: mild, moderate cognitive impairment |
| **Chen et al.(2015)** | Dexmedetomidine NS | MMSE | MMSE score < 24 points |
| **Chen et al.( 2020)** | Dexmedetomidine NS | MMSE、Wechsler Adult Intelligence Scale、Memory Scale、Simple Smart Scale、Digital Symbol Test Scale, Accumulation, Visual Regeneration, Digital Breadth Test Scale, Small Story Memory Test Scale, Trace Connection Test Scale | MMSE: A score lower than 23 is considered indicative of cognitive dysfunction. Wechsler Scale: A combined Z-score higher than 1.96 indicates the presence of POCD. This score is calculated by dividing the difference between the preoperative and postoperative scores by the standard deviation. |
| **Cheng et al.(2021)** | Dexmedetomidine NS | MMSE、ISPOCD | If the Z value of either of the two individual tests is ≥ 1.96, or the combined Z value is ≥ 1.96, then the patient is diagnosed as having delayed neurocognitive recovery. |
| **Deepak et al.(2013)** | Desflurane Sevoflurane | MMSE | The MMSE score decreased by 2 points or more compared to the preoperative value. |
| **Egawa et al.(2016)** | Propofol sevoflurane | MMSE、TMT、Digit Span Test、Grooved Pegboard Test | Among the seven tests, the scores of at least two tests decreased by more than 20% compared to the pre-test results. |
| **Fang et al.(2014)** | Dexamethasone NS | WBS、Halstead-Reitan、TMT (Part A) | In at least two tests, its postoperative performance decreased by ≥ 1 standard deviation compared to the preoperative level. |
| **Gao et al.(2020)** | Dexmedetomidine NS | MMSE | American Psychiatric Association's POCD diagnostic criteria |
| **Gao et al.(2021)** | Dexmedetomidine NS | MMSE | MMSE score less than 27 |
| **Geng et al.(2017)** | Propofol Sevoflurane Isoflurane | MMSE、Vision test、Digit Symbol Substitution Test、Cumulative test、Digit span、Trail Making Test Part A、RAVLT、Grooved Pegboard Test | A >20% decrease in performance in at least two tests compared to baseline. |
| **Ghazaly et al.(2023)** | Dexmedetomidine Ketamine  NS | MMSE | MMSE score < 26 points |
| **Glumac et al.(2017)** | Dexamethasone  NS | MMSE、RAVLT、WBS、SDMT、CPET | In at least one test, the reliable change index (RCI) is equal to or less than -1.96. |
| **Gong et al.(2018)** | Dexmedetomidine NS | MMSE、MoCA | MMSE: A score below 27 points. MoCA: A score below 26 points. |
| **Guo et al.(2020)** | Sevoflurane Propofol | MMSE、Visual Verbal Learning Test、Concept Shifting Test、Stroop Color Word Interference Test (Part C)、Letter-digit Coding Test | The Z scores in both independent tests were all ≥ 1.96 |
| **Hashemi et al.(2013)** | Lidocaine NS | MMSE | Postoperative assessment showed that the MMSE score was less than 23 points. |
| **He et al.(2019)** | Sufentanil Fentanyl | MoCA | Using the MoCA scale, if two or more items show functional impairment, the patient is diagnosed with POCD. |
| **He et al.(2020)** | Dexmedetomidine Ropivacaine NS | MMSE | A mean MMSE score decline was >2 points between postoperative and preoperative surgery. |
| **Hollinger et al.(2021)** | Haloperidol  Ketamine  NS | MMSE、DOS、Nu-DESC、 ICDSC | On one of the first three days after the surgery, the MMSE score was lower than 27 points and decreased by at least 2 points compared to before, or the MMSE score was lower than 24 points. |
| **Kim et al.(2022)** | Dexmedetomidine NS | MMSE-K、DLOTCA-G™ battery | Postoperative cognitive dysfunction is defined as a combined Z-score of ≤ -1.96. |
| **Kletecka et al. (2019)** | Propofol  Sevoflurane | DST、Letter-Number Sequencing、Verbal Fluency、COWAT、TMT、SCWT | A decrease of ≥ 3 test scores by ≥ 1 standard deviation |
| **Kurup et al.(2023)** | Dexmedetomidine Lidocaine | MMSE、MoCA、Stroop Test、Porteus Maze Test、Trail Making Test (Part B) | A reduction of more than 20% in the baseline scores of any 2 tests was defined as POCD. |
| **Kuzminskaite et al.(2023)** | Desflurane  Sevoflurane | 10-item battery covering memory, attention, logical reasoning (max 135 pts) | ≥ 20 % decrease in total cognitive score vs baseline |
| **Lee et al.(2015)** | Ketamine  NS | MMSE、TMT、DST | A Z-score of ≥ 1.96 in two or more tests, or a combined Z-score of ≥ 1.96. |
| **Li et al.(2015)** | Dexmedetomidine NS | MMSE | Cognitive Impairment: MMSE score < 27. Mild Cognitive Impairment: MMSE score 21-27. Moderate Cognitive Impairment: MMSE score 9-21. Severe Cognitive Impairment: MMSE score < 9. |
| **Li et al.(2019)** | Dexmedetomidine Propofol Midazolam | MoCA、SCWT、Digit Span Test、Digit Symbol Test、Associative Learning and Memory Test | The RCI score in at least two tests was less than -1.96, and/or its combined z-score was less than -1.96. |
| **Li et al.（2021）** | Propofol Sevoflurane | Word learning、Word recall、Cognitive flexibility、Distractibility、Working memory | The Z-score on two individual tests was ≥ 1.96, or the combined Z-score was ≥ 1.96. |
| **Liu et al.(2020)** | Dexmedetomidine NS | MMSE | A drop by one standard deviation (more than 2 scores) in the MMSE tests from before to after surgery。 |
| **Liu et al.(2025)** | Lidocaine  NS | MMSE | Decline >2 points in MMSE score compared with pre-operative baseline |
| **Livia et al.(2016)** | Dexamethasone NS | MMSE、TICS、RAVLT、TMT、SDMT、SF-36 | The diagnosis of POCD is based on the composite cognitive index, defined as cognitive impairment occurring in TICS and at least one of the 8 possible deficits in other neuropsychological tests. The changes in neuropsychological tests are compared with normative data matched by age, gender and educational level. |
| **Luo et al.(2024)** | Ketamine  NS | MMSE | The postoperative (MMSE score) decreased by at least 2 points. |
| **Ma et al.(2023)** | Ketamine  NS | MMSE | The MMSE score decreased by ≥ 2 points from the baseline. |
| **Mansouri et al.(2019)** | Dexmedetomidine Midazolam NS | MMSE | MMSE score less than 20 |
| **Meineake et al.(2014)** | Desflurane Sevoflurane | MMSE | A decrease in MMSE score of more than 2 points from baseline. |
| **Min et al.(2023)** | Remimazolam  Dexmedetomidine  NS | MMSE、MoCA | The difference between the postoperative score and the preoperative score shows that the standard deviation of this test before the surgery decreased by ≥ 1 standard deviation. |
| **Mohamed et al. (2014)** | Dexmedetomidine NS | MoCA、Stroop Color-Word Interference Test | The cognitive dysfunction in each test was defined using the 1-standard-deviation rule. |
| **Mu et al.(2017)** | Parecoxib NS | MMSE | A reduction of postoperative MMSE score ≥ 2 from preoperative value. |
| **Nagz et al.(2020)** | Dexmedetomidine NS | MoCA | A score of less than 26 in Moca is considered as postoperative cognitive dysfunction. |
| **Ottens et al.(2014)** | Dexamethasone NS | Corsi Blocks、Rey Auditory Verbal Learning、Grooved Pegboard、Trailmaking test A+B、WAIS Digit Span | The reliable change index (RCI) is less than or equal to -1.96, or the Z-score in at least two different tests is less than or equal to -1.96. |
| **Qiao et al.(2023)** | Desflurane propofol | MMSE | MMSE score that dropped by two or more points compared to baseline. |
| **Racman et al.(2023)** | Dexmedetomidine Propofol | MMSE | A decline in the MMSE score of more than 1 Standard Deviation (SD) from the preoperative baseline（a 1 SD reduction was defined as a drop of 2 or more points on the MMSE） |
| **Rohan et al.(2005)** | propofol Sevoflurane | MMSE、Stroop Color Word Interference Test、Modified Word-Recall Test | The Z score or the combined Z score in either of the two independent tests is ≥ 1.96 |
| **Rörtgen et al.(2010)** | Desflurane Sevoflurane | TAP、DSST、DST、TMT A & B、Well-being Test BF-S、STAI | POCD is defined as a situation where, in both the paper-and-pencil test and the TAP test, 20% of the test items show a 20% or greater decrease in scores. |
| **Sun et al.(2024)** | Dexmedetomidine NS | MMSE | A Z-score of \|Z\| ≥ 1.96 indicated PND |
| **Takazawa et al.(2023)** | Minocycline NS | MMSE、MoCA、VVT、TMT-Part B、SCMT、LDCT、GDS | Overall Z score > 1.96, or in more than two tests, the Z score > 1.96 |
| **Tanaka et al.(2017)** | Desflurane propofol | MMSE、DSST、DST、TMT A、B | In any test, the score decreased by 20% compared to the baseline level. |
| **Tian et al.（2015）** | Parecoxib  NS | MMSE，neuro-psychological battery, SCWT, COWAT | MMSE score fell ≥ 2 points from pre-operative value, or Z-score ≥ 1.96 in two of the neuro-psychological tests. |
| **Varsha et al. (2024)** | Propofol Sevoflurane | MoCA | Compared with the preoperative baseline assessment, the MoCA score decreased by more than 2 points. |
| **Verdonk et al.(2024)** | Ketamine  NS | MoCA、TMT | In at least one cognitive test (MoCA or TMT A or TMT B or TMT B-A), the absolute value of the difference in Z-scores between before and after the surgery was ≥ 1 |
| **Wang et al.(2002)** | Lidocaine NS | Wechsler Memory Scale、Wechsler Adult Intelligence Scale-Revised（Digit Symbol）、TMT（Part A）、Grooved Pegboard Test | If the postoperative score of any test is less than one standard deviation lower than the preoperative score, it is defined as a defect in that test. Any patient who fails in two or more tests is defined as having postoperative cognitive dysfunction. |
| **Wang et al.(2017)** | Ulinastatin NS | MMSE、Digit-span forward test、Digit-span backward test、Digit-symbol test、 Trail-making test、Verbal fluency test、 Word-recognition tasks. | If the Z value of two or more of the seven tests is ≥ 1.96, then the patient is diagnosed with POCD. |
| **Wang et al.(2018)** | Ulinastatin NS | MMSE、MOCA | Z-score is greater than or equal to 1.96 SD |
| **Wang et al.(2020)** | Dexmedetomidine NS | MMSE | Postoperative MMSE score decreased by 2 points or more than preoperative MMSE score |
| **Wen et al. (2024)** | Butorphanol  Sufentanil | MMSE、MoCA | Z-score > 1.96 on either MMSE or MoCA at any post-operative time-point |
| **Wittwer et al.(2023)** | Ketamine  Propofol | MMSE、TMT、HVLTR、Digit Span 、COWAT、Stroop Color and Word Test | A-priori as a decline of >1 SD (i.e. z-score decline of >1) on at least 2 neurocognitive tests. |
| **Wu et al.(2024)** | Remimazolam Midazolam | MMSE | POCD was defined as a reduction of MMSE scores more than 2 points of basal value. |
| **Xu et al.(2013)** | Ulinastatin NS | Wechsler Memory Scale, Wechsler Adult Intelligence Scale-Revised、TMT (Part A)、Grooved Pegboard Test | If the postoperative score of any test decreases by 20% or more compared to the preoperative value, it is defined as a defect in that test. Any patient who has a defect in two or more tests is considered to have POCD. |
| **Xu et al.(2017)** | Dexmedetomidine NS | MoCA | A MoCA score lower than 26 points, or a decrease of 2 points or more compared to the preoperative baseline score. |
| **Yang et al.(2015)** | Butorphanol  Fentanyl NS | MMSE | When the difference between the preoperative and postoperative MMSE scores is 2 points or more, the subject is determined to have POCD. |
| **Zhang et al.(2015)** | Dexmedetomidine  NS | MMSE、DSPT、DSYT、TMT、WRT、VFT | There are two or more test items that have malfunctioned. |
| **Zhang et al.(2018)** | Propofol Sevoflurane | MMSE、MoCA | Z ≥ 1.96 |
| **Zhang et al.(2019)** | Dexmedetomidine NS | MMSE | MMSE score less than 27 |
| **Zhang et al.(2021)** | Dexmedetomidine NS | MMSE | The decline in MMSE scores before and after the surgery was used to calculate POCD. |
| **Zhao et al.(2020)** | Dexmedetomidine Sufentanil | MMSE | Compared with the preoperative baseline score, a decrease of more than 2 points in the MMSE score after surgery indicates the presence of POCD. |
| **Zhou et al.(2019)** | Ulinastatin Dexmedetomidine NS | MMSE、MoCA | If there were not less than two items in the test abnormality ≥1.96, then POCD would be diagnosed. |
| **Zhu et al. (2021)** | Ropivacaine  NS | MMSE, MoCA | Decline ≥ 1 SD in both MMSE and MoCA relative to pre-operative baseline. |
| **Zhu et al.(2016)** | Parecoxib NS | MMSE、Wechsler Memory Scale、Wechsler Adult Intelligence Scale-Revised (WAIS-R)、Halstead-Reitan Trail Making Test (Part A)、Grooved Pegboard Test | In two or more tests, the score decreased by ≥ 1 standard deviation compared to the pre-test level. |
| **Zhu et al.(2024)** | Ropivacaine Sufentanil | MMSE | PND was defined as a decline ≥1 standard deviation (SD) in the postoperative MMSE score compared with the preoperative MMSE score. |

**Abbreviations**: WBS: Wechsler Memory Scale; SDMT: Symbol Digit Modalities Test; CPET: Computerized PsychE Test; TMT: Trail Making Test; DST: Digit Span Test; SCWT: Stroop Color-Word Test; VVT: Visual Verbal Test; HVLT-R: Hopkins Verbal Learning Test–Revised; COWAT: Controlled Oral Word Association Test; TAP: Test Battery for Attentional Performance; TICS: Telephone Interview for Cognitive Status; DOS: Delirium Observation Scale IQCODE:Informant Questionnaire on Cognitive Decline — short form; Katz-ADL: Katz Index of Activities of Daily Living DLOTCA-G:Dynamic Lowenstein Occupational Therapy Cognitive Assessment–Geriatric

# APPENDIX 4 **Risk of bias assessment**

| **Study** | **Selection bias**  Random sequence generation | **Selection bias**  Allocation concealment | **Performance Bias**  Blinding of participants | **Detection bias**  Blinding of primary outcome assessors | **Attrition bias**  Incomplete primary outcome data | **Reporting bias**  Selective reporting | **Other sources of bias**  Funding, baseline characteristics |
| --- | --- | --- | --- | --- | --- | --- | --- |
| **Chawdhary et al.（2020）** | **Low risk of bias** | **Low risk of bias** | **High risk of bias** | **Unclear risk of bias** | **Low risk of bias** | **Low risk of bias** | **Low risk of bias** |
| **Chen et al.（2001）** | **Low risk of bias** | **Unclear risk of bias** | **Low risk of bias** | **Low risk of bias** | **Low risk of bias** | **Low risk of bias** | **Low risk of bias** |
| **Chen et al.（2013）** | **Low risk of bias** | **Low risk of bias** | **Low risk of bias** | **Low risk of bias** | **Unclear risk of bias** | **Low risk of bias** | **Low risk of bias** |
| **Chen et al.（2015）** | **Low risk of bias** | **Low risk of bias** | **Unclear risk of bias** | **Low risk of bias** | **Low risk of bias** | **Low risk of bias** | **Low risk of bias** |
| **Chen et al.（2020）** | **Low risk of bias** | **Unclear risk of bias** | **Low risk of bias** | **Low risk of bias** | **Low risk of bias** | **Low risk of bias** | **Low risk of bias** |
| **Cheng et al.（2021）** | **Low risk of bias** | **Low risk of bias** | **Low risk of bias** | **Low risk of bias** | **Low risk of bias** | **Low risk of bias** | **Low risk of bias** |
| **Deepak et al.（2013）** | **Low risk of bias** | **Unclear risk of bias** | **Low risk of bias** | **Low risk of bias** | **Low risk of bias** | **Low risk of bias** | **Low risk of bias** |
| **Egawa et al.（2016）** | **Low risk of bias** | **Low risk of bias** | **Low risk of bias** | **Low risk of bias** | **Low risk of bias** | **Low risk of bias** | **Low risk of bias** |
| **Fang et al.（2014）** | **Low risk of bias** | **Low risk of bias** | **Low risk of bias** | **Low risk of bias** | **Low risk of bias** | **Low risk of bias** | **Low risk of bias** |
| **Gao et al.（2020）** | **Low risk of bias** | **Unclear risk of bias** | **Low risk of bias** | **Unclear risk of bias** | **Low risk of bias** | **Low risk of bias** | **Low risk of bias** |
| **Gao et al.（2021）** | **Low risk of bias** | **Unclear risk of bias** | **Low risk of bias** | **Low risk of bias** | **Low risk of bias** | **Low risk of bias** | **Low risk of bias** |
| **Geng et al.（2017）** | **Low risk of bias** | **Unclear risk of bias** | **Low risk of bias** | **Low risk of bias** | **Low risk of bias** | **Low risk of bias** | **Low risk of bias** |
| **Ghazaly et al.（2023）** | **Low risk of bias** | **Low risk of bias** | **Low risk of bias** | **Low risk of bias** | **Low risk of bias** | **Low risk of bias** | **Low risk of bias** |
| **Glumac et al.（2017）** | **Low risk of bias** | **Low risk of bias** | **Low risk of bias** | **Low risk of bias** | **Low risk of bias** | **Low risk of bias** | **Low risk of bias** |
| **Gong et al.（2018）** | **Low risk of bias** | **Unclear risk of bias** | **Low risk of bias** | **Low risk of bias** | **Low risk of bias** | **Low risk of bias** | **Low risk of bias** |
| **Guo et al.（2020）** | **Low risk of bias** | **Low risk of bias** | **Low risk of bias** | **Low risk of bias** | **Low risk of bias** | **Low risk of bias** | **Low risk of bias** |
| **Hashemi et al.（2013）** | **Low risk of bias** | **Unclear risk of bias** | **Low risk of bias** | **Low risk of bias** | **Low risk of bias** | **Low risk of bias** | **Low risk of bias** |
| **He et al.（2019）** | **Low risk of bias** | **Unclear risk of bias** | **High risk of bias** | **Low risk of bias** | **Low risk of bias** | **Low risk of bias** | **Low risk of bias** |
| **He et al.（2020）** | **Low risk of bias** | **Low risk of bias** | **Unclear risk of bias** | **Low risk of bias** | **Low risk of bias** | **Low risk of bias** | **Low risk of bias** |
| **Hollinger et al.（2021）** | **Low risk of bias** | **Low risk of bias** | **Low risk of bias** | **Low risk of bias** | **Low risk of bias** | **Low risk of bias** | **Low risk of bias** |
| **Kim et al.（2022）** | **Low risk of bias** | **Low risk of bias** | **Low risk of bias** | **Low risk of bias** | **Low risk of bias** | **Low risk of bias** | **Low risk of bias** |
| **Kletecka et al. (2019)** | **Low risk of bias** | **Low risk of bias** | **Low risk of bias** | **Low risk of bias** | **High risk of bias** | **Low risk of bias** | **Low risk of bias** |
| **Kurup et al.（2023）** | **Low risk of bias** | **Low risk of bias** | **Low risk of bias** | **Low risk of bias** | **Low risk of bias** | **Low risk of bias** | **Unclear risk of bias** |
| **Kuzminskaite et al.(2023)** | **Low risk of bias** | **Low risk of bias** | **Low risk of bias** | **Low risk of bias** | **Low risk of bias** | **Low risk of bias** | **Low risk of bias** |
| **Lee et al.（2015）** | **Low risk of bias** | **Unclear risk of bias** | **Low risk of bias** | **Low risk of bias** | **Low risk of bias** | **Low risk of bias** | **Low risk of bias** |
| **Li et al.（2015）** | **Low risk of bias** | **Low risk of bias** | **Low risk of bias** | **Low risk of bias** | **Low risk of bias** | **Low risk of bias** | **Low risk of bias** |
| **Li et al.（2019）** | **Low risk of bias** | **Unclear risk of bias** | **Low risk of bias** | **Low risk of bias** | **High risk of bias** | **Low risk of bias** | **Low risk of bias** |
| **Li et al.（2021）** | **Low risk of bias** | **Low risk of bias** | **Low risk of bias** | **Low risk of bias** | **Low risk of bias** | **Low risk of bias** | **Low risk of bias** |
| **Liu et al.（2020）** | **Low risk of bias** | **Unclear risk of bias** | **Unclear risk of bias** | **Low risk of bias** | **Low risk of bias** | **Low risk of bias** | **Low risk of bias** |
| **Liu et al.（2025）** | **Low risk of bias** | **Low risk of bias** | **Low risk of bias** | **Low risk of bias** | **Low risk of bias** | **Low risk of bias** | **Low risk of bias** |
| **Livia et al.（2016）** | **Low risk of bias** | **Low risk of bias** | **Low risk of bias** | **Low risk of bias** | **Low risk of bias** | **Low risk of bias** | **Low risk of bias** |
| **Luo et al.（2024）** | **Low risk of bias** | **Low risk of bias** | **Low risk of bias** | **Low risk of bias** | **Low risk of bias** | **Low risk of bias** | **Low risk of bias** |
| **Ma et al.（2023）** | **Low risk of bias** | **Low risk of bias** | **Low risk of bias** | **Low risk of bias** | **Low risk of bias** | **Low risk of bias** | **Low risk of bias** |
| **Mansouri et al.（2019）** | **Low risk of bias** | **Unclear risk of bias** | **Low risk of bias** | **Low risk of bias** | **Low risk of bias** | **Low risk of bias** | **Low risk of bias** |
| **Meineake et al.（2014）** | **Low risk of bias** | **Unclear risk of bias** | **Low risk of bias** | **Low risk of bias** | **Low risk of bias** | **Low risk of bias** | **Low risk of bias** |
| **Min et al.（2023）** | **Low risk of bias** | **Low risk of bias** | **Unclear risk of bias** | **Low risk of bias** | **Low risk of bias** | **Low risk of bias** | **Low risk of bias** |
| **Mohamed et al.（2014）** | **Low risk of bias** | **Low risk of bias** | **Low risk of bias** | **Low risk of bias** | **Low risk of bias** | **Low risk of bias** | **Low risk of bias** |
| **Mu et al.（2017）** | **Low risk of bias** | **Low risk of bias** | **Low risk of bias** | **Low risk of bias** | **Low risk of bias** | **Low risk of bias** | **Low risk of bias** |
| **Nagz et al.（2020）** | **Low risk of bias** | **Low risk of bias** | **Low risk of bias** | **Low risk of bias** | **Low risk of bias** | **Low risk of bias** | **Low risk of bias** |
| **Ottens et al.（2014）** | **Low risk of bias** | **Low risk of bias** | **Low risk of bias** | **Low risk of bias** | **Low risk of bias** | **Low risk of bias** | **Low risk of bias** |
| **Qiao et al.（2023）** | **Low risk of bias** | **Unclear risk of bias** | **Low risk of bias** | **Low risk of bias** | **Low risk of bias** | **Low risk of bias** | **Low risk of bias** |
| **Racman et al.（2023）** | **Low risk of bias** | **Low risk of bias** | **Low risk of bias** | **Low risk of bias** | **Low risk of bias** | **Low risk of bias** | **Unclear risk of bias** |
| **Rohan et al.（2005）** | **Low risk of bias** | **Low risk of bias** | **Low risk of bias** | **Low risk of bias** | **Low risk of bias** | **Low risk of bias** | **Low risk of bias** |
| **Rörtgen et al.（2010）** | **Low risk of bias** | **Low risk of bias** | **High risk of bias** | **Low risk of bias** | **High risk of bias** | **Low risk of bias** | **Low risk of bias** |
| **Sun et al.（2024）** | **Low risk of bias** | **Low risk of bias** | **Low risk of bias** | **Low risk of bias** | **Low risk of bias** | **Low risk of bias** | **Low risk of bias** |
| **Takazawa et al.（2023）** | **Low risk of bias** | **Low risk of bias** | **Low risk of bias** | **Low risk of bias** | **Low risk of bias** | **Low risk of bias** | **Low risk of bias** |
| **Tanaka et al.（2017）** | **Low risk of bias** | **Low risk of bias** | **High risk of bias** | **Unclear risk of bias** | **Low risk of bias** | **Low risk of bias** | **Low risk of bias** |
| **Tian et al. (2015)** | **Low risk of bias** | **Low risk of bias** | **Low risk of bias** | **Unclear risk of bias** | **Low risk of bias** | **Low risk of bias** | **Low risk of bias** |
| **Varsha et al.（2024）** | **Low risk of bias** | **Low risk of bias** | **Low risk of bias** | **Low risk of bias** | **Low risk of bias** | **Low risk of bias** | **Low risk of bias** |
| **Verdonk et al.（2024）** | **Low risk of bias** | **Low risk of bias** | **Low risk of bias** | **Low risk of bias** | **Low risk of bias** | **Low risk of bias** | **Unclear risk of bias** |
| **Wang et al.（2002）** | **Low risk of bias** | **Low risk of bias** | **Low risk of bias** | **Low risk of bias** | **Low risk of bias** | **Low risk of bias** | **Low risk of bias** |
| **Wang et al.（2017）** | **Low risk of bias** | **Unclear risk of bias** | **Low risk of bias** | **Low risk of bias** | **Low risk of bias** | **Low risk of bias** | **Low risk of bias** |
| **Wang et al.（2018）** | **Low risk of bias** | **Low risk of bias** | **Low risk of bias** | **Low risk of bias** | **Low risk of bias** | **Low risk of bias** | **Low risk of bias** |
| **Wang et al.（2020）** | **Low risk of bias** | **Unclear risk of bias** | **Unclear risk of bias** | **Low risk of bias** | **Low risk of bias** | **Low risk of bias** | **Low risk of bias** |
| **Wen et al. (2024)** | **Low risk of bias** | **Low risk of bias** | **Low risk of bias** | **Unclear risk of bias** | **High risk of bias** | **Low risk of bias** | **Low risk of bias** |
| **Wittwer et al.（2023）** | **Low risk of bias** | **Low risk of bias** | **High risk of bias** | **Low risk of bias** | **Low risk of bias** | **Low risk of bias** | **Low risk of bias** |
| **Wu et al.（2024）** | **Low risk of bias** | **Low risk of bias** | **Low risk of bias** | **Unclear risk of bias** | **High risk of bias** | **Low risk of bias** | **Low risk of bias** |
| **Xu et al.（2013）** | **Low risk of bias** | **Low risk of bias** | **Low risk of bias** | **Low risk of bias** | **Low risk of bias** | **Low risk of bias** | **Low risk of bias** |
| **Xu et al.（2017）** | **Low risk of bias** | **Unclear risk of bias** | **Low risk of bias** | **Unclear risk of bias** | **Low risk of bias** | **Low risk of bias** | **Low risk of bias** |
| **Yang et al.（2015）** | **Low risk of bias** | **Low risk of bias** | **Low risk of bias** | **Low risk of bias** | **Low risk of bias** | **Low risk of bias** | **Low risk of bias** |
| **Zhang et al.（2015）** | **Low risk of bias** | **Low risk of bias** | **Low risk of bias** | **Low risk of bias** | **Low risk of bias** | **Low risk of bias** | **Low risk of bias** |
| **Zhang et al.（2018）** | **Low risk of bias** | **Low risk of bias** | **Low risk of bias** | **Low risk of bias** | **Low risk of bias** | **Low risk of bias** | **Low risk of bias** |
| **Zhang et al.（2019）** | **Low risk of bias** | **Unclear risk of bias** | **Low risk of bias** | **Low risk of bias** | **Low risk of bias** | **Low risk of bias** | **Low risk of bias** |
| **Zhang et al.（2021）** | **Low risk of bias** | **Unclear risk of bias** | **Low risk of bias** | **Low risk of bias** | **Low risk of bias** | **Low risk of bias** | **Low risk of bias** |
| **Zhao et al.（2020）** | **Low risk of bias** | **Unclear risk of bias** | **Low risk of bias** | **Low risk of bias** | **Low risk of bias** | **Low risk of bias** | **Low risk of bias** |
| **Zhou et al.（2019）** | **Low risk of bias** | **Low risk of bias** | **Low risk of bias** | **Low risk of bias** | **Low risk of bias** | **Low risk of bias** | **Low risk of bias** |
| **Zhu et al. (2021)** | **Low risk of bias** | **Low risk of bias** | **Low risk of bias** | **Unclear risk of bias** | **Low risk of bias** | **Low risk of bias** | **Low risk of bias** |
| **Zhu et al.（2016）** | **Low risk of bias** | **Unclear risk of bias** | **Low risk of bias** | **Low risk of bias** | **Low risk of bias** | **Low risk of bias** | **Low risk of bias** |
| **Zhu et al.（2024）** | **Low risk of bias** | **Low risk of bias** | **Low risk of bias** | **Low risk of bias** | **Low risk of bias** | **Low risk of bias** | **Low risk of bias** |

# APPENDIX 5 **Results of Gelman-Rubin Diagnostic, Effective Sample Size**

**5.1 Anesthesia maintenance agents**

| QOR score： | | |
| --- | --- | --- |
| Rhat: | | |
| Parameter | Point est. | Upper C.I. |
| d.MDZ.NS | 1.004 | 1.012 |
| [d.MDZ.PRO](https://d.mdz.pro/) | 1.001 | 1.001 |
| d.MDZ.RMZ | 1.004 | 1.011 |
| d.PRO.DES | 1.003 | 1.009 |
| d.PRO.ISO | 1.000 | 1.001 |
| d.PRO.SEVO | 1.002 | 1.004 |
| sd.d | 1.006 | 1.020 |

| ESS: | |
| --- | --- |
| parameter | |
| d.MDZ.NS | 1338.84 |
| d.MDZ.PRO | 3723.92 |
| d.MDZ.RMZ | 999.14 |
| d.PRO.DES | 2064.39 |
| d.PRO.ISO | 2903.79 |
| d.PRO.SEVO | 3187.59 |
| sd.d | 746.20 |

| Model fit (residual deviance) | | |
| --- | --- | --- |
| Dbar | pD | DIC |
| 44.30205 | 31.30225 | 75.60430 |
| 42 data points | ratio | I² |
|  | 1.055 | 7% |

**5.2 Adjuvant sedative and neuroprotective agents**

| QOR score： | | |
| --- | --- | --- |
| Rhat: | | |
| Parameter | Point est. | Upper C.I. |
| d.NS.DEXA | 1.000 | 1.000 |
| d.NS.DEXM | 1.000 | 1.000 |
| d.NS.KET | 1.001 | 1.002 |
| d.NS.MINO | 1.000 | 1.001 |
| d.NS.UTI | 1.000 | 1.001 |
| sd.d | 1.004 | 1.010 |

| ESS: | |
| --- | --- |
| parameter | |
| d.NS.DEXA | 11990.61 |
| d.NS.DEXM | 5277.07 |
| d.NS.KET | 7521.48 |
| d.NS.MINO | 8859.15 |
| d.NS.UTI | 9005.82 |
| sd.d | 1795.36 |

| Model fit (residual deviance) | | |
| --- | --- | --- |
| Dbar | pD | DIC |
| 86.47513 | 61.36306 | 147.83819 |
| 80 data points | ratio | I² |
|  | 1.081 | 9% |

**5.3 Analgesic agents**

| QOR score： | | |
| --- | --- | --- |
| Rhat: | | |
| Parameter | Point est. | Upper C.I. |
| d.NS.DEXA | 1.000 | 1.000 |
| d.NS.DEXM | 1.000 | 1.001 |
| d.NS.KET | 1.001 | 1.002 |
| d.NS.MINO | 1.000 | 1.001 |
| d.NS.UTI | 1.000 | 1.001 |
| sd.d | 1.000 | 1.012 |

| ESS: | |
| --- | --- |
| parameter | |
| d.NS.DEXA | 5865 |
| d.NS.DEXM | 6268 |
| d.NS.KET | 7860 |
| d.NS.MINO | 6941 |
| d.NS.UTI | 7135 |
| sd.d | 6669 |

| Model fit (residual deviance) | | |
| --- | --- | --- |
| Dbar | pD | DIC |
| 40.25911 | 20.35064 | 60.60976 |
| 28 data points | ratio | I² |
|  | 1.438 | 33% |

# APPENDIX 6 **Results from pairwise meta-analysis**

**6.1 Paired meta-analysis summary table of anesthesia maintenance drugs and dNCR outcomes**

|  | **N*** | **n*** | **OR（(95% CI)** | **tau^2** | **I^2** | **P-value** |
| --- | --- | --- | --- | --- | --- | --- |
| **Desflurane vs. Sevoflurane** | 5 | 353 | 0.54 [0.28; 1.02] | <0.0001 | 0.0% | 0.76 |
| **Propofol vs. Sevoflurane** | 8 | 1435 | 0.66 [0.51; 0.86] | <0.0001 | 5.5% | 0.39 |
| **Propofol vs. Isoflurane** | 1 | 100 | 0.10 [0.02; 0.45] | / | / | / |
| **Sevoflurane vs. Isoflurane** | 1 | 100 | 0.58 [0.23; 1.46] | / | / | / |
| **Propofol vs. Midazolam** | 1 | 109 | 0.21 [0.09; 0.49] | / | / | / |
| **Midazolam vs. Normal Saline** | 1 | 100 | 0.35 [0.10; 1.20] | / | / | / |
| **Remimazolam vs. Normal Saline** | 1 | 69 | 0.24 [0.06; 0.97] | / | / | / |
| **Propofol vs. Desflurane** | 2 | 142 | 0.48 [0.21; 1.11] | <0.0001 | 0.0% | 0.68 |
| **Remimazolam vs. Midazolam** | 1 | 70 | 0.19 [0.01; 4.08] | / | / | / |
|  | 21 | 2478 |  |  |  |  |
| **Random effects model** |  |  | 0.51 [0.40 0.66] | 0.07 | 15.6% | 0.26 |

*** N= number of studies; n= number of patients.**

**6.2 Pairwise meta-analysis summary table of sedative-assistance and neuroprotective drugs and dNCR outcomes**

**6.3 Pairwise meta-analysis summary table of sedative-assistance and neuroprotective drugs and dNCR outcomes**

|  | **N*** | **N*** | **OR（(95% CI)** | **tau^2** | **I^2** | **P-value** |
| --- | --- | --- | --- | --- | --- | --- |
| **Dexmedetomidine vs. Normal Saline** | 22 | 2044 | 0.34 [0.25; 0.47] | 0.17 | 30.9% | 0.08 |
| **Ketamine vs. Normal Saline** | 6 | 665 | 0.90 [0.45; 1.78] | 0.36 | 72.8% | 0.01 |
| **Ulinsatatin vs. Normal Saline** | 4 | 292 | 0.60 [0.32; 1.14] | 0.30 | 50.9% | 0.07 |
| **Dexamethasone vs. Normal Saline** | 4 | 1533 | 0.32 [0.18; 0.58] | ＜0.0001 | 0% | 0.42 |
| **Dexmedetomidine vs. Ketamine** | 1 | 40 | 2.22 [0.64; 7.65] | / | / | / |
| **Dexmedetomidine vs. Ulinsatatin** | 1 | 77 | 0.18 [0.01; 4.01] | / | / | / |
| **Minocycline vs. Normal Saline** | 1 | 185 | 0.86 [0.26; 2.83] | / | / | / |
|  | 39 | 4836 |  |  |  |  |
| **Random effects model** |  |  | 0.45 [0.34; 0.58] | 0.37 | 61.2% | ＜0.0001 |

*** N= number of studies; n= number of patients.**

**6.4 Forest plot of sedative-assistance drugs and neuroprotective drugs and their results with dNCR**

**6.5 Pairwise meta-analysis summary table of analgesic drug and dNCR outcomes**

|  | **N*** | **n*** | **OR（(95% CI)** | **tau^2** | **I^2** | **P-value** |
| --- | --- | --- | --- | --- | --- | --- |
| **Lidocaine vs. Normal Saline** | 3 | 421 | 0.34 [0.13; 0.92] | 0.55 | 65.3% | 0.06 |
| **Ropivacaine vs. Normal Saline** | 2 | 107 | 0.45 [0.10; 2.03] | 0.88 | 57.6% | 0.12 |
| **Parecoxib vs. Normal Saline** | 3 | 815 | 0.42 [0.31; 0.57] | 0.00 | 0% | 0.55 |
| **Butorphanol vs. Normal Saline** | 1 | 130 | 0.45 [0.19; 1.11] | / | / | / |
| **Fentanyl vs. Normal Saline** | 1 | 130 | 0.92 [0.42; 2.03] | / | / | / |
|  | 10 | 1603 |  |  |  |  |
| **Random effects model** |  |  | 0.43 [0.31; 0.59] | 0.14 | 44.6% | 0.06 |

*** N= number of studies; n= number of patients.**

**6.6 Forest plot of analgesic drug and their results with dNCR**

# APPENDIX 7 **SUCRA Values and Ranking Probabilities of included studies**

**7.1 Anesthesia maintenance drugs ranked by probability and SUCRA value-based intervention measures**

| **Treatment** | **Rank** | **PrBest** | **SUCRA** | **MeanRank** |
| --- | --- | --- | --- | --- |
| **Propofol** | 1 | 1.5 | 0.908 | 3.21 |
| **Desflurane** | 2 | 15.8 | 0.773 | 4.68 |
| **Remimazolam** | 3 | 0.4 | 0.657 | 3.14 |
| **Sevoflurane** | 4 | 0.1 | 0.587 | 3.61 |
| **Isoflurane** | 5 | 43.7 | 0.259 | 3.45 |
| **Midazolam** | 6 | 31.4 | 0.258 | 4.72 |
| **Normal Saline** | 7 | 7.1 | 0.058 | 5.18 |

**7.2 Graph of sedative maintenance drugs (sucra column chart)**

**7.3 Adjuvant sedative and neuroprotective agents ranked by probability and SUCRA value-based intervention measures**

| **Treatment** | **Rank** | **PrBest** | **SUCRA** | **MeanRank** |
| --- | --- | --- | --- | --- |
| Dexmedetomidine | 1 | 0.9 | 0.887 | 3.67 |
| Ulinsatatin | 2 | 8.9 | 0.886 | 3.82 |
| Ketamine | 3 | 16.3 | 0.557 | 2.94 |
| Dexamethasone | 4 | 0.1 | 0.334 | 4.11 |
| Normal Saline | 5 | 33.8 | 0.257 | 3.49 |
| Minocycline | 6 | 40.0 | 0.080 | 2.98 |

**7.4 Schematic diagram of adjunctive sedatives and neuroprotective agents**

**7.5 Analgesic agents ranked by probability and SUCRA value-based intervention measures**

| **Treatment** | **Rank** | **PrBest** | **SUCRA** | **MeanRank** |
| --- | --- | --- | --- | --- |
| **Ropivacaine** | 1 | 35.6 | 0.776 | 2.34 |
| **Lidocaine** | 2 | 27.2 | 0.748 | 2.51 |
| **Parecoxib** | 3 | 18.2 | 0.696 | 2.82 |
| **Butorphanol** | 4 | 17.7 | 0.671 | 2.97 |
| **Sufentanil** | 5 | 1.2 | 0.324 | 4.06 |
| **Fentanyl** | 6 | 0.3 | 0.197 | 4.82 |
| **Normal Saline** | 7 | 0.0 | 0.087 | 5.48 |

**7.6 Schematic diagram of analgesic agents**

# APPENDIX 8 **Heterogeneity and Inconsistency Assessment by Bayesian network meta analysis**

**8.1.1 Anesthesia maintenance agents :Assessment of Heterogeneity and Inconsistency**

| **Component** | **Parameter** | **Estimate (95% CrI)** | **Interpretation** |
| --- | --- | --- | --- |
| **Heterogeneity Parameters** | τ² | 0.107 (0.0002, 0.758) | Low between-study heterogeneity |
|  | I² | 8.0% | Minor heterogeneity present |
| ****Model Fit Statistics**** |  |  |  |
| **- Consistency Model** | Dbar | 44.37 | Data fit (lower is better) |
|  | pD | 31.76 | Effective number of parameters |
|  | DIC | 76.13 | Deviance Information Criterion |
| **- Inconsistency Model** | Dbar | 44.26 | Data fit (lower is better) |
|  | pD | 31.56 | Effective number of parameters |
|  | DIC | 75.81 | Deviance Information Criterion |
| ****Model Comparison**** | ΔDIC | -0.31 |  |
|  | Bayes Factor (for consistency) | 1.17 |  |
|  | Posterior Model Probability |  |  |
|  | - Consistency model | 53.9% |  |
|  | - Inconsistency model | 46.1% |  |
| ****Overall Conclusion**** |  |  | No significant global inconsistency detected (ΔDIC< 2) |

**8.1.2 Anesthesia maintenance agents:Node-Splitting Analysis for Local Inconsistency on anesthesia maintenance agents**

| **Comparison** | **K*** | **Direct OR (95% CrI)*** | **Indirect OR (95% CrI)*** | **NMA OR (95% CrI)*** | **RoR (95% CrI)*** | **p-value*** |
| --- | --- | --- | --- | --- | --- | --- |
| DES vs PRO | 2 | 2.10 (0.88, 5.02) | 0.85 (0.42, 1.73) | 1.22 (0.69, 2.15) | 2.48 (0.81, 7.57) | 0.113 |
| DES vs SEVO | 5 | 0.54 (0.28, 1.04) | 1.34 (0.54, 3.34) | 0.74 (0.38, 1.26) | 0.40 (0.14, 1.17) | 0.113 |
| ISO vs PRO | 1 | 10.29 (2.17, 48.84) | 2.76 (1.03, 7.42) | 4.03 (1.69, 9.58) | 3.73 (0.63, 22.05) | 0.162 |
| ISO vs SEVO | 1 | 1.71 (0.66, 4.44) | 6.39 (1.31, 31.05) | 2.43 (1.07, 5.52) | 0.27 (0.04, 1.69) | 0.162 |
| MDZ vs NS | 1 | 0.35 (0.10, 1.22) | 1.28 (0.04, 38.10) | 0.41 (0.13, 1.33) | 0.27 (0.07, 1.07) | 0.480 |
| MDZ vs RMZ | 1 | 5.30 (0.24, 115.59) | 1.44 (0.22, 9.55) | 2.06 (0.41, 10.34) | 3.69 (0.54, 25.15) | 0.480 |
| RMZ vs NS | 1 | 0.24 (0.06, 115.59) | 0.07 (0.00, 1.83) | 0.20 (0.05, 0.73) | 3.69 (0.54, 25.15) | 0.480 |
| PRO vs SEVO | 8 | 0.66 (0.50, 0.87) | 0.23 (0.09, 0.58) | 0.60 (0.44, 0.82) | 2.87 (1.08, 7.64) | ****0.034**** |

*Note: OR = Odds Ratio; CrI = Credible Interval; RoR = Ratio of Odds Ratios (Direct/Indirect); k = number of studies. Bold indicates potential local inconsistency (p < 0.05).*

**8.1.3 Anesthesia maintenance agents:Pairwise comparison forest plot**

**8.2.1 Adjuvant sedative and neuroprotective agents:Assessment of Heterogeneity and Inconsistency**

| **Component** | **Parameter** | **Estimate (95% CrI)** | **Interpretation** |
| --- | --- | --- | --- |
| ****Heterogeneity Parameters**** | τ² | 0.358 (0.084, 0.926) | Moderate between-study heterogeneity |
|  | I² | 9.0% | Low heterogeneity |
| ****Model Fit Statistics**** |  |  |  |
| - Consistency Model | Dbar | 86.43 | Data fit |
|  | pD | 61.58 | Effective number of parameters |
|  | ****DIC**** | ****148.02**** | Deviance Information Criterion |
| - Inconsistency Model | Dbar | 86.58 | Data fit |
|  | pD | 61.41 | Effective number of parameters |
|  | ****DIC**** | ****148.00**** | Deviance Information Criterion |
| ****Model Comparison**** | ΔDIC | -0.02 |  |
|  | Bayes Factor (for consistency) | 1.01 |  |
|  | Posterior Model Probability: |  |  |
|  | - Consistency model | 50.2% |  |
|  | - Inconsistency model | 49.8% |  |
| ****Overall Conclusion**** |  |  | No significant global inconsistency detected (ΔDIC< 2) |

**8.2.2 Adjuvant sedative and neuroprotective agents:Node-Splitting Analysis for Local Inconsistency on anesthesia maintenance agents**

| **Comparison** | **K*** | **Direct OR (95% CrI)*** | **Indirect OR (95% CrI)*** | **NMA OR (95% CrI)*** | **RoR (95% CrI)*** | **p-value*** |
| --- | --- | --- | --- | --- | --- | --- |
| **DEXM vs KET** | 1 | 0.18 (0.01, 4.49) | 0.52 (0.28, 1.03) | 0.52 (0.27, 0.97) | 0.34 (0.02, 7.04) | 0.513 |
| **DEXM vs NS** | 23 | 0.34 (0.25, 0.47) | 0.22 (0.05, 0.95) | 0.34 (0.24, 0.46) | 1.56 (0.83, 2.95) | 0.560 |
| **DEXM vs UTI** | 1 | 0.86 (0.20, 3.70) | 1.12 (0.50, 2.53) | 1.05 (0.52, 2.14) | 0.76 (0.09, 6.26) | 0.752 |
| **KET vs NS** | 6 | 0.63 (0.36, 1.10) | 1.88 (0.07, 47.35) | 0.65 (0.37, 1.14) | 0.34 (0.06, 1.78) | 0.513 |
| **UTI vs NS** | 4 | 0.30 (0.14, 0.64) | 0.39 (0.09, 1.76) | 0.32 (0.16, 0.62) | 0.76 (0.16, 3.62) | 0.752 |

*Note: OR = Odds Ratio; CrI = Credible Interval; RoR = Ratio of Odds Ratios (Direct/Indirect); k = number of studies. Bold indicates potential local inconsistency (p < 0.05).*

**8.2.3 Adjuvant sedative and neuroprotective agents:Pairwise comparison forest plot**

**8.3.1 Analgesic agents:Pairwise comparison forest plot**

# APPENDIX 9 **Credibility assessment of the odds ratio (OR) for the incidence rate of dNCR based on the CINeMA framework**

**9.1 Anesthesia maintenance drugs**

| **Comparison** | **Within-study bias** | **Reporting bias** | **Indirectness** | **Imprecision** | **Heterogeneity** | **Incoherence** | **Confidence rating** | **Reason**  **(s) for downgrading** |
| --- | --- | --- | --- | --- | --- | --- | --- | --- |
| **Desflurane:Propofol** | **Some concerns** | **Some concerns** | **No concerns** | **Major concerns** | **No concerns** | **No concerns** | **Low** | **-1 within-study bias, -1 reporting bias, -2 imprecision** |
| **Desflurane:Sevoflurane** | **Some concerns** | **Some concerns** | **No concerns** | **Major concerns** | **No concerns** | **No concerns** | **Low** | **-1 within-study bias, -1 reporting bias, -2 imprecision** |
| **Isoflurane:Propofol** | **Some concerns** | **Some concerns** | **No concerns** | **No concerns** | **No concerns** | **No concerns** | **Moderate** | **-1 within-study bias, -1 reporting bias** |
| **Isoflurane:Sevoflurane** | **Some concerns** | **Some concerns** | **No concerns** | **No concerns** | **No concerns** | **No concerns** | **Moderate** | **-1 within-study bias, -1 reporting bias** |
| **Midazolam:NS** | **Some concerns** | **Some concerns** | **No concerns** | **Major concerns** | **No concerns** | **No concerns** | **Low** | **-1 within-study bias, -1 reporting bias, -2 imprecision** |
| **Midazolam:Propofol** | **Major concerns** | **Some concerns** | **No concerns** | **No concerns** | **No concerns** | **No concerns** | **Low** | **-2 within-study bias, -1 reporting bias** |
| **Midazolam:Remimazolam** | **Some concerns** | **Some concerns** | **No concerns** | **Major concerns** | **No concerns** | **No concerns** | **Low** | **-1 within-study bias, -1 reporting bias, -2 imprecision** |
| **NS:Remimazolam** | **Some concerns** | **Some concerns** | **No concerns** | **No concerns** | **No concerns** | **No concerns** | **Moderate** | **-1 within-study bias, -1 reporting bias** |
| **Propofol:Sevoflurane** | **No concerns** | **Some concerns** | **No concerns** | **No concerns** | **No concerns** | **No concerns** | **High** | **-1 reporting bias** |
| **Desflurane:Isoflurane** | **Some concerns** | **High risk** | **No concerns** | **No concerns** | **No concerns** | **No concerns** | **Low** | **-1 within-study bias, -2 reporting bias** |
| **Desflurane:Midazolam** | **Some concerns** | **High risk** | **No concerns** | **No concerns** | **No concerns** | **No concerns** | **Low** | **-1 within-study bias, -2 reporting bias** |
| **Desflurane:NS** | **Some concerns** | **High risk** | **No concerns** | **No concerns** | **No concerns** | **No concerns** | **Low** | **-1 within-study bias, -2 reporting bias** |
| **Desflurane:Remimazolam** | **Some concerns** | **High risk** | **No concerns** | **Major concerns** | **No concerns** | **No concerns** | **Very Low** | **-1 within-study bias, -2 reporting bias, -2 imprecision** |
| **Isoflurane:Midazolam** | **Some concerns** | **High risk** | **No concerns** | **Major concerns** | **No concerns** | **No concerns** | **Very Low** | **-1 within-study bias, -2 reporting bias, -2 imprecision** |
| **Isoflurane:NS** | **Some concerns** | **High risk** | **No concerns** | **Major concerns** | **No concerns** | **No concerns** | **Very Low** | **-1 within-study bias, -2 reporting bias, -2 imprecision** |
| **Isoflurane:Remimazolam** | **Some concerns** | **High risk** | **No concerns** | **Major concerns** | **No concerns** | **No concerns** | **Very Low** | **-1 within-study bias, -2 reporting bias, -2 imprecision** |
| **Midazolam:Sevoflurane** | **Some concerns** | **High risk** | **No concerns** | **No concerns** | **No concerns** | **No concerns** | **Low** | **-1 within-study bias, -2 reporting bias** |
| **NS:Propofol** | **Major concerns** | **Some concerns** | **No concerns** | **No concerns** | **No concerns** | **No concerns** | **Low** | **-2 within-study bias, -1 reporting bias** |
| **NS:Sevoflurane** | **Some concerns** | **High risk** | **No concerns** | **No concerns** | **No concerns** | **No concerns** | **Low** | **-1 within-study bias, -2 reporting bias** |
| **Propofol:Remimazolam** | **Major concerns** | **High risk** | **No concerns** | **Major concerns** | **No concerns** | **No concerns** | **Very Low** | **-2 within-study bias, -2 reporting bias, -2 imprecision** |
| **Remimazolam:Sevoflurane** | **Some concerns** | **High risk** | **No concerns** | **Major concerns** | **No concerns** | **No concerns** | **Very Low** | **-1 within-study bias, -2 reporting bias, -2 imprecision** |

## **9.2 Adjuvant sedative and neuroprotective agents**

| **Comparison** | **Within-study bias** | **Reporting bias** | **Indirectness** | **Imprecision** | **Heterogeneity** | **Incoherence** | **Confidence rating** | **Reason**  **(s) for downgrading** |
| --- | --- | --- | --- | --- | --- | --- | --- | --- |
| **Dexamethasone:Ketamine** | **No concerns** | **High risk** | **No concerns** | **Major concerns** | **No concerns** | **No concerns** | **Low** | **-1 reporting bias, -1 imprecision** |
| **Dexamethasone:Normal Saline** | **No concerns** | **High risk** | **No concerns** | **Major concerns** | **No concerns** | **Major concerns** | **Very low** | **-1 reporting bias, -1 imprecision, -1 incoherence** |
| **Dexmedetomidine:Normal Saline** | **Some concerns** | **Low risk** | **No concerns** | **No concerns** | **No concerns** | **Major concerns** | **Low** | **-1 within-study bias, -1 incoherence** |
| **Dexmedetomidine:Treatment** | **Some concerns** | **High risk** | **No concerns** | **Major concerns** | **No concerns** | **Major concerns** | **Very low** | **-1 within-study bias, -1 reporting bias, -1 imprecision, -1 incoherence** |
| **Ketamine:Normal Saline** | **No concerns** | **Low risk** | **No concerns** | **Major concerns** | **No concerns** | **No concerns** | **Moderate** | **-1 imprecision** |
| **Minocycline:Normal Saline** | **No concerns** | **Low risk** | **No concerns** | **Major concerns** | **No concerns** | **Major concerns** | **Low** | **-1 imprecision, -1 incoherence** |
| **Normal Saline:Ulinastatin** | **No concerns** | **Low risk** | **No concerns** | **No concerns** | **Major concerns** | **Major concerns** | **Low** | **-1 heterogeneity, -1 incoherence** |
| **Dexamethasone:Dexmedetomidine** | **No concerns** | **High risk** | **No concerns** | **No concerns** | **Major concerns** | **Major concerns** | **Very low** | **-1 reporting bias, -1 heterogeneity, -1 incoherence** |
| **Dexamethasone:Minocycline** | **No concerns** | **High risk** | **No concerns** | **Major concerns** | **No concerns** | **No concerns** | **Moderate** | **-1 reporting bias, -1 imprecision** |
| **Dexamethasone:Treatment** | **Some concerns** | **High risk** | **No concerns** | **Major concerns** | **No concerns** | **No concerns** | **Very low** | **-1 within-study bias, -1 reporting bias, -1 imprecision** |
| **Dexamethasone:Ulinastatin** | **No concerns** | **High risk** | **No concerns** | **No concerns** | **Major concerns** | **Major concerns** | **Very low** | **-1 reporting bias, -1 heterogeneity, -1 incoherence** |
| **Dexmedetomidine:Ketamine** | **No concerns** | **High risk** | **No concerns** | **No concerns** | **Major concerns** | **Major concerns** | **Very low** | **-1 reporting bias, -1 heterogeneity, -1 incoherence** |
| **Dexmedetomidine:Minocycline** | **No concerns** | **High risk** | **No concerns** | **No concerns** | **Major concerns** | **Major concerns** | **Very low** | **-1 reporting bias, -1 heterogeneity, -1 incoherence** |
| **Dexmedetomidine:Ulinastatin** | **No concerns** | **High risk** | **No concerns** | **Major concerns** | **No concerns** | **No concerns** | **Moderate** | **-1 reporting bias, -1 imprecision** |
| **Ketamine:Minocycline** | **No concerns** | **High risk** | **No concerns** | **Major concerns** | **No concerns** | **No concerns** | **Moderate** | **-1 reporting bias, -1 imprecision** |
| **Ketamine:Treatment** | **Some concerns** | **High risk** | **No concerns** | **Major concerns** | **No concerns** | **No concerns** | **Very low** | **-1 within-study bias, -1 reporting bias, -1 imprecision** |
| **Ketamine:Ulinastatin** | **No concerns** | **High risk** | **No concerns** | **No concerns** | **Major concerns** | **Major concerns** | **Very low** | **-1 reporting bias, -1 heterogeneity, -1 incoherence** |
| **Minocycline:Treatment** | **Some concerns** | **High risk** | **No concerns** | **Major concerns** | **No concerns** | **No concerns** | **Very low** | **-1 within-study bias, -1 reporting bias, -1 imprecision** |
| **Minocycline:Ulinastatin** | **No concerns** | **High risk** | **No concerns** | **No concerns** | **Major concerns** | **Major concerns** | **Very low** | **-1 reporting bias, -1 heterogeneity, -1 incoherence** |
| **Normal Saline:Treatment** | **Some concerns** | **High risk** | **No concerns** | **Major concerns** | **No concerns** | **No concerns** | **Very low** | **-1 within-study bias, -1 reporting bias, -1 imprecision** |
| **Treatment:Ulinastatin** | **Some concerns** | **High risk** | **No concerns** | **Major concerns** | **No concerns** | **No concerns** | **Very low** | **-1 within-study bias, -1 reporting bias, -1 imprecision** |

## **9.3 Analgesic drug**

| **Comparison** | **Within-study bias** | **Reporting bias** | **Indirectness** | **Imprecision** | **Heterogeneity** | **Incoherence** | **Confidence rating** | **Reason**  **(s) for downgrading** |
| --- | --- | --- | --- | --- | --- | --- | --- | --- |
| **Butorphanol : Fentanyl** | **No concerns** | **Low risk** | **No concerns** | **Major concerns** | **No concerns** | **No concerns** | **Moderate** | **Imprecision (-1)** |
| **Butorphanol : Normal saline** | **No concerns** | **Low risk** | **No concerns** | **Major concerns** | **No concerns** | **No concerns** | **Moderate** | **Imprecision (-1)** |
| **Butorphanol : Sufentanil** | **Some concerns** | **Low risk** | **No concerns** | **Major concerns** | **No concerns** | **No concerns** | **Low** | **Within-study bias (-1), Imprecision (-1)** |
| **Fentanyl : Normal saline** | **No concerns** | **Low risk** | **No concerns** | **Major concerns** | **No concerns** | **No concerns** | **Moderate** | **Imprecision (-1)** |
| **Fentanyl : Sufentanil** | **Some concerns** | **Low risk** | **No concerns** | **Major concerns** | **No concerns** | **No concerns** | **Low** | **Within-study bias (-1), Imprecision (-1)** |
| **Lidocaine : Normal saline** | **No concerns** | **Low risk** | **No concerns** | **No concerns** | **Major concerns** | **Major concerns** | **Low** | **Heterogeneity (-1), Incoherence (-1)** |
| **Normal saline : Parecoxib** | **Some concerns** | **Low risk** | **No concerns** | **No concerns** | **Major concerns** | **Major concerns** | **Very low** | **Within-study bias (-1), Heterogeneity (-1), Incoherence (-1)** |
| **Normal saline : Ropivacaine** | **Some concerns** | **Low risk** | **No concerns** | **Major concerns** | **No concerns** | **No concerns** | **Low** | **Within-study bias (-1), Imprecision (-1)** |
| **Ropivacaine : Sufentanil** | **No concerns** | **Low risk** | **No concerns** | **Major concerns** | **No concerns** | **No concerns** | **Moderate** | **Imprecision (-1)** |
| **Butorphanol : Lidocaine** | **No concerns** | **Low risk** | **No concerns** | **Major concerns** | **No concerns** | **Major concerns** | **Very low** | **Imprecision (-1), Incoherence (-1)** |
| **Butorphanol : Parecoxib** | **No concerns** | **Low risk** | **No concerns** | **Major concerns** | **No concerns** | **Major concerns** | **Very low** | **Imprecision (-1), Incoherence (-1)** |
| **Butorphanol : Ropivacaine** | **Some concerns** | **Low risk** | **No concerns** | **Major concerns** | **No concerns** | **Major concerns** | **Very low** | **Within-study bias (-1), Imprecision (-1), Incoherence (-1)*** |
| **Fentanyl : Lidocaine** | **No concerns** | **Low risk** | **No concerns** | **Major concerns** | **No concerns** | **Major concerns** | **Very low** | **Imprecision (-1), Incoherence (-1)** |
| **Fentanyl : Parecoxib** | **No concerns** | **Low risk** | **No concerns** | **Major concerns** | **No concerns** | **Major concerns** | **Very low** | **Imprecision (-1), Incoherence (-1)** |
| **Fentanyl : Ropivacaine** | **Some concerns** | **Low risk** | **No concerns** | **Major concerns** | **No concerns** | **Major concerns** | **Very low** | **Within-study bias (-1), Imprecision (-1), Incoherence (-1)*** |
| **Lidocaine : Parecoxib** | **No concerns** | **Low risk** | **No concerns** | **Major concerns** | **No concerns** | **Major concerns** | **Very low** | **Imprecision (-1), Incoherence (-1)** |
| **Lidocaine : Ropivacaine** | **Some concerns** | **Low risk** | **No concerns** | **Major concerns** | **No concerns** | **Major concerns** | **Very low** | **Within-study bias (-1), Imprecision (-1), Incoherence (-1)*** |
| **Lidocaine : Sufentanil** | **Some concerns** | **Low risk** | **No concerns** | **Major concerns** | **No concerns** | **Major concerns** | **Very low** | **Within-study bias (-1), Imprecision (-1), Incoherence (-1)*** |
| **Normal saline : Sufentanil** | **Some concerns** | **Low risk** | **No concerns** | **Major concerns** | **No concerns** | **Major concerns** | **Very low** | **Within-study bias (-1), Imprecision (-1), Incoherence (-1)*** |
| **Parecoxib : Ropivacaine** | **Some concerns** | **Low risk** | **No concerns** | **Major concerns** | **No concerns** | **Major concerns** | **Very low** | **Within-study bias (-1), Imprecision (-1), Incoherence (-1)*** |
| **Parecoxib : Sufentanil** | **Some concerns** | **Low risk** | **No concerns** | **Major concerns** | **No concerns** | **Major concerns** | **Very low** | **Within-study bias (-1), Imprecision (-1), Incoherence (-1)*** |

*Note: For comparisons without direct research evidence (i.e., no head-to-head comparisons), the Imprecision and Incoherence markers being labeled as "Major concerns" are based on theoretical assumptions rather than actual analyzable data.

# APPENDIX 10 Sensitivity analysis

## ****10.1.1** Anesthesia maintenance agents :**Summary of Leave-One-Out Sensitivity Analysis****

| Study Omitted | Pooled Estimate (RR) | 95% Confidence Interval |
| --- | --- | --- |
| ****Chen et al. 2001**** | 0.983 | 0.658 – 1.469 |
| ****Deepak et al. 2013**** | 0.983 | 0.658 – 1.469 |
| ****Egawa et al. 2016**** | 0.955 | 0.627 – 1.454 |
| ****Geng et al. 2017 (1)**** | 0.935 | 0.631 – 1.387 |
| ****Geng et al. 2017 (2)**** | 1.103 | 0.760 – 1.599 |
| ****Geng et al. 2017 (3)**** | 1.034 | 0.684 – 1.561 |
| ****Guo et al. 2020**** | 0.985 | 0.637 – 1.521 |
| ****Kletecka et al. 2019**** | 1.004 | 0.666 – 1.512 |
| ****Kuzminskaite et al. 2023**** | 1.008 | 0.675 – 1.507 |
| ****Li et al. 2019 (3)**** | 1.138 | 0.796 – 1.627 |
| ****Li et al. 2021**** | 0.968 | 0.622 – 1.507 |
| ****Mansouri et al. 2019 (2)**** | 0.946 | 0.631 – 1.420 |
| ****Meineake et al. 2014**** | 0.939 | 0.624 – 1.413 |
| ****Min et al. 2023 (2)**** | 1.068 | 0.720 – 1.583 |
| ****Qiao et al. 2023**** | 1.026 | 0.687 – 1.532 |
| ****Rohan et al. 2005**** | 0.997 | 0.661 – 1.504 |
| ****Rörtgen et al. 2010**** | 0.982 | 0.647 – 1.490 |
| ****Tanaka et al. 2017**** | 1.044 | 0.694 – 1.572 |
| ****Varsha et al. 2024**** | 0.936 | 0.624 – 1.403 |
| ****Wu et al. 2024**** | 1.023 | 0.687 – 1.524 |
| ****Zhang et al. 2018**** | 0.949 | 0.619 – 1.456 |
| ****Combined (All Studies)**** | ****0.999**** | ****0.672 – 1.484**** |

## ****10.1.2** Anesthesia maintenance agents :**Summary of Meta-Analysis Results by Model****

| ****Component**** | ****Random-Effects Model**** | ****Fixed-Effect Model**** |
| --- | --- | --- |
| ****Pooled OR (95% CI)**** | 0.999 (0.672 – 1.484) | 1.126 (0.915 – 1.385) |
| ****Test of Overall Effect (Z, p-value)**** | -0.006, p = 0.995 | 1.119, p = 0.263 |
| ****Heterogeneity Metrics**** |  |  |
| **• Cochran's Q (p-value)** | 55.27 (p < 0.001) | 55.27 (p < 0.001) |
| **• I² (95% CI)** | 63.8% (10.2% – 80.6%) | 63.8% (36.8% – 76.2%) |
| **• Tau² (τ²)** | 0.4396 | – |
| ****Model Comparison**** |  |  |
| **• Absolute Difference in OR** | – | 0.127 |
| **• Relative Difference in OR** | – | 12.7% |

## ****10.1.3** Anesthesia maintenance agents :**Analysis After Outlier Removal (IQR Method)****

| ****Parameter**** | ****Result**** |
| --- | --- |
| ****Number of Studies Included**** | 21 |
| ****Pooled Odds Ratio (OR)**** | 0.999 |
| ****95% Confidence Interval**** | 0.672 – 1.484 |
| ****Test of Overall Effect**** | Z = -0.006, p = 0.995 |
| ****Heterogeneity Statistics**** |  |
| • Cochran's Q | 55.27 (df=20), p < 0.001 |
| • I² | 63.8% (95% CI: 10.2% – 80.6%) |
| • Tau² (τ²) | 0.4396 |

****10.2.1** Adjuvant sedative and neuroprotective agents:Summary of Leave-One-Out Sensitivity Analysis**

| Study Omitted | Pooled Estimate (RR) | 95% Confidence Interval |
| --- | --- | --- |
| ****Chen et al. 2013**** | 1.806 | 1.321 – 2.471 |
| ****Chen et al. 2015**** | 1.794 | 1.314 – 2.450 |
| ****Chen et al. 2020**** | 1.707 | 1.269 – 2.295 |
| ****Cheng et al. 2021**** | 1.789 | 1.310 – 2.442 |
| ****Fang et al. 2014**** | 1.884 | 1.374 – 2.584 |
| ****Gao et al. 2020**** | 1.781 | 1.313 – 2.417 |
| ****Gao et al. 2021**** | 1.772 | 1.304 – 2.407 |
| ****Ghazaly et al. 2023 (1)**** | 1.780 | 1.309 – 2.421 |
| ****Ghazaly et al. 2023 (2)**** | 1.771 | 1.308 – 2.399 |
| ****Ghazaly et al. 2023 (3)**** | 1.798 | 1.323 – 2.444 |
| ****Glumac et al. 2017**** | 1.791 | 1.311 – 2.446 |
| ****Gong et al. 2018**** | 1.759 | 1.298 – 2.383 |
| ****He et al. 2020 (1)**** | 1.824 | 1.336 – 2.490 |
| ****Hollinger et al. 2021**** | 1.874 | 1.375 – 2.554 |
| ****Kim et al. 2022**** | 1.847 | 1.351 – 2.525 |
| ****Lee et al. 2015**** | 1.767 | 1.303 – 2.396 |
| ****Li et al. 2015**** | 1.788 | 1.310 – 2.441 |
| ****Liu et al. 2020**** | 1.802 | 1.325 – 2.452 |
| ****Livia et al. 2016**** | 1.830 | 1.335 – 2.509 |
| ****Luo et al. 2024**** | 1.844 | 1.347 – 2.524 |
| ****Ma et al. 2023**** | 1.786 | 1.310 – 2.434 |
| ****Mansouri et al. 2019 (1)**** | 1.818 | 1.330 – 2.484 |
| ****Min et al. 2023 (3)**** | 1.777 | 1.306 – 2.418 |
| ****Mohamed et al. 2014**** | 1.760 | 1.297 – 2.387 |
| ****Nagz et al. 2020**** | 1.793 | 1.312 – 2.449 |
| ****Ottens et al. 2014**** | 1.894 | 1.393 – 2.575 |
| ****Sun et al. 2024**** | 1.776 | 1.305 – 2.419 |
| ****Takazawa et al. 2023**** | 1.880 | 1.382 – 2.557 |
| ****Verdonk et al. 2024**** | 1.861 | 1.347 – 2.571 |
| ****Wang et al. 2017**** | 1.899 | 1.401 – 2.575 |
| ****Wang et al. 2018**** | 1.901 | 1.405 – 2.573 |
| ****Wang et al. 2020**** | 1.793 | 1.312 – 2.450 |
| ****Xu et al. 2013**** | 1.888 | 1.400 – 2.546 |
| ****Xu et al. 2017**** | 1.780 | 1.308 – 2.423 |
| ****Zhang et al. 2019**** | 1.767 | 1.305 – 2.392 |
| ****Zhang et al. 2021**** | 1.882 | 1.381 – 2.565 |
| ****Zhang et al. 2015**** | 1.792 | 1.318 – 2.436 |
| ****Zhou et al. 2019 (1)**** | 1.801 | 1.319 – 2.459 |
| ****Zhou et al. 2019 (2)**** | 1.885 | 1.386 – 2.565 |
| ****Zhou et al. 2019 (3)**** | 1.840 | 1.347 – 2.513 |
| ****Combined (All Studies)**** | ****1.814**** | ****1.337 – 2.460**** |

****10.2.2** Adjuvant sedative and neuroprotective agents:Summary of Meta-Analysis Results by Model**

| ****Component**** | ****Random-Effects Model**** | ****Fixed-Effect Model**** |
| --- | --- | --- |
| ****Number of Studies**** | 40 | 40 |
| ****Pooled OR (95% CI)**** | 1.814 (1.337 – 2.460) | 1.407 (1.207 – 1.640) |
| ****Test of Overall Effect (Z, p-value)**** | 3.828, p < 0.001 | 4.371, p < 0.001 |
| ****Heterogeneity Metrics**** |  |  |
| **• Cochran's Q (p-value)** | 121.30 (p < 0.001) | 121.30 (p < 0.001) |
| **• I² (95% CI)** | 67.8% (35.4% – 80.8%) | 67.8% (53.8% – 76.1%) |
| **• Tau² (τ²)** | 0.5464 | – |
| ****Model Comparison**** |  |  |
| **• Absolute Difference in OR** | – | -0.407 |
| **• Relative Difference in OR** | – | -22.4% |

****10.2.3** Adjuvant sedative and neuroprotective agents:Analysis After Outlier Removal (IQR Method)**

| ****Parameter**** | ****Result**** |
| --- | --- |
| ****Number of Studies Included**** | 39 (1 study removed) |
| ****Pooled Odds Ratio (OR)**** | 1.888 |
| ****95% Confidence Interval**** | 1.400 – 2.546 |
| ****Test of Overall Effect**** | Z = 4.166, p < 0.001 |
| ****Heterogeneity Statistics**** |  |
| **• Cochran's Q** | 113.25 (df=38), p < 0.001 |
| **• I²** | 66.4% (95% CI: 32.7% – 79.9%) |
| **• Tau² (τ²)** | 0.5027 |

****10.3.1** Analgesic agents：Summary of Leave-One-Out Sensitivity Analysis**

| Study Omitted | Pooled Estimate (RR) | 95% Confidence Interval |
| --- | --- | --- |
| **Hashemi et al. 2013** | **1.736** | **1.117 - 2.695He et al. 2020（2）** |
| **He et al. 2020（2）** | **1.715** | **1.100 - 2.674** |
| **Mu et al. 2017** | **1.894** | **1.339 - 2.681** |
| **Wang et al. 2002** | **1.608** | **1.021 - 2.532** |
| **He et al. 2019** | **1.567** | **1.018 - 2.410** |
| **Zhu et al. 2016** | **1.615** | **1.022 - 2.545** |
| **Zhu et al. 2024** | **1.802** | **1.218 - 2.667** |
| **Yang et al. 2015（1）** | **1.637** | **1.036 - 2.584** |
| **Yang et al. 2015（2）** | **1.626** | **1.031 - 2.564** |
| **Liu et al. 2025** | **1.481** | **0.996 - 2.203** |
| **Tian et al. 2015** | **1.623** | **1.027 - 2.571** |
| **Wen et al. 2024** | **1.572** | **1.011 - 2.451** |
| **Zhu et al. 2021** | **1.572** | **1.026 - 2.410** |
| **Combined (All Studies)** |  | **1.086 - 2.494** |

****10.3.2** Analgesic agents：Summary of Meta-Analysis Results by Model**

| ****Component**** | ****Random-Effects Model**** | ****Fixed-Effect Model**** |
| --- | --- | --- |
| ****Number of Studies**** | 14 | 14 |
| ****Pooled OR (95% CI)**** | 0.521 (0.309 – 0.880) | 0.624 (0.484 – 0.803) |
| ****Test of Overall Effect (Z, p-value)**** | -2.44, p = 0.015 | -3.66, p < 0.001 |
| ****Heterogeneity Metrics**** |  |  |
| **• Cochran's Q (p-value)** | 56.71 (p < 0.001) | -3.66, p < 0.001 |
| **• I² (95% CI)** | 78.8% (64.4% – 87.4%) | 78.8% (64.4% – 87.4%) |
| **• Tau² (τ²)** | 0.6411 | – |
| ****Model Comparison**** |  |  |
| **• Absolute Difference in OR** | – | +0.103 |
| **• Relative Difference in OR** | – | +19.8% |

****10.3.3** Analgesic agents：Analysis After Outlier Removal (IQR Method)**

| ****Parameter**** | ****Result**** |
| --- | --- |
| ****Number of Studies Included**** | 13(0 studies removed by IQR method) |
| ****Pooled Odds Ratio (OR)**** | 0.521 |
| ****95% Confidence Interval**** | 0.309 – 0.880 |
| ****Test of Overall Effect**** | Z = -2.44, p = 0.015 |
| ****Heterogeneity Statistics**** |  |
| **• Cochran's Q** | 56.71 (df = 12), p < 0.001 |
| **• I²** | 78.8% (95% CI: 64.4% – 87.4%) |
| **• Tau² (τ²)** | 0.6411 |

****10.3.4 Lidocaine：**Summary of Meta-Analysis Results by Model**

| **Component** | Random-Effects Model | Fixed-Effect Model |
| --- | --- | --- |
| ****Number of Studies**** | 3 | 3 |
| ****Pooled OR (95% CI)**** | 2.634 (0.849 – 8.173) | 3.114 (1.849 – 5.245) |
| ****Test of Overall Effect**** | Z = 1.676, p = 0.094 | Z = 4.270, p < 0.001 |
| ****Heterogeneity Metrics**** |  |  |
| **• Cochran's Q (p-value)** | 9.00 (p = 0.011) | 9.00 (p = 0.011) |
| **• I² (95% CI)** | 77.8% (0.0% – 94.0%) | 77.8% (0.0% – 91.1%) |
| **• Tau² (τ²)** | 0.7758 | – |
| ****Model Comparison**** |  |  |
| **• Absolute Difference in OR** | – | 0.480 |
| **• Relative Difference in OR** | – | 18.2% |

****10.3.5 Lidocaine：Subgroup analysis forest plot****

****10.3.6 Ropivacaine：**Summary of Meta-Analysis Results by Model**

| **Component** | Random-Effects Model | Fixed-Effect Model |
| --- | --- | --- |
| ****Number of Studies**** | 2 | 2 |
| ****Pooled OR (95% CI)**** | 0.474 (0.093 – 2.423) | 0.545 (0.202 – 1.471) |
| ****Test of Overall Effect**** | Z = -0.896, p = 0.370 | Z = -1.198, p = 0.231 |
| ****Heterogeneity Metrics**** |  |  |
| **• Cochran's Q (p-value)** | 2.52 (p = 0.112) | 2.52 (p = 0.112) |
| **• I² (95% CI)** | 60.3% (0.0% – 92.1%) | 60.3% (0.0% – 90.2%) |
| **• Tau² (τ²)** | 0.8453 | – |
| ****Model Comparison**** |  |  |
| **• Absolute Difference in OR** | – | 0.070 |
| **• Relative Difference in OR** | – | 14.9% |

****10.3.7 Parecoxib：**Summary of Meta-Analysis Results by Model**

| **Component** | Random-Effects Model | Fixed-Effect Model |
| --- | --- | --- |
| ****Number of Studies**** | 3 | 3 |
| ****Pooled OR (95% CI)**** | ****0.436 (0.296 – 0.644)**** | ****0.436 (0.296 – 0.644)**** |
| ****Test of Overall Effect**** | Z = -4.177, p < 0.001 | Z = -4.177, p < 0.001 |
| ****Heterogeneity Metrics**** |  |  |
| **• Cochran's Q (p-value)** | 0.48 (p = 0.786) | 0.48 (p = 0.786) |
| **• I² (95% CI)** | 0.0% (0.0% – 19.6%) | 0.0% (0.0% – 72.9%) |
| **• Tau² (τ²)** | 0.0000 | – |
| ****Model Comparison**** |  |  |
| **• Absolute Difference in OR** | – | 0.000 |
| **• Relative Difference in OR** | – | 0.0% |

****10.3.7 Parecoxib：Subgroup analysis forest plot****

# APPENDIX 11 **Subgroup analysis of sedative drugs**

**11.1 Lidocaine: Evaluation Time**

**11.2 Parecoxib: Evaluation Period**

# APPENDIX 12 **Egger's publication bias test**

**12.1 Egger test**

| **Network (studies, n)** | **Coefficient** | **Std. err.** | **t** | **P>\|t\|** | **95 % CI** |
| --- | --- | --- | --- | --- | --- |
| Network 1 （Anesthesia Maintenance Agents，21) |  |  |  |  |  |
| slope | 0.4346 | 0.3661 | 1.19 | 0.250 | –0.3317,1.2009 |
| bias | –0.7430 | 0.7545 | –0.98 | 0.337 | –2.3221,0.8360 |
| Network 2（Adjuvant sedative and neuroprotective agents，40) |  |  |  |  |  |
| slope | –0.3825 | 0.2581 | –1.48 | 0.147 | –0.9049,0.1399 |
| bias | 1.6702 | 0.5222 | 3.20 | **0.003** | 0.6131,2.7272 |
| Network 3 （Sedative，14) |  |  |  |  |  |
| slope | –0.3713 | 0.9347 | –0.40 | 0.698 | –2.4079,1.6653 |
| bias | 1.0055 | 2.0327 | 0.49 | 0.630 | –3.4234,5.4343 |

**12.2 Network 2's patching method funnel diagram.**
